# Prognostic and therapeutic implication of m6A methylation in Crohn disease

**DOI:** 10.1097/MD.0000000000032399

**Published:** 2022-12-23

**Authors:** Yujin He, Yonghui Hu, Mei Yuan, Weiwei Xu, Yaqin Du, Jinguo Liu

**Affiliations:** a Department of Gastroenterology, Huangshi Hospital of Traditional Chinese Medicine, Hubei Chinese Medical University, Hubei, China; b Endoscopy Center, Huangshi Hospital of Traditional Chinese Medicine, Hubei Chinese Medical University, Hubei, China; c Department of Anorectal Surgery, Huangshi Hospital of Traditional Chinese Medicine, Hubei Chinese Medical University, Hubei, China; d Nephrology, Huangshi Hospital of Traditional Chinese Medicine, Hubei Chinese Medical University, Hubei, China; e The First Affiliated Hospital, Zhejiang Chinese Medical University, Zhejiang, China.

**Keywords:** Crohn disease, disease risk, immune infiltration, m6A methylation, therapeutic responses

## Abstract

N6-methyladenosine (m6A) methylation has been reported to participate in inflammatory bowel disease (including Crohn disease [CD]). However, the prognostic and therapeutic implication of m6A methylation modification in CD is still unclear.

**Methods:**

Genomic information of CD patients was integrated to assess disease-related m6A regulators, and difference and correlation analyses of m6A regulators were explored by using the R packages. Next, CD patients were classified by the expression of differential and intersecting genes in m6A regulators, and difference and correlation analyses were conducted among immune infiltration and therapeutic responses. Finally, colon tissue resected from patients with CD were assessed to verify expression of Wilms tumor 1-associated protein (WTAP) and METTL14 from these m6A regulators.

**Results:**

We identified 23 m6A regulators in CD patients. Difference analysis of these regulators showed that expression of METTL14, WTAP, RBM15 and YTHDF2/3 was upregulated in the treatment group compared with the control group, with expression of METTL3, YTHDF1, leucine-rich pentatricopeptide repeat motif-containing protein, HNRNPA2B1, IGF2BP1 and fat mass and obesity-associated protein downregulated. Moreover, RBM15, WTAP, leucine-rich pentatricopeptide repeat motif-containing protein, YTHDF1 and YTHDF3 were considered the characteristic genes of CD in m6A regulators. In addition, we identified 4 intersection genes of 3 m6A cluster patterns. Based on the expression of these intersection genes, difference analysis among m6A regulators indicated that the expression of 8 m6A regulators had statistical differences among the 3 geneCluster patterns. Assays of colon tissues from CD patients showed that expression of WTAP and METTL14 were higher in areas of stenosis than non-stenosis.

**Conclusion:**

m6A methylation modification might affect disease risk, immune infiltration and therapeutic responses in CD. Evaluating the expression of m6A regulators might provide insight into the prediction of disease prognosis and therapeutic responses.

## 1. Introduction

Crohn disease (CD) is a chronic inflammatory bowel disease (IBD) of unclear etiology that affects the gastrointestinal tract, but genetic, environmental, microbial and immunological factors are implicated in disease onset and progression.^[1,2]^ Among these, immune factors are considered a major contributor to disease process via an imbalance of various immune cells, particularly between the pro-inflammatory effects of T helper (Th) 1, Th17, neutrophils and M1 macrophages and the anti-inflammatory effects of M2 macrophages and regulatory T cells.^[3–6]^ Although treatments for CD have witnessed tremendous progress over the recent decades, ranging from mesalazine, corticosteroids and immunosuppressants to biologic agents (primarily anti-tumor necrosis factor [TNF]), the outcomes of CD are still unsatisfactory. About 30% of patients do not respond to anti-TNF agents^[7]^ and certain patients suffer the decreasing sensitivity of secondary treatment. Hence, the underlying mechanisms of CD need to be further elucidated.

N6-methyladenosine (m6A) is the most abundant internal chemical modification in higher eukaryotic messenger ribonucleic acids (mRNAs), which plays a vital role in regulating gene expressions in cellular biology.^[8]^

About 0.1 to 0.4% of total RNA adenosines are modified by m6A methylation,^[9]^ which is catalyzed by m6A “writer” proteins, a methyltransferase (MTase) complex, including MTase-like 3/14/16, Wilms tumor 1-associated protein (WTAP), RNA binding motif protein 15/15B, Virlike m6A MTase associated, zinc finger CCCH-type containing 13 and Cbl proto-oncogene like 1.^[10–12]^ The m6A demethylation is mediated by fat mass and obesity-associated protein (FTO) and alkB homolog 5 (ALKBH5) that are referred to as “eraser” proteins.^[13,14]^ On the other side, m6A methylation modification is interpreted by “reader” proteins, such as YT521-B homology family (YTHDF1–3, YTHDC1–2), embryonic lethal vision-like protein 1, heterogeneous nuclear ribonucleoprotein family (HNRNPA2B1, HNRNPC), leucine-rich pentatricopeptide repeat motif-containing protein (LRPPRC), and insulin-like growth factor 2 mRNA-binding proteins.^[15–17]^ These m6A regulators, comprised of “writers,” “eraser” and “reader,” are mainly relevant to m6A-related mRNA stability and protein translation,^[18,19]^ and involved in inflammation, tumors, innate immunity and immunotherapy.^[17,20]^ Recent studies indicate that m6A modification plays an important role in IBD, including CD,^[20,21]^ but studies focusing on CD are lacking.

Accumulating evidences reveal that m6A methylation modification is closely associated with the infiltration of immune cells. For example, m6A methylation modification modulates T cell homeostasis via the IL-7/STAT5/SOCS pathways,^[22]^ as well as METTL3 boosting the activation of dendritic cells.^[23]^ Moreover, immune infiltration is associated with therapeutic responses, which links m6A methylation to therapeutic responses. For example, ALKBH5 enhances anti-programmeddeath-1 therapy response by regulating suppressive immune cell accumulation in tumor microenvironment.^[24]^ However, the underlying mechanisms of CD-related m6A methylation embracing immune infiltration and therapeutic responses have not yet been elucidated.

## 2. Materials and Methods

### 2.1. Disease dataset source and preparation

Clinical data of CD patients, including a series matrix file and a platform file, was downloaded from the Gene Expression Omnibus (GEO) database (https://www.ncbi.nlm.nih.gov/geo/) by screening expression profiling type of *Homo sapiens*, which was filtered by sample number ≥400. Of these, the series matrix file was annotated as gene names based on the platform file and divided into the treatment and control groups based on sample characteristics.

### 2.2. Difference and correlation analyses of m6A regulators

Based on the previous studies,^[20,25]^ 28 m6A regulators, including 9 “writers,” 17 “readers,” and 2 “erasers,” were served as reference and shown in Table 1. Their gene expression was extracted from the series matrix file using the R package of Bioconductor-limma, while R packages of pheatmap, reshape2 and ggpubr were used to distinguish differentially expressed genes of m6A regulators between the treatment and control groups. In addition, the locations of m6A regulators on chromosomes were determined using the Perl Programming Language. Moreover, the expression of m6A regulators was used to conduct correlation analysis of pairwise comparison via |related coefficient| > 0.4 and *P* < .001 using the R packages of limma, ggplot2, ggpubr and ggExtra.

**Table 1:** m6A regulators.

Gene	Type	Gene	Type
METTL3	Writer	ELAVL1	Reader
METTL14	Writer	HNRNPC	Reader
METTL16	Writer	HNRNPA2B1	Reader
WTAP	Writer	LRPPRC	Reader
VIRMA	Writer	FMR1	Reader
ZC3H13	Writer	IGFBP1	Reader
RBM15	Writer	IGFBP2	Reader
RBM15B	Writer	IGFBP3	Reader
CBLL1	Writer	IGF2BP1	Reader
YTHDF1	Reader	IGF2BP2	Reader
YTHDF2	Reader	IGF2BP3	Reader
YTHDF3	Reader	RBMX	Reader
YTHDC1	Reader	ALKBH5	Eraser
YTHDC2	Reader	FTO	Eraser

ALKBH5 = AlkB homolog 5, CBLL1 = Cbl proto-oncogene like 1, ELAVL1 = embryonic lethal vision-like protein 1, HNRNPC = heterogeneous nuclear ribonucleoprotein family, FTO = fat mass and obesity-associated protein, LRPPRC = leucine-rich pentatricopeptide repeat motif-containing protein, m6A = N6-methyladenosine, WTAP = Wilms tumor 1-associated protein, ZC3H13 = zinc finger CCCH-type containing 13.

### 2.3. Model selection for screening disease characteristic genes

Differential genes of m6A regulators were used to screen the characteristic genes of CD through methods of trees in random forest (RF) and support vector machine (SVM) using R packages of caret, DALEX, ggplot2, randomForest, kernlab and pROC. By defining the prediction function, the prediction results of RF and SVM models were obtained to draw the boxplot and reverse cumulative distribution diagram of |residual|, as well as a receiver operating characteristic (ROC) curve. Based on these results, the optimal model was selected to screen the characteristic genes of CD by using features with the highest mean decrease in Gini index (mean decrease Gini). In addition, a nomogram of these characteristic genes, as well as calibration, decision and clinical impact curves, were drawn by using R packages of rms and rmda.

### 2.4. Sample classification based on differential expression of m6A regulators

After removing the control group, differential expression of m6A regulators were administered using the R-package of ConsensusClusterPlus,^[20]^ with the *k* value identified by the clustering results. The optimal *k* value of consensus matrix was used to classify samples in the treatment group. Moreover, the expression of m6A clusters was used to draw a heatmap and boxplot, and conduct principal component (PCA) analysis via the R packages of limma, pheatmap, reshape2, ggpubr and ggplot2.

### 2.5. Difference and correlation analyses of immune cells

A series of immune cells were differentially analyzed by using the R packages of limma, GSEABase, GSVA and ggpubr, which evaluated the proportion of immune cell infiltration among different m6Acluster types. Their outcomes were used to perform correlation analysis and a heatmap was drawn for display. The gene with the most significant correlation was selected based on the heatmap analysis, and it was used to divide CD patients into high and low expression groups for further analysis via the R packages of pheatmap, reshape2 and ggpubr.

### 2.6. Enrichment and difference analyses of intersection genes

Intersection genes of different m6Acluster types were obtained through the R packages of limma and VennDiagram, which were used to perform Gene Ontology and Kyoto Encyclopedia of Genes and Genomes analyses. In addition, the sample classification of intersection genes was similar to the classification method of m6A regulators based on the optimal *k* value. Moreover, difference analyses of geneClusters and immune infiltration were conducted to evaluate the differential expression of intersection genes.

### 2.7. Difference analysis of m6A score and therapeutic responses

Differential gene expression of m6A regulators was scored by PCA analysis with the commands of prcomp and predict, which was used to perform differential analysis among m6Aclusters and geneClusters via the R packages of limma and ggpubr. Moreover, these datasets were divided into high and low groups based on the m6A score, and an alluvial diagram was drawn by using the R packages of ggplot2 and ggalluvial. In addition, the differential expressions of m6Aclusters and geneClusters at the response genes (TNF, IL4, IL5, IL13, IL25, IL33 and IDO1) were analyzed by the R packages of limma, reshape2 and ggpubr.

### 2.8. Experimental design

Surgically resected colon segments from narrow and non-narrow segments were collected from 8 CD patients in the First Affiliated Hospital of Zhejiang Chinese Medical University (Hangzhou, China).^[26]^ All patients provided written and oral informed consent and the study protocol was approved by the Institutional Ethics Committee of Zhejiang Chinese Medical University (2021-KL-209-01).

### 2.9. Hematoxylin-eosin (HE) staining

Human colon tissues were fixed with 10% formalin, embedded in paraffin, cut into 4 μm-thick slices and stained with HE (Solarbio, China) according to standard methods. The stained slices were inspected using a light microscope (Olympus, Japan).

### 2.10. Western blotting assay

Proteins were extracted from colon tissues of CD patients using radioimmunoprecipitation assay lysis buffer with phenylmethanesulfonylfluoride and a protease and phosphatase inhibitor cocktail (Solarbio, China). After protein concentrations were quantified, they were balanced with SDS-PAGE sample loading buffer (Beyotime). Protein samples were transferred to polyvinylidene fluoride membranes (General Electrics Healthcare, USA), blocked with 5% nonfat milk for 1 hour, and incubated overnight at 4°C with the following primary antibodies: rabbit anti-WTAP (1:1000, Abcam, UK) and rabbit anti-METTL14 (1:1000, Abcam). Mouse anti-β-actin (1:5000, Proteintech) and rabbit anti-glyceraldehyde-3-phosphate dehydrogenase were considered as internal references. After washing 3 times with tris-buffered saline containing tween 20, the membranes were incubated with the appropriate horseradish peroxidase-labeled goat secondary antibodies (1:5000, Thermo Fisher Scientific, USA) at room temperature for 1 hour. The membranes were incubated with enhanced chemiluminescence solution (Applygen, China) for 1 minute and visualized using a chemiluminescence imager (Clinx Science Instruments, China).

### 2.11. Immunohistochemistry

Paraffin-embedded colon tissue slices (4 μm) were deparaffinized in xylene and rehydrated through a series of gradient ethanol and tap water. The rehydrated tissue slices were immersed in sodium citrate buffer (10 mM sodium citrate, pH 6.0) (Servicebio, China) and microwaved for antigen retrieval. The tissue slices were soaked in 3% H_2_O_2_ (Dawen Biotec, China), blocked with 5 % bovine serum albumin and then incubated overnight at 4°C with rabbit anti-WTAP (1:500, Abcam) and rabbit anti-METTL14(1:500, Abcam) antibodies. The tissue slices were incubated with horseradish peroxidase-labeled anti-rabbit antibodies (1:500, Thermo Fisher Scientific), reacted with diaminobezidin chromogenic solution (Biosharp, China), counterstained with hematoxylin and inspected under a light microscope.

### 2.12. Statistical analysis

Analyses were conducted by the *R*-4.1.1 software and Statistical Package for the Social Sciences (SPSS) 21.0 software (IBM Corp, Armonk, NY). Results were expressed as mean ±  standard error of mean. Two-group comparisons were performed with 2-tailed *t* tests. Multigroup comparisons were performed with one-way analysis of variance, with pairwise comparisons assessed by least significant difference test. All *P* values were 2-sided, with *P* < .05 considered statistically significant. Digital images were quantified by Image-Pro Plus image analysis software (Media Cybernetics, USA).

## 3. Results

### 3.1. Landscape of m6A regulators in patients with CD

**Figure 1.**
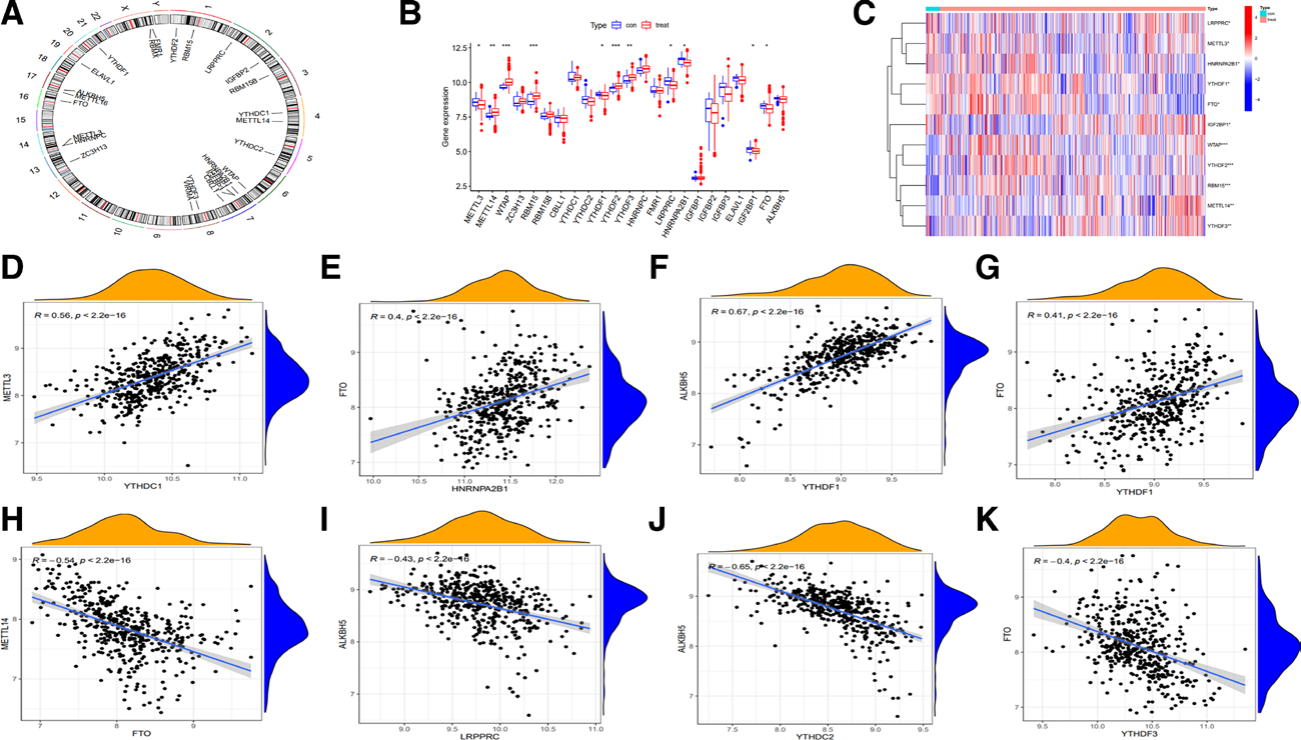
Landscape of m6A regulators in CD patients. (A) The location of m6A regulators on chromosomes. (B) Boxplot and (C) heatmap of difference analysis among m6A regulators. (D–G) Positive correlation analyses of (D) YTHDC1-METTL3, (E) HNRNPA2B1-FTO, (F) YTHDC1-ALKBH5, and (G) YTHDC1-FTO. (H–K) Negative correlation analyses of (H) FTO-METTL14, (I) LRPPRC-ALKBH5, (J) YTHDC2-ALKBH5, and (K) YTHDF3-FTO. ALKBH5 = AlkB homolog 5, FTO = fat mass and obesity-associated protein, LRPPRC = leucine-rich pentatricopeptide repeat motif-containing protein, m6A = N6-methyladenosine.

The series matrix file (GSE186582, n = 489) was enrolled in our study and annotated by the platform file (GPL570), and 23 m6A regulators, including 7 “writers,” 14 “readers,” and 2 “erasers,” were identified by the annotated matrix file. Their locations on chromosomes were shown in Figure 1A and Table S1, Supplemental Digital Content, http://links.lww.com/MD/I191. Difference analysis of m6A regulators (Fig. 1B and C, Table S2, Supplemental Digital Content, http://links.lww.com/MD/I192) showed that the mRNA expression of 5 m6A regulators, including METTL14, WTAP, RBM15 and YTHDF2/3, was upregulated in the treatment group compared with the control group (*P* < .05), especially WTAP, RBM15 and YTHDF2 (*P* < .001), whereas the expression of 6 regulators, including METTL3, YTHDF1, LRPPRC, HNRNPA2B1, IGF2BP1 and FTO, was downregulated (*P* < .05). Additionally, the outcomes of correlation analysis were shown in Table 2, indicating that the relationships of YTHDC1-METTL3/ ALKBH5/ FTO and HNRNPA2B1-FTO showed a positive correlation (Fig. 1D–G), with FTO-METTL14, LRPPRC/ YTHDC2-ALKBH5 and YTHDF3-FTO showing a negative correlation (Fig. 1H–K). These analyses revealed that m6A regulators performed expression alteration between the 2 groups, suggesting that the expression of different m6A regulators might affect the progression of CD.

**Table 2:** Correlation analysis of m6A regulators.

Gene1	Gene2	Related coefficient (R)	*P* value
YTHDC1	METTL3	0.562859431231653	3.94427030092717e–40
HNRNPA2B1	FTO	0.400076802025084	2.90827987016664e–19
YTHDF1	ALKBH5	0.673647923540975	1.16894308590444e–62
YTHDF1	FTO	0.414670521969699	1.04268048860219e–20
FTO	METTL14	−0.541146351962563	1.18171332137109e–36
LRPPRC	ALKBH5	−0.426121525396087	6.81230281372042e–22
YTHDC2	ALKBH5	−0.650317462543585	3.99726843885228e–57
YTHDF3	FTO	−0.400951739919633	2.39313576237165e–19

ALKBH5 = AlkB homolog 5, FTO = fat mass and obesity-associated protein, LRPPRC = leucine-rich pentatricopeptide repeat motif-containing protein, m6A = N6-methyladenosine.

### 3.2. Screening the characteristic genes of m6A regulators in CD

**Figure 2.**
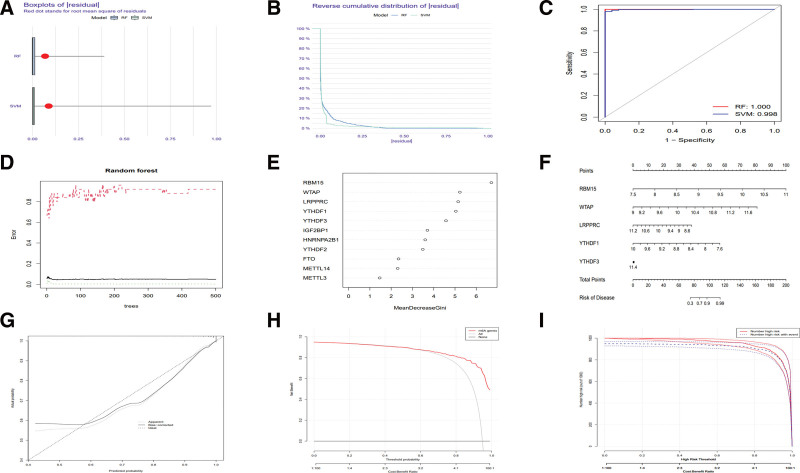
Model selection to screen disease characteristic genes and evaluate disease risk. (A) Boxplot of |residual|. (B) Reverse cumulative distribution diagram of |residual|. (C) ROC curves of RF and SVM. (D) RF model. (E) The score of 11 genes with the highest Mean Decrease Gini. (F) Nomogram of 5 genes with the highest importance scores. (G) Calibration curve. (H) Decision curve. (I) Clinical impact curve. RF = random forest, ROC = receiver operating characteristic, SVM = support vector machine.

The boxplot and reverse cumulative distribution diagram of |residual| showed that both RF and SVM were considered effective screening methods due to their small |residual| values (Fig. 2A and B), with the ROC curve showing a larger area (1.0 vs 0.998) under curve in RF (Fig. 2C). Hence, the RF model was chosen to screen the characteristic genes (Fig. 2D), with the red, green and black curves indicating error of the treatment, control and all groups, in which the least error tree was used to rebuild the RF model and view the importance score of genes with the highest mean decrease Gini (Fig. 2E). The 5 genes with the highest importance scores, including RBM15, WTAP, LRPPRC, YTHDF1 and YTHDF3, were considered the characteristic genes of CD in m6A regulators, whose expression was determined to draw a nomogram that was used to assess risk of disease in patients with CD (Table S3, Supplemental Digital Content, http://links.lww.com/MD/I193, Fig. 2F). The calibration curve showed that the bias-corrected line was close to the apparent line (Fig. 2G), the decision curve indicated a great difference between the lines (Fig. 2H) and the clinical impact curve indicated a similarity of curves between the high-risk patients predicted by the RF model and actual high-risk patients (Fig. 2I). These curves indicated a high accuracy of the RF model.

### 3.3. m6A modification patterns mediated by 11 differential m6A regulators

**Figure 3.**
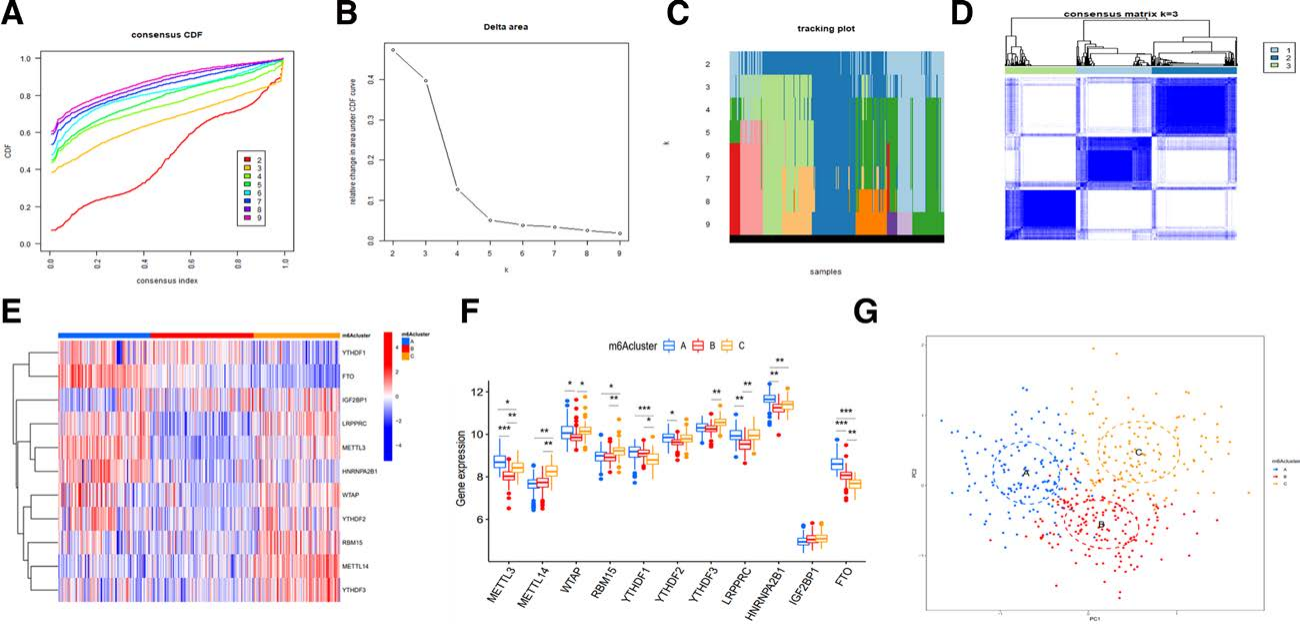
Differential m6A regulators among m6Acluster patterns in CD. (A–D) Sample classification according to the expression of m6A regulators, showing (A, B) relative changes in area under CDF curve, (C) tracking plot, and (D) the optimal *k* value (*k* = 3). (E) Heatmap of m6Aclusters. (F) Boxplot of m6Aclusters. (G) PCA analysis. CD = Crohn disease, CDF = cumulative distribution function, m6A = N6-methyladenosine, PCA = principal component.

The *k* value (*k* = 2–9) was determined by the proportion of ambiguous clustering measurement and the similarity of 11 m6A regulator expression (Fig. 3A–C), and *k* = 3 was identified as the optimal *k* value by the most significant relative change in area under consensus cumulative distribution function curve, which was used to classify samples in the treatment group, including 152 cases in m6Acluster A, 170 cases in m6Acluster B, and 142 cases in m6Acluster C (Fig. 3D and Table S4, Supplemental Digital Content, http://links.lww.com/MD/I194). Moreover, the heatmap of m6Aclusters showed that the expression of YTHDF1/2, FTO, LRPPRC, METTL3, HNRNPA2B1 and WTAP was upregulated in m6Acluster A, with IGF2BP1, RBM15, METTL14 and YTHDF3 downregulated; the expression of YTHDF1 and IGF2BP1 was upregulated in m6Acluster B, with YTHDF2/3, LRPPRC, METTL3/14, HNRNPA2B1, WTAP and RBM15 downregulated; and the expression of IGF2BP1, LRPPRC, METTL3, WTAP, YTHDF2/3, RBM15 and METTL14 was upregulated in m6Acluster C, with YTHDF1 and FTO downregulated (Fig. 3E). Moreover, the boxplot showed that the expression of METTL3, YTHDF1, HNRNPA2B1 and FTO all decreased in m6Acluster B and C, whereas METTL14 and IGF2BP1 both increased, compared with m6Acluster A, with YTHDF3 and LRPPRC decreasing in m6Acluster B and increasing in m6Acluster C; the expression of WTAP and YTHDF2 decreased in m6Acluster B compared with m6Acluster A and C, while RBM15 increased in m6Acluster C, compared with m6Acluster A and B (Fig. 3F). In addition, the PCA analysis indicated that samples of different groups could be distinguished by the expression of m6A regulators (Fig. 3G).

### 3.4. m6A modification patterns regulating immune infiltration in CD

**Figure 4.**
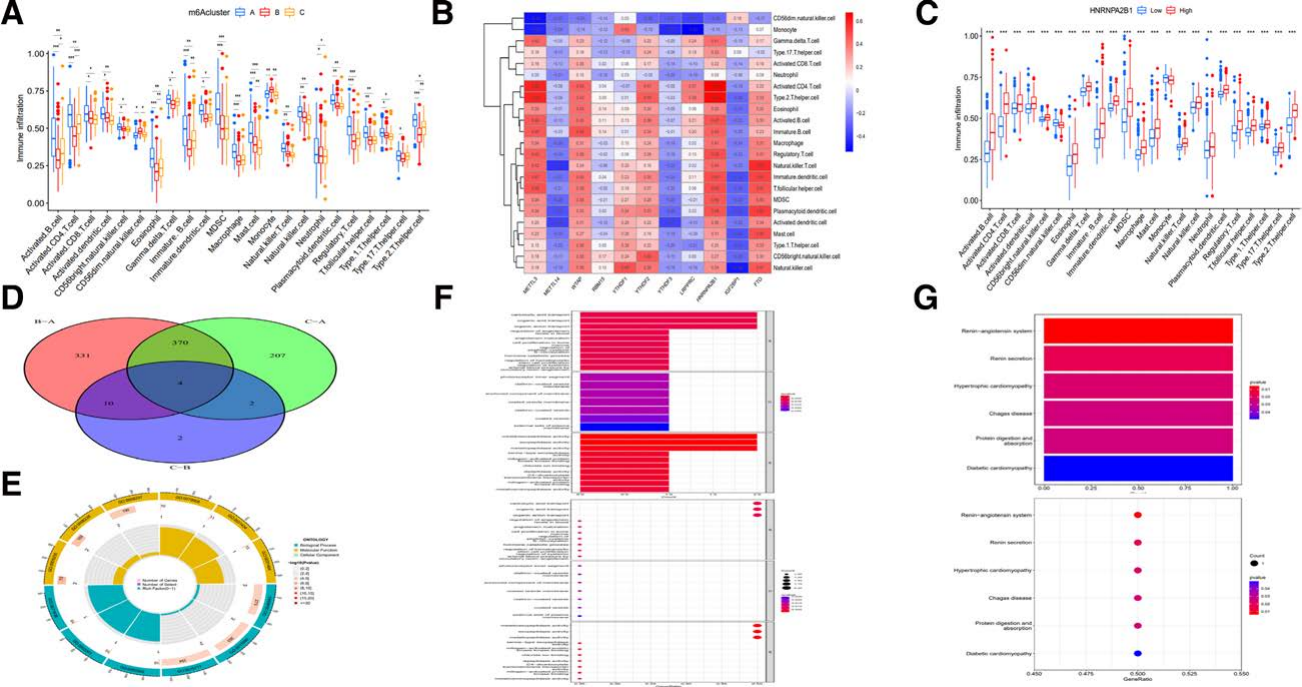
Immune infiltration and intersection genes among different m6Acluster patterns. (A,B) (A) Difference and (B) correlation analyses among immune cell infiltration. (C) Sample classification based on the expression of HNRNPA2B1. (D) Intersection genes of different m6Acluster patterns. (E) Circle graph of GO enrichment analysis. The outermost circle represents the ID of GO analysis, with the second circle indicating the number of genes on each GO analysis and the third circle indicating the number of intersection genes on each GO analysis. (F) GO enrichment analysis of intersection genes. (G) KEGG enrichment analysis of intersection genes. GO = Gene Ontology, KEGG = Kyoto Encyclopedia of Genes and Genomes, m6A = N6-methyladenosine.

Differences of immune cell infiltration were conducted by using the expression of immune cells in patients with CD (Table S5, Supplemental Digital Content, http://links.lww.com/MD/I195 and Table S6, Supplemental Digital Content, http://links.lww.com/MD/I196). The boxplot of difference analysis showed that activated B cell, immature B cell, activated CD4 T cell, eosinophil, mast cell and type 2 Th cell had statistical differences among 3 m6Acluster patterns, and various immune cells decreased in m6Acluster B and C, except for monocyte and CD56dim natural killer cell, compared with m6Acluster A (Fig. 4A). The heatmap of correlation analysis indicated that METTL3, WTAP, YTHDF2, HNRNPA2B1 and FTO were mainly upregulated among the expression of immune cells, especially HNRNPA2B1, whereas METTL14, YTHDF3 and IGF2BP1 were downregulated (Fig. 4B). In addition, HNRNPA2B1 was considered as the most significant gene in correlation analysis, and the expression of different immune cells mainly increased in its high expression group, except for CD56dim natural killer cell and monocyte (*P* < .01) (Fig. 4C). The significant differences of immune infiltration among 3 m6Aclusters suggested that m6A methylation modifications might alter the mucosal immune microenvironment of CD.

### 3.5. Consensus clustering for m6A phenotype-related intersection genes in CD

**Figure 5.**
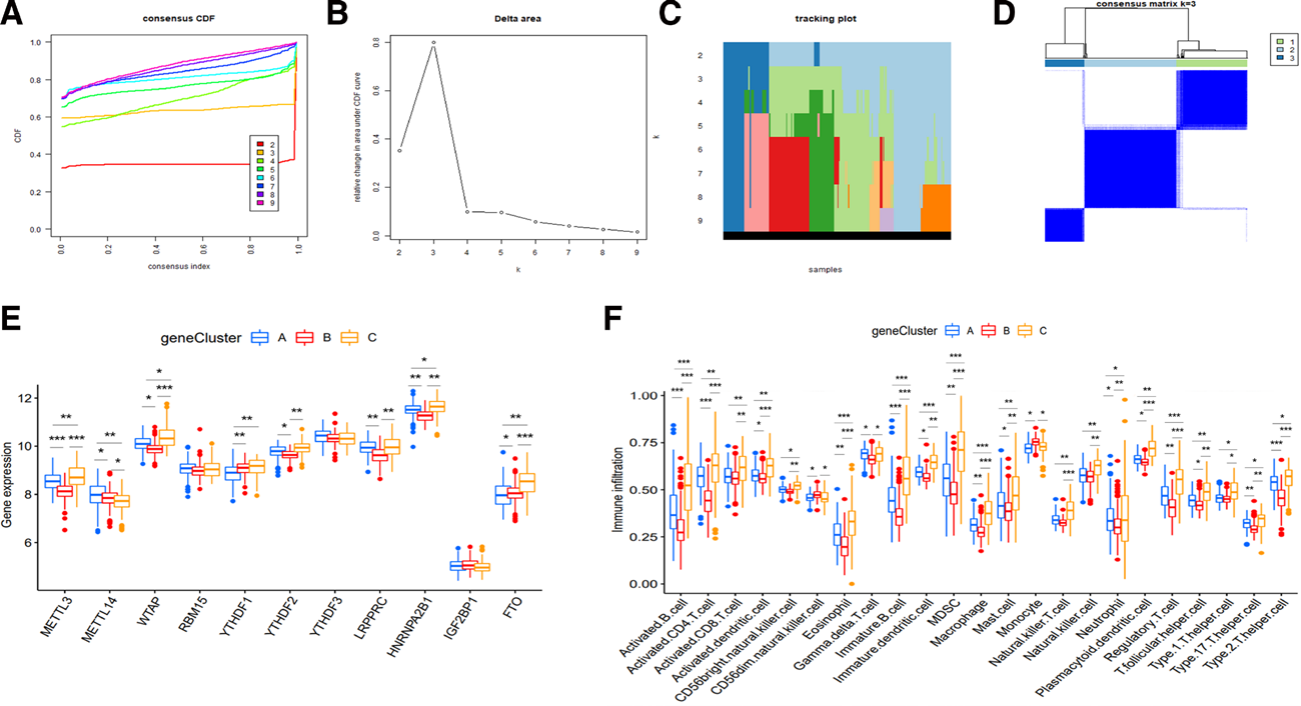
Difference analysis of m6A regulators and immune infiltration based on the expression of intersection genes. (A–D) Sample classification according to the expression of intersection genes. (E) Difference analysis of m6A regulators among different geneCluster patterns. (F) Difference analysis of immune infiltration among different geneCluster patterns. m6A = N6-methyladenosine.

Intersection genes of different m6Acluster types included XPNPEP2, REEP6, ACE and SLC13A2 (Fig. 4D, Table S7, Supplemental Digital Content, http://links.lww.com/MD/I197). Gene Ontology enrichment analysis mainly involved the transport of carboxylic acid, organic acid and organic anion, as well as the activities of metalloexopeptidase, exopeptidase and metallopeptidase (Fig. 4F), and the number of enriched genes with different functions was shown in Figure 4E and Table S8, Supplemental Digital Content, http://links.lww.com/MD/I198. Kyoto Encyclopedia of Genes and Genomes enrichment analysis showed that the signaling pathways were mainly associated with renin-angiotensin system, renin secretion, hypertrophic cardiomyopathy, chagas disease, protein digestion and absorption, and diabetic cardiomyopathy (Fig. 4G and Table S9, Supplemental Digital Content, http://links.lww.com/MD/I199). In addition, the classification of intersection genes embraced the same *k* value of m6Aclusters, including 162 cases in geneCluster A, 211 cases in geneCluster B and 91 cases in geneCluster C (Fig. 5A–D, Table S10, Supplemental Digital Content, http://links.lww.com/MD/I200).

### 3.6. m6A phenotype-related intersection genes associated with m6A methylation and immune infiltration

Difference analysis among m6A regulators indicated that the expression of METTL3/14, WTAP, HNRNPA2B1 and YTHDF3 decreased in geneCluster B, whereas METTL3, WTAP, HNRNPA2B1 and YTHDF1 increased in geneCluster C, compared with geneCluster A; the expression of YTHDF2 and LRPPRC both increased in geneCluster A and C, compared with geneCluster B (Fig. 5E). Moreover, difference analysis among infiltration of immune cells indicated that the expression of different immune cells mainly decreased in geneCluster B and increased in geneCluster C, compared with geneCluster A; CD56dim natural killer cell and monocyte decreased in geneCluster A and C, whereas γδ T cell increased in geneCluster A and C, compared with geneCluster B; CD56bright natural killer cell decreased in geneCluster A and B, compared with geneCluster C (Fig. 5F).

### 3.7. Difference analysis of m6A score and therapeutic responses

**Figure 6.**
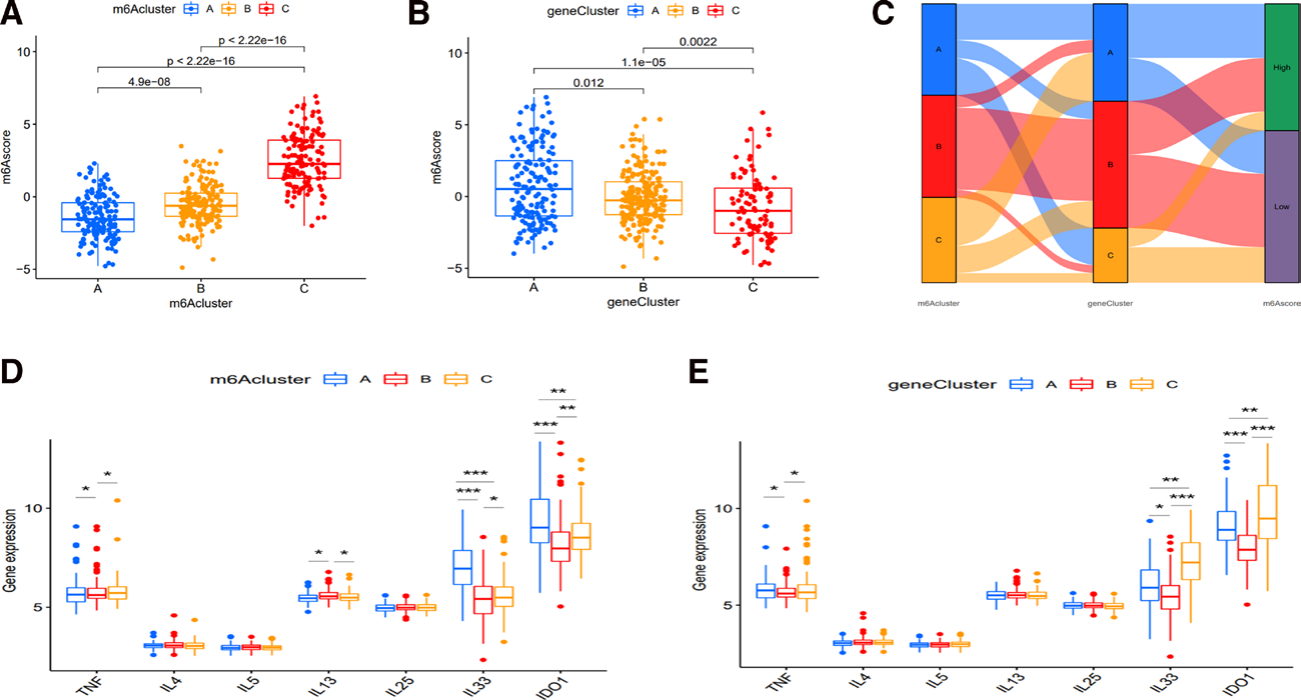
Difference analysis of m6A score and therapeutic responses. (A) Difference analysis between m6Ascore and m6Aclusters. (B) Difference analysis between m6Ascore and geneClusters. (C) Alluvial diagram showing the changes of m6Aclusters, geneClusters and m6Ascore. (D, E) Difference analysis of therapeutic responses among different (D) m6Acluster and (E) geneCluster patterns. m6A = N6-methyladenosine.

The score of samples with differential gene expression of m6A regulators was shown in Table S11, Supplemental Digital Content, http://links.lww.com/MD/I201. Difference analysis between m6Ascore and m6Aclusters or geneClusters showed that m6Ascore was statistically significant among the 3 groups (Fig. 6A and B). As shown in Figure 6C, the patients in m6Acluster B subtype were classified into geneCluster B, which mainly involved the low score group, as well as subtype C, whereas the subtype A mainly involved the high score group. Moreover, difference analyses of therapeutic responses showed that TNF, IL33, IDO1 had statistical differences among m6Acluster and geneCluster groups, as well as IL13 statistical difference among m6Aclusters (Fig. 6D and E), suggesting that the expression of m6A regulators and m6A-related intersection genes might affect the therapeutic effect of anti-TNF, IL13, IL33 and IDO1.

### 3.8. Pathological changes and expression of WTAP and METTL14 in CD patients

**Figure 7.**
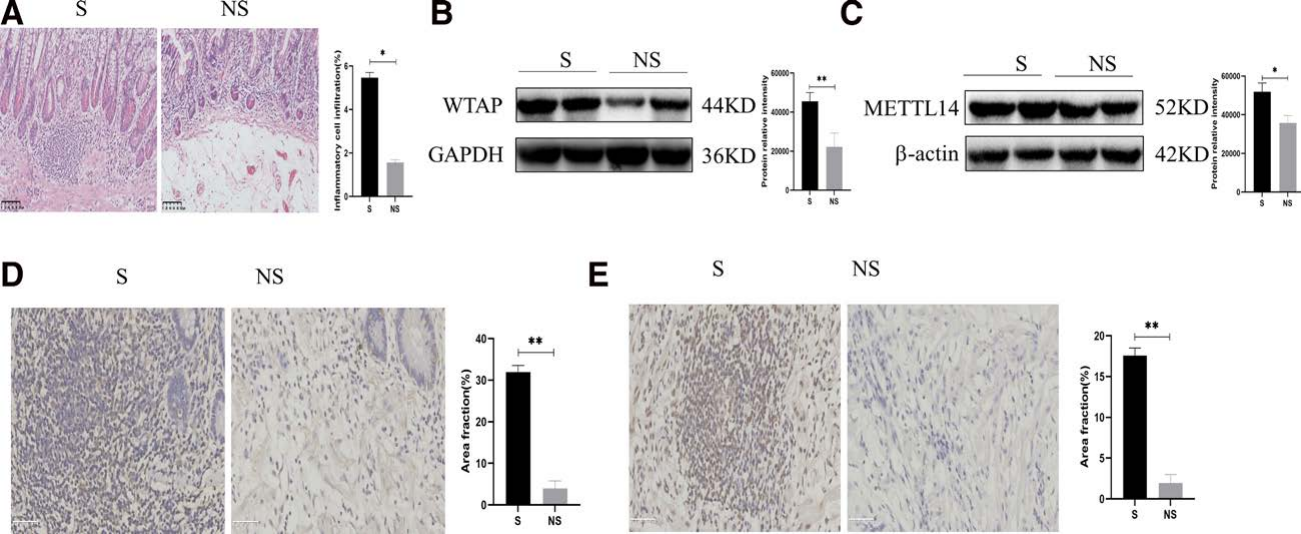
Pathological changes and expression of WTAP and METTL14 in CD patients. (A) HE staining. Scale bar: 100 µm, magnification: ×200. (B,C) Western blotting showing the relative protein intensities of (B) WTAP and (C) METTL14. (D, E) Immunohistochemistry of (D) WTAP and (E) METTL14. Scale bars: 50 µm, magnification: ×400. S: stenosis, NS: non-stenosis. *P < .05, **P < .01, ***P < .001, and ****P < .0001. CD = Crohn disease, HE = hematoxylin-eosin, WTAP = Wilms tumor 1-associated protein.

Pathologic evaluation of human colon specimens by HE staining (Fig. 7A) showed that inflammatory cell infiltration was greater in areas of stenosis than non-stenosis, as well as collagen deposition greater in stenosis.^[26]^ Western blotting of colonic specimens showed that the levels of WTAP and METTL14 proteins were higher in areas of stenosis than non-stenosis (Fig. 7B and C). Moreover, immunohistochemistry showed that the levels of WTAP and METTL14 were also higher in strictures (Fig. 7D and E). Taken together, these findings indicated that inflammation and fibrosis in patients with CD was closely associated with expression of WTAP and METTL14.

## 4. Discussion

Most studies on m6A methylation modification are limited to various cancers, focusing on single regulator or single immune cell type, the relationships between multiple m6A regulators and immune infiltration on CD progression are poorly understood. Clarifying these relationships may provide insight into our understanding of prognostic implications and therapeutic responses in CD.

m6A regulators were associated with onset and progression of inflammation. For example, deletion of METTL14 (a “writer” gene) in T cells induced spontaneous colitis in mice.^[27]^ ALKBH5 (an “eraser” gene) preserved the ability of naïve CD4^+^ T cells to induce colitis.^[28]^ In addition, FTO, an “eraser” gene, was considered a novel genetic marker to predict the response or toxicity of thiopurine therapy in patients with CD.^[29]^ Carriers of obesity-associated FTO variant rs9939609 AA genotype indicated an increased risk of CD.^[30]^ In the present study, we found that remarkable differences and correlations existed among “writers,” “readers” and “erasers.” Moreover, m6A methylation was catalyzed by “writers,” and its demethylation was mediated by “erasers,” indicating that their trends might be opposite. However, interestingly, we found that the expression of METTL3 (a “writer” gene) and FTO was downregulated in the treatment group, while METTL3, WTAP and FTO were upregulated among the expression of immune cells. Moreover, METTL3, WTAP and FTO also increased in m6Acluster A, with RBM15 and METTL14 decreasing, whereas METTL3/14, WTAP and RBM15 increased in m6Acluster C, with YTHDF1 and FTO decreasing. As oncogenes, METTL14 and KIAA1429 (“writer” genes) were found to increase the RNA m6A level,^[31,32]^ with FTO and ALKBH5 decreasing the RNA m6A level.^[33,34]^ These findings suggested that multiple mechanisms might exist in disease development. In addition, m6A “readers” involved post-transcriptional functions to interpret m6A methylation modification, which might partly clarify the seemingly contradictory relationships between them.^[20]^

IBD-related colorectal cancer (CRC) was approximately 2% in the mortality of CRC, but it was 10 to 15% in IBD patients.^[35]^ However, the risk of CD-related CRC was more contentious. Previous studies reported that this risk was 2.5- to 4.5-fold higher than healthy subjects,^[36,37]^ whereas it had changed over time without an increased risk of CRC followed by CD diagnosis.^[38,39]^ Enterotoxigenic *Bacteroides fragilis* promoted the proliferation of CRC cell by downregulating METTL14-mediated miR-149-3p,^[40]^ and knockdown RBM15 (a “writer” gene) suppressed the proliferation and metastasis of CRC cell via m6A methylation of MyD88 mRNA.^[41]^ Moreover, the upregulated expression of WTAP (a “writer” gene) in CRC involved tumor site and differentiation,^[42]^ and the expression of LRPPRC (a “reader” gene) in CRC tissue was 1.5-fold higher than in normal tissues.^[43]^ In addition, YTHDF1 (a “reader” gene) promoted the translation of m6A-Rho/Rac guanine nucleotide exchange factor 2 in CRC,^[44]^ with YTHDF3 embracing a negative function to regulate CRC-related long non-coding RNA growth arrestspecific 5.^[45]^ However, the underlying mechanisms by which m6A regulators regulate the development of CD and CRC remain unclear.

m6A regulators were found to play crucial roles in immune cell infiltration.^[20]^ Immune cells contributing to IBD progression mainly involved M1/M2 macrophages, neutrophils and T cells.^[3,5,6,20]^ METTL3 was potentially considered as an anti-inflammatory target to drive M1 macrophage polarization,^[46,47]^ with YTHDF2 regulating lipopolysaccharide-induced inflammatory responses in macrophages^[48]^ and FTO silencing inhibiting both M1 and M2 polarization via YTHDF2 involvement.^[49]^ Moreover, METTL14 induced spontaneous colitis via induction of naïve T cells into Treg cells.^[27]^ In addition, the high expression of HNRNPA2B1 (a “reader” gene) and KIAA1429, characterized by high Th2 cell infiltration and low infiltration of Th17 cell and M1/M2 macrophages, was associated with a poor prognosis in prostate cancer.^[50]^ HNRNPA2B1 and HNRNPC (a “reader” gene) might affect the infiltration of immune cells in endometriosis.^[51]^ In the present study, we found that difference analysis among 3 m6Acluster patterns involved acquired immune cells, such as activated B cell, immature B cell, activated CD4 T cell, and type 2 Th cell, and innate immune cells, such as eosinophil and mast cell. Moreover, the expression of METTL3, WTAP, YTHDF2, HNRNPA2B1 and FTO increased among immune cells, with the expression of METTL14, YTHDF3 and IGF2BP1 decreasing. Eosinophil, M2 macrophage and neutrophil were positively correlated with the risk score of CRC based on a m6A-related prognostic model.^[52]^ However, our study on m6A regulators were limited to bioinformatics analysis, and its underlying mechanisms needed to be explored by which these m6A regulators induced immune cell infiltration in CD.

Next, we identified 4 intersection genes from m6Acluster types. XPNPEP2 was one of the critical genes in thyroid cancer targeted by miR-222-3p,^[53]^ and it was associated with lymph node metastasis in prostate and cervical cancers.^[54,55]^ REEP6, an accessory protein, refined CXCR1-mediated cellular responses in lung cancer.^[56]^ Serum ACE levels in IBD patients were lower than in healthy controls, regardless of the genotype of ACE.^[57]^ SLC13A2 involved liver metastases in CRC patients with an upward trend.^[58]^ Moreover, the expression of METTL3/14, WTAP, HNRNPA2B1 and FTO had significant differences between 3 geneCluster types. Few reports exist in the association of these genes with CD. Our study indicates that these genes may be the key targets of m6A regulators, which may trigger new therapeutic strategies for CD. However, further studies are needed to elucidate their roles in CD development.

m6A regulators were also associated with immunotherapy.^[20]^ TNF-α mediated by METTL14 increased the expression of engulfment and cell motility protein 1,^[59]^ and METTL14 also played a major role in TNF-α-induced endothelial cell inflammation.^[60]^ In addition, METTL3-deficient macrophages exhibited reduced TNF-α production with lipopolysaccharide stimulation.^[61]^ In the present study, we found that TNF, IL13, IL33 and IDO1 had statistical differences among m6Acluster and geneCluster patterns, suggesting that different expression of m6A regulators and intersection genes might affect these therapeutic responses. Induction of IDO-1 by immunostimulatory DNA inhibited severity of colitis.^[62]^ Sprouty2 loss leaded to epithelial IL-33 expression, which increased stromal IL-13+ cells and in turn induced intestinal tuft and goblet cell expansion.^[63]^ In addition, the biological agent anti-TNF (primarily infliximab) was often applied for the treatment of refractory and severe CD,^[64]^ which was also recommended to treat CD patients with surgically induced remission with or without thiopurines.^[65]^ However, our study focused on bioinformatics with retrospective clinical data, and the predictive value of these targets should be further verified.

## 5. Conclusion

We identified differences and correlations among m6A regulators, disease risk, immune infiltration, and therapeutic responses in patients with CD. The expression of m6A-related genes in CD could be used to assess disease risk and therapeutic responses to biologic agents.

## Author contributions

YH, WX,YD, and JL participated in research design. YH, MY, YH, and JL performed data analysis. YH and JL wrote or contributed to the writing of the manuscript.

**Conceptualization:** Jinguo Liu.

**Formal analysis:** Yujin He.

**Methodology:** Yaqin Du.

**Resources:** Weiwei Xu.

**Software:** Yonghui Hu.

**Validation:** Mei Yuan.

## Supplementary Material
