# Supplementary material for: Prognostic and therapeutic implication of m6A methylation in Crohn disease
Source: Medicine (Baltimore). 2022 Dec 23;101(51):e32399. doi: 10.1097/MD.0000000000032399 (PMC9794314; doi:10.1097/MD.0000000000032399)
Supplement: Supplementary file 1 [file medi-101-e32399-s001.pdf]

Supplemental Table 1. The location of m6A regulators on chromosomes

Chromosome	ChromStart	ChromEnd	Gene	Chromosome	ChromStart	ChromEnd	Gene
chr1	28736621	28769775	YTHDF2	chrX	136848004	136880764	RBMX
chr1	110338506	110346681	RBM15	chrX	147911951	147951125	FMR1
chr2	43886508	43996005	LRPPRC	chr8	63168553	63212786	YTHDF3
chr2	216632828	216664436	IGFBP2	chr8	94487693	94553529	VIRMA
chr3	51391268	51397908	RBM15B	chr13	45954465	46052759	ZC3H13
chr4	68310387	68350089	YTHDC1	chr14	21209136	21269494	HNRNPC
chr4	118685368	118715433	METTL14	chr14	21498133	21511375	METTL3
chr5	113513683	113595285	YTHDC2	chr16	53703963	54121941	FTO
chr6	159725585	159756319	WTAP	chr17	2405562	2511891	METTL16
chr7	26189927	26201529	HNRNPA2B1	chr17	18183078	18209954	ALKBH5
chr7	45888357	45893668	IGFBP1	chr17	48997412	49055650	IGF2BP1
chr7	45912245	45921874	IGFBP3	chr19	7958579	8445041	ELAVL1
chr7	107743697	107761667	CBLL1	chr20	63195429	63216234	YTHDF1
