# Supplementary material for: Prognostic and therapeutic implication of m6A methylation in Crohn disease
Source: Medicine (Baltimore). 2022 Dec 23;101(51):e32399. doi: 10.1097/MD.0000000000032399 (PMC9794314; doi:10.1097/MD.0000000000032399)
Supplement: Supplementary file 2 [file medi-101-e32399-s002.pdf]

Supplemental Table 2. Differential gene expression of m6A regulators

ID	METTL3	METTL14	WTAP	RBM15	YTHDF1	YTHDF2	YTHDF3	LRPPRC	HNRNPA2B1	IGF2BP1	FTO
GSM5656180_con	8.416177	7.688959	9.490316	8.195646	8.933446	9.603287	9.738499	9.822183	11.782887	5.180607	8.046636
GSM5656185_con	7.973438	7.359646	9.798115	8.402181	9.204653	9.27699	10.13743	9.085628	11.046948	5.802126	7.882408
GSM5656189_con	8.17656	7.562001	9.548409	9.349861	9.202668	9.40131	10.699004	10.337597	11.057792	5.321211	8.12695
GSM5656190_con	8.998814	8.262293	9.960509	9.341857	9.101128	9.322438	10.907465	10.836292	12.243964	5.174984	8.110928
GSM5656202_con	9.107295	7.488307	9.836428	8.514494	9.03215	9.663275	10.16398	9.580974	11.666934	4.899383	8.188276
GSM5656208_con	9.312689	8.155489	9.992057	9.286637	9.364418	9.189907	10.470778	10.631469	11.666934	5.149284	8.131952
GSM5656230_con	8.381459	7.893298	9.420998	9.141092	9.137253	9.258814	10.93873	10.49957	11.148069	4.970838	8.045255
GSM5656232_con	8.820432	7.261195	9.564796	8.68237	9.133869	9.570165	9.95122	9.836428	11.82225	4.930606	8.310306
GSM5656238_con	8.418829	7.673222	9.810531	8.611861	9.274418	9.743702	9.71486	10.202473	11.666934	4.857852	8.23864
GSM5656245_con	8.75883	7.546914	9.566511	8.635174	9.111587	9.591845	9.600884	9.898468	11.664024	5.234033	8.381073
GSM5656258_con	8.223828	7.281679	9.591845	8.12487	8.778636	9.25983	10.079404	9.198238	11.093054	5.338509	8.056133
GSM5656270_con	8.569903	7.784548	9.484177	9.178869	9.424215	9.133869	10.621259	10.689705	11.192781	4.921849	8.449995
GSM5656271_con	8.437923	7.543843	9.61232	8.519859	9.279057	9.623923	9.81121	10.152344	11.316444	5.147892	8.206992
GSM5656279_con	9.19192	7.31345	9.735817	8.482011	9.007866	9.668213	9.879639	9.61232	11.898687	5.129932	8.55936
GSM5656283_con	9.065532	7.340624	9.510403	8.168483	9.044112	9.339174	10.142775	10.074607	11.757489	5.226156	8.383364
GSM5656287_con	9.050645	7.467869	9.676213	8.757975	9.402936	9.971426	9.86678	10.113097	11.792794	4.370603	8.360274
GSM5656288_con	8.572255	7.71646	9.982037	8.62246	9.386039	9.948235	10.110577	10.139176	11.726185	4.720019	8.654117
GSM5656292_con	8.300532	7.492996	9.614103	8.401764	9.141092	9.618463	10.096076	10.00457	11.300917	5.421109	8.52214
GSM5656299_con	8.654117	7.774946	9.667618	8.63634	9.133869	9.718165	10.025462	9.899188	11.711152	5.377153	8.694987
GSM5656300_con	8.688023	7.618195	9.519029	9.308513	9.384496	9.610497	10.569012	11.06682	11.880704	5.213039	8.337496
GSM5656304_con	8.589903	7.471208	9.802249	8.349293	9.009234	9.56194	10.155995	9.516179	11.56482	4.851901	8.463362
GSM5656310_con	8.259514	7.543843	9.583499	8.467074	9.14495	9.663275	10.054751	10.098665	11.62292	5.201407	8.381459
GSM5656313_con	7.93118	7.4213	9.782673	8.39662	8.962339	9.233072	10.042485	8.585605	11.148069	5.754081	7.706839
GSM5656314_con	8.558983	7.885802	9.59731	9.185441	9.188384	9.3986	10.738281	10.630173	11.65219	5.221063	8.299829
GSM5656539_con	8.052294	7.915743	9.934713	8.897173	9.409576	9.665084	10.4381	10.109781	11.717096	5.289732	8.525524
GSM5656171_treat	8.544012	7.957355	10.109781	9.061386	9.391474	10.0449	10.30402	10.138298	11.643447	4.866809	8.38776
GSM5656174_treat	7.677646	7.950205	9.82763	9.095475	9.160869	9.608104	10.593831	9.235548	10.963783	5.386779	7.967638
GSM5656175_treat	8.958331	6.941657	10.402164	9.27699	8.997885	9.909465	10.005376	9.52354	11.52701	5.435742	8.990224
GSM5656177_treat	8.536313	8.535153	10.109781	9.101626	8.117866	9.881728	10.810593	10.334647	11.170523	5.039543	7.163486
GSM5656179_treat	8.703883	7.726624	10.073787	9.069762	9.192872	9.71045	10.197884	9.757626	11.370332	4.878085	8.789028
GSM5656183_treat	7.466526	7.626919	10.129502	8.969099	8.972696	10.091895	10.602545	9.670031	11.303003	5.484282	7.589368
GSM5656184_treat	8.958331	7.979678	9.90793	8.793269	8.924222	9.618463	10.170264	10.256516	12.100667	5.067794	8.668943
GSM5656186_treat	8.318806	7.744985	10.027096	9.010091	9.289221	9.763737	10.61497	10.116532	11.330425	4.802578	8.246804
GSM5656187_treat	8.606297	8.459976	10.155025	8.945504	8.584036	10.00229	10.711559	9.790003	11.493281	4.836386	7.849459
GSM5656192_treat	7.804206	8.509861	9.866056	8.213804	8.471317	8.926399	10.301157	9.891722	11.693311	5.019052	6.949304
GSM5656193_treat	7.987204	7.262957	10.840867	9.168725	9.546624	9.934713	10.257504	9.690156	11.10085	5.052675	8.902824
GSM5656195_treat	8.013694	7.906253	9.664461	8.586375	9.227037	9.543106	10.131143	9.63846	11.335162	4.973294	8.337496
GSM5656199_treat	8.048753	8.437923	9.768392	8.854027	8.938364	9.594314	10.467376	9.67996	11.271896	4.846941	7.77118
GSM5656200_treat	7.807981	7.139445	9.752345	8.974914	8.952954	9.184403	10.120841	9.580974	10.821163	5.283095	8.037606
GSM5656203_treat	7.916777	8.033379	10.11485	9.33704	9.1599	9.823512	10.473136	9.660788	11.446481	5.253434	7.798432
GSM5656207_treat	7.564686	7.870924	9.773141	8.921251	9.085178	9.431279	10.136531	9.282064	11.007166	5.15941	8.073604

GSM5656209_treat	8.989762	7.571457	10.714212	8.517499	9.317324	9.795413	10.117439	9.200706	11.654894	4.763051	8.828418
GSM5656212_treat	8.410292	8.179405	10.123428	8.987963	9.731782	9.734423	9.838553	9.230023	11.28748	4.90478	8.273787
GSM5656214_treat	7.748749	8.053355	9.652744	9.196281	9.489777	9.775784	10.509957	9.539572	11.28973	4.544793	8.37771
GSM5656217_treat	9.527043	7.340285	9.738499	8.571087	8.728186	9.203204	10.044104	9.909465	11.930702	4.888692	8.330556
GSM5656220_treat	8.369942	7.782548	11.572902	9.225027	9.517868	10.063823	10.54565	9.848956	10.993128	4.7971	9.43076
GSM5656223_treat	8.198142	8.102934	10.439195	8.992885	9.449623	9.513294	9.88952	9.187923	11.102815	4.883458	8.371444
GSM5656226_treat	8.370321	8.074286	9.837184	8.697823	9.000637	9.558299	10.312397	9.499846	11.508563	4.95096	8.188973
GSM5656228_treat	9.217115	7.913032	9.747082	8.448875	8.571486	9.234565	10.125075	9.942151	11.923466	5.203846	7.658821
GSM5656231_treat	8.407345	8.25445	10.552953	9.558871	9.479725	9.933207	10.496184	9.979812	11.074241	4.999362	7.749788
GSM5656235_treat	7.959113	7.580573	9.953541	9.142997	9.428565	9.772497	10.10461	9.522972	11.472	4.614516	8.247529
GSM5656237_treat	8.180788	8.315444	10.329562	9.301395	9.139655	9.826929	10.444658	9.316829	11.52701	4.88486	7.792027
GSM5656240_treat	8.500239	7.44302	9.49529	8.725779	9.14495	9.15023	10.390464	10.309798	11.720354	5.235038	7.792719
GSM5656243_treat	8.194609	7.493335	9.931676	8.600448	9.346094	9.634872	10.015003	9.430203	11.353915	4.718527	8.315063
GSM5656246_treat	8.495772	7.160708	9.485311	9.239612	9.249098	9.490316	10.496184	10.288314	11.708191	5.106477	8.043869
GSM5656249_treat	8.337142	7.817459	9.958993	9.03215	9.152173	9.885983	10.493802	10.197884	11.172537	4.798141	8.287128
GSM5656254_treat	8.095637	7.031317	9.559453	8.835628	9.101128	9.801539	9.919168	10.280355	11.690352	5.099306	8.174421
GSM5656255_treat	8.849337	7.609767	10.965403	8.879049	8.721267	9.116752	10.541926	9.557131	11.446481	4.923613	8.848052
GSM5656259_treat	8.467851	7.686965	9.920645	8.775377	9.780628	9.82763	10.187719	9.568943	11.597616	4.669679	8.845483
GSM5656262_treat	8.278733	7.119844	9.634273	9.155599	8.9437	9.905629	10.579097	9.95586	11.365644	4.774612	8.229424
GSM5656264_treat	8.752161	7.613175	9.265283	8.724167	8.658097	8.785334	10.119953	9.459064	11.479787	5.505148	7.506172
GSM5656265_treat	9.162818	7.749788	9.749112	8.942818	8.741057	9.445124	10.384159	10.130245	12.290403	4.944629	7.999276
GSM5656267_treat	7.948479	7.66618	9.948235	9.106328	9.532704	9.82763	10.187719	9.208453	11.392059	4.743807	8.093155
GSM5656273_treat	9.812546	7.114041	10.109781	9.99129	9.567107	9.890241	9.962039	10.387269	12.10474	5.145175	8.860061
GSM5656274_treat	8.563686	7.715437	10.158634	8.795289	9.060454	10.128593	10.092791	9.99129	11.401811	5.02777	7.882408
GSM5656277_treat	8.649394	7.810055	10.364464	9.372999	9.104434	10.03595	10.596244	9.975144	11.54836	4.725917	8.192508
GSM5656280_treat	9.053926	7.784876	10.417277	9.32964	8.809671	10.108065	10.797698	9.92577	11.426348	4.872804	8.148
GSM5656282_treat	7.833967	7.418544	9.848226	9.124779	8.804635	9.536149	10.464988	9.552534	11.380084	5.236773	8.039028
GSM5656285_treat	7.624859	7.594084	9.989744	9.005133	9.230523	9.918428	10.272732	9.216121	11.269591	5.04506	7.857682
GSM5656291_treat	8.727809	7.633442	9.605701	8.534756	8.788254	9.062288	10.24267	9.788033	11.687218	5.324248	7.752573
GSM5656294_treat	9.205624	7.508946	10.015003	8.794882	8.82464	9.069301	10.001525	9.850374	11.643447	5.080445	7.805564
GSM5656297_treat	8.847648	7.481701	9.544261	8.407345	8.6868	9.126192	10.100337	9.971426	11.91639	5.088977	8.025391
GSM5656303_treat	7.954531	8.004378	10.067114	9.240616	9.245609	9.741117	10.708868	9.640322	11.222008	4.711492	7.97009
GSM5656305_treat	7.711976	7.806248	9.919168	8.730614	9.199675	9.525906	10.276544	9.034041	10.822663	5.018372	8.141368
GSM5656309_treat	8.68276	7.336831	9.498706	9.255287	9.27496	9.63725	10.547934	10.183966	11.56738	5.047537	8.809273
GSM5656311_treat	7.585706	7.111317	9.603844	9.308513	8.972696	9.448492	10.337597	9.287694	11.05063	5.484622	8.030323
GSM5656316_treat	8.433071	7.73264	10.424855	8.91862	9.27496	10.163066	10.591388	10.010974	11.646349	4.453727	8.471317
GSM5656318_treat	8.78282	7.542489	10.147086	8.714408	9.329114	10.207977	10.38622	10.239681	11.786079	4.929893	8.730219
GSM5656321_treat	8.324285	7.668522	10.824086	8.880345	9.354189	9.812546	10.501868	9.742392	11.337603	4.54784	9.128625
GSM5656323_treat	8.806731	7.591649	10.533659	8.784928	9.674331	9.823512	10.384159	9.610497	11.611727	4.77321	9.317324
GSM5656325_treat	8.945504	7.920847	10.129048	9.074902	9.362836	9.823512	10.194161	9.798115	11.73237	4.745728	8.914657
GSM5656327_treat	9.099701	7.609058	10.086795	9.121917	9.439552	9.807098	10.394671	9.902836	11.887791	4.946739	9.282064
GSM5656329_treat	8.637891	7.371708	10.125075	8.646981	9.371379	9.853171	10.328517	10.383151	11.54031	4.737982	8.352155
GSM5656334_treat	8.390703	7.379358	10.246394	8.835628	9.389337	9.918428	10.183966	9.801539	11.82225	4.971183	9.07156

GSM5656335_treat	9.138677	7.944085	10.461604	8.860983	9.413851	10.073787	10.221747	10.149751	11.5757	4.960396	8.825455
GSM5656338_treat	8.007533	7.695239	9.965824	8.941927	9.470252	9.68319	10.177437	9.64461	11.62292	5.051318	8.079868
GSM5656342_treat	9.14401	7.738792	10.918644	9.117201	9.432476	10.179235	9.989744	9.84539	11.934315	5.08456	9.11346
GSM5656346_treat	8.70146	7.843471	9.986651	8.906723	9.323442	9.577388	10.881487	9.851812	11.283089	4.8093	8.697823
GSM5656348_treat	8.535153	7.874638	10.012545	9.149771	9.390398	9.977483	10.483518	10.205212	11.643447	4.91105	8.235736
GSM5656351_treat	8.242133	8.273103	10.269803	9.639084	9.101626	9.933976	10.42937	9.755641	11.236751	5.310291	7.851518
GSM5656353_treat	8.654919	8.058188	10.10461	8.958331	9.372999	9.95049	10.373807	9.759648	11.782887	4.692571	8.508716
GSM5656355_treat	8.754688	7.12364	10.633964	8.678722	9.448492	10.103012	10.305907	9.563044	11.711152	4.7856	9.289716
GSM5656359_treat	8.32798	7.668851	10.656702	9.115374	8.94595	9.649708	10.526488	9.877499	11.198815	5.14754	9.367138
GSM5656361_treat	8.475581	7.486049	11.356345	9.041843	9.323981	9.933207	10.246394	9.473618	11.708191	4.515923	8.827119
GSM5656365_treat	8.498387	7.802803	10.358257	9.118128	9.280574	9.935398	10.374843	9.581576	11.543137	5.170537	8.130539
GSM5656366_treat	9.095018	7.700328	10.859629	8.944144	9.434129	9.993709	10.205212	9.200199	11.766845	4.957302	9.310623
GSM5656368_treat	8.890642	7.131184	10.991338	9.299343	9.615352	10.223599	10.497408	9.790721	11.729238	4.953086	8.617444
GSM5656372_treat	9.234078	8.24283	10.593831	9.454577	9.32964	10.325654	10.689705	10.00699	11.570059	5.310953	9.127177
GSM5656374_treat	8.984794	8.098695	10.119953	9.100664	8.842921	9.85246	10.3021	10.042485	11.611727	4.807322	8.381459
GSM5656375_treat	8.447368	8.14622	9.634872	9.49927	8.779094	9.453428	10.736718	9.580974	10.500728	4.663376	9.556546
GSM5656380_treat	9.079095	7.765786	11.198815	8.911636	8.978938	9.933207	10.195108	9.588263	11.912789	4.754734	8.516033
GSM5656381_treat	8.622855	7.874981	10.168531	8.895873	9.208955	9.692684	10.085169	10.126895	11.941645	5.146868	8.083989
GSM5656384_treat	8.68473	8.361362	10.626268	9.283092	9.295817	9.538442	9.95122	9.532108	11.669565	4.824966	8.293689
GSM5656387_treat	8.097664	7.757957	9.862475	8.861824	9.101128	9.165271	9.533254	9.095946	11.274075	4.727389	8.077404
GSM5656388_treat	7.825258	6.880177	9.486444	8.997885	9.006923	9.576235	10.164925	9.502039	11.384712	5.689532	8.129497
GSM5656390_treat	7.795388	8.27799	10.038451	9.395265	8.131952	9.70091	10.989618	9.352588	10.747926	5.767608	7.038236
GSM5656392_treat	9.233591	8.050552	10.497408	8.872585	9.184912	9.705307	9.90793	9.898468	11.832042	4.683358	8.844665
GSM5656393_treat	8.425562	8.170948	11.763732	9.254252	8.587124	9.61232	10.424855	9.239148	10.84394	5.828767	7.63277
GSM5656395_treat	8.548559	6.71978	9.628236	8.911187	9.268328	9.483045	10.18015	10.107216	11.556403	5.037433	8.814242
GSM5656399_treat	8.316169	7.754998	9.822183	9.049267	9.014222	9.330218	10.05548	9.687672	10.816578	5.21411	9.547839
GSM5656400_treat	8.877726	8.607524	10.602545	9.238678	9.023012	9.798115	10.288314	10.307895	11.485292	5.016367	8.128438
GSM5656403_treat	8.845068	8.111977	10.264164	9.235548	8.707418	9.818114	10.597401	9.781998	11.959821	5.217776	8.126255
GSM5656406_treat	9.6565	7.658134	11.124017	9.550147	8.786168	9.942917	10.261355	10.21992	11.643447	5.064679	8.314349
GSM5656407_treat	8.906273	8.061291	10.334647	9.027589	8.578119	9.924325	10.775367	10.259363	12.028056	4.663751	8.329457
GSM5656409_treat	8.535922	6.562623	10.130245	9.241116	9.11394	9.856009	10.029454	9.562474	11.572902	4.819265	8.355488
GSM5656412_treat	8.574639	7.85421	11.627299	8.885505	8.867066	9.678112	10.012545	9.224504	11.278483	4.77067	8.273103
GSM5656416_treat	8.632348	8.006112	9.894661	9.050171	9.17553	9.579162	10.153267	9.318799	11.25216	4.880968	8.149411
GSM5656418_treat	8.441411	7.978318	10.086795	9.109211	8.950757	10.03595	10.374843	10.209802	11.603468	4.802578	7.734674
GSM5656421_treat	8.284219	7.363305	9.751043	9.164254	9.017002	9.441722	10.227358	9.8475	10.439195	5.510633	9.750434
GSM5656424_treat	8.381073	8.060254	10.121762	9.139194	8.769546	9.648427	10.086795	9.587638	11.592233	4.802948	8.012327
GSM5656426_treat	8.12027	7.559334	10.133857	9.31478	7.947114	10.160385	10.421688	10.853619	11.124017	5.689532	8.227659
GSM5656429_treat	8.342691	8.008216	10.0613	9.279057	9.083799	9.899949	10.61497	9.633077	11.344293	5.164747	7.902466
GSM5656431_treat	6.520236	7.513017	10.80057	9.476908	8.576224	9.009234	9.945961	9.072034	11.583872	4.565102	9.489777
GSM5656432_treat	8.184017	7.653154	10.253766	8.56597	9.176979	9.771813	10.068853	9.751696	11.448901	4.91351	8.097326
GSM5656434_treat	8.712421	8.514857	10.307895	9.244615	9.07156	10.122599	9.91695	9.833623	11.56738	4.583778	8.532835
GSM5656435_treat	8.385296	8.172692	10.165812	9.285109	9.108257	9.82419	10.239681	9.439552	11.519007	4.635169	8.185439
GSM5656439_treat	8.3885	8.30309	10.106412	9.035893	8.928576	9.796078	10.255529	9.95122	11.553652	4.792503	7.810386

GSM5656443_treat	9.137253	8.181164	10.918644	9.003359	8.628005	9.59972	10.61497	10.025462	11.498276	4.593585	8.902419
GSM5656444_treat	9.138677	7.964229	10.466146	9.283092	8.462253	10.072899	10.570222	10.689705	11.82225	5.028482	7.994445
GSM5656447_treat	7.913353	7.402649	9.489235	9.225027	8.805067	9.674331	10.557702	10.049884	11.303003	5.354044	7.892235
GSM5656451_treat	8.246804	8.133016	10.416161	9.58416	9.367663	10.119953	10.912057	10.124222	11.464173	4.604253	8.050552
GSM5656452_treat	8.084346	8.114379	10.612491	9.224504	9.477513	10.103012	10.513459	9.740453	11.174511	5.005173	7.533784
GSM5656454_treat	9.174497	8.556716	10.544394	10.113097	8.684328	10.434805	10.793233	10.758133	11.971726	4.836386	8.025066
GSM5656458_treat	7.982027	7.87193	9.82621	8.64462	8.868785	9.150712	10.289289	9.316829	10.962069	5.184698	7.849805
GSM5656460_treat	8.389607	7.674566	9.907109	9.010091	9.328064	9.780628	10.142775	9.952044	11.877169	4.953086	8.512586
GSM5656461_treat	8.428981	8.569528	10.114003	9.177919	8.861824	9.97284	10.766564	10.360305	11.426348	4.969798	7.628269
GSM5656464_treat	7.923901	8.450373	10.41282	9.596098	9.475239	9.781998	10.213498	8.803808	10.782909	4.817415	7.422619
GSM5656465_treat	8.562105	7.673222	9.691395	9.126192	9.219048	9.484746	10.16398	9.992927	11.451588	5.280048	7.79168
GSM5656467_treat	8.405832	7.967989	10.15685	9.107295	9.308513	9.947486	10.639254	9.99447	10.962069	4.879509	9.580974
GSM5656469_treat	8.146911	7.818486	9.952823	9.302381	9.015145	9.642758	10.13743	9.564796	11.005453	4.721111	7.836315
GSM5656473_treat	8.966842	7.59841	9.882457	8.760889	9.422034	10.366439	10.569012	10.470778	12.368535	4.856395	8.742265
GSM5656478_treat	8.241787	6.988032	9.816096	9.048381	8.994661	9.735094	10.406306	9.522972	11.135958	5.019367	8.050885
GSM5656481_treat	8.724963	8.275894	10.194161	9.265283	8.895459	10.135633	10.261355	9.896928	11.589427	4.728838	7.914046
GSM5656483_treat	8.088579	8.376632	10.601288	9.304447	9.152659	10.237802	10.776825	9.945961	11.409385	4.9453	7.450139
GSM5656486_treat	8.653346	7.741966	10.219457	9.122371	9.400215	9.960509	10.364464	9.859627	11.937898	4.757629	8.06165
GSM5656487_treat	7.961147	8.376977	9.932072	10.703312	9.121917	9.70091	10.556426	9.095475	10.972139	5.421793	7.318115
GSM5656490_treat	9.190888	7.265406	10.491484	9.395265	8.718854	10.283343	10.651277	10.163066	11.693311	5.32725	8.106066
GSM5656493_treat	7.943726	7.54794	9.95586	8.825069	9.390934	9.643383	9.941381	9.410697	11.628654	5.076326	8.30891
GSM5656495_treat	8.382205	7.795061	9.54076	8.769952	9.375118	10.081854	10.187719	9.990489	11.738205	4.996954	8.675469
GSM5656498_treat	8.47095	7.874638	10.173009	8.681195	8.96321	9.702155	10.165812	9.3986	11.870154	4.832784	8.192508
GSM5656501_treat	8.834381	8.142059	10.227358	9.30039	9.419391	10.040062	10.514599	10.277444	12.01166	4.842164	8.110242
GSM5656504_treat	8.983428	8.048389	10.320706	9.379001	9.238146	10.488042	10.755174	10.428312	11.766845	4.897968	8.381835
GSM5656505_treat	7.956315	6.693045	10.782909	8.896282	9.181363	9.734423	10.30402	9.539572	11.180437	4.916284	8.587903
GSM5656509_treat	8.610677	7.988598	10.210726	9.254782	9.251653	10.222663	10.403258	9.820813	11.720354	4.912813	8.414715
GSM5656511_treat	8.650961	8.507942	10.243587	9.433021	8.665001	10.263237	10.481092	10.42937	11.90587	4.856748	8.086126
GSM5656513_treat	8.148705	7.229947	9.678765	9.184912	8.912483	9.604469	10.207073	10.116532	11.786079	5.260255	8.319166
GSM5656516_treat	9.153593	7.756353	10.422725	9.295817	9.05578	10.298254	10.464988	10.380122	11.909348	4.775378	8.524428
GSM5656517_treat	7.665502	8.085074	9.718773	9.53729	9.108257	9.921343	10.411836	9.738499	11.172537	4.975059	7.73264
GSM5656520_treat	8.800843	6.837682	10.109781	8.691274	9.428565	9.246579	10.008562	9.022587	11.606199	4.825313	8.626383
GSM5656521_treat	7.960123	7.807981	11.211381	8.903712	8.962339	9.521872	9.973622	9.137253	11.170523	5.012435	7.85935
GSM5656522_treat	9.220031	7.76136	11.034182	8.996458	8.782416	10.022291	10.506468	9.724032	11.708191	4.834225	8.709495
GSM5656524_treat	8.074647	7.64292	9.996089	8.901989	8.616679	9.820813	10.393582	9.639084	11.316444	4.93592	7.844458
GSM5656525_treat	9.091709	8.034069	10.464988	9.141092	9.555423	9.846784	9.719403	9.896186	11.664024	5.124108	8.517499
GSM5656527_treat	8.762555	7.923565	9.955108	9.021659	9.076326	10.155995	10.591388	10.526488	11.880704	5.003841	8.664573
GSM5656528_treat	8.113701	7.620852	10.350936	9.789315	9.145931	9.910207	10.208923	9.580389	11.054189	4.728463	8.011624
GSM5656531_treat	9.237641	7.961869	10.351967	9.439003	8.927689	9.988147	10.427188	10.136531	11.95624	4.771777	8.343791
GSM5656533_treat	8.259514	7.521074	9.939133	8.429763	9.072034	9.943667	10.570222	9.764405	11.597616	5.124108	7.5738
GSM5656535_treat	8.503704	7.184406	9.794692	9.052984	9.347708	9.875275	9.969007	9.931676	11.485292	5.082483	9.004241
GSM5656536_treat	8.257364	7.584009	10.009333	9.020687	9.081922	9.906359	10.352968	9.658942	11.776455	4.926764	8.562908
GSM5656541_treat	7.969746	7.779132	9.825555	8.886328	9.262773	9.456217	10.16398	9.121917	11.351571	4.803299	7.940394

GSM5656543_treat	8.609101	8.02615	9.63846	9.367663	8.898495	9.921343	9.868984	10.372761	12.17764	5.138761	8.970009
GSM5656545_treat	8.25877	7.737077	9.954353	8.752991	9.465818	10.016611	10.481092	9.590032	11.349203	4.801844	8.350713
GSM5656549_treat	9.114445	7.604415	11.114146	9.658942	9.549593	10.148875	9.611092	9.790003	12.280064	5.117224	9.099701
GSM5656551_treat	9.076326	8.043525	10.673695	8.998324	9.663275	9.767768	9.838553	9.464683	11.426348	4.986588	8.897605
GSM5656554_treat	8.338982	7.544169	9.888851	8.969099	9.111587	10.016611	10.435837	9.876792	11.720354	4.95382	8.352855
GSM5656556_treat	8.740657	7.90695	10.276544	9.150712	8.994661	10.464988	10.636552	10.501868	11.835381	4.853679	7.654444
GSM5656559_treat	8.677113	7.473617	10.019043	8.804249	9.515576	10.045721	9.999905	9.952044	12.015797	4.964246	8.679536
GSM5656563_treat	7.86786	7.621165	9.789315	8.762954	9.270423	9.520184	10.307895	8.91862	11.10663	5.42726	8.301264
GSM5656565_treat	8.356213	7.778805	9.945245	8.783227	9.290225	9.936188	10.337597	9.522972	11.545921	4.814183	8.542434
GSM5656567_treat	9.112067	7.653782	10.138298	8.964995	9.52354	10.018234	10.031041	9.930209	12.249482	5.027069	8.766662
GSM5656569_treat	8.717237	7.767155	10.217084	8.880758	9.43076	9.794026	9.965091	10.066293	11.779333	4.412791	8.629936
GSM5656571_treat	8.036154	7.033406	9.917677	8.839105	9.072034	9.59731	10.207073	9.392	11.0686	4.844399	8.156887
GSM5656573_treat	8.660071	7.527105	11.178435	8.642235	9.196281	10.034289	10.358257	8.933005	11.699447	4.736123	8.720853
GSM5656576_treat	9.11346	7.349639	10.381147	8.675469	9.59731	9.90065	9.746373	9.727843	12.057057	5.055756	8.646981
GSM5656578_treat	8.067202	7.667487	9.690156	9.112067	9.079095	9.65153	10.167641	9.612904	11.556403	5.244991	8.216622
GSM5656581_treat	8.340481	7.75972	9.78541	8.901559	9.022587	9.772497	10.29725	10.273645	11.849335	5.083513	8.28606
GSM5656583_treat	8.287128	8.239305	10.374843	8.635542	9.191404	9.882457	9.8475	9.574389	11.990963	4.701849	8.952505
GSM5656585_treat	8.519074	6.651423	9.579746	8.583271	8.99975	9.942151	9.831592	10.402164	11.699447	5.045971	8.7924
GSM5656588_treat	7.963874	7.64292	9.465818	8.752574	9.022102	9.737835	10.527563	9.429109	11.56206	4.90478	8.382205
GSM5656589_treat	8.767077	7.492672	10.222663	8.741862	9.190888	10.290254	10.23593	10.203395	12.154019	4.732504	8.519461
GSM5656591_treat	9.164254	7.727576	10.165812	8.509492	9.195802	9.967368	9.985152	10.184926	11.998867	4.895473	9.093659
GSM5656595_treat	8.834813	7.742976	10.763803	9.011933	9.298858	9.87259	10.093601	9.269877	11.472	5.221063	8.290753
GSM5656596_treat	8.163264	7.838337	10.024642	8.984339	9.127177	10.042485	10.449121	9.86391	11.763732	4.678914	8.807607
GSM5656600_treat	9.628785	8.248997	10.813648	9.468633	9.003797	10.316816	10.608863	9.775784	11.73237	5.049553	8.690067
GSM5656601_treat	8.438703	7.722272	9.765097	8.63634	9.066051	9.930209	10.264164	9.948235	11.660937	5.088266	8.395162
GSM5656602_treat	8.404737	7.524776	10.546816	8.990224	9.247076	10.210726	10.825685	9.387703	11.278483	4.711492	8.628005
GSM5656603_treat	7.760041	7.336489	9.630013	8.858391	8.880758	9.800867	10.540817	9.665725	11.198815	5.190471	8.192878
GSM5656604_treat	8.52214	8.548927	9.648427	9.080058	9.447386	9.77924	10.324687	9.649708	11.529426	4.691099	9.749744
GSM5656606_treat	8.446218	7.531132	9.415485	8.384887	9.26034	9.70091	9.419943	9.905629	11.09121	5.083909	8.31212
GSM5656608_treat	8.607112	7.801485	9.792099	9.147393	9.408459	9.958993	10.151487	9.894661	11.664024	4.641229	8.745166
GSM5656611_treat	8.425562	7.540124	10.147997	9.045979	9.500413	10.076231	10.320706	9.425318	11.559153	4.684886	8.860983
GSM5656612_treat	8.266216	7.97454	10.270739	9.159419	9.350925	9.902836	10.325654	9.835042	11.285284	4.906533	7.593401
GSM5656615_treat	7.935258	7.679339	10.119953	8.970918	9.504751	9.669399	10.046598	9.61723	11.128129	4.922903	8.210578
GSM5656617_treat	8.41547	8.189351	10.220816	9.286133	9.128149	9.762361	10.097812	9.40184	11.485292	4.802578	8.107417
GSM5656619_treat	8.130539	7.84075	9.876015	8.662093	9.323442	9.496975	9.913146	9.497568	11.337603	4.838952	8.650584
GSM5656621_treat	7.974906	7.302945	10.187719	9.24013	9.138677	9.939876	10.165812	10.031861	11.545921	4.948146	8.168849
GSM5656624_treat	9.01884	8.004378	10.483518	9.12004	9.430203	10.124222	10.286379	9.822183	11.572902	4.672613	8.591471
GSM5656625_treat	8.155489	7.554535	10.312865	9.153151	9.636673	10.081	10.029454	9.659557	11.428862	4.707797	8.177642
GSM5656627_treat	8.45077	7.903206	9.983595	8.733869	9.32964	9.322929	9.854597	9.278493	11.120224	4.778273	8.133327
GSM5656631_treat	7.380036	7.895313	9.9497	9.354712	9.289716	9.553701	9.982827	9.116752	10.839361	4.713318	7.633442
GSM5656632_treat	7.79168	7.917458	9.730508	8.693342	9.125735	9.504751	10.111389	8.886328	10.845541	5.12722	8.015757
GSM5656635_treat	7.806917	7.813773	9.87259	9.234565	9.205127	9.598488	10.259363	8.794882	11.118185	5.036404	8.143481
GSM5656637_treat	8.773311	7.868911	10.250942	9.648427	9.228044	9.943667	10.406306	9.614103	11.396901	5.054388	8.017812

GSM5656639_treat	8.232302	7.879706	10.095244	8.907161	9.528715	9.88033	10.203395	9.487566	11.419087	4.916631	8.252988
GSM5656641_treat	8.671381	7.442358	10.872098	9.238678	9.513294	9.837184	10.175685	9.557713	11.842267	4.765914	8.976268
GSM5656645_treat	9.07156	7.469542	10.182985	9.207983	9.444	10.018234	10.081	9.738499	11.559153	4.810969	8.787864
GSM5656647_treat	7.770166	7.978994	10.086795	9.353118	9.377283	9.812546	10.511098	9.663275	11.209347	4.908926	8.150138
GSM5656648_treat	8.593022	7.846759	9.985872	8.967325	8.806304	9.873927	9.591845	10.127747	11.617242	4.848394	8.014037
GSM5656653_treat	7.69127	7.705476	9.591247	8.800019	9.302909	9.715529	10.501868	9.52647	11.124017	4.979565	8.293335
GSM5656655_treat	8.958331	7.647624	11.03962	9.364418	9.535561	10.038451	10.03595	9.543106	11.986949	4.762317	8.856184
GSM5656657_treat	8.209108	7.312781	10.132033	9.273463	9.082827	9.868984	10.134728	9.68123	11.754266	5.101354	8.088223
GSM5656173_treat	8.073261	7.889862	9.864646	9.131446	9.270423	9.580974	10.626268	9.38879	11.061421	4.91351	7.744335
GSM5656182_treat	8.106421	7.654444	9.905629	8.805888	9.11346	9.691395	10.303079	9.411246	11.247566	5.257883	7.962507
GSM5656191_treat	9.021194	7.624539	9.792099	7.900429	7.719151	9.410697	10.228275	10.183966	11.61441	5.295897	8.813815
GSM5656194_treat	8.027196	7.506859	9.695887	8.878191	9.050645	9.611726	10.279365	9.782673	11.021462	4.710352	8.30891
GSM5656196_treat	7.9287	7.850857	9.437959	8.483155	9.106328	9.626433	10.326612	9.742392	11.249925	5.125846	8.207345
GSM5656198_treat	8.376244	8.528256	10.051478	8.868785	8.889795	9.851812	10.517006	9.97671	11.451588	5.035402	7.647284
GSM5656205_treat	8.015757	7.883051	10.009333	9.58222	9.088323	10.031041	10.309798	10.150591	11.643447	5.316436	7.615212
GSM5656210_treat	8.56407	7.985818	10.816578	9.255786	8.95383	10.081854	10.494973	9.973622	11.466689	4.872448	7.750109
GSM5656211_treat	7.749788	8.006838	9.933976	9.261323	8.839105	9.844753	10.965403	9.449054	10.953528	5.082483	7.965546
GSM5656215_treat	7.589368	8.065166	9.83432	9.011461	9.286133	9.395265	10.501868	9.389877	11.160365	4.90365	8.043525
GSM5656218_treat	8.539024	7.645583	9.848956	8.222763	8.414319	9.323981	10.177437	9.72651	11.409385	5.470985	8.343033
GSM5656219_treat	7.967295	8.310306	10.041667	9.243114	9.069301	9.916174	10.5053	9.853889	10.671	5.029152	7.524427
GSM5656222_treat	7.223092	7.106184	9.557131	8.801648	8.943253	9.313736	10.369592	9.376701	10.726769	5.226156	8.156543
GSM5656227_treat	7.860045	7.390104	9.925008	9.134358	9.296856	9.849629	10.492597	9.977483	11.09121	5.126724	8.079164
GSM5656234_treat	8.084696	7.744985	9.828983	8.952505	9.182912	9.568943	10.207977	10.184926	11.344293	4.795305	8.111265
GSM5656236_treat	8.103271	8.752574	10.464988	9.743044	8.599263	10.128593	11.065022	9.388226	11.192781	4.828544	7.248007
GSM5656239_treat	9.155599	6.945414	9.990489	8.375473	7.99864	9.355247	10.449121	9.868984	11.479787	5.48226	8.600057
GSM5656241_treat	9.00153	7.638177	9.769792	8.116502	8.06863	9.500413	10.635242	10.300132	11.628654	5.65771	8.597304
GSM5656244_treat	7.419887	7.122612	9.550704	8.755898	9.030299	9.480258	10.169417	9.217607	10.84394	5.268222	7.922199
GSM5656247_treat	9.292246	7.597742	10.034289	8.518308	8.184017	9.241628	10.194161	10.380122	11.608908	5.154978	8.553999
GSM5656251_treat	8.064436	7.923247	9.667618	9.407888	8.780382	9.849629	11.349203	10.443456	11.351571	5.33136	8.053032
GSM5656253_treat	7.791509	7.476325	9.446815	8.68315	9.130527	9.641524	10.017404	9.85893	11.495881	4.944968	8.468629
GSM5656256_treat	8.854462	7.732979	9.780628	8.708658	8.032725	9.395813	10.527563	10.182985	11.570059	5.13775	8.490028
GSM5656263_treat	8.696619	7.661522	9.916174	8.701853	8.085425	9.46355	10.56648	10.132033	11.495881	5.514706	8.520967
GSM5656268_treat	7.001771	8.26942	10.077034	9.100203	9.376701	10.008562	10.527563	8.980723	10.897495	4.875662	7.651763
GSM5656272_treat	8.33567	8.388139	9.965824	8.934324	8.652949	9.720091	10.434805	10.0613	11.363323	5.013845	7.99167
GSM5656275_treat	8.143803	7.377714	9.854597	8.738158	8.897605	9.602705	10.2853	9.728502	11.316444	5.316088	8.043869
GSM5656278_treat	8.674279	7.672202	10.022291	9.009234	8.957449	9.744364	10.517006	10.040473	11.514109	4.943567	8.690858
GSM5656281_treat	7.882408	7.869934	9.67308	9.144447	8.878191	9.593075	10.439195	9.352588	11.260961	5.303123	7.710292
GSM5656286_treat	7.539782	7.68302	9.746373	8.92378	9.181859	9.594958	10.195108	9.671219	11.209347	5.103077	7.835957
GSM5656289_treat	8.105717	7.927668	9.276502	8.817049	9.40184	9.235061	10.571467	10.440291	11.433736	5.118278	7.857303
GSM5656293_treat	8.318448	7.858366	9.573131	9.093157	9.169648	9.082381	10.617627	10.234962	11.238882	5.019749	7.87767
GSM5656296_treat	9.066051	7.633442	9.985152	8.375095	8.01643	9.62586	10.309798	10.103868	11.508563	5.275978	8.663745
GSM5656301_treat	8.114742	7.942696	9.992057	8.952063	9.139655	9.674331	10.616253	9.419943	11.213455	5.049916	8.078808
GSM5656307_treat	7.367973	7.181302	9.732475	8.861824	9.016103	9.460737	10.317785	9.444591	10.778345	4.980265	8.255494

GSM5656308_treat	9.059991	7.649863	9.828337	8.313977	8.344515	9.101128	10.031041	9.905629	11.514109	5.157343	8.410292
GSM5656320_treat	8.486567	7.957703	10.224611	8.926818	9.392568	9.81741	10.587754	9.820134	11.406923	4.904027	8.675469
GSM5656324_treat	8.194962	7.697278	10.072035	9.235061	9.705307	9.910936	10.451364	9.755641	11.625943	4.759113	8.928576
GSM5656326_treat	8.406216	7.67731	9.978266	9.118614	9.381752	9.802957	10.276544	10.220816	11.506026	5.188777	8.334558
GSM5656328_treat	7.38141	7.656116	9.781319	9.069762	9.270423	9.871843	10.458181	9.382807	11.007166	4.999719	8.16533
GSM5656331_treat	8.68276	7.345298	10.485815	8.672233	9.533804	10.165812	10.254613	9.792715	11.76988	4.837868	8.992446
GSM5656332_treat	8.151139	7.854909	9.208453	8.822528	8.905422	9.338087	10.402164	9.735817	11.230394	4.939378	8.927689
GSM5656336_treat	8.129497	8.040473	9.694542	9.054837	9.305977	9.456217	10.3306	9.695192	11.406923	5.168495	8.063779
GSM5656340_treat	8.159755	7.860398	10.231261	9.749744	9.888851	9.720736	10.128593	10.059625	11.456579	4.943953	7.729915
GSM5656343_treat	8.1099	7.767155	9.870357	8.731431	9.258319	9.790003	10.248102	9.551943	11.594975	4.839327	8.40772
GSM5656345_treat	8.275155	7.94607	10.045721	9.253211	9.210985	9.814706	10.125075	10.091088	11.380084	5.104774	8.062387
GSM5656349_treat	8.103634	7.638854	9.786729	9.207983	9.439552	9.398089	10.265975	9.289716	11.052467	5.045424	7.99864
GSM5656350_treat	8.526337	8.005406	10.046598	9.251653	9.259307	9.856787	10.639254	9.758982	11.190666	5.376123	7.797762
GSM5656354_treat	7.85556	8.006838	9.759648	9.03215	9.26937	9.44009	10.211679	9.386861	11.24303	5.305201	7.961519
GSM5656357_treat	8.205978	7.848143	9.882457	8.908471	9.242606	9.711661	10.271728	9.970613	11.472	5.244991	8.337142
GSM5656362_treat	7.976586	7.730262	9.687672	8.594957	9.360649	9.658942	10.540817	9.550147	11.44393	5.236418	8.978059
GSM5656363_treat	8.41657	7.890542	9.835042	9.110171	9.257818	9.56194	10.188646	9.381184	11.416737	5.17761	8.051935
GSM5656367_treat	8.149411	7.870924	10.490337	8.843356	9.521322	9.833623	10.228275	9.379001	11.411895	4.860386	8.842504
GSM5656370_treat	8.465956	7.826922	9.548995	8.728985	8.957918	9.837184	10.756657	10.114003	11.51653	4.744547	8.237915
GSM5656371_treat	8.177642	7.593026	10.151487	9.225548	9.507525	10.506468	10.758133	10.358257	11.86634	4.918067	8.458077
GSM5656373_treat	8.019459	7.686623	9.605107	8.720853	8.752574	9.317324	10.396812	9.848956	11.215633	5.334428	8.168849
GSM5656377_treat	8.053683	7.693247	9.614738	9.466357	8.768696	9.596098	10.80914	9.345023	10.584117	4.893373	8.857972
GSM5656378_treat	8.844665	7.675931	9.993709	9.404039	8.640261	10.128593	10.721232	10.167641	11.411895	5.111464	8.235736
GSM5656379_treat	8.210578	8.828004	10.557702	9.094124	8.669753	9.684436	10.677625	9.916174	11.139733	5.151298	6.977107
GSM5656382_treat	8.106724	8.236812	9.588868	8.773732	8.701057	9.60028	10.561626	9.831592	11.18473	5.057096	7.673566
GSM5656386_treat	7.730629	8.511812	10.354024	9.661406	8.996458	9.516751	10.266919	8.769546	10.692417	4.988309	7.29895
GSM5656391_treat	8.388866	8.36772	10.326612	9.412835	8.788254	9.749112	10.387269	9.875275	11.346646	5.102729	7.517344
GSM5656397_treat	8.689664	7.057314	9.510982	8.656128	8.398095	9.497568	10.490337	10.18957	11.448901	5.307223	8.77616
GSM5656401_treat	8.232641	8.038647	9.781998	9.159419	8.796159	9.521872	10.018234	9.444591	11.204936	4.86859	7.916777
GSM5656402_treat	7.752573	7.692896	9.616618	8.755476	9.182912	9.68319	10.478749	9.727843	10.791743	5.114213	7.915083
GSM5656404_treat	9.045515	8.620145	10.4381	9.45177	8.406216	9.790721	10.818144	10.006103	11.578478	4.748243	7.397185
GSM5656408_treat	7.9983	7.997574	9.798115	8.907582	8.640261	9.599099	10.296257	9.538994	11.292	5.34046	7.996873
GSM5656411_treat	8.256248	8.298777	10.385216	9.078649	8.853173	9.606945	10.300132	9.563656	11.180437	5.154978	7.878336
GSM5656413_treat	8.083989	7.860744	10.099493	9.238678	8.366198	10.039311	10.887934	9.78541	11.196777	4.95589	7.479988
GSM5656415_treat	8.350376	8.227659	10.169417	9.420447	8.916844	9.733115	10.611276	9.497568	10.968712	5.09276	7.362318
GSM5656419_treat	8.25877	7.824571	9.855284	8.871376	9.000158	9.58705	10.082646	9.625196	11.426348	5.152958	8.121645
GSM5656422_treat	8.301264	7.454917	9.678765	8.945038	9.045979	9.534988	10.090221	9.916174	11.219664	4.936256	7.91914
GSM5656427_treat	7.98305	8.344515	9.555971	9.524097	8.246073	9.934713	10.707421	10.526488	11.245291	4.664866	7.826922
GSM5656433_treat	7.530129	7.436297	10.119056	8.849337	9.209463	9.582858	10.289289	8.986198	10.899049	5.506821	7.813773
GSM5656436_treat	8.821678	8.611861	10.374843	9.116752	8.597304	9.790721	10.620066	10.212558	11.594975	4.798141	7.664488
GSM5656438_treat	8.815857	8.081962	10.38832	9.44009	8.596543	9.986651	10.892709	10.089347	10.804868	5.059169	7.829634
GSM5656440_treat	8.171309	8.425924	10.193283	9.117201	8.982101	9.72651	10.462712	9.73379	11.344293	5.251783	7.587058
GSM5656442_treat	7.920847	8.352486	9.68123	8.911636	8.706628	9.798801	10.624942	9.557131	10.936999	5.204187	7.964585

GSM5656446_treat	8.509861	8.517499	9.376176	9.562474	8.309286	9.509857	10.677625	9.790003	11.176465	5.58225	7.5823
GSM5656449_treat	7.982382	7.764423	10.086795	9.547286	9.272446	9.944472	10.723953	9.763057	11.120224	5.302104	7.543507
GSM5656453_treat	7.929021	8.132296	9.956656	9.280574	9.223017	9.622664	10.585254	9.510982	11.168561	5.396642	7.401294
GSM5656456_treat	8.754688	7.80386	10.237802	9.971426	8.748834	10.119056	10.508729	9.913146	11.236751	5.058142	8.485445
GSM5656457_treat	8.024027	7.917797	9.668213	8.784928	8.895873	9.223557	10.477623	9.640322	11.063258	4.955164	7.457288
GSM5656459_treat	8.255147	7.498109	9.837843	8.94944	9.372999	9.693914	10.136531	9.899188	11.660937	4.838952	8.539024
GSM5656462_treat	8.201304	8.129849	9.99129	8.89421	9.034516	9.510403	10.562789	9.816721	11.209347	5.290083	7.73164
GSM5656466_treat	8.88938	7.746396	9.844753	9.056248	9.181859	9.36342	10.440291	10.293282	11.307668	5.197287	7.799123
GSM5656468_treat	7.765786	7.180984	9.590614	8.953403	9.104434	9.604469	10.372761	9.702748	11.026711	5.286262	8.268718
GSM5656472_treat	8.32798	6.517783	10.018234	9.058096	9.174011	9.823512	10.144546	9.639084	11.54031	4.887672	8.541697
GSM5656475_treat	9.432476	7.658134	10.332578	9.1677	9.412835	9.82419	10.264164	10.213498	11.990963	4.937994	9.142074
GSM5656476_treat	8.242133	7.211141	10.418387	8.960109	8.637077	9.678112	10.474264	8.931685	11.072233	4.863312	7.468222
GSM5656479_treat	7.953868	7.912365	10.015003	9.37084	8.855751	9.935398	10.473136	9.400769	11.254459	4.814183	7.319851
GSM5656484_treat	7.900096	8.019792	9.947486	8.861824	9.170128	9.469687	10.224611	9.204653	10.960393	4.972942	7.89566
GSM5656491_treat	8.709495	7.40505	10.397857	9.233591	8.813396	10.086795	10.376933	9.758314	11.581209	5.043678	7.700328
GSM5656494_treat	8.088928	8.54552	10.218993	9.259307	9.161321	9.562474	10.766564	9.688295	11.10837	5.225774	7.09669
GSM5656497_treat	8.293335	8.114016	9.969773	9.250085	8.629194	9.822183	10.424855	9.490316	11.720354	5.218089	7.766138
GSM5656499_treat	9.081471	7.958765	10.300132	9.273918	9.115836	9.985872	10.303079	10.211679	12.158794	4.968758	7.757601
GSM5656502_treat	8.385644	8.298056	10.027096	9.16676	9.068842	9.837184	10.514599	9.766459	11.487945	5.12962	7.582653
GSM5656507_treat	8.044586	8.16432	10.269803	9.265785	8.733869	10.155025	10.872098	9.818114	11.351571	4.862954	7.781532
GSM5656510_treat	8.625242	7.948799	10.387269	9.459639	9.120531	10.098665	10.561626	10.401068	11.625943	5.128638	7.74126
GSM5656512_treat	7.569698	8.060254	9.846078	8.984794	9.045052	9.747082	10.474264	9.067417	11.026711	4.872105	7.905223
GSM5656515_treat	8.858391	8.136783	10.322702	9.214004	9.071094	9.857538	10.281357	9.987401	11.983103	4.603107	8.077043
GSM5656519_treat	8.612254	8.065166	10.39146	8.94989	9.219573	9.563656	9.978266	9.534988	11.280832	4.743107	7.800782
GSM5656523_treat	8.208101	7.861386	9.913916	8.971831	8.579695	9.566511	10.265047	9.716143	11.409385	5.196908	8.304905
GSM5656526_treat	8.457318	7.431642	9.639084	9.247618	8.669346	9.444	10.19236	9.70344	11.323554	4.90478	8.301264
GSM5656529_treat	8.423763	8.258382	9.977483	9.941381	8.721267	9.966609	10.53124	9.494708	11.61441	4.878806	7.685662
GSM5656538_treat	8.235063	8.247903	10.111389	8.980723	8.78282	9.572527	10.229252	9.521872	11.446481	4.969455	7.889489
GSM5656540_treat	7.954203	7.662164	9.662614	9.127177	9.041381	9.721355	10.251868	9.404582	11.300917	5.091048	8.000633
GSM5656544_treat	8.330909	8.100126	9.9497	9.114904	9.118614	9.783371	10.460447	10.12598	11.446481	4.891172	8.07636
GSM5656547_treat	8.663334	7.66618	9.525906	8.981183	9.200706	9.61965	10.231261	9.773141	11.553652	4.791776	8.206629
GSM5656550_treat	8.137803	7.46205	9.173543	8.46943	8.462253	9.403506	10.041667	9.774414	11.773153	5.003125	8.604729
GSM5656552_treat	8.261246	7.95121	9.567715	8.731431	8.462628	9.604469	10.3675	9.876792	11.524426	5.177285	8.321019
GSM5656553_treat	8.607524	7.906604	9.895428	9.02892	8.700245	9.606945	10.349955	9.83432	11.456579	4.840719	7.947459
GSM5656557_treat	8.142059	7.727268	10.015837	9.221031	8.945504	9.746373	10.579097	9.608719	11.141861	5.267558	7.726624
GSM5656560_treat	8.699457	8.200232	9.934713	8.912039	8.729393	9.655285	10.563991	9.867489	11.380084	5.403508	8.117183
GSM5656561_treat	7.219235	7.80386	9.456217	8.883743	9.104885	9.428565	10.513459	9.652122	10.978983	5.363962	8.178039
GSM5656566_treat	8.499514	7.858692	10.042485	8.910268	9.356304	9.821517	10.310944	10.314723	11.819196	4.959686	8.679128
GSM5656568_treat	7.944733	7.969394	9.987401	8.676298	9.391474	9.609296	10.252837	9.719403	11.370332	4.839327	7.955615
GSM5656572_treat	8.672637	8.049128	9.885983	9.03866	8.800019	9.531014	10.502984	10.315792	11.592233	4.957302	7.539459
GSM5656574_treat	8.015411	7.647624	9.757626	8.766662	8.984794	9.500413	10.21437	10.049884	11.375262	5.263737	7.887455
GSM5656577_treat	8.675897	7.782199	9.767768	8.568315	8.377346	9.592478	10.427188	10.689705	11.487945	5.383293	8.059936
GSM5656580_treat	8.586375	8.260167	9.838553	8.963645	8.887629	9.709787	10.250942	10.098665	11.620199	5.120021	7.814771

GSM5656582_treat	8.409913	7.907966	9.921343	8.734743	9.259307	9.732795	9.818114	9.730508	11.975749	5.238178	8.69046
GSM5656586_treat	8.30891	6.790271	9.965824	8.681195	9.197272	9.792099	10.187719	9.789315	11.42394	4.993574	8.110242
GSM5656587_treat	8.105717	7.685316	9.455688	9.079586	8.978059	9.471933	10.475426	9.687672	11.603468	5.111126	8.79113
GSM5656592_treat	8.787864	6.437707	9.68319	8.932117	8.996914	9.648427	9.986651	9.943667	11.511313	5.204187	8.577373
GSM5656593_treat	7.635136	7.999974	10.335598	8.919531	9.271459	9.746373	10.119505	8.93656	11.353915	5.038823	8.097664
GSM5656598_treat	8.326164	8.132673	9.878223	8.924222	8.979379	9.758314	10.049074	9.536713	11.003719	5.236773	7.44302
GSM5656605_treat	7.857303	7.683346	9.822183	8.927255	9.202668	9.496975	10.422725	9.525906	11.15441	4.697442	8.17727
GSM5656607_treat	8.098349	7.781532	9.45177	9.096422	8.968659	9.566511	10.044104	9.860362	11.339906	4.991822	8.108146
GSM5656610_treat	7.899759	7.582999	9.788659	8.843775	9.226537	9.478601	10.282346	9.191404	11.008991	4.955532	8.342691
GSM5656613_treat	7.912036	7.733658	9.964318	9.107295	8.938813	9.367663	10.223599	8.641045	11.419087	4.896544	7.908312
GSM5656616_treat	7.647624	8.12027	9.807784	9.021194	9.096422	9.524722	10.351967	9.90793	11.192781	4.937679	8.275894
GSM5656618_treat	8.008216	7.741966	9.384496	8.798324	8.994661	9.33077	10.173911	9.485311	11.404375	5.144824	8.080215
GSM5656626_treat	8.015757	7.84075	9.83432	9.265283	8.926818	9.641524	10.234962	9.190888	10.9825	5.070876	8.078106
GSM5656628_treat	7.977643	8.194609	9.892426	8.665001	9.128149	9.334454	10.436913	8.857972	11.021462	5.034347	7.008035
GSM5656630_treat	8.287128	7.922902	9.988147	9.134858	9.033593	9.665725	10.090221	9.308012	11.070533	4.766277	7.258814
GSM5656633_treat	7.574142	7.883051	9.998478	9.020687	9.001976	9.40131	10.343656	8.98754	10.946884	5.107486	8.035164
GSM5656636_treat	7.703048	8.376977	9.807784	9.784717	9.153151	9.563044	10.3675	9.720091	11.139733	4.855717	7.927668
GSM5656638_treat	8.27345	7.42397	9.716792	9.278493	8.829692	9.63786	10.188646	9.2563	11.392059	5.017398	8.26409
GSM5656640_treat	7.758656	7.546214	9.31321	8.852292	8.972271	9.481369	10.368511	9.234078	11.008991	5.351023	8.094187
GSM5656642_treat	8.082977	7.469542	9.807098	8.912483	9.022587	9.822183	10.270739	9.543656	11.556403	4.883827	8.232302
GSM5656644_treat	8.692932	7.28575	9.916174	9.12004	9.292246	9.764405	10.017404	9.92577	11.877169	4.954482	8.521741
GSM5656646_treat	8.134035	8.084346	9.960509	8.922063	9.250608	9.601492	10.546816	9.767768	11.230394	4.901489	7.924916
GSM5656650_treat	8.988844	8.521741	10.521822	9.615962	8.978059	9.676856	9.915389	10.658019	11.61441	5.110478	7.902466
GSM5656651_treat	7.760731	7.70236	9.465818	8.496139	8.842921	9.658312	10.128593	9.512121	10.82717	4.97015	8.101907
GSM5656654_treat	7.447454	8.225227	10.088094	9.006037	9.393092	9.860362	10.489217	9.384496	11.0686	5.199067	7.644312
GSM5656658_treat	7.819805	7.753617	9.678765	8.969564	8.902824	9.542485	10.191482	9.36342	11.20721	5.317807	7.85556
GSM5656170_treat	8.267677	7.786929	9.898468	8.944609	9.066531	9.870357	10.284303	10.057983	11.451588	5.068118	7.911338
GSM5656172_treat	9.396943	7.885802	10.078611	8.466714	8.839956	9.524722	10.440291	10.224611	12.09194	5.465888	8.41547
GSM5656176_treat	8.102934	8.230478	10.079404	8.984339	9.125735	9.808428	10.338583	9.737195	11.404375	4.938349	7.861726
GSM5656178_treat	8.083294	7.366701	9.711661	8.838244	8.88122	9.381184	10.360305	9.47414	11.019607	5.39595	7.577148
GSM5656181_treat	8.645824	7.934901	10.225527	9.131446	8.049128	9.700236	10.656702	10.043282	11.061421	5.221063	7.815126
GSM5656188_treat	8.530926	8.101205	10.11825	9.243114	8.998324	10.232213	10.596244	10.243587	11.649343	5.067794	7.710292
GSM5656197_treat	8.382571	8.895873	10.016611	8.838675	8.215517	9.543656	10.596244	10.062939	11.43616	5.640949	7.245594
GSM5656201_treat	8.296947	6.868726	9.676213	8.734743	9.294832	9.406226	10.163066	10.031041	11.141861	5.260957	8.263728
GSM5656204_treat	8.262635	8.065812	10.110577	9.010578	8.869614	9.702748	10.433745	9.775096	11.485292	5.305886	8.198495
GSM5656206_treat	8.430483	8.239305	9.914658	8.796592	8.691274	9.554296	10.397857	9.942151	11.409385	5.267892	7.907966
GSM5656213_treat	8.17167	7.288148	9.968193	8.99975	9.425318	9.677452	10.136531	9.957466	11.358639	5.527804	8.605114
GSM5656216_treat	8.285666	6.505453	9.925008	9.235548	9.010578	9.888159	10.295246	10.0449	11.204936	5.224746	8.160451
GSM5656221_treat	8.191797	8.34747	10.205212	9.002455	7.947788	9.788659	10.689705	9.754328	11.06682	5.535261	7.424313
GSM5656224_treat	8.797021	7.415843	10.083476	8.649394	9.127177	9.809879	9.9497	10.010174	11.370332	5.053391	8.24037
GSM5656225_treat	8.030323	8.027888	9.993709	8.745166	8.825455	9.6021	10.11825	9.576235	11.421431	4.837144	7.833967
GSM5656229_treat	8.696201	6.87736	9.913146	8.797895	8.99975	9.299867	10.255529	10.090221	11.178435	5.183706	8.355488
GSM5656233_treat	9.332872	7.557586	10.15412	8.729393	8.895054	9.928737	10.178342	10.254613	12.207407	4.915607	8.817895

GSM5656242_treat	9.104885	7.291924	10.043282	8.574248	9.522972	9.797466	9.90065	10.940317	11.660937	5.716427	8.828877
GSM5656248_treat	9.71045	7.173228	9.790003	8.485803	8.348922	9.346094	9.913146	10.260329	11.849335	5.110796	8.194962
GSM5656250_treat	8.635174	8.288198	9.608719	9.073934	8.945504	9.606945	10.368511	9.525283	11.763732	5.270212	8.849337
GSM5656252_treat	8.486199	8.502926	10.250033	9.100664	7.874981	9.278013	10.649872	10.390464	11.139733	5.160057	7.583351
GSM5656257_treat	7.972776	6.974642	9.577388	9.212002	9.03215	9.728502	9.985872	9.554296	9.970613	5.348258	7.794732
GSM5656260_treat	8.597304	8.296571	10.224611	9.172084	8.477841	10.101176	10.801985	10.2853	11.472	4.906184	7.79643
GSM5656261_treat	8.957918	6.770407	9.698344	8.658097	9.006037	9.376701	10.248102	10.366439	11.56482	5.244991	8.355857
GSM5656266_treat	8.838244	7.15587	9.910936	8.972271	8.696201	9.081471	10.181093	9.731782	11.194903	5.357872	7.895313
GSM5656269_treat	8.856184	7.262243	9.654603	8.750502	9.458521	9.594314	10.366439	10.371642	11.459113	5.305532	8.387052
GSM5656276_treat	8.849337	7.870924	10.360305	9.157485	9.221516	10.08599	10.394671	10.23877	11.660937	4.78522	7.80939
GSM5656284_treat	8.007886	7.910993	9.881728	8.618554	9.30191	9.609922	10.196044	9.772497	11.466689	4.826414	8.125906
GSM5656290_treat	8.834381	6.600914	9.844081	8.671381	9.40131	9.888159	9.91695	10.502984	11.895226	5.018709	8.428981
GSM5656295_treat	8.649818	7.288849	9.781998	9.436317	9.433021	9.674973	10.196964	10.145373	11.606199	5.009277	8.292961
GSM5656298_treat	8.867066	6.652778	9.671219	8.865805	8.400668	9.648427	10.2853	10.195108	11.551018	5.324601	8.544012
GSM5656302_treat	8.541324	8.231917	10.404224	9.349861	8.654919	10.03595	10.591388	9.825555	11.553652	5.08967	7.594084
GSM5656306_treat	8.20946	6.819442	9.678112	8.980723	9.30751	9.775784	10.172078	9.766459	10.872098	5.120694	8.250122
GSM5656312_treat	8.804249	7.722272	9.90211	8.628801	8.125566	9.47139	10.363404	10.772393	11.56206	5.676817	7.982382
GSM5656315_treat	8.387381	7.810386	10.053148	8.747608	9.622048	9.754978	10.408582	9.965091	11.660937	5.066049	8.404038
GSM5656317_treat	8.458434	8.756314	9.380058	9.5196	8.602404	9.86391	11.017876	10.173009	10.925186	5.508907	7.479287
GSM5656319_treat	8.125906	7.732308	9.774414	8.859236	9.284603	9.987401	10.316816	9.798115	11.757489	5.169208	8.247903
GSM5656322_treat	8.205978	7.792027	10.090221	8.991598	9.53729	9.99447	10.321746	9.927296	11.640582	4.777214	8.270124
GSM5656330_treat	8.240019	7.929756	9.857538	8.646597	9.261323	9.73379	10.138298	9.754978	11.779333	5.16816	8.402181
GSM5656333_treat	7.638854	8.267323	9.908706	8.859643	8.990674	9.783371	10.313332	9.893129	11.375262	5.010701	7.448456
GSM5656337_treat	8.730219	8.467074	10.601288	9.707222	8.964995	10.194161	10.842356	10.491484	11.323554	5.01527	7.804206
GSM5656339_treat	8.259514	8.097664	9.853171	8.526711	9.023012	9.680589	10.128593	9.995275	11.553652	5.003501	8.114742
GSM5656341_treat	8.040473	7.738442	9.948235	8.693342	9.182383	9.601492	10.287341	9.572527	11.314075	4.75585	8.197437
GSM5656344_treat	7.896001	7.850174	9.575629	8.794089	9.090259	9.593075	10.529922	9.603287	10.973868	4.657748	7.455976
GSM5656347_treat	8.424823	8.601609	10.310944	9.280087	8.975335	9.965091	10.900752	10.090221	11.738205	5.020087	7.484062
GSM5656352_treat	8.34528	7.60741	9.640322	8.530926	9.252672	9.800867	10.518216	9.475794	11.500656	5.075275	7.807625
GSM5656356_treat	8.115422	7.47729	10.129502	9.067417	9.488658	9.982037	10.317785	9.551943	11.862939	4.616729	8.005043
GSM5656358_treat	8.279466	7.529798	9.441722	9.023939	9.110171	9.723376	10.339566	9.632447	11.349203	5.196249	8.587903
GSM5656360_treat	8.226633	8.151859	9.926543	8.762555	9.045052	9.942917	10.506468	9.563044	11.912789	5.221727	7.769833
GSM5656364_treat	8.396253	7.61488	10.000687	9.021194	8.944609	9.895428	10.652613	9.80365	11.342089	5.127938	8.30309
GSM5656369_treat	8.325402	7.490283	9.891722	9.023012	9.050171	9.959742	10.53124	9.81328	11.873714	5.315746	8.221677
GSM5656376_treat	8.441411	6.650352	9.343982	8.817452	8.510257	9.188923	9.687038	10.035106	11.166455	5.309982	8.09979
GSM5656383_treat	8.216239	8.162892	10.091895	8.850607	8.687625	9.628236	10.533659	9.901359	11.32127	5.262366	7.692896
GSM5656385_treat	8.358799	7.355587	10.361311	8.909809	8.666574	9.57686	10.481092	10.17114	11.128129	5.269539	7.936599
GSM5656389_treat	8.057845	7.9841	10.096076	9.096422	8.56597	9.636673	10.550387	9.14401	11.166455	5.229218	7.554535
GSM5656394_treat	8.182951	8.763818	10.02382	9.239612	8.477841	9.718773	11.054189	9.776442	10.902368	5.102374	7.137013
GSM5656396_treat	9.229013	8.053032	10.222663	8.982101	8.753431	9.466357	10.444658	10.648606	12.023974	4.872804	7.774253
GSM5656398_treat	7.864118	8.840834	10.259363	9.345023	8.842099	9.668841	10.467376	9.809158	11.361081	4.806604	7.096383
GSM5656405_treat	8.818733	7.958055	10.298254	9.343443	8.977173	10.00699	10.596244	10.269803	11.461648	4.954819	8.228711
GSM5656410_treat	9.266816	8.063445	10.091088	8.760889	8.866214	9.347141	10.501868	10.155995	11.551018	5.281074	7.096383

GSM5656414_treat	8.742687	7.98305	9.819429	8.458812	8.45077	9.465272	10.069617	9.82621	11.995093	5.166828	7.987923
GSM5656417_treat	8.182239	8.222424	9.99693	9.161795	8.680359	9.44009	10.482294	9.239148	10.953528	5.369401	7.864118
GSM5656420_treat	8.52214	8.70146	10.260329	9.257818	8.180092	9.405694	10.562789	9.731782	11.394381	4.953086	7.511311
GSM5656423_treat	8.779984	8.008907	9.846784	8.203138	8.628402	9.373492	10.073787	9.73912	11.757489	5.282084	8.37474
GSM5656425_treat	8.149072	7.595779	9.83432	9.31321	8.871776	9.893129	10.350936	9.945245	11.168561	5.399104	8.069338
GSM5656428_treat	8.116502	8.535153	10.256516	9.389337	8.69046	9.824876	10.776825	9.550147	10.897495	4.748243	7.542489
GSM5656430_treat	8.272383	7.769833	9.794026	9.081471	9.25373	9.794692	10.244934	9.394251	11.365644	4.98591	7.968316
GSM5656437_treat	8.853173	7.696961	9.490316	8.68473	8.924675	9.750434	10.250942	9.956656	11.62292	4.813516	8.330556
GSM5656441_treat	8.550909	8.47095	10.046598	9.062735	8.55747	9.67936	10.692417	9.925008	11.485292	5.002099	7.644932
GSM5656445_treat	8.376244	8.482011	10.196964	9.065061	8.294425	9.67996	10.82717	9.939133	11.219664	5.160741	7.500093
GSM5656448_treat	8.328352	8.101543	9.863202	8.947702	8.668144	9.643991	10.278434	9.956656	11.498276	5.300693	8.136445
GSM5656450_treat	8.245327	8.90584	10.270739	9.455688	8.687625	10.001525	10.497408	9.718773	10.993128	5.183014	6.911722
GSM5656455_treat	8.208779	7.728921	10.463911	9.346636	9.133869	9.798801	10.641762	9.986651	11.139733	5.205516	7.233023
GSM5656463_treat	8.30891	8.327258	10.260329	9.006037	9.375118	10.120841	10.580391	9.99129	11.380084	4.858223	7.376397
GSM5656470_treat	8.408811	8.595357	10.014171	9.31478	8.645023	9.464129	10.784328	9.931676	10.998591	5.350678	7.164873
GSM5656471_treat	8.762149	8.048753	10.010974	9.127177	9.065061	9.563656	10.641762	10.147997	11.56738	5.26306	7.607731
GSM5656474_treat	8.497618	8.093838	9.978266	9.30495	8.754688	9.724032	10.590226	9.941381	11.446481	5.122099	7.709267
GSM5656477_treat	8.470191	8.510257	10.190567	9.199197	8.442162	9.992057	10.620066	10.374843	11.508563	5.333718	7.192215
GSM5656480_treat	8.555164	8.591872	10.151487	9.237641	8.845483	10.18015	10.685484	9.933976	11.451588	5.084257	7.150069
GSM5656482_treat	7.968658	8.113004	9.848226	8.709069	9.058562	9.514393	10.053148	9.622048	10.998591	5.426224	7.99033
GSM5656485_treat	7.96523	8.390703	10.019043	9.194846	8.299829	9.71486	10.582815	9.949023	11.065022	5.446219	8.005043
GSM5656488_treat	8.693342	8.371793	10.059625	9.296307	8.47095	9.751696	10.595059	10.276544	11.353915	5.337129	7.551898
GSM5656489_treat	9.119576	8.141368	10.049074	9.059475	9.043667	9.668841	10.552953	10.580391	11.508563	5.622911	7.596789
GSM5656492_treat	8.152936	7.469874	9.818802	8.747233	9.062288	9.736495	10.169417	9.525906	11.342089	5.007207	7.981024
GSM5656496_treat	8.579316	8.362833	10.031861	8.898495	8.56407	9.786729	10.555176	10.148875	11.43616	5.15532	7.789002
GSM5656500_treat	8.537115	9.075872	10.376933	9.492534	8.505269	9.843386	10.712879	10.162651	11.045108	4.943953	7.030973
GSM5656503_treat	8.943253	8.369171	10.926844	9.657132	8.769546	10.710194	10.778345	10.693808	11.923466	4.726647	7.463111
GSM5656506_treat	8.615491	8.914212	10.320706	9.086488	8.618935	9.517868	10.847089	10.198841	11.372691	5.406546	7.457627
GSM5656508_treat	8.265506	7.212153	10.212558	9.100203	8.909809	9.652744	10.3675	10.009333	11.245291	5.622223	8.264435
GSM5656514_treat	8.362833	8.603145	10.197884	9.58705	8.18366	10.207977	11.112311	10.315258	11.012483	5.15229	7.343295
GSM5656518_treat	8.487694	8.88122	10.134728	9.243648	8.53786	9.763057	10.87374	10.225527	11.409385	4.765544	7.379688
GSM5656530_treat	8.014382	7.984748	9.841957	9.29123	8.576224	9.634872	10.509957	9.394747	10.696467	5.25444	7.917458
GSM5656532_treat	8.410634	7.723556	10.062939	8.648184	9.093659	9.734423	10.989618	9.786048	11.498276	5.119305	7.581586
GSM5656534_treat	8.275155	8.298417	9.913146	8.873832	9.051123	9.555423	10.282346	9.561318	11.537615	4.952042	7.95121
GSM5656537_treat	8.552858	8.48084	10.109781	8.857504	8.517126	9.301395	10.475426	9.832234	11.274075	5.368422	7.554201
GSM5656542_treat	8.481235	8.68846	10.255529	9.442317	8.238289	9.711661	10.735286	9.649086	11.057792	4.709288	7.225831
GSM5656546_treat	8.482762	7.778095	9.90793	8.786605	8.950757	9.968193	10.322702	9.836428	11.474573	4.803698	8.039749
GSM5656548_treat	8.527118	6.91699	9.802249	8.996051	9.204185	9.652122	10.18015	9.301395	11.247566	5.115872	8.271258
GSM5656555_treat	8.897173	8.17405	9.942151	9.132883	8.677927	9.327007	10.544394	10.248102	10.926844	5.552886	7.770505
GSM5656558_treat	8.55012	8.638294	10.347849	9.202668	8.591082	10.117439	10.975562	10.84869	11.249925	5.450264	7.116761
GSM5656562_treat	8.63986	8.073261	10.350936	9.060921	9.25983	9.647821	10.468484	9.862475	11.156359	5.225085	7.354567
GSM5656564_treat	7.987204	8.019459	10.067999	8.876042	8.860983	9.535561	10.529922	8.763389	11.04882	5.473036	7.821244
GSM5656570_treat	8.794482	8.080912	9.908706	8.988844	8.902824	9.674973	10.485815	9.859627	10.972139	4.944298	7.56666

GSM5656575_treat	8.423763	8.075333	9.786048	8.654536	8.674685	9.500981	10.475426	9.505852	11.307668	5.166486	7.769833
GSM5656579_treat	8.465956	8.558594	10.399989	9.218556	8.814655	9.934713	10.621259	10.40746	11.634585	5.026704	7.548904
GSM5656584_treat	9.165271	8.614281	10.536125	8.660872	9.134358	9.521322	10.402164	9.777177	11.687218	4.950597	7.702698
GSM5656590_treat	8.199225	8.830565	10.350936	9.553106	8.731431	9.714288	11.037824	10.311929	11.041442	5.140491	7.162798
GSM5656594_treat	8.774943	8.516398	10.585254	9.642758	8.411748	9.9759	10.884757	10.196964	11.44393	5.106146	7.06732
GSM5656597_treat	7.753269	7.942084	9.796078	8.833548	9.265283	9.631851	10.169417	9.051598	11.145921	5.501782	7.903544
GSM5656599_treat	8.5691	8.614662	9.999222	8.880758	8.181164	9.505319	10.471975	10.075444	11.675378	5.466892	7.763421
GSM5656609_treat	8.541324	7.452218	10.617627	8.961913	9.083327	9.640898	10.124222	9.766459	11.699447	4.756205	7.763759
GSM5656614_treat	8.428981	7.621839	9.816096	8.716849	9.176535	9.886731	10.234962	9.904251	11.382451	5.001757	8.244947
GSM5656620_treat	8.077043	8.533991	10.426011	9.164254	8.448875	9.896928	10.640497	9.627644	11.118185	5.13843	7.376052
GSM5656622_treat	7.920847	7.942377	10.269803	8.664573	8.690067	9.481369	10.358257	9.27194	11.087347	4.957977	6.895744
GSM5656623_treat	8.672637	8.0953	10.61497	9.206083	8.638294	9.962039	10.810593	9.929449	11.464173	5.141835	7.471551
GSM5656629_treat	8.944609	8.161136	11.245291	9.796764	8.750502	10.354024	10.736718	10.060506	11.411895	5.314718	8.106066
GSM5656634_treat	7.712676	7.780184	9.958234	8.617039	8.675469	9.739771	10.12598	10.331599	11.365644	5.461077	7.897367
GSM5656643_treat	8.450373	8.27049	10.210726	9.248594	9.165271	9.652122	10.752297	9.988147	11.06682	5.259253	7.174907
GSM5656649_treat	8.997885	7.963874	10.389393	8.761315	8.90584	9.67996	9.936901	9.465272	11.380084	4.783409	7.447813
GSM5656652_treat	8.225586	7.710958	9.705307	8.978491	8.668535	9.350925	9.943667	9.763057	10.71995	5.823526	8.325784
GSM5656656_treat	8.508716	7.552903	9.904954	8.727809	9.390398	9.918428	10.205212	9.788033	11.474573	5.232972	8.206305
