# Supplementary material for: Prognostic and therapeutic implication of m6A methylation in Crohn disease
Source: Medicine (Baltimore). 2022 Dec 23;101(51):e32399. doi: 10.1097/MD.0000000000032399 (PMC9794314; doi:10.1097/MD.0000000000032399)
Supplement: Supplementary file 3 [file medi-101-e32399-s003.pdf]

Supplemental Table 3. The expression of characteristic genes in m6A regulators

ID	RBM15	WTAP	LRPPRC	YTHDF1	YTHDF3
GSM5656180_con	8.195646	9.490316	9.822183	8.933446	9.738499
GSM5656185_con	8.402181	9.798115	9.085628	9.204653	10.13743
GSM5656189_con	9.349861	9.548409	10.337597	9.202668	10.699004
GSM5656190_con	9.341857	9.960509	10.836292	9.101128	10.907465
GSM5656202_con	8.514494	9.836428	9.580974	9.03215	10.16398
GSM5656208_con	9.286637	9.992057	10.631469	9.364418	10.470778
GSM5656230_con	9.141092	9.420998	10.49957	9.137253	10.93873
GSM5656232_con	8.68237	9.564796	9.836428	9.133869	9.95122
GSM5656238_con	8.611861	9.810531	10.202473	9.274418	9.71486
GSM5656245_con	8.635174	9.566511	9.898468	9.111587	9.600884
GSM5656258_con	8.12487	9.591845	9.198238	8.778636	10.079404
GSM5656270_con	9.178869	9.484177	10.689705	9.424215	10.621259
GSM5656271_con	8.519859	9.61232	10.152344	9.279057	9.81121
GSM5656279_con	8.482011	9.735817	9.61232	9.007866	9.879639
GSM5656283_con	8.168483	9.510403	10.074607	9.044112	10.142775
GSM5656287_con	8.757975	9.676213	10.113097	9.402936	9.86678
GSM5656288_con	8.62246	9.982037	10.139176	9.386039	10.110577
GSM5656292_con	8.401764	9.614103	10.00457	9.141092	10.096076
GSM5656299_con	8.63634	9.667618	9.899188	9.133869	10.025462
GSM5656300_con	9.308513	9.519029	11.06682	9.384496	10.569012
GSM5656304_con	8.349293	9.802249	9.516179	9.009234	10.155995
GSM5656310_con	8.467074	9.583499	10.098665	9.14495	10.054751
GSM5656313_con	8.39662	9.782673	8.585605	8.962339	10.042485
GSM5656314_con	9.185441	9.59731	10.630173	9.188384	10.738281
GSM5656539_con	8.897173	9.934713	10.109781	9.409576	10.4381
GSM5656171_treat	9.061386	10.109781	10.138298	9.391474	10.30402
GSM5656174_treat	9.095475	9.82763	9.235548	9.160869	10.593831
GSM5656175_treat	9.27699	10.402164	9.52354	8.997885	10.005376
GSM5656177_treat	9.101626	10.109781	10.334647	8.117866	10.810593
GSM5656179_treat	9.069762	10.073787	9.757626	9.192872	10.197884
GSM5656183_treat	8.969099	10.129502	9.670031	8.972696	10.602545
GSM5656184_treat	8.793269	9.90793	10.256516	8.924222	10.170264
GSM5656186_treat	9.010091	10.027096	10.116532	9.289221	10.61497
GSM5656187_treat	8.945504	10.155025	9.790003	8.584036	10.711559
GSM5656192_treat	8.213804	9.866056	9.891722	8.471317	10.301157
GSM5656193_treat	9.168725	10.840867	9.690156	9.546624	10.257504
GSM5656195_treat	8.586375	9.664461	9.63846	9.227037	10.131143
GSM5656199_treat	8.854027	9.768392	9.67996	8.938364	10.467376
GSM5656200_treat	8.974914	9.752345	9.580974	8.952954	10.120841
GSM5656203_treat	9.33704	10.11485	9.660788	9.1599	10.473136
GSM5656207_treat	8.921251	9.773141	9.282064	9.085178	10.136531

GSM5656209_treat	8.517499	10.714212	9.200706	9.317324	10.117439
GSM5656212_treat	8.987963	10.123428	9.230023	9.731782	9.838553
GSM5656214_treat	9.196281	9.652744	9.539572	9.489777	10.509957
GSM5656217_treat	8.571087	9.738499	9.909465	8.728186	10.044104
GSM5656220_treat	9.225027	11.572902	9.848956	9.517868	10.54565
GSM5656223_treat	8.992885	10.439195	9.187923	9.449623	9.88952
GSM5656226_treat	8.697823	9.837184	9.499846	9.000637	10.312397
GSM5656228_treat	8.448875	9.747082	9.942151	8.571486	10.125075
GSM5656231_treat	9.558871	10.552953	9.979812	9.479725	10.496184
GSM5656235_treat	9.142997	9.953541	9.522972	9.428565	10.10461
GSM5656237_treat	9.301395	10.329562	9.316829	9.139655	10.444658
GSM5656240_treat	8.725779	9.49529	10.309798	9.14495	10.390464
GSM5656243_treat	8.600448	9.931676	9.430203	9.346094	10.015003
GSM5656246_treat	9.239612	9.485311	10.288314	9.249098	10.496184
GSM5656249_treat	9.03215	9.958993	10.197884	9.152173	10.493802
GSM5656254_treat	8.835628	9.559453	10.280355	9.101128	9.919168
GSM5656255_treat	8.879049	10.965403	9.557131	8.721267	10.541926
GSM5656259_treat	8.775377	9.920645	9.568943	9.780628	10.187719
GSM5656262_treat	9.155599	9.634273	9.95586	8.9437	10.579097
GSM5656264_treat	8.724167	9.265283	9.459064	8.658097	10.119953
GSM5656265_treat	8.942818	9.749112	10.130245	8.741057	10.384159
GSM5656267_treat	9.106328	9.948235	9.208453	9.532704	10.187719
GSM5656273_treat	9.99129	10.109781	10.387269	9.567107	9.962039
GSM5656274_treat	8.795289	10.158634	9.99129	9.060454	10.092791
GSM5656277_treat	9.372999	10.364464	9.975144	9.104434	10.596244
GSM5656280_treat	9.32964	10.417277	9.92577	8.809671	10.797698
GSM5656282_treat	9.124779	9.848226	9.552534	8.804635	10.464988
GSM5656285_treat	9.005133	9.989744	9.216121	9.230523	10.272732
GSM5656291_treat	8.534756	9.605701	9.788033	8.788254	10.24267
GSM5656294_treat	8.794882	10.015003	9.850374	8.82464	10.001525
GSM5656297_treat	8.407345	9.544261	9.971426	8.6868	10.100337
GSM5656303_treat	9.240616	10.067114	9.640322	9.245609	10.708868
GSM5656305_treat	8.730614	9.919168	9.034041	9.199675	10.276544
GSM5656309_treat	9.255287	9.498706	10.183966	9.27496	10.547934
GSM5656311_treat	9.308513	9.603844	9.287694	8.972696	10.337597
GSM5656316_treat	8.91862	10.424855	10.010974	9.27496	10.591388
GSM5656318_treat	8.714408	10.147086	10.239681	9.329114	10.38622
GSM5656321_treat	8.880345	10.824086	9.742392	9.354189	10.501868
GSM5656323_treat	8.784928	10.533659	9.610497	9.674331	10.384159
GSM5656325_treat	9.074902	10.129048	9.798115	9.362836	10.194161
GSM5656327_treat	9.121917	10.086795	9.902836	9.439552	10.394671
GSM5656329_treat	8.646981	10.125075	10.383151	9.371379	10.328517
GSM5656334_treat	8.835628	10.246394	9.801539	9.389337	10.183966

GSM5656335_treat	8.860983	10.461604	10.149751	9.413851	10.221747
GSM5656338_treat	8.941927	9.965824	9.64461	9.470252	10.177437
GSM5656342_treat	9.117201	10.918644	9.84539	9.432476	9.989744
GSM5656346_treat	8.906723	9.986651	9.851812	9.323442	10.881487
GSM5656348_treat	9.149771	10.012545	10.205212	9.390398	10.483518
GSM5656351_treat	9.639084	10.269803	9.755641	9.101626	10.42937
GSM5656353_treat	8.958331	10.10461	9.759648	9.372999	10.373807
GSM5656355_treat	8.678722	10.633964	9.563044	9.448492	10.305907
GSM5656359_treat	9.115374	10.656702	9.877499	8.94595	10.526488
GSM5656361_treat	9.041843	11.356345	9.473618	9.323981	10.246394
GSM5656365_treat	9.118128	10.358257	9.581576	9.280574	10.374843
GSM5656366_treat	8.944144	10.859629	9.200199	9.434129	10.205212
GSM5656368_treat	9.299343	10.991338	9.790721	9.615352	10.497408
GSM5656372_treat	9.454577	10.593831	10.00699	9.32964	10.689705
GSM5656374_treat	9.100664	10.119953	10.042485	8.842921	10.3021
GSM5656375_treat	9.49927	9.634872	9.580974	8.779094	10.736718
GSM5656380_treat	8.911636	11.198815	9.588263	8.978938	10.195108
GSM5656381_treat	8.895873	10.168531	10.126895	9.208955	10.085169
GSM5656384_treat	9.283092	10.626268	9.532108	9.295817	9.95122
GSM5656387_treat	8.861824	9.862475	9.095946	9.101128	9.533254
GSM5656388_treat	8.997885	9.486444	9.502039	9.006923	10.164925
GSM5656390_treat	9.395265	10.038451	9.352588	8.131952	10.989618
GSM5656392_treat	8.872585	10.497408	9.898468	9.184912	9.90793
GSM5656393_treat	9.254252	11.763732	9.239148	8.587124	10.424855
GSM5656395_treat	8.911187	9.628236	10.107216	9.268328	10.18015
GSM5656399_treat	9.049267	9.822183	9.687672	9.014222	10.05548
GSM5656400_treat	9.238678	10.602545	10.307895	9.023012	10.288314
GSM5656403_treat	9.235548	10.264164	9.781998	8.707418	10.597401
GSM5656406_treat	9.550147	11.124017	10.21992	8.786168	10.261355
GSM5656407_treat	9.027589	10.334647	10.259363	8.578119	10.775367
GSM5656409_treat	9.241116	10.130245	9.562474	9.11394	10.029454
GSM5656412_treat	8.885505	11.627299	9.224504	8.867066	10.012545
GSM5656416_treat	9.050171	9.894661	9.318799	9.17553	10.153267
GSM5656418_treat	9.109211	10.086795	10.209802	8.950757	10.374843
GSM5656421_treat	9.164254	9.751043	9.8475	9.017002	10.227358
GSM5656424_treat	9.139194	10.121762	9.587638	8.769546	10.086795
GSM5656426_treat	9.31478	10.133857	10.853619	7.947114	10.421688
GSM5656429_treat	9.279057	10.0613	9.633077	9.083799	10.61497
GSM5656431_treat	9.476908	10.80057	9.072034	8.576224	9.945961
GSM5656432_treat	8.56597	10.253766	9.751696	9.176979	10.068853
GSM5656434_treat	9.244615	10.307895	9.833623	9.07156	9.91695
GSM5656435_treat	9.285109	10.165812	9.439552	9.108257	10.239681
GSM5656439_treat	9.035893	10.106412	9.95122	8.928576	10.255529

GSM5656443_treat	9.003359	10.918644	10.025462	8.628005	10.61497
GSM5656444_treat	9.283092	10.466146	10.689705	8.462253	10.570222
GSM5656447_treat	9.225027	9.489235	10.049884	8.805067	10.557702
GSM5656451_treat	9.58416	10.416161	10.124222	9.367663	10.912057
GSM5656452_treat	9.224504	10.612491	9.740453	9.477513	10.513459
GSM5656454_treat	10.113097	10.544394	10.758133	8.684328	10.793233
GSM5656458_treat	8.64462	9.82621	9.316829	8.868785	10.289289
GSM5656460_treat	9.010091	9.907109	9.952044	9.328064	10.142775
GSM5656461_treat	9.177919	10.114003	10.360305	8.861824	10.766564
GSM5656464_treat	9.596098	10.41282	8.803808	9.475239	10.213498
GSM5656465_treat	9.126192	9.691395	9.992927	9.219048	10.16398
GSM5656467_treat	9.107295	10.15685	9.99447	9.308513	10.639254
GSM5656469_treat	9.302381	9.952823	9.564796	9.015145	10.13743
GSM5656473_treat	8.760889	9.882457	10.470778	9.422034	10.569012
GSM5656478_treat	9.048381	9.816096	9.522972	8.994661	10.406306
GSM5656481_treat	9.265283	10.194161	9.896928	8.895459	10.261355
GSM5656483_treat	9.304447	10.601288	9.945961	9.152659	10.776825
GSM5656486_treat	9.122371	10.219457	9.859627	9.400215	10.364464
GSM5656487_treat	10.703312	9.932072	9.095475	9.121917	10.556426
GSM5656490_treat	9.395265	10.491484	10.163066	8.718854	10.651277
GSM5656493_treat	8.825069	9.95586	9.410697	9.390934	9.941381
GSM5656495_treat	8.769952	9.54076	9.990489	9.375118	10.187719
GSM5656498_treat	8.681195	10.173009	9.3986	8.96321	10.165812
GSM5656501_treat	9.30039	10.227358	10.277444	9.419391	10.514599
GSM5656504_treat	9.379001	10.320706	10.428312	9.238146	10.755174
GSM5656505_treat	8.896282	10.782909	9.539572	9.181363	10.30402
GSM5656509_treat	9.254782	10.210726	9.820813	9.251653	10.403258
GSM5656511_treat	9.433021	10.243587	10.42937	8.665001	10.481092
GSM5656513_treat	9.184912	9.678765	10.116532	8.912483	10.207073
GSM5656516_treat	9.295817	10.422725	10.380122	9.05578	10.464988
GSM5656517_treat	9.53729	9.718773	9.738499	9.108257	10.411836
GSM5656520_treat	8.691274	10.109781	9.022587	9.428565	10.008562
GSM5656521_treat	8.903712	11.211381	9.137253	8.962339	9.973622
GSM5656522_treat	8.996458	11.034182	9.724032	8.782416	10.506468
GSM5656524_treat	8.901989	9.996089	9.639084	8.616679	10.393582
GSM5656525_treat	9.141092	10.464988	9.896186	9.555423	9.719403
GSM5656527_treat	9.021659	9.955108	10.526488	9.076326	10.591388
GSM5656528_treat	9.789315	10.350936	9.580389	9.145931	10.208923
GSM5656531_treat	9.439003	10.351967	10.136531	8.927689	10.427188
GSM5656533_treat	8.429763	9.939133	9.764405	9.072034	10.570222
GSM5656535_treat	9.052984	9.794692	9.931676	9.347708	9.969007
GSM5656536_treat	9.020687	10.009333	9.658942	9.081922	10.352968
GSM5656541_treat	8.886328	9.825555	9.121917	9.262773	10.16398

GSM5656543_treat	9.367663	9.63846	10.372761	8.898495	9.868984
GSM5656545_treat	8.752991	9.954353	9.590032	9.465818	10.481092
GSM5656549_treat	9.658942	11.114146	9.790003	9.549593	9.611092
GSM5656551_treat	8.998324	10.673695	9.464683	9.663275	9.838553
GSM5656554_treat	8.969099	9.888851	9.876792	9.111587	10.435837
GSM5656556_treat	9.150712	10.276544	10.501868	8.994661	10.636552
GSM5656559_treat	8.804249	10.019043	9.952044	9.515576	9.999905
GSM5656563_treat	8.762954	9.789315	8.91862	9.270423	10.307895
GSM5656565_treat	8.783227	9.945245	9.522972	9.290225	10.337597
GSM5656567_treat	8.964995	10.138298	9.930209	9.52354	10.031041
GSM5656569_treat	8.880758	10.217084	10.066293	9.43076	9.965091
GSM5656571_treat	8.839105	9.917677	9.392	9.072034	10.207073
GSM5656573_treat	8.642235	11.178435	8.933005	9.196281	10.358257
GSM5656576_treat	8.675469	10.381147	9.727843	9.59731	9.746373
GSM5656578_treat	9.112067	9.690156	9.612904	9.079095	10.167641
GSM5656581_treat	8.901559	9.78541	10.273645	9.022587	10.29725
GSM5656583_treat	8.635542	10.374843	9.574389	9.191404	9.8475
GSM5656585_treat	8.583271	9.579746	10.402164	8.99975	9.831592
GSM5656588_treat	8.752574	9.465818	9.429109	9.022102	10.527563
GSM5656589_treat	8.741862	10.222663	10.203395	9.190888	10.23593
GSM5656591_treat	8.509492	10.165812	10.184926	9.195802	9.985152
GSM5656595_treat	9.011933	10.763803	9.269877	9.298858	10.093601
GSM5656596_treat	8.984339	10.024642	9.86391	9.127177	10.449121
GSM5656600_treat	9.468633	10.813648	9.775784	9.003797	10.608863
GSM5656601_treat	8.63634	9.765097	9.948235	9.066051	10.264164
GSM5656602_treat	8.990224	10.546816	9.387703	9.247076	10.825685
GSM5656603_treat	8.858391	9.630013	9.665725	8.880758	10.540817
GSM5656604_treat	9.080058	9.648427	9.649708	9.447386	10.324687
GSM5656606_treat	8.384887	9.415485	9.905629	9.26034	9.419943
GSM5656608_treat	9.147393	9.792099	9.894661	9.408459	10.151487
GSM5656611_treat	9.045979	10.147997	9.425318	9.500413	10.320706
GSM5656612_treat	9.159419	10.270739	9.835042	9.350925	10.325654
GSM5656615_treat	8.970918	10.119953	9.61723	9.504751	10.046598
GSM5656617_treat	9.286133	10.220816	9.40184	9.128149	10.097812
GSM5656619_treat	8.662093	9.876015	9.497568	9.323442	9.913146
GSM5656621_treat	9.24013	10.187719	10.031861	9.138677	10.165812
GSM5656624_treat	9.12004	10.483518	9.822183	9.430203	10.286379
GSM5656625_treat	9.153151	10.312865	9.659557	9.636673	10.029454
GSM5656627_treat	8.733869	9.983595	9.278493	9.32964	9.854597
GSM5656631_treat	9.354712	9.9497	9.116752	9.289716	9.982827
GSM5656632_treat	8.693342	9.730508	8.886328	9.125735	10.111389
GSM5656635_treat	9.234565	9.87259	8.794882	9.205127	10.259363
GSM5656637_treat	9.648427	10.250942	9.614103	9.228044	10.406306

GSM5656639_treat	8.907161	10.095244	9.487566	9.528715	10.203395
GSM5656641_treat	9.238678	10.872098	9.557713	9.513294	10.175685
GSM5656645_treat	9.207983	10.182985	9.738499	9.444	10.081
GSM5656647_treat	9.353118	10.086795	9.663275	9.377283	10.511098
GSM5656648_treat	8.967325	9.985872	10.127747	8.806304	9.591845
GSM5656653_treat	8.800019	9.591247	9.52647	9.302909	10.501868
GSM5656655_treat	9.364418	11.03962	9.543106	9.535561	10.03595
GSM5656657_treat	9.273463	10.132033	9.68123	9.082827	10.134728
GSM5656173_treat	9.131446	9.864646	9.38879	9.270423	10.626268
GSM5656182_treat	8.805888	9.905629	9.411246	9.11346	10.303079
GSM5656191_treat	7.900429	9.792099	10.183966	7.719151	10.228275
GSM5656194_treat	8.878191	9.695887	9.782673	9.050645	10.279365
GSM5656196_treat	8.483155	9.437959	9.742392	9.106328	10.326612
GSM5656198_treat	8.868785	10.051478	9.97671	8.889795	10.517006
GSM5656205_treat	9.58222	10.009333	10.150591	9.088323	10.309798
GSM5656210_treat	9.255786	10.816578	9.973622	8.95383	10.494973
GSM5656211_treat	9.261323	9.933976	9.449054	8.839105	10.965403
GSM5656215_treat	9.011461	9.83432	9.389877	9.286133	10.501868
GSM5656218_treat	8.222763	9.848956	9.72651	8.414319	10.177437
GSM5656219_treat	9.243114	10.041667	9.853889	9.069301	10.5053
GSM5656222_treat	8.801648	9.557131	9.376701	8.943253	10.369592
GSM5656227_treat	9.134358	9.925008	9.977483	9.296856	10.492597
GSM5656234_treat	8.952505	9.828983	10.184926	9.182912	10.207977
GSM5656236_treat	9.743044	10.464988	9.388226	8.599263	11.065022
GSM5656239_treat	8.375473	9.990489	9.868984	7.99864	10.449121
GSM5656241_treat	8.116502	9.769792	10.300132	8.06863	10.635242
GSM5656244_treat	8.755898	9.550704	9.217607	9.030299	10.169417
GSM5656247_treat	8.518308	10.034289	10.380122	8.184017	10.194161
GSM5656251_treat	9.407888	9.667618	10.443456	8.780382	11.349203
GSM5656253_treat	8.68315	9.446815	9.85893	9.130527	10.017404
GSM5656256_treat	8.708658	9.780628	10.182985	8.032725	10.527563
GSM5656263_treat	8.701853	9.916174	10.132033	8.085425	10.56648
GSM5656268_treat	9.100203	10.077034	8.980723	9.376701	10.527563
GSM5656272_treat	8.934324	9.965824	10.0613	8.652949	10.434805
GSM5656275_treat	8.738158	9.854597	9.728502	8.897605	10.2853
GSM5656278_treat	9.009234	10.022291	10.040473	8.957449	10.517006
GSM5656281_treat	9.144447	9.67308	9.352588	8.878191	10.439195
GSM5656286_treat	8.92378	9.746373	9.671219	9.181859	10.195108
GSM5656289_treat	8.817049	9.276502	10.440291	9.40184	10.571467
GSM5656293_treat	9.093157	9.573131	10.234962	9.169648	10.617627
GSM5656296_treat	8.375095	9.985152	10.103868	8.01643	10.309798
GSM5656301_treat	8.952063	9.992057	9.419943	9.139655	10.616253
GSM5656307_treat	8.861824	9.732475	9.444591	9.016103	10.317785

GSM5656308_treat	8.313977	9.828337	9.905629	8.344515	10.031041
GSM5656320_treat	8.926818	10.224611	9.820134	9.392568	10.587754
GSM5656324_treat	9.235061	10.072035	9.755641	9.705307	10.451364
GSM5656326_treat	9.118614	9.978266	10.220816	9.381752	10.276544
GSM5656328_treat	9.069762	9.781319	9.382807	9.270423	10.458181
GSM5656331_treat	8.672233	10.485815	9.792715	9.533804	10.254613
GSM5656332_treat	8.822528	9.208453	9.735817	8.905422	10.402164
GSM5656336_treat	9.054837	9.694542	9.695192	9.305977	10.3306
GSM5656340_treat	9.749744	10.231261	10.059625	9.888851	10.128593
GSM5656343_treat	8.731431	9.870357	9.551943	9.258319	10.248102
GSM5656345_treat	9.253211	10.045721	10.091088	9.210985	10.125075
GSM5656349_treat	9.207983	9.786729	9.289716	9.439552	10.265975
GSM5656350_treat	9.251653	10.046598	9.758982	9.259307	10.639254
GSM5656354_treat	9.03215	9.759648	9.386861	9.26937	10.211679
GSM5656357_treat	8.908471	9.882457	9.970613	9.242606	10.271728
GSM5656362_treat	8.594957	9.687672	9.550147	9.360649	10.540817
GSM5656363_treat	9.110171	9.835042	9.381184	9.257818	10.188646
GSM5656367_treat	8.843356	10.490337	9.379001	9.521322	10.228275
GSM5656370_treat	8.728985	9.548995	10.114003	8.957918	10.756657
GSM5656371_treat	9.225548	10.151487	10.358257	9.507525	10.758133
GSM5656373_treat	8.720853	9.605107	9.848956	8.752574	10.396812
GSM5656377_treat	9.466357	9.614738	9.345023	8.768696	10.80914
GSM5656378_treat	9.404039	9.993709	10.167641	8.640261	10.721232
GSM5656379_treat	9.094124	10.557702	9.916174	8.669753	10.677625
GSM5656382_treat	8.773732	9.588868	9.831592	8.701057	10.561626
GSM5656386_treat	9.661406	10.354024	8.769546	8.996458	10.266919
GSM5656391_treat	9.412835	10.326612	9.875275	8.788254	10.387269
GSM5656397_treat	8.656128	9.510982	10.18957	8.398095	10.490337
GSM5656401_treat	9.159419	9.781998	9.444591	8.796159	10.018234
GSM5656402_treat	8.755476	9.616618	9.727843	9.182912	10.478749
GSM5656404_treat	9.45177	10.4381	10.006103	8.406216	10.818144
GSM5656408_treat	8.907582	9.798115	9.538994	8.640261	10.296257
GSM5656411_treat	9.078649	10.385216	9.563656	8.853173	10.300132
GSM5656413_treat	9.238678	10.099493	9.78541	8.366198	10.887934
GSM5656415_treat	9.420447	10.169417	9.497568	8.916844	10.611276
GSM5656419_treat	8.871376	9.855284	9.625196	9.000158	10.082646
GSM5656422_treat	8.945038	9.678765	9.916174	9.045979	10.090221
GSM5656427_treat	9.524097	9.555971	10.526488	8.246073	10.707421
GSM5656433_treat	8.849337	10.119056	8.986198	9.209463	10.289289
GSM5656436_treat	9.116752	10.374843	10.212558	8.597304	10.620066
GSM5656438_treat	9.44009	10.38832	10.089347	8.596543	10.892709
GSM5656440_treat	9.117201	10.193283	9.73379	8.982101	10.462712
GSM5656442_treat	8.911636	9.68123	9.557131	8.706628	10.624942

GSM5656446_treat	9.562474	9.376176	9.790003	8.309286	10.677625
GSM5656449_treat	9.547286	10.086795	9.763057	9.272446	10.723953
GSM5656453_treat	9.280574	9.956656	9.510982	9.223017	10.585254
GSM5656456_treat	9.971426	10.237802	9.913146	8.748834	10.508729
GSM5656457_treat	8.784928	9.668213	9.640322	8.895873	10.477623
GSM5656459_treat	8.94944	9.837843	9.899188	9.372999	10.136531
GSM5656462_treat	8.89421	9.99129	9.816721	9.034516	10.562789
GSM5656466_treat	9.056248	9.844753	10.293282	9.181859	10.440291
GSM5656468_treat	8.953403	9.590614	9.702748	9.104434	10.372761
GSM5656472_treat	9.058096	10.018234	9.639084	9.174011	10.144546
GSM5656475_treat	9.1677	10.332578	10.213498	9.412835	10.264164
GSM5656476_treat	8.960109	10.418387	8.931685	8.637077	10.474264
GSM5656479_treat	9.37084	10.015003	9.400769	8.855751	10.473136
GSM5656484_treat	8.861824	9.947486	9.204653	9.170128	10.224611
GSM5656491_treat	9.233591	10.397857	9.758314	8.813396	10.376933
GSM5656494_treat	9.259307	10.218993	9.688295	9.161321	10.766564
GSM5656497_treat	9.250085	9.969773	9.490316	8.629194	10.424855
GSM5656499_treat	9.273918	10.300132	10.211679	9.115836	10.303079
GSM5656502_treat	9.16676	10.027096	9.766459	9.068842	10.514599
GSM5656507_treat	9.265785	10.269803	9.818114	8.733869	10.872098
GSM5656510_treat	9.459639	10.387269	10.401068	9.120531	10.561626
GSM5656512_treat	8.984794	9.846078	9.067417	9.045052	10.474264
GSM5656515_treat	9.214004	10.322702	9.987401	9.071094	10.281357
GSM5656519_treat	8.94989	10.39146	9.534988	9.219573	9.978266
GSM5656523_treat	8.971831	9.913916	9.716143	8.579695	10.265047
GSM5656526_treat	9.247618	9.639084	9.70344	8.669346	10.19236
GSM5656529_treat	9.941381	9.977483	9.494708	8.721267	10.53124
GSM5656538_treat	8.980723	10.111389	9.521872	8.78282	10.229252
GSM5656540_treat	9.127177	9.662614	9.404582	9.041381	10.251868
GSM5656544_treat	9.114904	9.9497	10.12598	9.118614	10.460447
GSM5656547_treat	8.981183	9.525906	9.773141	9.200706	10.231261
GSM5656550_treat	8.46943	9.173543	9.774414	8.462253	10.041667
GSM5656552_treat	8.731431	9.567715	9.876792	8.462628	10.3675
GSM5656553_treat	9.02892	9.895428	9.83432	8.700245	10.349955
GSM5656557_treat	9.221031	10.015837	9.608719	8.945504	10.579097
GSM5656560_treat	8.912039	9.934713	9.867489	8.729393	10.563991
GSM5656561_treat	8.883743	9.456217	9.652122	9.104885	10.513459
GSM5656566_treat	8.910268	10.042485	10.314723	9.356304	10.310944
GSM5656568_treat	8.676298	9.987401	9.719403	9.391474	10.252837
GSM5656572_treat	9.03866	9.885983	10.315792	8.800019	10.502984
GSM5656574_treat	8.766662	9.757626	10.049884	8.984794	10.21437
GSM5656577_treat	8.568315	9.767768	10.689705	8.377346	10.427188
GSM5656580_treat	8.963645	9.838553	10.098665	8.887629	10.250942

GSM5656582_treat	8.734743	9.921343	9.730508	9.259307	9.818114
GSM5656586_treat	8.681195	9.965824	9.789315	9.197272	10.187719
GSM5656587_treat	9.079586	9.455688	9.687672	8.978059	10.475426
GSM5656592_treat	8.932117	9.68319	9.943667	8.996914	9.986651
GSM5656593_treat	8.919531	10.335598	8.93656	9.271459	10.119505
GSM5656598_treat	8.924222	9.878223	9.536713	8.979379	10.049074
GSM5656605_treat	8.927255	9.822183	9.525906	9.202668	10.422725
GSM5656607_treat	9.096422	9.45177	9.860362	8.968659	10.044104
GSM5656610_treat	8.843775	9.788659	9.191404	9.226537	10.282346
GSM5656613_treat	9.107295	9.964318	8.641045	8.938813	10.223599
GSM5656616_treat	9.021194	9.807784	9.90793	9.096422	10.351967
GSM5656618_treat	8.798324	9.384496	9.485311	8.994661	10.173911
GSM5656626_treat	9.265283	9.83432	9.190888	8.926818	10.234962
GSM5656628_treat	8.665001	9.892426	8.857972	9.128149	10.436913
GSM5656630_treat	9.134858	9.988147	9.308012	9.033593	10.090221
GSM5656633_treat	9.020687	9.998478	8.98754	9.001976	10.343656
GSM5656636_treat	9.784717	9.807784	9.720091	9.153151	10.3675
GSM5656638_treat	9.278493	9.716792	9.2563	8.829692	10.188646
GSM5656640_treat	8.852292	9.31321	9.234078	8.972271	10.368511
GSM5656642_treat	8.912483	9.807098	9.543656	9.022587	10.270739
GSM5656644_treat	9.12004	9.916174	9.92577	9.292246	10.017404
GSM5656646_treat	8.922063	9.960509	9.767768	9.250608	10.546816
GSM5656650_treat	9.615962	10.521822	10.658019	8.978059	9.915389
GSM5656651_treat	8.496139	9.465818	9.512121	8.842921	10.128593
GSM5656654_treat	9.006037	10.088094	9.384496	9.393092	10.489217
GSM5656658_treat	8.969564	9.678765	9.36342	8.902824	10.191482
GSM5656170_treat	8.944609	9.898468	10.057983	9.066531	10.284303
GSM5656172_treat	8.466714	10.078611	10.224611	8.839956	10.440291
GSM5656176_treat	8.984339	10.079404	9.737195	9.125735	10.338583
GSM5656178_treat	8.838244	9.711661	9.47414	8.88122	10.360305
GSM5656181_treat	9.131446	10.225527	10.043282	8.049128	10.656702
GSM5656188_treat	9.243114	10.11825	10.243587	8.998324	10.596244
GSM5656197_treat	8.838675	10.016611	10.062939	8.215517	10.596244
GSM5656201_treat	8.734743	9.676213	10.031041	9.294832	10.163066
GSM5656204_treat	9.010578	10.110577	9.775096	8.869614	10.433745
GSM5656206_treat	8.796592	9.914658	9.942151	8.691274	10.397857
GSM5656213_treat	8.99975	9.968193	9.957466	9.425318	10.136531
GSM5656216_treat	9.235548	9.925008	10.0449	9.010578	10.295246
GSM5656221_treat	9.002455	10.205212	9.754328	7.947788	10.689705
GSM5656224_treat	8.649394	10.083476	10.010174	9.127177	9.9497
GSM5656225_treat	8.745166	9.993709	9.576235	8.825455	10.11825
GSM5656229_treat	8.797895	9.913146	10.090221	8.99975	10.255529
GSM5656233_treat	8.729393	10.15412	10.254613	8.895054	10.178342

GSM5656242_treat	8.574248	10.043282	10.940317	9.522972	9.90065
GSM5656248_treat	8.485803	9.790003	10.260329	8.348922	9.913146
GSM5656250_treat	9.073934	9.608719	9.525283	8.945504	10.368511
GSM5656252_treat	9.100664	10.250033	10.390464	7.874981	10.649872
GSM5656257_treat	9.212002	9.577388	9.554296	9.03215	9.985872
GSM5656260_treat	9.172084	10.224611	10.2853	8.477841	10.801985
GSM5656261_treat	8.658097	9.698344	10.366439	9.006037	10.248102
GSM5656266_treat	8.972271	9.910936	9.731782	8.696201	10.181093
GSM5656269_treat	8.750502	9.654603	10.371642	9.458521	10.366439
GSM5656276_treat	9.157485	10.360305	10.23877	9.221516	10.394671
GSM5656284_treat	8.618554	9.881728	9.772497	9.30191	10.196044
GSM5656290_treat	8.671381	9.844081	10.502984	9.40131	9.91695
GSM5656295_treat	9.436317	9.781998	10.145373	9.433021	10.196964
GSM5656298_treat	8.865805	9.671219	10.195108	8.400668	10.2853
GSM5656302_treat	9.349861	10.404224	9.825555	8.654919	10.591388
GSM5656306_treat	8.980723	9.678112	9.766459	9.30751	10.172078
GSM5656312_treat	8.628801	9.90211	10.772393	8.125566	10.363404
GSM5656315_treat	8.747608	10.053148	9.965091	9.622048	10.408582
GSM5656317_treat	9.5196	9.380058	10.173009	8.602404	11.017876
GSM5656319_treat	8.859236	9.774414	9.798115	9.284603	10.316816
GSM5656322_treat	8.991598	10.090221	9.927296	9.53729	10.321746
GSM5656330_treat	8.646597	9.857538	9.754978	9.261323	10.138298
GSM5656333_treat	8.859643	9.908706	9.893129	8.990674	10.313332
GSM5656337_treat	9.707222	10.601288	10.491484	8.964995	10.842356
GSM5656339_treat	8.526711	9.853171	9.995275	9.023012	10.128593
GSM5656341_treat	8.693342	9.948235	9.572527	9.182383	10.287341
GSM5656344_treat	8.794089	9.575629	9.603287	9.090259	10.529922
GSM5656347_treat	9.280087	10.310944	10.090221	8.975335	10.900752
GSM5656352_treat	8.530926	9.640322	9.475794	9.252672	10.518216
GSM5656356_treat	9.067417	10.129502	9.551943	9.488658	10.317785
GSM5656358_treat	9.023939	9.441722	9.632447	9.110171	10.339566
GSM5656360_treat	8.762555	9.926543	9.563044	9.045052	10.506468
GSM5656364_treat	9.021194	10.000687	9.80365	8.944609	10.652613
GSM5656369_treat	9.023012	9.891722	9.81328	9.050171	10.53124
GSM5656376_treat	8.817452	9.343982	10.035106	8.510257	9.687038
GSM5656383_treat	8.850607	10.091895	9.901359	8.687625	10.533659
GSM5656385_treat	8.909809	10.361311	10.17114	8.666574	10.481092
GSM5656389_treat	9.096422	10.096076	9.14401	8.56597	10.550387
GSM5656394_treat	9.239612	10.02382	9.776442	8.477841	11.054189
GSM5656396_treat	8.982101	10.222663	10.648606	8.753431	10.444658
GSM5656398_treat	9.345023	10.259363	9.809158	8.842099	10.467376
GSM5656405_treat	9.343443	10.298254	10.269803	8.977173	10.596244
GSM5656410_treat	8.760889	10.091088	10.155995	8.866214	10.501868

GSM5656414_treat	8.458812	9.819429	9.82621	8.45077	10.069617
GSM5656417_treat	9.161795	9.99693	9.239148	8.680359	10.482294
GSM5656420_treat	9.257818	10.260329	9.731782	8.180092	10.562789
GSM5656423_treat	8.203138	9.846784	9.73912	8.628402	10.073787
GSM5656425_treat	9.31321	9.83432	9.945245	8.871776	10.350936
GSM5656428_treat	9.389337	10.256516	9.550147	8.69046	10.776825
GSM5656430_treat	9.081471	9.794026	9.394251	9.25373	10.244934
GSM5656437_treat	8.68473	9.490316	9.956656	8.924675	10.250942
GSM5656441_treat	9.062735	10.046598	9.925008	8.55747	10.692417
GSM5656445_treat	9.065061	10.196964	9.939133	8.294425	10.82717
GSM5656448_treat	8.947702	9.863202	9.956656	8.668144	10.278434
GSM5656450_treat	9.455688	10.270739	9.718773	8.687625	10.497408
GSM5656455_treat	9.346636	10.463911	9.986651	9.133869	10.641762
GSM5656463_treat	9.006037	10.260329	9.99129	9.375118	10.580391
GSM5656470_treat	9.31478	10.014171	9.931676	8.645023	10.784328
GSM5656471_treat	9.127177	10.010974	10.147997	9.065061	10.641762
GSM5656474_treat	9.30495	9.978266	9.941381	8.754688	10.590226
GSM5656477_treat	9.199197	10.190567	10.374843	8.442162	10.620066
GSM5656480_treat	9.237641	10.151487	9.933976	8.845483	10.685484
GSM5656482_treat	8.709069	9.848226	9.622048	9.058562	10.053148
GSM5656485_treat	9.194846	10.019043	9.949023	8.299829	10.582815
GSM5656488_treat	9.296307	10.059625	10.276544	8.47095	10.595059
GSM5656489_treat	9.059475	10.049074	10.580391	9.043667	10.552953
GSM5656492_treat	8.747233	9.818802	9.525906	9.062288	10.169417
GSM5656496_treat	8.898495	10.031861	10.148875	8.56407	10.555176
GSM5656500_treat	9.492534	10.376933	10.162651	8.505269	10.712879
GSM5656503_treat	9.657132	10.926844	10.693808	8.769546	10.778345
GSM5656506_treat	9.086488	10.320706	10.198841	8.618935	10.847089
GSM5656508_treat	9.100203	10.212558	10.009333	8.909809	10.3675
GSM5656514_treat	9.58705	10.197884	10.315258	8.18366	11.112311
GSM5656518_treat	9.243648	10.134728	10.225527	8.53786	10.87374
GSM5656530_treat	9.29123	9.841957	9.394747	8.576224	10.509957
GSM5656532_treat	8.648184	10.062939	9.786048	9.093659	10.989618
GSM5656534_treat	8.873832	9.913146	9.561318	9.051123	10.282346
GSM5656537_treat	8.857504	10.109781	9.832234	8.517126	10.475426
GSM5656542_treat	9.442317	10.255529	9.649086	8.238289	10.735286
GSM5656546_treat	8.786605	9.90793	9.836428	8.950757	10.322702
GSM5656548_treat	8.996051	9.802249	9.301395	9.204185	10.18015
GSM5656555_treat	9.132883	9.942151	10.248102	8.677927	10.544394
GSM5656558_treat	9.202668	10.347849	10.84869	8.591082	10.975562
GSM5656562_treat	9.060921	10.350936	9.862475	9.25983	10.468484
GSM5656564_treat	8.876042	10.067999	8.763389	8.860983	10.529922
GSM5656570_treat	8.988844	9.908706	9.859627	8.902824	10.485815

GSM5656575_treat	8.654536	9.786048	9.505852	8.674685	10.475426
GSM5656579_treat	9.218556	10.399989	10.40746	8.814655	10.621259
GSM5656584_treat	8.660872	10.536125	9.777177	9.134358	10.402164
GSM5656590_treat	9.553106	10.350936	10.311929	8.731431	11.037824
GSM5656594_treat	9.642758	10.585254	10.196964	8.411748	10.884757
GSM5656597_treat	8.833548	9.796078	9.051598	9.265283	10.169417
GSM5656599_treat	8.880758	9.999222	10.075444	8.181164	10.471975
GSM5656609_treat	8.961913	10.617627	9.766459	9.083327	10.124222
GSM5656614_treat	8.716849	9.816096	9.904251	9.176535	10.234962
GSM5656620_treat	9.164254	10.426011	9.627644	8.448875	10.640497
GSM5656622_treat	8.664573	10.269803	9.27194	8.690067	10.358257
GSM5656623_treat	9.206083	10.61497	9.929449	8.638294	10.810593
GSM5656629_treat	9.796764	11.245291	10.060506	8.750502	10.736718
GSM5656634_treat	8.617039	9.958234	10.331599	8.675469	10.12598
GSM5656643_treat	9.248594	10.210726	9.988147	9.165271	10.752297
GSM5656649_treat	8.761315	10.389393	9.465272	8.90584	9.936901
GSM5656652_treat	8.978491	9.705307	9.763057	8.668535	9.943667
GSM5656656_treat	8.727809	9.904954	9.788033	9.390398	10.205212
