# Supplementary material for: Prognostic and therapeutic implication of m6A methylation in Crohn disease
Source: Medicine (Baltimore). 2022 Dec 23;101(51):e32399. doi: 10.1097/MD.0000000000032399 (PMC9794314; doi:10.1097/MD.0000000000032399)
Supplement: Supplementary file 5 [file medi-101-e32399-s005.pdf]

Supplemental Table 5. Immune cell set

Activated.B.cell	Activated.CD4.T.cell	Activated.CD8.T.cell	Activated.dendritic.cell	CD56bright.natural.killer.cell	CD56dim.natural.killer.cell	Eosinophil	Gamma.delta.T.cell	Immature.B.cell	Immature.dendritic.cell	MDSC	Macrophage	Mast.cell	Monocyte	Natural.killer.T.cell	Natural.killer.cell	Neutrophil	Plasmacytoid.dendritic.cell	Regulatory.T.cell	T.follicular.helper.cell	Type.1.T.helper.cell	Type.17.T.helper.cell	Type.2.T.helper.cell
ADAM28	AIM2	ADRM1	ABCD1	ABAT	CYP27A1	GIPR	ACP5	CD22	ACADM	CCR2	AIF1	ADAMTS3	ASGR2	BTN2A2	AKT3	CREB5	CBX6	CCL3L1	B3GAT1	CD70	IL17A	ASB2
CD180	BIRC3	AHSA1	C1QC	C11orf75	DDX55	KRT18P50	AQP9	CYBB	AHCYL1	CD14	CCL1	CPA3	CFP	CD101	AXL	CDA	DAB2	CD72	CDK5R1	TBX21	IL17RA	CSRP2
CD79B	BRIP1	C1GALT1C1	CAPG	C5orf15	DYRK2	LRMP	BTN3A2	FAM129C	ALDH1A2	CD2	CCL14	CMA1	ASGR1	CD109	BST2	CHST15	DDX17	CLEC5A	PDCD1	ADAM8	C2CD4A	DAPK1
BLK	CCL20	CCT6B	CCL3L3	CDHR1	RPL37A	FOSB	C1orf54	FCRL1	ALDH3A2	CD86	CCL23	CTSG	CD1D	CNPY3	CDH2	S100A12	HIGD1A	FOXP3	BCL6	AHCYL2	C2CD4B	DLC1
CD19	CCL4	CD37	CD207	DCAF12	NOTCH3	RRP12	CARD8	FCRL3	ALDH9A1	CXCR4	CCL26	ARHGAP15	UPK3A	CNPY4	CRTAM	APOBEC3A	IDH3A	ITGA4	CD200	ALCAM	CA2	DNAJC12
MS4	CCL5	CD3D	CD302	DYNLL1	AKR7A	GP	CCL1	FCR	ALOX	FC	CD	CP	AC	CREB	CSF2	CA	IL3RA	L1C	CD83	B3GA	CCDC	DUSP

A1					3	R183	8	L5	15	GR2A	300LB	M	TG1	1	RA	SP5		AM		LNT1	65	6
TNFRSF17	CCNB1	CD3E	ATP5B	GPR137B	GPRC5C	NR4A3	CD209	FCRLA	AMT	FCGR2B	CNR1	FCN1	ANXA5	CRTC2	CTSZ	MMP25	MAGED1	LIPA	CD84	BBS12	CEACAM3	GNAI1
IGHM	CCR7	CD3G	ATP5L	HCP5	GRIN1	ST3GAL6	CD33	HDAC9	ARL1	FCGR3A	CNR2	FTL	ATP6V1B2	CRTC3	CXCL1	HAL	NUCB2	LRP1	FGF2	BST1	IL17C	LAMP3
GNG7	DUSP2	CD69	ATP6V1A	HDGFRP2	HLA-E	DEPDC5	CD36	HLA-DQA1	ATIC	FERMT3	EIF1	HSPA6	CFL1	CSF2	CYTH1	C1orf183	OFD1	LRRC42	GPR18	CD151	IL17F	NRP2
MICAL3	ESCO2	CD8A	BCL2L1	KRT86	PORCN	PDE6C	CDK5	HVCN1	ATP5A1	GPSM3	EIF4A1	ITGA9	DAZAP2	KLRC1	DAXX	FFAR2	OGT	MARCO	CEBPA	CD47	IL17RC	OSBPL1A
SPIB	ETS1	CETN3	C1QB	MLST8	PSMC4	PKD2L2	IL10RB	KIAA0226	CAPZA1	IL18BP	FPR1	RNASE3	CTBS	FUT4	DGKH	MAK	PDIA4	MMP12	CECR1	CD48	IL17RE	PDE4B
HLA-DOB	EXO1	CSE1L	SNURF	ELMOD3	UPP1	GPR65	KLRF1	NCF1	LILRA5	IL4R	FPR2	S100A4	EMR4P	ICAM2	DLL4	CXCR1	SERTAD2	MNDA	CLEC10A	CD52	IL23A	PHLDA1
IG	EX	GE	SPC	ENT	IL21	I	LG	N	RD	I	F	SI	H	IL3	DP	S	SIR	M	CL	CD	IL	PL

KC	OC6	MIN6	S3	PD5	R	L5RA	ALS1	CF1B	X	TGAL	RAT2	GLEC8	IVEP2	2	YD	TEAP4	PA	RC1	EC4A	53	DR1	A2G4A
PNOC	IARS	GNLY	CCNA1	FAM119A	KIR2DS1	P2RY14	MAPK7	P2RY10	RRAGD	ITGAM	GPR27	SLC6A4	MARCKSL1	LAMP2	ERBB3	MGAM	TMED2	MS4A6A	CSF1R	CD59	LONRF3	RAB27B
FCRL2	ITK	GPT2	CEACAM8	FAM179A	KIR2DS2	DACH1	KLHL7	SP100	TACSTD2	PARVG	GPR77	PTGS2	MBP	LILRB5	F11R	BTNL8	ENG	PELO	CTSS	CD6	SH2D6	RBMS3
BACH2	KIF11	GZMA	NOS2	CLIC2	KIR2DS5	DAPK2	KRT80	TXNIP	INPP5F	PSAP	RNASE2	EGR3	MMP15	KLRG1	FAM27A	CXCR2	FCAR	PLEK	DMN	CD68	TNIP2	RNF125
CR2	KNTC1	GZMH	SRA1	COX7A2L		EMR3	LAMC1	STAP1	RAB38	PTGER2	MS4A2	PILRA	PNPLA6	HSPA4	FAM49A	TNFRSF10C	IGF1	PRSS23	DPP4	CD7	ABCA1	TMPRSS3
TCL1A	NUF2	GZMK	TNFRSF6B	CREB3L4			LCORL	TAGAP	PLAU	PTGES2	BASP1		TMBIM6	HSPB6	FASLG	VNN3	ITGA2B	PTGIR	LRRC32	CD96	ABCB1	GATA3
AKNA	PRC1	IL2RB	TREM1	CSF1			LMNB1	ZCCHC2	CSF3R	S100A8	IGSF6		PQBP1	ISM2	FCGR1A		GABARAP	ST8SIA4	MC5R	CFHR3	ADAMTS12	BIRC5

ARHGAP25	PSAT1	LCK	TREML1	CSNK2A2			MEIS3P1		SLC18A2	S100A9	HK3		TEX264	ITIH2	FN1		GPX1	STAB1	MICA	CHRM3	ANK1	CDC25C
CCL21	RGS1	MPZL1	RHOA	CSTA			MPL		AMPD2		VNN1		IKZF1	KDM4C	FSTL1		KRT23		NCAM1	CLEC7A	ANKRD22	CDC7
CD27	RTKN2	NKG7	SLC25A37	CSTB			FABP1		CLTB		FES			KIR2DS4	FUCA1		PROK2		NCR2	COL23A1	B3GALT2	CENPF
CD38	SAMSN1	PIK3IP1	TNFSF14	CTPS			FABP5		C1orf162		NPL			KIRREL3	GBP3		RALB		NRP1	COL4A4	CAMTA1	CXCR6
CLEC17A	SELL	PTRH2	TREML4	CTSD			FADD				FZD2			SDCBP	GLS2		RETNLB		PDCD1LG2	COL5A3	CCR9	DHFR
CLEC9A	TRAT1	TIMM13	VNN2	FST			MFAP3L				FAM198B			NFATC2IP	GRB2		RNF141		PDCD6	DAB1	CD40	EVI5
CLECL1		ZAP70	XPO6	GATA2			MINPP1				HNMT			MICB	LST1		SEC14L1		PRDX1	DLEU7	GPR4	GSTA4
			CLEC4C	GMPR			RPS24				SLC15A3			KIR2DL1	BCL2		SEPX1		RAE1	DOC2B	IFT80	HELLS
			TNFAI	HDC			RPS7				CD			KIR2	CD		EMP3		RAET	EMP1		IL26

			P2								4			DL3	C5L				1E			
			UBD	HEY1			RPS9				TXNDC3			KIR3DL1	FGF18		CD300LF		SIGLEC7	F12		LAIR2
			ACTR3	HOXA1			DBNL				FRMD4A			KIR3DL2	FUT5		ABTB1		SIGLEC9	FURIN		
			RAB1A	HS2ST1			CCL13				CRYBB1			NCR1	FZR1		KLHL21		TYRO3	GAB3		
			SLA	HS3ST1							HRH1			FOSL1	GAGE2		PHRF1		CHST12	GATM		
			HLA-DQA2	BCL11B							WNT5B			TSLP	IGFBP5				CLIC3	GFPT2		
			SIGLEC5	CDH3										SLC7A7	KANK2				IVNS1ABP	GPR25		
			SLAMF9	MYL6B										SPP1	LDB3				KIR2DL2	GREM2		
				NAA16										TREM2					LGMN	HAVCR1		
				ClQA										UBASH3A						HSD11B1		

				C1QB										YBX2						HUNK		
				CYP27B1										CCDC88A						IGF2		
				EIF3M										CLEC1A						RCSD1		
														THBD						RYR1		
														PDPN						SAV1		
														VCAM1						SELE		
														EMR1						SELP		
																				SH3KBP1		
																				SIT1		
																				SLC35B3		
																				SIGLEC10		
																				SKAP1		
																				THUMPD2		
																				TIGIT		
																				ZE		

																				B2		
																				EN<br>C1		
																				FA<br>MI<br>34<br>B		
																				FB<br>XO<br>30		
																				FC<br>GR<br>2C		
																				ST<br>AC		
																				LT<br>C4<br>S		
																				M<br>AN<br>1B<br>1		
																				M<br>DH<br>1		
																				M<br>M<br>D		
																				RG<br>S1<br>6		
																				IL1<br>2A		
																				P2<br>RX<br>5		
																				CD<br>97		
																				IT<br>GB<br>4		
																				IC<br>A<br>M3		

																				METRNL		
																				TNFRSF1A		
																				IRF1		
																				HTR2B		
																				CALD1		
																				MOCOS		
																				TRAF3IP2		
																				TLR8		
																				TRAF1		
																				DUSP14		
