# Supplementary material for: Prognostic and therapeutic implication of m6A methylation in Crohn disease
Source: Medicine (Baltimore). 2022 Dec 23;101(51):e32399. doi: 10.1097/MD.0000000000032399 (PMC9794314; doi:10.1097/MD.0000000000032399)
Supplement: Supplementary file 6 [file medi-101-e32399-s006.pdf]

Supplemental Table 6. The expression of immune cells

ID	Activated.B.cell	Activated.CD4.T.cell	Activated.CD8.T.cell	Activated.dendritic.cell	CD56bright.natural.killer.cell	CD56dim.natural.killer.cell	Eosinophil	Gamma.delta.T.cell	Immature.B.cell	Immature.dendritic.cell	MDSC	Macrophage	Mast.cell	Monocyte	Natural.killer.T.cell	Natural.killer.cell	Neutrophil	Plasmacytoid.dendritic.cell	Regulatory.T.cell	T.follicular.helper.cell	Type.1.T.helper.cell	Type.17.T.helper.cell	Type.2.T.helper.cell
GSM5656180_con	0.315436599	0.38876919	0.51771049	0.523383556	0.486128242	0.486658127	0.127248765	0.683046903	0.385715576	0.547120603	0.394012004	0.230494005	0.335002778	0.753682456	0.307096229	0.532924863	0.289983037	0.627657154	0.35350891	0.432120148	0.432443687	0.307537439	0.382934406
GSM5656185_con	0.056831968	0.207388157	0.479791712	0.449347809	0.442599818	0.503808271	0.026897831	0.484693781	0.220684752	0.488164555	0.318399208	0.144796891	0.152989414	0.759094636	0.289090165	0.351618127	0.294260866	0.544393183	0.244966203	0.323539297	0.38237449	0.336294096	0.224752547
GSM5656189_con	0.177644087	0.347257073	0.428350901	0.504094987	0.499803233	0.435597756	0.152024659	0.632753661	0.32503541	0.566079155	0.2870108	0.223625644	0.361256414	0.687449543	0.315586471	0.536247654	0.190961185	0.644867306	0.284067262	0.373387189	0.411529441	0.254254072	0.447065696
GSM5656	0.841576	0.7939627	0.6737490	0.554941044	0.535965129	0.423231104	0.337	0.6800228	0.912861	0.570123469	0.608	0.324	0.438	0.726	0.3729408	0.567730	0.232	0.637142092	0.552883	0.5097862	0.4925139	0.2796382	0.6004804

190_con	174	66	61				652405	9	211		281951	54793	499289	199694	61	308	644599		253	1	74	29	26
GSM5656202_con	0.662516225	0.481363586	0.580095922	0.571787189	0.498758017	0.474726002	0.287090199	0.703279147	0.618617328	0.561364946	0.572811399	0.298980044	0.413190438	0.754393092	0.351605429	0.569394904	0.206698237	0.66838967	0.491076192	0.478383623	0.459842674	0.295674675	0.430016928
GSM5656208_con	0.825863728	0.699822417	0.657299426	0.558828908	0.50856572	0.414414355	0.319956806	0.698563025	0.892673645	0.572792182	0.611634123	0.316679296	0.445118683	0.714328135	0.374061884	0.572734972	0.272263972	0.653181368	0.53751057	0.498989243	0.486737932	0.294879007	0.542858152
GSM5656230_con	0.365938623	0.50061595	0.506791349	0.50189997	0.495407658	0.41132961	0.19723609	0.660888772	0.424949871	0.560281122	0.42221857	0.257114718	0.39143086	0.693555568	0.338814533	0.533305282	0.27011241	0.618448293	0.396896887	0.43147419	0.43419282	0.263787498	0.506419473
GSM5656232_con	0.311295652	0.439220061	0.529078105	0.571827659	0.49730348	0.470739446	0.2049704	0.711256889	0.342897957	0.566897422	0.5584122	0.2831177	0.3907151	0.7503238	0.35540577	0.588741865	0.3077622	0.640034596	0.451751664	0.464491956	0.450438758	0.299344724	0.484648251

							53				62	86		03			41						
GSM5656238_con	0.397894916	0.430395889	0.563744817	0.543140954	0.506081552	0.465964858	0.178608156	0.718747284	0.425272592	0.564726273	0.437011048	0.26851237	0.35611988	0.763905188	0.323526212	0.551797312	0.260746351	0.619277909	0.425515908	0.444676558	0.429868859	0.294005531	0.453067014
GSM5656245_con	0.250130178	0.512114162	0.589858065	0.539827943	0.520625509	0.478002744	0.319772865	0.696376702	0.371125109	0.577130315	0.478198727	0.2540841	0.505009039	0.740317326	0.349536056	0.580296659	0.147524534	0.666920888	0.396065578	0.464695588	0.447461995	0.278966774	0.483704822
GSM5656258_con	0.233394471	0.171498269	0.402449947	0.544496968	0.460313844	0.502261437	0.042393523	0.603585199	0.326271295	0.524497047	0.336111102	0.196501014	0.237755237	0.76422031	0.305078489	0.461972919	0.298988601	0.609674693	0.355930842	0.373820772	0.394304502	0.335812715	0.273732737
GSM5656270_con	0.394826449	0.508285423	0.466826276	0.522654621	0.509818461	0.425558165	0.216792081	0.661297106	0.523726693	0.588630312	0.415641082	0.260519368	0.382936326	0.699691858	0.348997187	0.557044405	0.240845265	0.646731653	0.387832951	0.425001513	0.445793155	0.244762664	0.528853174
GSM	0.29	0.376	0.520	0.5426	0.489591	0.46825	0.1	0.692	0.29	0.5517	0.3	0.2	0.3	0.7	0.285	0.48	0.2	0.62847	0.36	0.420	0.422	0.299	0.398

5656271_con	2632113	209754	066284	81226	835	7724	05419344	259429	2872568	73485	9077393	49410879	16123923	48422399	751574	9608768	5091001	4642	3001626	642781	916732	079908	643907
GSM5656279_con	0.5663595	0.514319796	0.540039244	0.555577138	0.500202063	0.461280074	0.241371915	0.68670011	0.613390149	0.577520432	0.573235612	0.303139286	0.40116087	0.774615861	0.333194277	0.581476071	0.292497666	0.663522711	0.447549868	0.464746227	0.459031598	0.309897917	0.477771234
GSM5656283_con	0.226232864	0.399813258	0.464692721	0.540869646	0.474417002	0.465489215	0.207242595	0.661721841	0.384613529	0.55782816	0.444860874	0.241661666	0.420890134	0.746254157	0.334917421	0.560105154	0.272089497	0.667369522	0.371146883	0.423565542	0.425074428	0.285114321	0.448347042
GSM5656287_con	0.764262955	0.542538037	0.662581511	0.557125507	0.517139353	0.473517505	0.238255912	0.716155449	0.712744171	0.547488515	0.564143927	0.308214509	0.38954458	0.765880006	0.334601161	0.546766253	0.237315401	0.656763786	0.439558443	0.478071612	0.476000501	0.282236643	0.423635296
GSM5656288_	0.759905873	0.628265734	0.715721103	0.575393435	0.545360189	0.469114982	0.25261	0.735808614	0.79585596	0.584118709	0.61377	0.32491	0.44079	0.78693	0.358090779	0.606347686	0.17810	0.659336177	0.528945844	0.528772708	0.493610385	0.31916714	0.496458825

con							7475				6086	1848	2804	3123			8749						
GSM5656292_con	0.416077659	0.377214675	0.503753936	0.528718986	0.525047703	0.466823552	0.172842867	0.725742344	0.475785627	0.562386528	0.476008524	0.294010889	0.376415588	0.750589911	0.334011857	0.575906857	0.310629556	0.642384731	0.409338016	0.450855253	0.442885949	0.320560277	0.425859951
GSM5656299_con	0.631760601	0.541597295	0.612468894	0.543116704	0.538778577	0.459605579	0.22146187	0.728515949	0.583697525	0.566050604	0.543515197	0.316181016	0.418378076	0.760198898	0.326528495	0.56222717	0.312400331	0.630366213	0.482795086	0.48102653	0.460629796	0.295894777	0.44667432
GSM5656300_con	0.394323615	0.588911484	0.441977445	0.505903258	0.49864438	0.425793658	0.264853207	0.666104288	0.428311399	0.602279482	0.435428796	0.245865886	0.400728125	0.682990943	0.351644507	0.547858672	0.234922243	0.644500643	0.404407925	0.406308074	0.416618353	0.282204047	0.602885457
GSM5656304_con	0.226262448	0.348889613	0.467260069	0.558694505	0.471841318	0.469328677	0.194189809	0.681012675	0.280196473	0.55950152	0.463476496	0.281227276	0.391406558	0.771599887	0.31769044	0.578161327	0.287892248	0.629306303	0.383664056	0.440665828	0.430213389	0.278212557	0.433307745

GSM5656310_con	0.358892059	0.455547126	0.526500538	0.527562819	0.503262294	0.47951753	0.154376707	0.713465944	0.364904886	0.561105288	0.460348426	0.268868559	0.351105799	0.749658811	0.302246066	0.543097495	0.291752669	0.630448288	0.377969144	0.440512759	0.43014168	0.329533665	0.443494534
GSM5656313_con	0.013261662	0.17806299	0.492234106	0.472427848	0.454985919	0.551456183	0.038488492	0.518942891	0.234769142	0.487947479	0.309299687	0.167350975	0.108079447	0.789365631	0.29047089	0.355614615	0.313090096	0.574385391	0.230874453	0.343174799	0.35807613	0.337762843	0.192404084
GSM5656314_con	0.518799432	0.620954782	0.521530164	0.517718309	0.49732066	0.416788724	0.306240454	0.657980351	0.62654507	0.58741274	0.490977225	0.267452375	0.408098585	0.675882728	0.347036338	0.543849151	0.251904126	0.625182857	0.456067494	0.443257023	0.451014213	0.254328997	0.585484644
GSM5656539_con	0.276914719	0.476055391	0.535105834	0.500668945	0.47968454	0.450606134	0.254392562	0.669821009	0.311099946	0.564122278	0.467325297	0.278130712	0.343847379	0.724115257	0.33782739	0.572746103	0.288959908	0.652539539	0.426616629	0.408702508	0.42711199	0.288130081	0.502204252
GSM5656	0.577675	0.6657515	0.6410780	0.62041671	0.510075897	0.462421173	0.346	0.7118193	0.522560	0.598018081	0.639	0.344	0.418	0.782	0.3465728	0.624136	0.350	0.70140263	0.496922	0.4786480	0.4718108	0.3401234	0.5711395

171_treat	169	12	6				63537	34	828		86564	739114	781975	16461	1	24	676996		195	69	41	29	49
GSM5656174_treat	0.320123864	0.389487556	0.541994884	0.546624684	0.481717576	0.475404796	0.230689658	0.671341352	0.320538007	0.550557036	0.525663548	0.291215129	0.451401122	0.752270336	0.315202552	0.598285463	0.244588542	0.648853441	0.417339352	0.424655143	0.478049611	0.283430085	0.46062154
GSM5656175_treat	0.683272589	0.764319456	0.699027235	0.739344729	0.577511373	0.530373993	0.379172277	0.737817147	0.751945418	0.693844901	0.970567823	0.587310463	0.642556229	0.771220195	0.526966271	0.649547174	0.583744556	0.787278764	0.762604058	0.60535916	0.579818912	0.384263094	0.578290049
GSM5656177_treat	0.402766236	0.577809415	0.564684503	0.507359399	0.492958062	0.432806958	0.206950167	0.702726641	0.484801151	0.555280951	0.422172434	0.277134398	0.323765836	0.68527791	0.29782154	0.531466107	0.318269523	0.626272781	0.424235198	0.412024519	0.418257498	0.315250229	0.51802048
GSM5656179_treat	0.359709624	0.472077031	0.434627946	0.578317806	0.519442925	0.467135228	0.3826675	0.73568131	0.393663439	0.650976257	0.5896728	0.3590957	0.6417506	0.6928666	0.383622366	0.637567313	0.4214453	0.7044457	0.553453277	0.482709634	0.466265589	0.258831112	0.540913517

							95				96	55	8	9			11						
GSM5656183_treat	0.523106415	0.413026599	0.515322057	0.590027862	0.516998757	0.542972679	0.228017223	0.655783272	0.541335322	0.546201868	0.546169403	0.268832008	0.281135593	0.718880484	0.307802027	0.56676762	0.289755607	0.657861504	0.442309915	0.410119058	0.417748111	0.403363223	0.402975229
GSM5656184_treat	0.215731166	0.550031405	0.502343764	0.539388952	0.483973323	0.425859832	0.283464362	0.684315962	0.306162811	0.569897305	0.401347479	0.243821565	0.39438024	0.701837746	0.338324117	0.583401706	0.230984597	0.631926156	0.370291571	0.421252559	0.417235504	0.272226875	0.591614319
GSM5656186_treat	0.247732658	0.510515503	0.543674369	0.573960246	0.497738564	0.458125925	0.203090719	0.688160772	0.319475966	0.595909716	0.553023322	0.306477719	0.443033055	0.73890025	0.32647365	0.591111768	0.393193804	0.635839781	0.44771865	0.426083653	0.440053604	0.313688436	0.499668483
GSM5656187_treat	0.324722152	0.55440079	0.517626973	0.5631957	0.47953446	0.453989145	0.348554607	0.657227839	0.367024652	0.574500926	0.487374625	0.272478219	0.378333883	0.692154568	0.336658443	0.581539948	0.343334803	0.65140541	0.427406926	0.422217334	0.438481169	0.318016721	0.530643183
GSM	0.52	0.685	0.640	0.5430	0.473577	0.43032	0.3	0.733	0.51	0.5877	0.6	0.4	0.3	0.6	0.321	0.55	0.3	0.66410	0.54	0.451	0.486	0.339	0.604

5656192_treat	674444	920153	274577	45819	015	5468	75275281	538803	4643204	06624	16067165	00094146	52059903	95513579	207827	5047865	21078887	0705	5205632	398538	986427	576882	967123
GSM5656193_treat	0.338703526	0.525059617	0.574572757	0.664902008	0.537669276	0.54618537	0.280253705	0.669651291	0.414820198	0.690058234	0.714768335	0.434073871	0.534979426	0.750468427	0.448747409	0.66429722	0.433297153	0.756776274	0.563356187	0.466845771	0.543433159	0.316195096	0.559061418
GSM5656195_treat	0.270186263	0.370680532	0.482099491	0.515460152	0.495961564	0.470665088	0.195884316	0.627372444	0.242369593	0.561240772	0.373171127	0.212920422	0.424975142	0.762339443	0.298529871	0.529446102	0.257203059	0.652372699	0.276235952	0.382531628	0.40924302	0.263986433	0.405974277
GSM5656199_treat	0.295528438	0.425955333	0.570539213	0.520007938	0.484301006	0.44029575	0.170455869	0.641380327	0.365338323	0.547212804	0.458054234	0.26254166	0.402546719	0.746693466	0.303023407	0.572097247	0.284763744	0.638674908	0.346047802	0.39392877	0.425446574	0.311841792	0.465471057
GSM5656200_t	0.324124409	0.297814209	0.480689557	0.536811748	0.455856677	0.503206543	0.26917	0.602062883	0.384134037	0.545647719	0.42689	0.25000	0.39717	0.75863	0.332036012	0.557625404	0.24017	0.629640758	0.354196815	0.379073484	0.46360062	0.269307689	0.413434942

reat							6482				3417	4169	2796	9683			119						
GSM5656203_treat	0.414068901	0.54257444	0.53082653	0.581636679	0.483466316	0.480619552	0.295027974	0.653300934	0.459937197	0.549808475	0.515557635	0.288210911	0.336872451	0.758595246	0.298119036	0.568112671	0.3233821	0.66393896	0.414031468	0.403019443	0.463860324	0.31462403	0.469080678
GSM5656207_treat	0.202139472	0.310100843	0.514224924	0.528532566	0.486857506	0.495551794	0.091530524	0.631236068	0.332452506	0.532989721	0.372924041	0.243258651	0.30916447	0.772025579	0.310526943	0.543860778	0.324034903	0.645710222	0.367249052	0.406003172	0.442717428	0.274815065	0.353307651
GSM5656209_treat	0.490752894	0.578447702	0.551303591	0.661992195	0.498035948	0.482125926	0.278654289	0.693554499	0.578594304	0.645027021	0.786265689	0.406236249	0.496952042	0.735474903	0.450289707	0.668882615	0.488754557	0.719045286	0.597625465	0.508993452	0.533168097	0.363121192	0.543000159
GSM5656212_treat	0.368487621	0.530614097	0.588670823	0.651266521	0.526577969	0.488752781	0.317055024	0.69083932	0.423396707	0.601066834	0.666798165	0.334015243	0.491325193	0.780825747	0.345957501	0.624438473	0.469422851	0.678060214	0.51874266	0.488118176	0.487590416	0.290934872	0.500779995

GSM5656214_treat	0.387223739	0.384711521	0.553709499	0.560680607	0.498475559	0.456511343	0.213947804	0.646761753	0.395297717	0.564957417	0.451935978	0.268967804	0.423379453	0.7642665	0.333697131	0.616227121	0.24137949	0.670289489	0.365405868	0.450123339	0.46366384	0.246400387	0.473214597
GSM5656217_treat	0.801605915	0.709872906	0.676528402	0.627404927	0.507338307	0.431175161	0.368739866	0.696297059	0.803279949	0.608884441	0.766154327	0.407602018	0.553991338	0.729935134	0.370399631	0.565561579	0.429592263	0.681348	0.595146301	0.515130874	0.481573533	0.34735164	0.554653481
GSM5656220_treat	0.594129621	0.602548179	0.551497863	0.654293465	0.509150102	0.383482696	0.451908065	0.687148392	0.573476939	0.70432457	0.786416562	0.440995985	0.725295425	0.754550167	0.441358153	0.680438369	0.451111777	0.75909746	0.592668451	0.529047924	0.528985744	0.215452324	0.549705236
GSM5656223_treat	0.307064482	0.467461454	0.606534159	0.61518114	0.497910825	0.475072034	0.286308081	0.672605746	0.382462067	0.631292595	0.625281803	0.33932191	0.520185144	0.759841089	0.334079376	0.614787916	0.505480872	0.69896261	0.447981347	0.467226024	0.479524068	0.278924314	0.459695555
GSM5656	0.521233	0.4897443	0.6210661	0.589021511	0.515921284	0.461659498	0.252	0.6903147	0.506028	0.577466512	0.590	0.319	0.434	0.777	0.3350419	0.595113	0.336	0.647035613	0.458381	0.4800124	0.4862843	0.2890949	0.4707338

226_treat	982	48	78				911994	4	131		160342	947714	88188	155903	32	281	876264		535	52	04	17	1
GSM5656228_treat	0.358322685	0.615811814	0.577557207	0.587481268	0.502928679	0.442608404	0.294780676	0.689413857	0.442111938	0.580863657	0.633429891	0.309710458	0.438938263	0.691972142	0.356383961	0.577759943	0.399653954	0.651408001	0.516587952	0.431146852	0.456147794	0.32054907	0.58442636
GSM5656231_treat	0.580511138	0.43041565	0.527681501	0.555248799	0.4718311	0.488726704	0.295259991	0.597266269	0.527807779	0.536827848	0.53784799	0.208563803	0.285386803	0.705492102	0.281773315	0.538640558	0.203961532	0.673013896	0.431026545	0.36580273	0.450268205	0.335540087	0.392053831
GSM5656235_treat	0.282988882	0.504020562	0.593332347	0.575625675	0.506887408	0.483476391	0.22221663	0.639725198	0.331143279	0.550819891	0.486437019	0.236261093	0.348387514	0.762867037	0.307635419	0.564600387	0.232717978	0.678124189	0.393346737	0.392601418	0.443960099	0.277853521	0.490475117
GSM5656237_treat	0.300770552	0.600084494	0.558107139	0.631880043	0.505401064	0.460267557	0.3397239	0.695729167	0.404711221	0.621324245	0.6344810	0.3545549	0.4458881	0.7611806	0.367761019	0.608993232	0.4053332	0.691334434	0.509041879	0.480825729	0.478906498	0.311326613	0.534648044

							55				47	45	38	58			89						
GSM5656240_treat	0.230981619	0.573447964	0.546660129	0.554903007	0.45669534	0.470291352	0.177317377	0.686411764	0.324758232	0.586988192	0.499428969	0.285226168	0.410798045	0.721660341	0.349214927	0.536574483	0.311212067	0.640262454	0.455525585	0.385217308	0.457310399	0.271511504	0.530308475
GSM5656243_treat	0.522810271	0.486441733	0.539444176	0.585719516	0.513500674	0.447096153	0.229920602	0.655524639	0.526648337	0.574016232	0.553803094	0.30113907	0.395756914	0.738740679	0.346728989	0.618018457	0.335726242	0.643963591	0.430869925	0.459044518	0.478797886	0.260354516	0.481554745
GSM5656246_treat	0.365754559	0.630471644	0.514852316	0.624823717	0.504502858	0.47957146	0.359243092	0.727344518	0.491090512	0.652817161	0.682734141	0.391550776	0.488788843	0.720208199	0.377381534	0.598781051	0.484639177	0.697725926	0.568696638	0.455432925	0.457865661	0.328216149	0.576673981
GSM5656249_treat	0.267056636	0.401454023	0.537597629	0.546246507	0.473481104	0.471849587	0.151340799	0.656439241	0.312186432	0.535906458	0.427104633	0.248501305	0.369336902	0.742150567	0.306768612	0.54620290	0.291724163	0.641988342	0.337420846	0.387102355	0.44668979	0.278913381	0.390534799
GSM	0.49	0.613	0.543	0.5905	0.516846	0.50352	0.1	0.650	0.45	0.5661	0.5	0.2	0.4	0.7	0.323	0.53	0.4	0.64000	0.44	0.393	0.431	0.343	0.512

5656254_treat	0035014	932335	426215	35575	506	1909	77971031	695101	2215709	77462	62915547	82927605	13066452	64822566	72881	6610176	07597522	6909	3953557	203325	428652	325514	203918
GSM5656255_treat	0.446733468	0.531331873	0.512047419	0.707987385	0.521003014	0.364773874	0.609557076	0.682957251	0.597434812	0.706791197	0.824216589	0.498273779	0.803044863	0.718686799	0.44589829	0.667235993	0.762273842	0.775222083	0.612543009	0.551920205	0.549935796	0.301243937	0.548638959
GSM5656259_treat	0.280869451	0.491693719	0.411515346	0.55796183	0.523939477	0.476485276	0.251340172	0.65052196	0.323416637	0.638780284	0.459011	0.286730192	0.376213532	0.739588987	0.36285597	0.612390597	0.285907878	0.707801657	0.423321437	0.45314725	0.444761568	0.294086692	0.522326556
GSM5656262_treat	0.292731328	0.505253347	0.569159774	0.573529423	0.48767465	0.486081575	0.280161499	0.666622386	0.313963273	0.599091017	0.57785678	0.325605034	0.45125511	0.727101335	0.37776674	0.618476893	0.338189064	0.675088231	0.486848033	0.393827846	0.455305269	0.316139068	0.524920484
GSM5656264_t	0.262628288	0.472406413	0.555532808	0.55838664	0.438517334	0.450118408	0.19060	0.640486656	0.430271214	0.522518094	0.53347	0.28588	0.33008	0.73075	0.314129244	0.493189769	0.26850	0.629639334	0.41805881	0.397017544	0.429579599	0.33967316	0.394242801

reat							2308				8651	2271	0032	5513			1831						
GSM5656265_treat	0.820322089	0.751750573	0.662650476	0.577303873	0.505407062	0.421464341	0.341401426	0.690327334	0.868102276	0.556711434	0.62111851	0.318040379	0.390234841	0.727710106	0.354110545	0.558094111	0.246224732	0.6792026	0.561070848	0.48639255	0.477421055	0.324190505	0.588541199
GSM5656267_treat	0.418597316	0.581563068	0.550182146	0.676824449	0.523042689	0.487243179	0.280175914	0.709772671	0.470838885	0.650402929	0.697980158	0.383375284	0.524694781	0.790388246	0.369908888	0.628104665	0.555634625	0.730027561	0.530122906	0.478004007	0.487241226	0.362314596	0.529606
GSM5656273_treat	0.991576586	0.766861527	0.683575518	0.622561232	0.551131291	0.467331827	0.391751928	0.58959089	0.930919878	0.601144531	0.787397233	0.345158781	0.328210763	0.782574069	0.406868081	0.607099574	0.029033896	0.686923851	0.562209311	0.510927058	0.499750171	0.254571284	0.522834559
GSM5656274_treat	0.349173096	0.609426223	0.628048525	0.639994691	0.54412129	0.475131717	0.245096543	0.723429361	0.460593071	0.645992158	0.632130762	0.374154571	0.388907939	0.742223061	0.387138051	0.625882732	0.400507079	0.702560206	0.51804118	0.44454253	0.486921407	0.350529878	0.609332568

GSM5656277_treat	0.486159255	0.677862168	0.673295789	0.65024108	0.540496348	0.445547956	0.50399621	0.748825695	0.527986581	0.643047585	0.692967683	0.373728546	0.600811792	0.742965264	0.362274989	0.658573261	0.292606682	0.694684569	0.540168914	0.508920934	0.523270258	0.297702483	0.665293507
GSM5656280_treat	0.353437547	0.562676356	0.59438367	0.555874291	0.52299358	0.404733691	0.432074593	0.70724759	0.410372798	0.642725983	0.579425765	0.331157562	0.521998421	0.660075812	0.347292986	0.644945268	0.311828327	0.683118981	0.51471569	0.501876612	0.479535739	0.277874015	0.621499655
GSM5656282_treat	0.230815208	0.44218753	0.49728755	0.584349666	0.480626239	0.491901092	0.279482552	0.638349087	0.405612949	0.521216435	0.514163422	0.294226935	0.357569912	0.778960572	0.340416301	0.509515065	0.355987765	0.640495418	0.43754312	0.371457595	0.427531306	0.305966956	0.412821343
GSM5656285_treat	0.311894416	0.500719452	0.58669373	0.645352306	0.521327666	0.494006191	0.239971174	0.677796969	0.332259495	0.596601864	0.604334249	0.308216063	0.429819903	0.798199336	0.332137256	0.604642528	0.415862565	0.660797482	0.453366012	0.428898906	0.468185988	0.324221284	0.491331641
GSM5656	0.408345	0.5878061	0.5832972	0.596992687	0.481733321	0.462493259	0.333	0.6803081	0.522362	0.594308763	0.616	0.353	0.475	0.728	0.3589114	0.542575	0.367	0.650900244	0.508442	0.4460225	0.4607119	0.3132212	0.4929070

291_treat	428	73	38				844614	18	807		851751	154691	973099	200744	03	219	872757		861	18	06	68	29
GSM5656294_treat	0.537494571	0.724608271	0.732948991	0.694596989	0.513424304	0.448538597	0.312435806	0.748375183	0.572532719	0.662943372	0.808251652	0.445321389	0.536050928	0.763737853	0.386637175	0.62686634	0.542555962	0.701865118	0.609017881	0.535825985	0.518665988	0.367316303	0.597037922
GSM5656297_treat	0.279832179	0.637220488	0.595187677	0.572901484	0.505481685	0.450537747	0.253221515	0.684941226	0.388959359	0.568445668	0.626887188	0.32901965	0.464184186	0.727443192	0.334621238	0.548754796	0.364208285	0.659124416	0.5162032	0.449202031	0.451886038	0.328986262	0.524954159
GSM5656303_treat	0.275624832	0.359686473	0.546812982	0.517756113	0.484806239	0.438903686	0.2295322	0.643390948	0.235272367	0.53680507	0.394474156	0.195225901	0.385000522	0.75739878	0.323893237	0.573179173	0.277266359	0.629078215	0.302439272	0.362566043	0.441383554	0.28842081	0.430135481
GSM5656305_treat	0.183276756	0.252967837	0.518673687	0.502992993	0.468783328	0.497322513	0.1550062	0.583536387	0.239080039	0.517375505	0.3490860	0.1743033	0.2371868	0.7543572	0.280236105	0.532430561	0.2715376	0.620359493	0.258584619	0.347566802	0.406067593	0.266375122	0.318014021

							86				93	28	16	07			44						
GSM5656309_treat	0.531260174	0.517492278	0.518973681	0.565796465	0.523429408	0.440385669	0.330719538	0.666617912	0.469516011	0.58892043	0.528559165	0.321140398	0.555949241	0.703626079	0.361196067	0.61501903	0.29028148	0.693572467	0.480387271	0.462604928	0.457323254	0.281135951	0.577000859
GSM5656311_treat	0.236120292	0.426131017	0.606618222	0.5940537	0.497327257	0.519147272	0.122639776	0.660577793	0.341536722	0.556514663	0.533191821	0.313323635	0.336415932	0.7996049	0.353086677	0.554213852	0.391897517	0.650846207	0.445773347	0.40254698	0.451097608	0.323654561	0.397549859
GSM5656316_treat	0.526399264	0.690597318	0.749305513	0.686792582	0.536370667	0.445777797	0.354559758	0.7534587	0.558819143	0.697696662	0.825028116	0.470138322	0.576618741	0.754788372	0.443133546	0.694805286	0.543657028	0.782354644	0.661786999	0.527466545	0.557960126	0.314371487	0.628619658
GSM5656318_treat	0.553293696	0.63350596	0.598196782	0.598379055	0.508335403	0.445972669	0.321718542	0.732678306	0.496782375	0.644605355	0.647796532	0.374563041	0.472725367	0.717489051	0.381396403	0.640224366	0.43795519	0.72371826	0.506651196	0.456590955	0.489381152	0.343577312	0.58398741
GSM	0.32	0.507	0.591	0.6429	0.507073	0.44728	0.3	0.699	0.45	0.6841	0.7	0.4	0.7	0.7	0.385	0.67	0.4	0.73734	0.59	0.505	0.506	0.268	0.537

5656321_treat	2800517	223991	633643	80928	177	6575	79492973	033842	2731007	53821	20801547	03939623	09360497	24987861	81892	4283783	88620186	4858	7769019	557899	48463	471138	119607
GSM5656323_treat	0.587260165	0.619396467	0.667049027	0.653317095	0.533574773	0.40156571	0.367027155	0.718976882	0.575783735	0.678542064	0.805170013	0.398148551	0.608182556	0.731273186	0.411777193	0.701842511	0.316482991	0.748720467	0.618710582	0.531265492	0.539049225	0.282073066	0.592761734
GSM5656325_treat	0.558084595	0.608748511	0.656714757	0.604842715	0.539212229	0.467573775	0.272027709	0.720611511	0.566143659	0.640780736	0.689103977	0.34982227	0.585617304	0.776971142	0.377208161	0.661937651	0.300849714	0.675932046	0.562740707	0.516868383	0.504408929	0.275488782	0.565545953
GSM5656327_treat	0.856479619	0.640553765	0.696716514	0.571356086	0.520539386	0.460045348	0.379078759	0.596816731	0.777073491	0.621537847	0.809160676	0.3563329	0.582693105	0.749205796	0.438003633	0.637639205	0.143311039	0.729569849	0.588609727	0.511084425	0.516312449	0.21203601	0.550388899
GSM5656329_t	0.470565163	0.532888381	0.587347136	0.565957997	0.508261107	0.460504285	0.37081	0.674964629	0.521885104	0.588891714	0.62703	0.28498	0.43916	0.71661	0.332377971	0.618438993	0.15895	0.675896659	0.496618912	0.459974902	0.448793166	0.35420366	0.500917188

reat							8794				3347	7468	2017	7937			4492						
GSM5656334_treat	0.469956228	0.527148332	0.505577292	0.642777969	0.528124859	0.511796987	0.164017737	0.678274783	0.495084786	0.683135898	0.731420817	0.403393439	0.389775942	0.719176013	0.420051327	0.642845997	0.243813587	0.758125331	0.520384995	0.488527396	0.523096828	0.356837036	0.521878767
GSM5656335_treat	0.723927606	0.617637005	0.568279123	0.578154403	0.510588447	0.420172694	0.297619485	0.711108555	0.715550885	0.651128897	0.687769367	0.386203971	0.475097362	0.712233366	0.410668168	0.635491435	0.322095159	0.720063562	0.586092287	0.510579793	0.503305552	0.319608252	0.578960889
GSM5656338_treat	0.316505055	0.578331019	0.650166928	0.64255308	0.515700535	0.503485082	0.240868711	0.651327659	0.401471413	0.63512896	0.66091125	0.345485907	0.41383976	0.796586373	0.317897739	0.60213357	0.56234966	0.693573447	0.440602879	0.414038888	0.492186757	0.357490347	0.52695256
GSM5656342_treat	0.805992401	0.764666154	0.722730629	0.664911809	0.532609368	0.449438765	0.34691025	0.723513622	0.761712302	0.679600096	0.869081205	0.412627407	0.572660647	0.755198079	0.441644003	0.683285752	0.29148195	0.766323073	0.649956776	0.569435071	0.555749567	0.330013166	0.593959312

GSM5656346_treat	0.480613939	0.406822014	0.573235139	0.566651781	0.4922039	0.422031946	0.248032462	0.63648351	0.53711819	0.645723832	0.575700958	0.315157966	0.529044608	0.715872943	0.389079333	0.624335203	0.19069555	0.693513243	0.497220067	0.482668283	0.451250139	0.2461836	0.50056766
GSM5656348_treat	0.304090679	0.490012558	0.529269988	0.537251083	0.49875701	0.449807085	0.185262574	0.67179556	0.30174241	0.575876273	0.448011107	0.249001723	0.439586111	0.711597739	0.309226509	0.596064979	0.227527034	0.637645363	0.338303072	0.416207602	0.43912731	0.302549652	0.522280498
GSM5656351_treat	0.300659863	0.526525104	0.592372027	0.536441281	0.508662525	0.428189589	0.279724704	0.690134328	0.400938638	0.567531412	0.49716034	0.305308454	0.409122443	0.715650475	0.331696589	0.582957228	0.284421458	0.654616657	0.429236506	0.411455756	0.474578705	0.338793155	0.545962644
GSM5656353_treat	0.443474856	0.586746654	0.611703197	0.550282144	0.506021145	0.439297775	0.291754175	0.659320068	0.499106175	0.605538234	0.517599773	0.280882485	0.466092338	0.721656277	0.354884981	0.630575202	0.2516489	0.678869758	0.428363557	0.458466541	0.460476687	0.281253323	0.574076334
GSM5656	0.700599	0.6804507	0.6828474	0.670406196	0.522000008	0.442232673	0.372	0.7314310	0.600829	0.657701965	0.874	0.483	0.561	0.755	0.4428691	0.662817	0.496	0.782328726	0.670577	0.5537054	0.5666623	0.3436189	0.5180121

355_treat	632	88	01				554554	24	055		167596	304685	257476	236367	15	25	288231		974	15	5	94	33
GSM5656359_treat	0.534939664	0.547519591	0.663554065	0.667187376	0.549962359	0.393699814	0.556567343	0.714708221	0.671903142	0.666811245	0.774930346	0.487545889	0.758330745	0.72028688	0.426076534	0.650874948	0.646854696	0.783720628	0.57692317	0.584961471	0.527903308	0.289322359	0.582214033
GSM5656361_treat	0.645476894	0.711601661	0.763744648	0.744677311	0.549548319	0.462860613	0.555189281	0.742518033	0.653772339	0.682432896	0.935617648	0.486685834	0.714240292	0.768106401	0.454532211	0.719644808	0.538399439	0.784376224	0.674486139	0.600341277	0.594585416	0.372307705	0.593303378
GSM5656365_treat	0.518976508	0.56557775	0.656372906	0.605820915	0.520508985	0.426681302	0.362983308	0.717221946	0.523084408	0.626133836	0.642601834	0.361485119	0.472347898	0.748497723	0.378130285	0.659305474	0.368278363	0.726526448	0.54495577	0.484218623	0.468562235	0.310689403	0.541223055
GSM5656366_treat	0.616238856	0.684472486	0.741120751	0.688718741	0.544766579	0.435531063	0.4286665	0.682476669	0.672380361	0.682458326	0.8755535	0.4429096	0.6739075	0.7537794	0.451128837	0.703956982	0.3434618	0.754540653	0.71341718	0.578410583	0.577921845	0.312487107	0.621840746

							04				43	6	98	46			83						
GSM5656368_treat	0.701090698	0.547427637	0.680844699	0.620832152	0.501368664	0.446075927	0.245813944	0.633415783	0.760420615	0.646792379	0.787783309	0.355272597	0.488650238	0.779314917	0.410908916	0.658826848	0.312413904	0.720867903	0.610336697	0.537958776	0.536361009	0.283933299	0.516000419
GSM5656372_treat	0.890005174	0.64822384	0.701858625	0.55965505	0.544526575	0.419011481	0.310535188	0.610202204	0.925964016	0.62165434	0.715417592	0.314984778	0.447620272	0.72400171	0.401863409	0.625436193	0.064558626	0.711370462	0.561968406	0.551583738	0.517425243	0.273259029	0.535368407
GSM5656374_treat	0.328856265	0.481244548	0.587448052	0.569544934	0.520668948	0.430511353	0.216411719	0.719795526	0.448880883	0.629870117	0.485427666	0.313115165	0.461658475	0.698579103	0.339425804	0.595457351	0.234508537	0.654145178	0.446851146	0.464399513	0.449516048	0.284502069	0.534214555
GSM5656375_treat	0.269918104	0.241117858	0.440706163	0.507561063	0.480548828	0.417882172	0.33129404	0.572502955	0.391195878	0.678190584	0.542283742	0.321019316	0.614869189	0.619910634	0.333754429	0.55835655	0.229143353	0.681977376	0.461857025	0.40746221	0.439809834	0.164095347	0.450214727
GSM	0.62	0.738	0.694	0.6954	0.510350	0.46185	0.4	0.697	0.71	0.6768	0.8	0.4	0.5	0.7	0.443	0.67	0.5	0.78501	0.62	0.509	0.564	0.361	0.616

5656380_treat	7703984	436813	307868	14531	776	4364	48174996	560384	9983136	92811	4380634	56362022	91609288	43903041	72288	6454415	2226861	3936	7962768	831872	471782	43044	36596
GSM5656381_treat	0.510672553	0.592979656	0.577043526	0.632759208	0.480310237	0.486980138	0.20527427	0.6589076	0.545768484	0.633656965	0.643466617	0.336900392	0.380566164	0.77724484	0.35745174	0.585400435	0.372401979	0.704127147	0.444937446	0.452228284	0.459453399	0.333946506	0.52335995
GSM5656384_treat	0.520123497	0.559755231	0.69174796	0.620814006	0.531005654	0.484499969	0.341074649	0.639894343	0.610791183	0.639560604	0.648399026	0.343339186	0.547628323	0.804992156	0.358539812	0.648960868	0.268105036	0.691561858	0.457213124	0.506850362	0.488570038	0.255524001	0.539300062
GSM5656387_treat	0.196300588	0.315412865	0.579585146	0.562084244	0.51951604	0.473889511	0.167618879	0.661375788	0.372925999	0.576149054	0.49767154	0.260068571	0.394183136	0.796858865	0.333839009	0.512260697	0.253321743	0.641587516	0.396075063	0.436404261	0.450974063	0.286984195	0.366112859
GSM5656388_t	0.205759825	0.453836508	0.421086741	0.586937213	0.505591814	0.510809505	0.21746	0.615589682	0.295048383	0.564104977	0.50610	0.30526	0.34790	0.77155	0.330878221	0.51618166	0.38877	0.657260651	0.475916134	0.378709664	0.418435606	0.345611617	0.4438387

reat							4999				6149	229	6634	1427			7963						
GSM5656390_treat	0.188416969	0.506523193	0.527436223	0.530016475	0.462195635	0.494021045	0.207975961	0.626655064	0.427142721	0.542667614	0.475564568	0.287482063	0.247337214	0.688107546	0.307386536	0.465987836	0.326154204	0.597137842	0.415203373	0.387208733	0.435606898	0.387151023	0.466407231
GSM5656392_treat	0.649784448	0.553946349	0.548654816	0.589142599	0.522056664	0.481782035	0.294944034	0.621586291	0.670572468	0.666804269	0.671709657	0.33815103	0.468291507	0.751176091	0.369536387	0.655831738	0.229194463	0.719114815	0.496876851	0.498190428	0.486808431	0.293091287	0.580896761
GSM5656393_treat	0.629277322	0.685352969	0.647598772	0.764446506	0.542222986	0.443389607	0.631578274	0.706510812	0.847376496	0.743492641	0.981897923	0.589659929	0.645508461	0.801750701	0.521069648	0.647376765	0.9784507	0.843697818	0.729683537	0.590006956	0.597729826	0.391017306	0.525584924
GSM5656395_treat	0.348984277	0.537854397	0.50043021	0.590474435	0.489432609	0.548592854	0.195029481	0.685897292	0.409575092	0.625391393	0.609563579	0.354205803	0.38322883	0.693478591	0.432577848	0.577510714	0.421348302	0.709504678	0.512414125	0.461520712	0.461890666	0.380596401	0.56607825

GSM5656399_treat	0.414942209	0.423173796	0.707914617	0.702680781	0.572535241	0.410004727	0.438002914	0.665812988	0.580550548	0.690974275	0.827535104	0.456736531	0.648594137	0.747453384	0.398932633	0.626235234	0.637653502	0.790192777	0.563764853	0.545795084	0.511750124	0.26907846	0.450243618
GSM5656400_treat	0.286935895	0.592012037	0.475448209	0.577217169	0.487770798	0.440049345	0.251856527	0.717990663	0.432245946	0.60653833	0.54552892	0.292872171	0.386804566	0.706737963	0.335445865	0.589294195	0.352593582	0.663261159	0.433485148	0.398117691	0.439913623	0.270286858	0.583691929
GSM5656403_treat	0.446149218	0.639905847	0.548756536	0.57335102	0.477198829	0.464150109	0.179127676	0.678863935	0.457739322	0.572516236	0.550976624	0.29411762	0.332381024	0.72179712	0.317240615	0.550657567	0.299962665	0.662935044	0.40644706	0.420142161	0.445720097	0.328011188	0.544616505
GSM5656406_treat	0.857988628	0.751425002	0.775966907	0.645022245	0.530554762	0.477348509	0.330387209	0.654570133	0.830189257	0.647155543	0.825818764	0.38203856	0.46247918	0.708936819	0.441455899	0.641378442	0.245476582	0.703497579	0.615639225	0.518766589	0.530982653	0.30594023	0.606204965
GSM5656	0.424745	0.6326186	0.5416912	0.542436102	0.489376394	0.428529925	0.411	0.6783072	0.457190	0.599697728	0.508	0.310	0.451	0.676	0.3478770	0.589756	0.266	0.658531439	0.417343	0.4427366	0.4543800	0.3195307	0.6266341

407_treat	245	58	38				068477	9	807		487596	168408	643168	644087	33	5	588271		334	96	11	61	25
GSM5656409_treat	0.615715291	0.680924067	0.654498806	0.711146791	0.538962149	0.500003542	0.340380524	0.702066612	0.562845568	0.689058999	0.803342766	0.462974631	0.469117891	0.770197642	0.419099657	0.642160593	0.604368538	0.790513205	0.581021279	0.48377277	0.541641722	0.366305294	0.612332159
GSM5656412_treat	0.398413364	0.702101978	0.722921653	0.801242948	0.569183232	0.452888203	0.511777021	0.70964784	0.575366557	0.748679387	1	0.583589523	0.611126949	0.797776884	0.53014517	0.708742167	0.763167614	0.815432085	0.752308392	0.651623175	0.611906767	0.428030875	0.571591888
GSM5656416_treat	0.200161617	0.40999781	0.493265949	0.49439457	0.482622146	0.464247491	0.147487309	0.62041032	0.27486573	0.569906274	0.375077176	0.239975246	0.320356049	0.746744702	0.303502464	0.549573087	0.252202159	0.647175305	0.284092895	0.400388781	0.420032227	0.293901089	0.39806852
GSM5656418_treat	0.209841939	0.63574342	0.571048271	0.602227107	0.524753297	0.467984425	0.2598291	0.710219838	0.356531316	0.617885475	0.5828058	0.3199201	0.4326365	0.6874508	0.322275718	0.558217831	0.4529732	0.664707627	0.471457692	0.398017387	0.468928201	0.355106883	0.638972092

							78				75	72	58	4			48						
GSM5656421_treat	0.310165809	0.284564146	0.533348357	0.619824894	0.542743781	0.384942943	0.316672283	0.666391386	0.440220091	0.670366536	0.682268419	0.419213573	0.610901356	0.682105116	0.389889682	0.579628143	0.444029559	0.747781653	0.545811039	0.532648487	0.469515842	0.256835651	0.471009143
GSM5656424_treat	0.41291146	0.566318847	0.633123389	0.648239136	0.508634057	0.488875106	0.348743526	0.697902117	0.546895885	0.627040809	0.717019274	0.372519222	0.552410337	0.777477808	0.355277143	0.625592813	0.469501385	0.714760918	0.523161291	0.530027438	0.514181039	0.285060099	0.506738064
GSM5656426_treat	0.603098334	0.619748732	0.580815597	0.574680663	0.512511227	0.503902047	0.303460168	0.684280021	0.659031896	0.5853003	0.576922308	0.269066428	0.308889009	0.743802322	0.308420346	0.514675488	0.191629223	0.646569334	0.476597954	0.399735972	0.45134966	0.353628925	0.466156099
GSM5656429_treat	0.464568286	0.52367216	0.65089845	0.577362804	0.494812201	0.456067943	0.280198217	0.699054828	0.470191992	0.599270369	0.568713664	0.30394933	0.425640238	0.748190533	0.344606758	0.64135324	0.255011221	0.681902959	0.463132282	0.455416998	0.477775767	0.293522756	0.509801327
GSM	0.34	0.606	0.682	0.6644	0.516509	0.50594	0.3	0.687	0.57	0.7401	0.7	0.4	0.6	0.7	0.449	0.55	0.6	0.67982	0.54	0.545	0.533	0.440	0.658

5656431_treat	1618601	552239	240546	87405	694	3147	80184489	925613	0148116	17914	81330063	70488438	65030301	33246288	490799	8076188	53309818	7954	9040181	204753	118342	109619	634503
GSM5656432_treat	0.210423769	0.575287698	0.476061275	0.681707326	0.474523569	0.504781396	0.293367993	0.641194986	0.387456974	0.616120976	0.677059785	0.373621606	0.507192055	0.760297676	0.353733346	0.560180131	0.635058989	0.695802771	0.43635088	0.436226623	0.460332826	0.389517814	0.458457848
GSM5656434_treat	0.409325516	0.547454303	0.663341286	0.653737791	0.562404715	0.431887517	0.345337653	0.760278561	0.512628153	0.686108502	0.736598917	0.419906774	0.553300996	0.768272736	0.422126456	0.692941443	0.267738966	0.74115365	0.633634832	0.544658172	0.493106106	0.301731402	0.58502076
GSM5656435_treat	0.447286117	0.464947952	0.499573962	0.572730688	0.497594259	0.443438583	0.271283633	0.659219812	0.551013605	0.594622986	0.594486517	0.323696935	0.466761747	0.739553524	0.336111961	0.567506713	0.320205387	0.633745758	0.490844778	0.467215451	0.461989485	0.268573384	0.397055404
GSM5656439_t	0.349057202	0.591509104	0.570321886	0.625613247	0.485462543	0.474938437	0.20310	0.698975373	0.409120365	0.609921035	0.60823	0.32366	0.44286	0.73855	0.355314785	0.583563429	0.38799	0.681395104	0.475521274	0.431499231	0.456982346	0.35991109	0.550590169

reat							9932				6181	6052	3592	8111			0555						
GSM5656443_treat	0.632305775	0.629413475	0.689539365	0.66526395	0.535414893	0.386622663	0.463256505	0.753972886	0.74757213	0.720392641	0.820749316	0.460345844	0.653855968	0.689227423	0.455806153	0.65993927	0.465203993	0.750737952	0.717893584	0.558585956	0.584334648	0.300146203	0.628396261
GSM5656444_treat	0.699406166	0.69986508	0.657956524	0.561785255	0.48551585	0.470221252	0.389192716	0.653311114	0.690793394	0.615110116	0.634729425	0.280184414	0.371147081	0.682783411	0.333199171	0.579497888	0.025472052	0.641890771	0.45656824	0.456311497	0.462017095	0.330896619	0.542507907
GSM5656447_treat	0.235353727	0.475122008	0.584694064	0.555830842	0.477341836	0.455508601	0.114695104	0.662487634	0.376415302	0.53966394	0.513810906	0.294807054	0.321530695	0.740101026	0.317331466	0.528525619	0.320590557	0.640989419	0.405553502	0.381425478	0.432712671	0.332891335	0.442223161
GSM5656451_treat	0.584255042	0.60660097	0.686773431	0.543482073	0.528533198	0.444645544	0.383757849	0.63638972	0.522237721	0.594765871	0.597886929	0.287892221	0.421265464	0.711896948	0.318154694	0.661793527	0.186284406	0.696705077	0.413661297	0.431786631	0.472778995	0.337984301	0.558875046

GSM5656452_treat	0.4894030552	0.586222206	0.598195011	0.670650582	0.532093269	0.476959571	0.430856056	0.740564596	0.575608249	0.651482478	0.724935069	0.399069104	0.536626412	0.759526962	0.370253674	0.621028805	0.533269692	0.682476599	0.554338404	0.4919984	0.519583677	0.371955692	0.521737245
GSM5656454_treat	0.91683788	0.776431868	0.737364468	0.567087074	0.541458408	0.408264962	0.320739909	0.628327807	0.884364053	0.564356884	0.64948874	0.294873877	0.353193875	0.747059902	0.35300344	0.612673842	0.111677608	0.670877482	0.54659271	0.484036777	0.487576807	0.26275334	0.58977475
GSM5656458_treat	0.218067003	0.364910218	0.516826927	0.560915949	0.467701408	0.47936624	0.16416937	0.664036855	0.300376846	0.547628461	0.419418609	0.257916303	0.340331876	0.774398185	0.304933272	0.557038225	0.247521135	0.643588911	0.403050997	0.400505612	0.453704257	0.291028095	0.393851923
GSM5656460_treat	0.248923899	0.424150618	0.423793502	0.557701325	0.48279907	0.472499767	0.099009374	0.671862159	0.285503929	0.58629369	0.445954474	0.264157572	0.379467467	0.752335752	0.315144525	0.603550782	0.299825683	0.649014344	0.439304668	0.4364051	0.413175771	0.285436878	0.465697805
GSM5656	0.335654	0.5669315	0.5683878	0.560846756	0.502860319	0.431484732	0.295	0.7016662	0.457078	0.586402292	0.580	0.321	0.500	0.698	0.3257567	0.591721	0.376	0.661580534	0.481346	0.4233387	0.4563965	0.3089089	0.5593772

461_treat	454	22	32				151242	7	813		514878	491951	754359	656043	44	808	834087		174	82	9	17	67
GSM5656464_treat	0.262559031	0.393650119	0.621295702	0.586375859	0.504557262	0.488429198	0.227679875	0.644151312	0.369320035	0.573717464	0.537208418	0.271711098	0.345842568	0.784276667	0.315352535	0.593336205	0.401107193	0.648985911	0.46393537	0.419651535	0.468955756	0.306465652	0.450739016
GSM5656465_treat	0.342483001	0.584028787	0.566555948	0.598601238	0.47937088	0.459390308	0.252409741	0.715335306	0.370243675	0.634062379	0.616831912	0.356200746	0.516280823	0.729413792	0.368369542	0.582165181	0.353858929	0.648452728	0.526964206	0.446497706	0.463498751	0.292578266	0.561140683
GSM5656467_treat	0.297078713	0.35056028	0.57958095	0.656833908	0.566998563	0.396451584	0.429548003	0.732167149	0.475907882	0.691072623	0.740256596	0.443123248	0.729055596	0.699695012	0.417172806	0.598960158	0.42575475	0.742736741	0.599413159	0.552879892	0.48620452	0.228723386	0.498740175
GSM5656469_treat	0.242937098	0.367223523	0.528181385	0.527163832	0.484023995	0.466288538	0.2673086	0.61057035	0.391277867	0.567784001	0.4381710	0.2695172	0.4307923	0.7192168	0.308442614	0.595347378	0.2663486	0.633214851	0.381560847	0.405884112	0.456902854	0.278234544	0.467009241

							7				18	37	28	3			3						
GSM5656473_treat	0.41620857	0.65619553	0.617627077	0.548834989	0.480249154	0.439776208	0.178333632	0.642826762	0.410791459	0.590421925	0.533363825	0.278514847	0.302765723	0.695708646	0.317892222	0.565033091	0.224435238	0.667253637	0.327592879	0.408905825	0.42350615	0.37280681	0.580971455
GSM5656478_treat	0.414410951	0.450308718	0.602353236	0.566251756	0.512585059	0.507444093	0.238934503	0.64872515	0.446124825	0.582504399	0.558063239	0.330833797	0.401453265	0.757893004	0.369922342	0.570455342	0.391921561	0.642670541	0.492489984	0.423360649	0.459257691	0.337581782	0.544697305
GSM5656481_treat	0.557559875	0.625396518	0.53226229	0.60138862	0.515502206	0.466811853	0.381871114	0.714016915	0.509202304	0.629619978	0.636346382	0.33058932	0.440314193	0.741792101	0.324030909	0.598773696	0.355565598	0.679928129	0.541430698	0.461713847	0.444381345	0.326888898	0.566527195
GSM5656483_treat	0.329713013	0.567386973	0.62069906	0.565540181	0.523911398	0.446626826	0.344643421	0.6589955	0.381001924	0.5766279	0.566355386	0.279158544	0.410737862	0.714983904	0.306723101	0.580012458	0.431022262	0.651158554	0.441321908	0.407693384	0.459337773	0.355907395	0.575392292
GSM	0.48	0.671	0.590	0.6419	0.513965	0.46870	0.2	0.695	0.59	0.6115	0.6	0.3	0.4	0.7	0.366	0.59	0.3	0.69576	0.47	0.481	0.483	0.367	0.562

5656486_treat	6530995	953873	478683	8307	28	6346	21056495	991585	0940225	96751	58326007	54679268	07215396	50841343	028483	8484769	62950726	57	2057743	968311	608983	79533	647831
GSM5656487_treat	0.38632697	0.433676343	0.695946847	0.550980033	0.523795552	0.504096044	0.295936295	0.59909497	0.442504224	0.586347048	0.590028968	0.291269892	0.45218151	0.760424371	0.339615316	0.633220296	0.353796246	0.643096244	0.434983302	0.416753457	0.492698523	0.254644137	0.42269596
GSM5656490_treat	0.582903818	0.661166333	0.685767938	0.642295313	0.525660294	0.434021389	0.344321034	0.698104375	0.582467184	0.665705958	0.75142319	0.388300804	0.464688039	0.737622443	0.396229912	0.653347557	0.312037222	0.738818169	0.607109816	0.522823946	0.467500967	0.301138929	0.65288717
GSM5656493_treat	0.408085475	0.528211669	0.521556548	0.62021777	0.494114436	0.509148397	0.324954834	0.624560248	0.39665373	0.597153524	0.610169781	0.324467623	0.429519993	0.775960102	0.33987732	0.615693655	0.369523576	0.701588222	0.440667113	0.421684338	0.45974143	0.348241878	0.497044364
GSM5656495_t	0.266384636	0.488962363	0.550519057	0.596082525	0.5125063	0.446434587	0.29419	0.710812577	0.333313242	0.61056951	0.64171	0.32682	0.43047	0.75252	0.344788678	0.648933034	0.37997	0.67373456	0.52087262	0.435797257	0.456717913	0.290711005	0.48654064

reat							3756				1423	5602	0982	3335			1893						
GSM5656498_treat	0.309774477	0.527466099	0.556714703	0.669457623	0.498674619	0.4771574	0.394909739	0.730917577	0.366389696	0.681243064	0.701366792	0.391583131	0.53384809	0.736894209	0.380789265	0.635230356	0.409664038	0.688808187	0.581410367	0.497182567	0.483513705	0.357736742	0.578517485
GSM5656501_treat	0.424811625	0.60331186	0.521174183	0.576026375	0.512143855	0.410404144	0.245957966	0.737882089	0.466252391	0.63161724	0.564427943	0.314510724	0.40555996	0.737250435	0.344557442	0.642117497	0.179190824	0.694341619	0.496548649	0.470761487	0.430576621	0.283615137	0.629906984
GSM5656504_treat	0.395443405	0.585741575	0.574332509	0.55619284	0.50080142	0.391276537	0.33053594	0.754853912	0.496227955	0.641729284	0.622817913	0.347731562	0.44277551	0.698956774	0.350217838	0.624643275	0.324152081	0.727754787	0.548800496	0.466825284	0.447468842	0.303071786	0.627513621
GSM5656505_treat	0.328706573	0.518338498	0.553433471	0.658125229	0.52908074	0.550677944	0.255909403	0.661609995	0.420556485	0.664818977	0.674168713	0.426630812	0.495923737	0.750144606	0.434612829	0.653869973	0.472716999	0.769268218	0.532609351	0.458797879	0.518468368	0.345391405	0.541744104

GSM5656509_treat	0.477223789	0.54406762	0.542263907	0.596816843	0.488038236	0.446968668	0.252176368	0.658591567	0.44621415	0.609288309	0.584879653	0.313842567	0.315408398	0.745072401	0.33720117	0.611444741	0.304329132	0.672666054	0.471109084	0.453345135	0.462439631	0.326812799	0.502292518
GSM5656511_treat	0.472851016	0.625568318	0.555761442	0.548908345	0.49433647	0.424637836	0.253774317	0.682982398	0.512915582	0.623628658	0.518306319	0.326082302	0.390430144	0.709950581	0.348080912	0.625900006	0.290535922	0.668100084	0.467085843	0.469212225	0.445215084	0.325494406	0.611819942
GSM5656513_treat	0.341687194	0.593258381	0.664050438	0.578290126	0.520039998	0.477003727	0.244491219	0.714356147	0.381376529	0.60941728	0.65381993	0.344737929	0.483908054	0.752376946	0.350463437	0.616976888	0.344704435	0.688399641	0.547268847	0.467618548	0.470190787	0.342196025	0.552217679
GSM5656516_treat	0.276375691	0.66782818	0.605838522	0.560877652	0.495812374	0.388816477	0.338803223	0.690933057	0.415555538	0.617721949	0.549583076	0.302480079	0.439655455	0.670755259	0.345397226	0.656225728	0.182813173	0.671280931	0.479445565	0.460596276	0.440946561	0.270498183	0.654669715
GSM5656	0.261433	0.3893843	0.5050955	0.569735799	0.510474221	0.507949372	0.186	0.6384485	0.276655	0.54418002	0.416	0.246	0.314	0.757	0.3100815	0.536417	0.207	0.641558198	0.415885	0.4013070	0.4160955	0.3064036	0.3816519

517_treat	628	73	06				895629	25	436		178994	123688	362082	181411	68	404	488491		162	81	45	06	31
GSM5656520_treat	0.355800993	0.384397319	0.566907878	0.603930726	0.501038203	0.512990088	0.302775552	0.631969964	0.337686195	0.607144114	0.596000603	0.251024858	0.412989106	0.794146171	0.325323304	0.629242016	0.412572652	0.682966858	0.474092528	0.44608682	0.466176728	0.254892991	0.447733225
GSM5656521_treat	0.324820479	0.691515689	0.664062091	0.769586551	0.538590774	0.516644413	0.575748888	0.734650301	0.500019869	0.732591546	0.899056016	0.467314416	0.621555369	0.813022358	0.403726382	0.640014403	0.863993121	0.795948666	0.606636956	0.539961197	0.546806219	0.402512907	0.564848136
GSM5656522_treat	0.697167109	0.690574027	0.665750661	0.702029149	0.521198433	0.4488273	0.374816427	0.729323377	0.800048378	0.691368842	0.881123312	0.466583073	0.538505362	0.694461391	0.457357535	0.688286954	0.3781508	0.732824322	0.708520063	0.562794328	0.5740242	0.385745754	0.567504794
GSM5656524_treat	0.243354436	0.493584304	0.537969787	0.60222653	0.508693231	0.47787408	0.2095676	0.689033599	0.373734867	0.602648557	0.5434900	0.3047385	0.4467063	0.7525634	0.350138081	0.608077079	0.2506703	0.667282241	0.47462445	0.430399433	0.443056856	0.287646028	0.518138227

							74				97	96	35	96			48						
GSM5656525_treat	0.478483473	0.665548939	0.579695089	0.646161758	0.529713038	0.465469292	0.37259626	0.691243176	0.559324739	0.690684966	0.716997464	0.365404975	0.490231573	0.740526443	0.381126492	0.622028117	0.444542935	0.743695906	0.529727165	0.475391739	0.493263441	0.356376546	0.626190385
GSM5656527_treat	0.364723021	0.593523163	0.479611518	0.573656455	0.481545353	0.435241425	0.296358649	0.699149943	0.449886702	0.602311972	0.525615724	0.290709176	0.402084256	0.6999802	0.343723722	0.584997399	0.258606081	0.687185524	0.458252938	0.419674168	0.428140993	0.297796925	0.595756393
GSM5656528_treat	0.27973187	0.505744813	0.638634203	0.571868048	0.499186645	0.473230698	0.279969076	0.577403841	0.341981222	0.631029656	0.565073644	0.307068984	0.449482993	0.740102164	0.360454547	0.62132678	0.309208583	0.676180209	0.370565338	0.392015247	0.446482497	0.297685007	0.563587996
GSM5656531_treat	0.387091047	0.56983622	0.481445803	0.564658872	0.488915229	0.44940347	0.239847927	0.708907905	0.505800705	0.617726967	0.492661666	0.276760088	0.324605137	0.691721554	0.34871026	0.561215153	0.265770231	0.659364989	0.471121073	0.435636281	0.428198333	0.305602662	0.538060524
GSM	0.49	0.696	0.628	0.6153	0.515017	0.47213	0.2	0.707	0.58	0.6209	0.6	0.3	0.4	0.7	0.361	0.62	0.3	0.67278	0.53	0.448	0.469	0.370	0.557

5656533_treat	1139395	109652	49181	19223	596	8303	87631379	539699	9026533	87062	7041871	55172968	08121864	23767868	002997	5914659	47209412	7621	3844647	980393	258435	954757	422399
GSM5656535_treat	0.303941952	0.408368133	0.579497468	0.548282224	0.500766068	0.459968463	0.238708872	0.664033409	0.36148889	0.588452351	0.522781002	0.264107657	0.399976171	0.724011985	0.328728544	0.580742763	0.273524686	0.68121696	0.4143052	0.421955614	0.44003902	0.345005216	0.496853652
GSM5656536_treat	0.293130006	0.629470184	0.586228094	0.603951218	0.509448776	0.444240928	0.23983084	0.694641445	0.480962243	0.604883504	0.597754145	0.354188987	0.470257233	0.723500148	0.357738292	0.611458926	0.368748153	0.673456764	0.503583662	0.453323251	0.456150465	0.347815452	0.56352634
GSM5656541_treat	0.358738371	0.466195421	0.590280205	0.588658469	0.494742483	0.492959499	0.210643271	0.65965143	0.347633655	0.577426714	0.605163877	0.318013541	0.411669247	0.795887429	0.317282133	0.600942371	0.348320682	0.680505219	0.429487148	0.433406928	0.471539063	0.296289644	0.438730123
GSM5656543_t	0.241864739	0.616401657	0.491173911	0.569053745	0.528297029	0.450706375	0.23270	0.633539207	0.365892593	0.627069105	0.58277	0.33256	0.39212	0.71090	0.322292322	0.537890872	0.26793	0.671676344	0.364680362	0.395111641	0.395767459	0.346836957	0.591222308

reat							8453				7359	954	1923	2531			4807						
GSM5656545_treat	0.419299386	0.43084972	0.568416595	0.550251428	0.53504629	0.431721625	0.294669332	0.639017651	0.440192497	0.604977666	0.51451784	0.298398158	0.404041248	0.717968866	0.315718149	0.557996364	0.196469769	0.660292002	0.368908061	0.428218454	0.433133714	0.356293862	0.480267683
GSM5656549_treat	0.791256972	0.914864768	0.785027748	0.707616633	0.534206682	0.448853841	0.42582219	0.710435875	0.806020474	0.68495793	0.915570953	0.482126934	0.53320994	0.751166395	0.468393772	0.653194801	0.609345468	0.782460738	0.705895183	0.566053894	0.574742709	0.343753332	0.594473985
GSM5656551_treat	0.546816922	0.573505401	0.597826566	0.669200794	0.563416473	0.452724684	0.351220008	0.710927016	0.599415721	0.70326399	0.739845181	0.408225962	0.559555987	0.764645687	0.400244285	0.664148987	0.435685687	0.778296951	0.563922002	0.49551274	0.49751554	0.298031458	0.603319999
GSM5656554_treat	0.391681443	0.555942623	0.554771202	0.616637123	0.524593522	0.476845154	0.249790204	0.658973975	0.462324334	0.600883064	0.61325057	0.328700077	0.410326988	0.713362136	0.329482303	0.599581499	0.340948857	0.686320575	0.48950666	0.438004165	0.45859583	0.352964808	0.591548799

GSM5656556_treat	0.374135832	0.644689661	0.491856087	0.588787825	0.516077575	0.443010916	0.2664014888	0.691110381	0.418237869	0.603450637	0.57005604	0.337660988	0.384440478	0.704521891	0.317800854	0.578691633	0.277476156	0.684736002	0.480891829	0.420380011	0.443832647	0.347575869	0.626719527
GSM5656559_treat	0.25053137	0.557689759	0.485958178	0.60388057	0.516913534	0.451513555	0.270164048	0.68842427	0.455397891	0.626449596	0.598039664	0.319884185	0.429795519	0.71934243	0.32914463	0.589989094	0.21123469	0.691938729	0.470167229	0.438574645	0.431965866	0.369257139	0.584771628
GSM5656563_treat	0.191003565	0.374904654	0.441273446	0.554325309	0.504381481	0.469327546	0.159719869	0.690151448	0.396004038	0.5867686	0.518325856	0.317009728	0.354106597	0.754789835	0.35358714	0.594018941	0.324595236	0.63833487	0.469841421	0.437993138	0.446532971	0.346606249	0.441760546
GSM5656565_treat	0.325690903	0.498819376	0.518970053	0.548675996	0.495021181	0.440904046	0.307086143	0.66792342	0.369811681	0.590275217	0.527028718	0.292009564	0.38959109	0.71820535	0.33612811	0.597456013	0.24096265	0.651814688	0.449503461	0.454215932	0.42058	0.320578395	0.501028775
GSM5656	0.519805	0.7098314	0.6975394	0.625344324	0.53939213	0.417823732	0.346	0.7575212	0.608418	0.679327529	0.739	0.389	0.448	0.749	0.4072154	0.647840	0.248	0.706860978	0.602457	0.5371028	0.4871308	0.3504867	0.6703168

567_treat	344	33	74				978228	84	538		54707	151877	416253	181506	61	597	674035		978	1	56	88	2
GSM565659_treat	0.626006473	0.620176901	0.575476323	0.590884047	0.508046891	0.446580261	0.373219272	0.666449487	0.612873129	0.608809127	0.625306247	0.344686798	0.467416503	0.74418681	0.352388957	0.601912874	0.35350292	0.693933494	0.502445192	0.47925465	0.480566962	0.363519511	0.562851688
GSM565571_treat	0.330019268	0.521061361	0.566283302	0.663052439	0.489286181	0.500518428	0.326878115	0.691878725	0.439055951	0.617951715	0.644167792	0.361638003	0.415549039	0.760289989	0.360849799	0.623926653	0.445967782	0.679704258	0.527182244	0.46763523	0.467573459	0.345415599	0.476196114
GSM565573_treat	0.472812227	0.726942047	0.690349736	0.738472841	0.570348569	0.46603616	0.450318465	0.747457025	0.659148215	0.707226486	0.942785334	0.535895015	0.631477937	0.762923484	0.482641788	0.695508104	0.675117056	0.797246158	0.737714752	0.627574073	0.595563547	0.362919412	0.654878953
GSM565576_treat	0.520972944	0.679217624	0.601628805	0.696998784	0.541037087	0.449572812	0.3085803	0.735062565	0.53220914	0.670238852	0.7846808	0.3888941	0.4745001	0.7564533	0.393195158	0.639148841	0.4432304	0.740578182	0.577810573	0.510273735	0.500314449	0.374960236	0.62086016

							6				96	14	91	79			49						
GSM5656578_treat	0.2034761055	0.422493055	0.369341288	0.55048423	0.489899089	0.480901887	0.158141865	0.593408877	0.308230288	0.58590987	0.457913525	0.270581061	0.375422886	0.756613694	0.308472201	0.539341125	0.328260009	0.666090919	0.397903243	0.38450264	0.406769864	0.349571793	0.507199495
GSM5656581_treat	0.351870276	0.684646537	0.625561015	0.640625461	0.532338947	0.466872145	0.289970085	0.750337105	0.505309909	0.626825954	0.676710937	0.40879051	0.403822146	0.727117972	0.343367583	0.609709626	0.484760509	0.695153436	0.557096481	0.476771423	0.462997878	0.389822861	0.601552578
GSM5656583_treat	0.560562651	0.715383698	0.661227485	0.664511614	0.561778915	0.429942884	0.441531747	0.755023468	0.561939728	0.686190478	0.789049961	0.440190854	0.600141604	0.738647807	0.434736183	0.656446054	0.603800126	0.754600917	0.617991688	0.520979501	0.52183035	0.355556034	0.659353259
GSM5656585_treat	0.591596728	0.764322776	0.635423566	0.66494366	0.546100984	0.484999634	0.260374747	0.706727778	0.625920794	0.641758834	0.756412103	0.431990074	0.442396768	0.769322965	0.398523723	0.590064979	0.518787337	0.726030855	0.60462995	0.498071452	0.482482262	0.414594068	0.611374478
GSM	0.33	0.554	0.602	0.6618	0.529913	0.47744	0.3	0.726	0.46	0.6544	0.7	0.4	0.6	0.7	0.394	0.61	0.5	0.70580	0.58	0.505	0.482	0.364	0.543

5656588_treat	0379304	968339	546037	56645	92	9127	09853865	445841	3124812	62828	01236289	12556227	16395755	66857702	026251	7188128	18735382	2656	9528192	148374	273324	946001	036545
GSM5656589_treat	0.435600363	0.693326267	0.64966447	0.665834439	0.547569494	0.440232188	0.362689262	0.725183981	0.561390893	0.684847467	0.766864409	0.411528108	0.574048196	0.747441901	0.385011251	0.627099325	0.374432127	0.728961538	0.589551062	0.517992328	0.500280871	0.407958482	0.65249571
GSM5656591_treat	0.646297971	0.616876124	0.624905768	0.593518123	0.543171931	0.483209786	0.226608566	0.688118393	0.713325187	0.627255605	0.720808378	0.351789879	0.40524667	0.735280924	0.412477396	0.62914152	0.286017998	0.712389666	0.564773424	0.523518919	0.49532815	0.291394601	0.54549049
GSM5656595_treat	0.796913656	0.655812884	0.652300892	0.74670917	0.561200018	0.49036982	0.422791176	0.71602178	0.843705213	0.736515378	0.901949492	0.448349968	0.616394951	0.783780973	0.442905194	0.676564424	0.759348582	0.803949045	0.691284243	0.560693043	0.570728027	0.346845908	0.593569679
GSM5656596_t	0.371223298	0.551541339	0.566712123	0.528639026	0.514266089	0.421786412	0.36045	0.734382775	0.505490614	0.592977762	0.58922	0.34542	0.48967	0.70332	0.344690005	0.616363682	0.29851	0.6741401	0.522742965	0.499314633	0.470659321	0.300629936	0.579092324

reat							2291				1196	7804	1673	8834			7465						
GSM5656600_treat	0.915457576	0.688756071	0.738329432	0.627108293	0.515143315	0.440412478	0.432145173	0.677800615	0.9498491	0.61044153	0.842467825	0.4132384	0.521664615	0.745650104	0.447977703	0.643141679	0.253612925	0.743318137	0.661811909	0.569392599	0.53898804	0.259132556	0.541477698
GSM5656601_treat	0.333049854	0.529482031	0.574386698	0.580605826	0.482944343	0.457400919	0.281559398	0.694981919	0.45840125	0.606496468	0.552513528	0.313654403	0.471347147	0.732228314	0.343037186	0.610238168	0.300920684	0.685177972	0.456414186	0.447851592	0.458751821	0.349696258	0.580835646
GSM5656602_treat	0.493147631	0.62155272	0.607343389	0.6504254	0.515413156	0.435897538	0.390110631	0.727585669	0.549971828	0.679537471	0.767817682	0.416310788	0.669694338	0.719207412	0.381958229	0.697174234	0.5108529	0.737448574	0.605933272	0.507420016	0.542035576	0.334325849	0.570588047
GSM5656603_treat	0.196469665	0.459302929	0.56019895	0.553890062	0.509722414	0.48518368	0.210371103	0.643688258	0.247701973	0.596797644	0.496262333	0.272168722	0.409829787	0.704922942	0.338127177	0.584972602	0.369757619	0.672731445	0.398532965	0.377499214	0.444148258	0.325489431	0.549392934

GSM5656604_treat	0.212523813	0.344849778	0.452654189	0.517497729	0.465198369	0.391268549	0.155857341	0.600174539	0.307223378	0.638790691	0.427943294	0.262432499	0.506005637	0.651537261	0.345358873	0.612338132	0.219605696	0.67433547	0.406339873	0.404856407	0.412686978	0.20729989	0.506497417
GSM5656606_treat	0.40449827	0.516829474	0.635983123	0.600177092	0.501928332	0.498217457	0.186241806	0.660801072	0.40328074	0.624532226	0.599697862	0.342118304	0.398275219	0.78299843	0.356629366	0.632855647	0.332004103	0.674264732	0.424978418	0.429332179	0.468318485	0.337922129	0.509747994
GSM5656608_treat	0.524960676	0.588766256	0.576473416	0.609622314	0.533084452	0.435497844	0.37199604	0.67270274	0.501118136	0.640877028	0.648885628	0.341654222	0.480822912	0.744899341	0.35771127	0.63049157	0.31475028	0.68646007	0.497815541	0.464506395	0.472693417	0.352929575	0.561000224
GSM5656611_treat	0.571766148	0.521218098	0.628743706	0.610586093	0.467911091	0.438876999	0.216325463	0.695622985	0.574883628	0.606634498	0.620561605	0.314729433	0.458425187	0.750536341	0.369515213	0.643388439	0.310767682	0.711753613	0.484099481	0.465151498	0.479692529	0.302930398	0.51621158
GSM5656	0.272983	0.5429507	0.5640601	0.608131377	0.479147781	0.451162824	0.196	0.7044971	0.357541	0.60764208	0.603	0.340	0.460	0.732	0.3144175	0.589452	0.492	0.67086511	0.474687	0.4392312	0.4639675	0.3307771	0.5182181

612_treat	783	25	82				216695	97	175		686518	818299	971364	588559	07	881	722833		01	04	74	95	42
GSM5656615_treat	0.361097321	0.504563043	0.599928894	0.60289957	0.509295317	0.467113642	0.238821851	0.671101503	0.43436541	0.591038829	0.581708299	0.311447425	0.441077428	0.757384775	0.340851215	0.617421696	0.32952245	0.679453812	0.412647526	0.425384567	0.465189726	0.350759529	0.493085503
GSM5656617_treat	0.43375276	0.648922146	0.663144016	0.631757673	0.516671613	0.454925381	0.318437785	0.695378395	0.553510735	0.620210197	0.66754781	0.363196971	0.469372235	0.776370153	0.344144037	0.637723441	0.438426307	0.680929213	0.490209254	0.478750498	0.478467665	0.306041459	0.557131769
GSM5656619_treat	0.414495479	0.383917118	0.522546828	0.578502894	0.486522504	0.481175022	0.184301658	0.621162439	0.355764238	0.565661574	0.461943257	0.271313518	0.432861713	0.760377434	0.333557943	0.619840299	0.27394954	0.66598313	0.378441157	0.449220522	0.458678133	0.257578052	0.431913584
GSM5656621_treat	0.501679749	0.656902817	0.661865409	0.663636355	0.505338178	0.527866558	0.2518967	0.658264911	0.483073538	0.655630895	0.7856274	0.3839369	0.4870351	0.7762393	0.401516832	0.619157823	0.5152198	0.756806623	0.526296268	0.445449035	0.498600068	0.3272944	0.569038333

							15				36	6	78	67			09						
GSM5656624_treat	0.842726415	0.614236832	0.696532161	0.597049798	0.506498636	0.456463797	0.281684622	0.659319615	0.831814306	0.561053829	0.646838834	0.304494878	0.4504688	0.770689383	0.359564433	0.611285725	0.299966891	0.674852901	0.495339499	0.509423981	0.487410617	0.284701198	0.468978074
GSM5656625_treat	0.417540772	0.619101608	0.570357646	0.64864629	0.529618818	0.468496738	0.429340995	0.672263632	0.498300699	0.642428591	0.64467185	0.333829543	0.485934987	0.762006086	0.33562269	0.597105869	0.535957257	0.71972777	0.454001237	0.428923329	0.470464906	0.352864532	0.554617934
GSM5656627_treat	0.361969827	0.474833388	0.619843097	0.62857515	0.48202923	0.466409446	0.34199011	0.704200543	0.3917868	0.618728495	0.611466154	0.317837659	0.498137545	0.762943803	0.342480747	0.616724009	0.366046733	0.675830298	0.458250451	0.431813351	0.48088209	0.277268399	0.506593064
GSM5656631_treat	0.250116757	0.486387164	0.528625512	0.605261669	0.527682213	0.514949075	0.230479506	0.656184741	0.290856352	0.6012011	0.590160383	0.31954299	0.456282646	0.808033967	0.323088458	0.585868211	0.451981035	0.670891492	0.42244086	0.366584995	0.475114098	0.321745929	0.477512022
GSM	0.23	0.296	0.486	0.5183	0.457383	0.49080	0.1	0.573	0.37	0.5392	0.4	0.2	0.3	0.7	0.294	0.54	0.1	0.63444	0.34	0.355	0.430	0.272	0.358

5656632_treat	3454488	578795	251444	62855		1795	0835772	767218	318757	64726	37030758	6401581	34300027	89750652	687937	9740293	8687272	9419	731718	670379	770665	790957	582819
GSM5656635_treat	0.347095573	0.386709699	0.566779282	0.596891942	0.486428227	0.525126339	0.27268436	0.648966645	0.432313148	0.577719089	0.54407037	0.288160623	0.434117501	0.803805795	0.341446052	0.628604695	0.348696348	0.671088526	0.429269545	0.440431767	0.471617242	0.273997003	0.432292317
GSM5656637_treat	0.393788719	0.555108643	0.596878134	0.601125944	0.507661916	0.424731855	0.261186972	0.717866555	0.48313388	0.615393903	0.577141207	0.341376241	0.482948717	0.717754431	0.33880337	0.625516052	0.393065877	0.676344113	0.463521709	0.444193788	0.464229435	0.321141962	0.573074487
GSM5656639_treat	0.273558468	0.431186943	0.55189071	0.551829778	0.498126975	0.440977564	0.245350683	0.668723855	0.313755634	0.576174796	0.437946123	0.278794739	0.386934051	0.686905081	0.335435641	0.640524967	0.305674276	0.672948151	0.407587649	0.43436987	0.459372183	0.306425801	0.521353816
GSM5656641_t	0.670117922	0.654081214	0.615801162	0.628888961	0.496160524	0.491785128	0.40630	0.670847992	0.643722756	0.64069768	0.73459	0.35657	0.56784	0.77303	0.410848458	0.691186074	0.35896	0.732399978	0.542166763	0.534005195	0.533758977	0.291791249	0.564814942

reat							0443				4912	5065	9639	1189			234						
GSM5656645_treat	0.519411042	0.496998385	0.642991784	0.616077323	0.532712183	0.415106636	0.276368627	0.70529381	0.5591433	0.660647761	0.657265269	0.366529553	0.42670181	0.749715903	0.385398495	0.679589394	0.257987468	0.716968269	0.543028845	0.515793866	0.488673776	0.316666808	0.547851279
GSM5656647_treat	0.292375049	0.434838978	0.486800799	0.510101302	0.507431524	0.457041648	0.137495728	0.626370112	0.361342787	0.549022368	0.344408419	0.254856689	0.403498814	0.73906693	0.302530363	0.581247502	0.280275682	0.662548264	0.337386451	0.383572454	0.432988964	0.324924714	0.502440913
GSM5656648_treat	0.243802224	0.546414467	0.538527397	0.577643887	0.534716239	0.507604098	0.289143247	0.684002689	0.35503953	0.595446047	0.554708624	0.304788549	0.329827999	0.769104919	0.312152492	0.562432605	0.266176963	0.660476998	0.469963077	0.416404958	0.443804932	0.327151236	0.504000552
GSM5656653_treat	0.211974117	0.346502378	0.519870558	0.553200539	0.476630133	0.4552464	0.227989202	0.664791772	0.351941944	0.552271066	0.414143659	0.275139689	0.373769899	0.730063274	0.319627779	0.57669773	0.300208435	0.647830328	0.360930501	0.395300398	0.450955839	0.310155769	0.457168635

GSM5656655_treat	0.742172709	0.740334559	0.672850403	0.657156101	0.532131483	0.460382473	0.364307534	0.695859337	0.786850391	0.679228605	0.857987783	0.386264663	0.563420109	0.747645707	0.444354605	0.682158536	0.307645788	0.732401122	0.645048718	0.54697675	0.564531687	0.317803043	0.59207707
GSM5656657_treat	0.647507514	0.671843504	0.679806623	0.680949255	0.522424307	0.514029416	0.280233139	0.66219715	0.635034882	0.600395117	0.70870378	0.344643095	0.445857528	0.807213314	0.348709906	0.629704205	0.430644523	0.693479657	0.477429143	0.459270935	0.513245332	0.372197763	0.541157283
GSM5656173_treat	0.596749727	0.46037851	0.574138252	0.552807064	0.488723068	0.478079642	0.191300387	0.669684388	0.483972492	0.545912787	0.489149765	0.261136697	0.330523602	0.751720596	0.31512219	0.576798129	0.210702917	0.646139726	0.384144265	0.431231382	0.495438184	0.29572355	0.44462472
GSM5656182_treat	0.190676647	0.32368956	0.581559284	0.561975181	0.49454273	0.491586135	0.247297515	0.643444669	0.343515175	0.558534236	0.478376781	0.240574899	0.377379545	0.764971412	0.314407832	0.580945783	0.33018327	0.664381144	0.368545044	0.422714151	0.443263567	0.272822068	0.407893107
GSM5656	0.510690	0.6535651	0.6796321	0.615466323	0.452066594	0.472991275	0.320	0.7286224	0.489082	0.615653836	0.670	0.362	0.473	0.686	0.3835510	0.520709	0.478	0.669924012	0.576630	0.4647080	0.4703713	0.3514539	0.5891917

191_treat	857	86	21				713878	12	176		081946	927631	959236	809005	96	586	106488		419	59	09	18	56
GSM5656194_treat	0.5611621136	0.548496656	0.641057794	0.583558631	0.505488726	0.459697712	0.263668582	0.674419607	0.499315515	0.577967992	0.564695252	0.292056516	0.429314405	0.748733984	0.344233111	0.623448996	0.347553579	0.640911283	0.454122082	0.446539336	0.485590161	0.284432683	0.493012541
GSM5656196_treat	0.231981916	0.397860765	0.497995827	0.527604152	0.485664491	0.459916748	0.191499223	0.626274057	0.223928406	0.533455384	0.379084162	0.225773763	0.416926269	0.757748503	0.30522075	0.53467621	0.285072307	0.655250043	0.287297062	0.379557025	0.418311067	0.297258952	0.42809531
GSM5656198_treat	0.418225752	0.590108847	0.582784222	0.589759907	0.50168592	0.431186782	0.307848244	0.69317903	0.509432361	0.579084388	0.592648915	0.334817872	0.476853523	0.755346553	0.320626166	0.601757354	0.378023072	0.668413987	0.456667142	0.430363038	0.455497312	0.327946269	0.563783171
GSM5656205_treat	0.438646347	0.427113533	0.461158529	0.547864107	0.450424101	0.538157331	0.2412328	0.580815533	0.441890914	0.54514215	0.4668799	0.2632107	0.2512499	0.7177042	0.310730377	0.531421573	0.1704741	0.680259262	0.417270778	0.33351735	0.420153152	0.344757498	0.379894024

							46				08	53	17	15			72						
GSM5656210_treat	0.471373751	0.624887694	0.641578425	0.643510158	0.509747787	0.461979162	0.415304662	0.701497512	0.504904984	0.655476078	0.701561003	0.368458917	0.575903482	0.762914706	0.382004629	0.653557187	0.517872426	0.696919009	0.561956638	0.463302466	0.496982225	0.32717055	0.604766633
GSM5656211_treat	0.120779081	0.484071493	0.583397356	0.529848781	0.4979219	0.464573286	0.223153773	0.660268431	0.323493176	0.523436534	0.493830822	0.269952537	0.369657459	0.708585169	0.317852575	0.54126308	0.339267318	0.610197516	0.431566389	0.39061448	0.446719161	0.278656473	0.468328228
GSM5656215_treat	0.152060169	0.24639978	0.536502059	0.472528689	0.488415656	0.490671342	0.086873639	0.589117606	0.264687917	0.526836497	0.354873228	0.207819824	0.339484972	0.765039102	0.297480091	0.57201221	0.266187529	0.611511237	0.259367822	0.363608048	0.435989748	0.244166387	0.360570767
GSM5656218_treat	0.304059682	0.512929501	0.620977143	0.572272012	0.484661033	0.506696712	0.20686396	0.685583957	0.382431753	0.545196525	0.501860224	0.291293778	0.331617305	0.703551911	0.34609181	0.4707536	0.33196037	0.610576483	0.429775647	0.446352518	0.434840918	0.329808336	0.448382382
GSM	0.30	0.403	0.536	0.5341	0.497419	0.42860	0.2	0.649	0.33	0.5573	0.4	0.2	0.3	0.7	0.296	0.57	0.3	0.63425	0.35	0.404	0.431	0.284	0.447

5656219_treat	8005368	752604	812158	41534	848	6808	48670116	263751	4658918	50078	75944243	51815069	89427697	43321962	113221	9492653	22086641	9687	0852006	865739	410815	82722	062788
GSM5656222_treat	0.18427848	0.331539879	0.620544556	0.532427538	0.478273572	0.530824646	0.133803984	0.601993641	0.307286072	0.506764539	0.457523694	0.230806546	0.263376392	0.781643292	0.32388746	0.519354553	0.319574357	0.615920408	0.345820987	0.384480942	0.442658758	0.292892204	0.290786573
GSM5656227_treat	0.642069664	0.463883667	0.567465132	0.56815109	0.513260258	0.494964813	0.334166742	0.624370889	0.535666923	0.576174529	0.526927914	0.311256272	0.371106405	0.73554082	0.323700058	0.574085619	0.139843201	0.677457336	0.400303254	0.441953952	0.440511392	0.359852098	0.47918143
GSM5656234_treat	0.244479061	0.372771019	0.567806023	0.513930329	0.477453408	0.481735635	0.135108689	0.666368081	0.31619519	0.541823922	0.398583772	0.223790109	0.290465563	0.75195316	0.31222204	0.557432324	0.202338062	0.627269498	0.316035463	0.394410753	0.435570493	0.282929676	0.417618118
GSM5656236_t	0.208899873	0.61106627	0.516420105	0.550132864	0.500756105	0.434348133	0.33020	0.660216839	0.350690143	0.560010159	0.50354	0.30649	0.36593	0.70046	0.324348263	0.544341426	0.33095	0.638900887	0.420544605	0.389856584	0.445398959	0.312418903	0.56379806

reat							1065				3034	4919	5185	0891			7128						
GSM5656239_treat	0.512288038	0.63361397	0.638691366	0.639492561	0.490072459	0.455404988	0.31199099	0.723714403	0.551702473	0.664066422	0.706649412	0.419896216	0.533807444	0.656499683	0.411620649	0.523348987	0.588335258	0.675425794	0.609874354	0.472943323	0.473660068	0.391944622	0.593858291
GSM5656241_treat	0.309754076	0.65706796	0.586394831	0.546890734	0.443579714	0.46927573	0.239072301	0.696765386	0.358024337	0.593204547	0.520866881	0.292622335	0.414879286	0.653841681	0.360592589	0.480851462	0.352442189	0.615824752	0.46334785	0.393134927	0.434228741	0.33163774	0.580948238
GSM5656244_treat	0.230320475	0.291679637	0.503413069	0.541797124	0.486150642	0.510926765	0.187297623	0.613057272	0.319057522	0.516586251	0.422947106	0.235317446	0.379583467	0.75959757	0.313649195	0.56350398	0.335217838	0.598349457	0.334207239	0.362183264	0.423341784	0.265017527	0.355859329
GSM5656247_treat	0.433279426	0.726159739	0.613656203	0.637315186	0.494921531	0.440836657	0.395552507	0.746482779	0.532058665	0.661568457	0.748552962	0.415618709	0.519413945	0.691532299	0.388486516	0.555071121	0.50974905	0.681915561	0.599348708	0.48935555	0.454927791	0.36681114	0.63695397

GSM5656251_treat	0.263390188	0.389225478	0.476846404	0.535352291	0.464620311	0.460076668	0.172517795	0.643490102	0.292601424	0.538693457	0.424578887	0.266705106	0.32922571	0.753919435	0.310302947	0.5465184	0.287969042	0.621007054	0.338816302	0.395822526	0.428182364	0.292123654	0.394645851
GSM5656253_treat	0.379373929	0.492631342	0.550754982	0.595775342	0.505854604	0.492886743	0.182074541	0.641427568	0.377749256	0.586497043	0.600758099	0.338428572	0.476464588	0.771730482	0.334388549	0.583107539	0.389851749	0.666203923	0.479628618	0.433509099	0.437812404	0.280995072	0.477293081
GSM5656256_treat	0.373244161	0.673742904	0.640519867	0.588319356	0.480984053	0.46893421	0.428690017	0.719803512	0.450119726	0.603030941	0.594177097	0.33820349	0.555141939	0.674417792	0.384047091	0.517994407	0.431797365	0.624573077	0.552114934	0.464533651	0.453103798	0.3147552	0.629418005
GSM5656263_treat	0.294676401	0.603308723	0.539889524	0.548975696	0.453925641	0.453539654	0.252256494	0.66889479	0.439609016	0.552387505	0.49263574	0.292655871	0.371835236	0.681355741	0.353217264	0.495020088	0.33010527	0.612974472	0.461119109	0.418755861	0.419670294	0.320011193	0.546915626
GSM5656	0.308278	0.4181528	0.6160901	0.612662104	0.533078314	0.485263459	0.144	0.6695959	0.391085	0.550387748	0.563	0.323	0.384	0.794	0.3290842	0.598087	0.401	0.635777696	0.448595	0.4139004	0.4733384	0.2967801	0.4064079

268_treat	657	71	04				920531	1	006		119769	70957	690715	299866	54	769	552252		877	71	79	65	27
GSM5656272_treat	0.318838474	0.466161194	0.548941802	0.506635115	0.456675369	0.454327637	0.149083086	0.637812422	0.397431397	0.554827568	0.383341722	0.246044594	0.319829478	0.73018248	0.304160142	0.496216718	0.272725253	0.631899836	0.363507937	0.392504442	0.410107771	0.262304253	0.455036128
GSM5656275_treat	0.341333452	0.512871906	0.684334308	0.595561831	0.498027542	0.452875336	0.203885693	0.717600707	0.353631827	0.596227091	0.586736094	0.33288231	0.420094062	0.772449975	0.341131048	0.617418527	0.277239472	0.690541802	0.489892121	0.432651891	0.491333081	0.349336736	0.541146563
GSM5656278_treat	0.358864997	0.477547378	0.554360114	0.511948827	0.506639893	0.391402941	0.450281238	0.637352968	0.338996342	0.592867535	0.486458365	0.248177038	0.567925927	0.700175001	0.328041405	0.616632089	0.206988668	0.658752736	0.357150819	0.415927189	0.463881593	0.250378173	0.547808223
GSM5656281_treat	0.200746069	0.368765512	0.516073075	0.542827525	0.482619213	0.491186676	0.199004	0.624736963	0.309057631	0.535809256	0.3634162	0.2709405	0.3125266	0.7531056	0.314042011	0.525092634	0.3144703	0.622901602	0.38478388	0.406647007	0.435684669	0.283168534	0.402000126

							69				07	2	66				36						
GSM5656286_treat	0.246593898	0.347543945	0.544750487	0.575524927	0.49160026	0.485117392	0.2190088889	0.644156327	0.266426196	0.577017726	0.50582945	0.26996073	0.419808604	0.771683873	0.308794417	0.611995509	0.374604433	0.645272571	0.355522777	0.386946487	0.459617748	0.287603064	0.443046263
GSM5656289_treat	0.204464621	0.479342415	0.464775525	0.525532852	0.475869641	0.443063324	0.23852061	0.681186553	0.417452088	0.555633686	0.443414419	0.285168217	0.405720121	0.740278677	0.330440019	0.559447105	0.30347693	0.630987358	0.396559722	0.40977113	0.431793773	0.311654581	0.503944252
GSM5656293_treat	0.240575605	0.524865416	0.540766477	0.56106542	0.484412692	0.440536321	0.217663954	0.753707105	0.385456281	0.581610676	0.588571508	0.362024826	0.459983733	0.749383021	0.328450337	0.578844905	0.398262085	0.629393958	0.495235615	0.459260612	0.472716194	0.310684995	0.535730078
GSM5656296_treat	0.483184056	0.679285973	0.628918183	0.601562909	0.49910123	0.450203423	0.315923831	0.727380395	0.506317394	0.593083343	0.635124995	0.361547317	0.519304883	0.679771957	0.391196503	0.550794695	0.434053215	0.658130348	0.564497508	0.477497705	0.458302714	0.340696918	0.591178792
GSM	0.25	0.326	0.578	0.5514	0.468471	0.44833	0.2	0.647	0.27	0.5393	0.4	0.2	0.4	0.7	0.316	0.59	0.2	0.62838	0.31	0.390	0.458	0.314	0.439

5656301_treat	4128393	270604	827632	32364	065	9335	49197878	396536	2130139	18182	31515343	27867492	15507777	66487485	227128	8867234	4489459	8639	9975486	452855	860313	975058	456552
GSM5656307_treat	0.258347246	0.30146266	0.534745874	0.552732955	0.477335993	0.481959228	0.078932431	0.614242466	0.235075603	0.548517488	0.463947255	0.262993318	0.395070038	0.783615958	0.335558093	0.574960784	0.292785686	0.655810717	0.333111685	0.365494585	0.451328949	0.264115926	0.423881649
GSM5656308_treat	0.53443219	0.592724628	0.618364628	0.563865034	0.49687194	0.445190482	0.31879893	0.700627591	0.507429223	0.582111722	0.610227147	0.360642024	0.482632215	0.711636768	0.355383202	0.571637983	0.373107861	0.66377006	0.507017189	0.47345165	0.45623839	0.314733126	0.551353936
GSM5656320_treat	0.212309733	0.358853027	0.508828209	0.542561744	0.471496537	0.425574891	0.296158713	0.625673693	0.323176802	0.597564834	0.493624256	0.287152769	0.572431862	0.721941012	0.331106053	0.623279732	0.288058675	0.653324277	0.40673511	0.412078436	0.436794467	0.250523372	0.466202956
GSM5656324_t	0.394194884	0.55025107	0.593497731	0.549996923	0.495492934	0.434633844	0.24866	0.668301195	0.385977776	0.586154157	0.51386	0.26665	0.46479	0.73845	0.322628163	0.634156963	0.29365	0.663617256	0.399515002	0.43035397	0.46454042	0.290901073	0.550338884

reat							9216				7699	0983	231	8653			0638						
GSM5656326_treat	0.389646991	0.465016703	0.634229795	0.54564435	0.505278758	0.462275183	0.236660023	0.693519616	0.448272474	0.576224606	0.476347001	0.279137243	0.433057655	0.767130575	0.35318227	0.606222966	0.286293835	0.612316818	0.413224177	0.427312236	0.476579031	0.287325814	0.488833915
GSM5656328_treat	0.28521267	0.468939828	0.592561405	0.578462419	0.516441576	0.496862959	0.25224952	0.650191297	0.319469499	0.56831253	0.524792399	0.29681338	0.451894024	0.759375977	0.329470595	0.592366693	0.370808025	0.636046266	0.429354166	0.411143506	0.455537749	0.310220559	0.497050186
GSM5656331_treat	0.626256466	0.677494492	0.656450394	0.638394538	0.539847633	0.439491991	0.396056416	0.756246324	0.673330226	0.664302274	0.800984649	0.421418253	0.559220059	0.728537232	0.402063401	0.664541849	0.381808962	0.750927333	0.603630272	0.52344038	0.524652934	0.400811071	0.571991489
GSM5656332_treat	0.24018331	0.258429574	0.42832607	0.473813135	0.458800547	0.443209932	0.128615865	0.619450325	0.279736897	0.581932702	0.365913804	0.285128034	0.457313763	0.702210819	0.367709395	0.595077667	0.188976713	0.651066213	0.366944939	0.392326676	0.418235691	0.230683169	0.423325745

GSM5656336_treat	0.520386239	0.442128599	0.546985922	0.509516287	0.485743871	0.451119201	0.225258651	0.659038752	0.539507986	0.551821436	0.378515215	0.247846478	0.398159079	0.741243026	0.31462416	0.577285222	0.246001596	0.615035131	0.340380882	0.415262825	0.458652233	0.263469478	0.456500031
GSM5656340_treat	0.27755957	0.530020917	0.585337634	0.571031424	0.524977921	0.480948169	0.255436693	0.626532705	0.328038613	0.629779368	0.514531217	0.250892413	0.326751776	0.72011168	0.302003993	0.618790015	0.321599169	0.669772624	0.408146108	0.363014257	0.425292984	0.3151071	0.568233598
GSM5656343_treat	0.383975709	0.561228249	0.66525059	0.552047716	0.497431423	0.438146516	0.22438009	0.690612188	0.376816256	0.59180762	0.534015229	0.278581413	0.457408901	0.762072783	0.344967056	0.610345841	0.258403383	0.643672755	0.40038077	0.445028213	0.492914001	0.300342472	0.522415269
GSM5656345_treat	0.52100565	0.503341627	0.588918092	0.546890037	0.508853711	0.440779421	0.301643243	0.640074861	0.566627533	0.557162747	0.500743101	0.269361491	0.419457338	0.755390209	0.324371437	0.622118361	0.28582031	0.662161069	0.42001939	0.428949529	0.473052085	0.279767263	0.488221342
GSM5656	0.227758	0.3044886	0.5547419	0.547870835	0.491836863	0.497914863	0.157	0.6076514	0.232795	0.538448293	0.427	0.214	0.385	0.759	0.3005151	0.624019	0.266	0.626990428	0.293140	0.3998347	0.4603581	0.2641890	0.3737065

349_treat	257	38	48				023525	62	216		771591	471965	949137	066609	97	607	735023		107	76	42	84	48
GSM5656350_treat	0.247093943	0.424976832	0.597530201	0.539606216	0.481731324	0.465219062	0.215768198	0.643685558	0.349297864	0.550725285	0.429196379	0.243855562	0.348715525	0.75780993	0.307513196	0.547454368	0.284175782	0.621366488	0.334004938	0.386436182	0.456189705	0.296336955	0.452402298
GSM5656354_treat	0.296849741	0.302718904	0.555857804	0.5437682	0.483837415	0.47493091	0.109248098	0.636693471	0.323069647	0.529600678	0.416945664	0.235216927	0.336762775	0.781375633	0.313987981	0.581547955	0.256856295	0.622711677	0.345856826	0.415409674	0.462436814	0.267012183	0.377791431
GSM5656357_treat	0.304764746	0.48249209	0.539110721	0.560280233	0.513938788	0.451918753	0.290266748	0.678154399	0.327813515	0.58388476	0.512327641	0.26597731	0.555029256	0.718037086	0.329796638	0.607447088	0.312013912	0.667168755	0.414027546	0.440084471	0.454683994	0.295935561	0.537760501
GSM5656362_treat	0.2116382	0.346661388	0.523739816	0.504905173	0.477849712	0.451397364	0.3285359	0.630213304	0.258960885	0.586612648	0.4697587	0.2631673	0.5751113	0.6823922	0.328891238	0.635512734	0.2446751	0.637500689	0.350616322	0.398468367	0.471697553	0.265219336	0.449085755

							61				26	67	59	59			81						
GSM5656363_treat	0.230404698	0.34541284	0.539399502	0.508400701	0.501294334	0.439279037	0.270383974	0.639197455	0.267494023	0.598167271	0.481809142	0.283732234	0.507856573	0.725582244	0.334666706	0.600907943	0.342987755	0.648824445	0.341410734	0.424101799	0.451331637	0.277170078	0.461831744
GSM5656367_treat	0.206954387	0.365230004	0.476129221	0.544547475	0.462901534	0.421620544	0.345335784	0.650557159	0.366710995	0.60781107	0.540644087	0.299130832	0.663802146	0.691787136	0.364885525	0.628858182	0.37800591	0.640106818	0.416476638	0.426733404	0.482748904	0.263333446	0.490996703
GSM5656370_treat	0.261535257	0.494199857	0.537774311	0.560265431	0.522411732	0.476906095	0.172962632	0.674854875	0.357058561	0.587635018	0.476083626	0.278271949	0.412119556	0.757629546	0.301450754	0.586751342	0.358383851	0.650533398	0.41839744	0.433519301	0.441542464	0.301229419	0.524410636
GSM5656371_treat	0.737489722	0.649532244	0.618829657	0.554701563	0.529157182	0.498970427	0.365818363	0.699821909	0.728142423	0.601495861	0.610945135	0.317566593	0.489672606	0.709438527	0.340197739	0.609411836	0.165334617	0.676674474	0.507212253	0.488675971	0.48308389	0.351427053	0.514482623
GSM	0.16	0.325	0.537	0.5356	0.484109	0.48067	0.1	0.650	0.36	0.5492	0.4	0.2	0.3	0.7	0.301	0.52	0.3	0.61373	0.34	0.417	0.429	0.284	0.375

5656373_treat	0477621	709494	17299	25363	034	001	1963174	679064	0403005	40823	51088751	58827597	40374434	52289954	901079	5313061	0391195	2196	3548939	93236	329867	881945	443908
GSM5656377_treat	0.254121207	0.319378744	0.56289302	0.499395739	0.473947493	0.438368677	0.233458827	0.588524162	0.368217758	0.617344172	0.520538615	0.304412542	0.434942162	0.639822692	0.336497058	0.607845895	0.307354558	0.662529259	0.406441571	0.404783572	0.452218299	0.212514135	0.439660794
GSM5656378_treat	0.567768818	0.563819169	0.622651624	0.582013132	0.526663399	0.402619774	0.419073942	0.710052661	0.628145038	0.64617051	0.629359869	0.345350336	0.506384495	0.67579544	0.388478746	0.622605053	0.231179115	0.657279681	0.535942099	0.499036057	0.4711697	0.290329331	0.624918216
GSM5656379_treat	0.274529959	0.541156805	0.573263696	0.539986971	0.460647763	0.449287877	0.278202691	0.679334847	0.465860946	0.559355742	0.520510894	0.2930111	0.401400666	0.681405428	0.318003613	0.520531016	0.413090705	0.627510212	0.441766738	0.3929711	0.438312026	0.273510993	0.501198882
GSM5656382_t	0.313983459	0.440740414	0.564149469	0.546539004	0.483958552	0.463786587	0.17575	0.643000318	0.400885197	0.538543806	0.44059	0.26366	0.31953	0.76444	0.315276391	0.51320045	0.29766	0.643263343	0.399637743	0.411006937	0.438018066	0.281150185	0.424542934

reat							9428				4156	057	5771	0918			3992						
GSM5656386_treat	0.246408531	0.351003906	0.591343897	0.534098867	0.500682407	0.500321776	0.148726076	0.568106501	0.449090763	0.531756914	0.466413925	0.229003314	0.295898554	0.756037413	0.302291964	0.457792124	0.241929901	0.604191163	0.347919484	0.377750633	0.433049134	0.248924039	0.337673839
GSM5656391_treat	0.532750798	0.545343114	0.587917979	0.584402625	0.493444175	0.470372789	0.296573338	0.649313472	0.592481155	0.585289741	0.598256961	0.299551412	0.43503356	0.756573661	0.328220185	0.554695987	0.339154779	0.640171628	0.452586854	0.431664668	0.464110836	0.29615371	0.519529191
GSM5656397_treat	0.315120607	0.595193515	0.5267229	0.59269847	0.501118012	0.463477037	0.258150207	0.716489311	0.375114793	0.606062736	0.589543837	0.330606789	0.485347665	0.705672547	0.36886495	0.547997972	0.492969748	0.672207374	0.527164427	0.442783936	0.424112051	0.368329566	0.553901658
GSM5656401_treat	0.223104027	0.427768173	0.578769714	0.570761045	0.485558308	0.505391693	0.127709768	0.605294068	0.322305147	0.545001084	0.38509949	0.261721542	0.325138364	0.760679823	0.308162172	0.526338002	0.251204537	0.645286575	0.348553793	0.385936485	0.418404331	0.316478277	0.447263235

GSM5656402_treat	0.226551108	0.391808719	0.509725809	0.523795958	0.488389901	0.491902189	0.124542005	0.603062001	0.299909075	0.527397853	0.358424856	0.229757509	0.260544576	0.741391856	0.306118338	0.497931593	0.255610802	0.648254456	0.299374095	0.363082789	0.399790029	0.292682161	0.376220617
GSM5656404_treat	0.285014738	0.577837939	0.540589741	0.514654655	0.484726712	0.418169296	0.243911073	0.680393203	0.378229169	0.549656961	0.448998442	0.264532712	0.33635835	0.657920736	0.310505806	0.528374343	0.262987116	0.631489939	0.398943506	0.385521268	0.435856207	0.298056525	0.558893221
GSM5656408_treat	0.272214033	0.460701447	0.571015989	0.528406395	0.489497131	0.467791733	0.278252937	0.674497917	0.332432319	0.543139313	0.439527816	0.272713082	0.361290806	0.749926853	0.306349073	0.5524194	0.319027031	0.635768335	0.370347304	0.413893293	0.443551729	0.289424792	0.455288476
GSM5656411_treat	0.269565111	0.498154678	0.583713746	0.581066016	0.491673588	0.473830058	0.208446738	0.664163057	0.429142305	0.58869954	0.563479108	0.34369402	0.417092629	0.732548888	0.296049755	0.520112636	0.415517462	0.649077141	0.435156274	0.430974247	0.455753951	0.261926371	0.46093443
GSM5656	0.190724	0.5161337	0.5162040	0.524835134	0.492821741	0.434239798	0.273	0.6626789	0.392745	0.554953633	0.429	0.266	0.371	0.715	0.2927916	0.498838	0.270	0.638485999	0.377588	0.3924472	0.4196451	0.3281432	0.5090096

413_treat	579	27	47				877996	19	774		302917	99568	318546	673028	88	486	443899		041	13	81	87	02
GSM5656415_treat	0.280619706	0.437279732	0.548987157	0.514734418	0.489590075	0.466447362	0.185689721	0.620394145	0.345874762	0.56033715	0.496076969	0.262458277	0.35676461	0.735763926	0.293080829	0.535942168	0.359636772	0.626470385	0.36600585	0.388429454	0.436950613	0.288344617	0.411840115
GSM5656419_treat	0.252638671	0.483658589	0.591899305	0.571701199	0.476134746	0.46687556	0.240606152	0.67869655	0.310195121	0.575831861	0.496293591	0.247820248	0.370435316	0.741905529	0.316836743	0.544494502	0.313271516	0.643673114	0.410593147	0.415058955	0.474482355	0.301623873	0.516214631
GSM5656422_treat	0.432173332	0.460408497	0.532577954	0.570095754	0.492579585	0.467076043	0.176674465	0.605611983	0.370445142	0.57257846	0.437999817	0.274303286	0.325834104	0.756296733	0.303627317	0.510166994	0.267359478	0.659877881	0.376406387	0.387451733	0.435414778	0.284907564	0.479032661
GSM5656427_treat	0.332385014	0.529956677	0.495016417	0.516339244	0.487539574	0.476841988	0.2330196	0.667675999	0.454217275	0.560554449	0.4380680	0.2558837	0.3521764	0.7255138	0.303050994	0.518124783	0.1914065	0.642908045	0.402134049	0.409526889	0.433139239	0.37576807	0.491009968

							4				12	88	1	25			19						
GSM5656433_treat	0.12926126	0.392987124	0.585358364	0.606899158	0.501307761	0.54285017	0.196631799	0.605194923	0.408482906	0.556311707	0.56238798	0.294444138	0.399046073	0.753054358	0.302201849	0.425743386	0.375061942	0.617407526	0.428064081	0.360894595	0.463496948	0.326535027	0.308080773
GSM5656436_treat	0.597201428	0.575048619	0.528621358	0.543224034	0.486118626	0.421498469	0.300256886	0.688661457	0.658096385	0.573738946	0.526167869	0.29043566	0.387999985	0.69890141	0.321160995	0.550974286	0.231506169	0.614844574	0.451570617	0.459676083	0.444309446	0.283997757	0.50545846
GSM5656438_treat	0.644952783	0.63884289	0.598856653	0.54757956	0.480192421	0.436195685	0.371084699	0.670307188	0.671404266	0.596780713	0.528589522	0.313635067	0.402622308	0.689162229	0.362509658	0.557423328	0.227846325	0.649806129	0.486184848	0.474551345	0.460347659	0.314343973	0.578282556
GSM5656440_treat	0.296716975	0.510417527	0.594473362	0.589053571	0.483059725	0.458765111	0.271348593	0.659438548	0.377327202	0.573815208	0.544342402	0.299720433	0.465216047	0.757641536	0.324628126	0.555946598	0.305513335	0.650239382	0.458592499	0.444823673	0.462033177	0.275100813	0.473618182
GSM	0.25	0.389	0.576	0.5317	0.482460	0.50143	0.1	0.657	0.30	0.5430	0.3	0.2	0.3	0.7	0.308	0.53	0.2	0.63033	0.35	0.410	0.451	0.291	0.379

5656442_treat	0065299	726301	091962	09859	598	865	51764361	927838	8272318	15211	90750068	53758364	24319251	48254717	285488	9318995	97861576	6566	8337933	913064	248491	246809	797211
GSM5656446_treat	0.247434167	0.526377538	0.542680709	0.537357596	0.447681868	0.461746669	0.170460459	0.663304597	0.434216058	0.544388978	0.465080382	0.282961809	0.271776469	0.670591587	0.325561874	0.494261897	0.325921331	0.610055119	0.412464188	0.386143767	0.41009052	0.347295789	0.466949331
GSM5656449_treat	0.189811897	0.250559856	0.567561673	0.513686863	0.48951938	0.486887945	0.216239535	0.608306184	0.248467032	0.517114236	0.337437328	0.20440835	0.318684962	0.724217597	0.287070339	0.570181622	0.260740709	0.653129269	0.274076549	0.369205442	0.43961082	0.266491723	0.370471057
GSM5656453_treat	0.42408619	0.401216376	0.556709602	0.54050856	0.487678589	0.471082081	0.206422637	0.644232768	0.520539524	0.544437083	0.513948085	0.291194229	0.394463552	0.762878037	0.32750084	0.589821735	0.337752342	0.610766599	0.389401841	0.404533466	0.464599655	0.257557788	0.385385831
GSM5656456_t	0.59726765	0.535198647	0.573795032	0.547155807	0.523213493	0.451156397	0.25514	0.663498538	0.545330829	0.608179336	0.49916	0.25826	0.37534	0.68449	0.344685294	0.604814097	0.22044	0.657008254	0.476451045	0.460203624	0.441586433	0.249505746	0.570605836

reat							3031				1316	6898	3622	1225			4609						
GSM5656457_treat	0.232119374	0.335717004	0.553455169	0.548279338	0.485654245	0.503837789	0.1275400542	0.613994885	0.294501228	0.525311891	0.446619102	0.25204755	0.307147273	0.784643699	0.311801257	0.573171621	0.219682442	0.633320677	0.347467647	0.391969264	0.463666051	0.286287995	0.361667002
GSM5656459_treat	0.271599247	0.436877123	0.498826497	0.5737825	0.488134494	0.48577763	0.117891611	0.647770279	0.273501718	0.58477609	0.478236492	0.262915934	0.400583839	0.757046536	0.327204749	0.580068733	0.369241189	0.649890137	0.410910168	0.424709292	0.428631086	0.28573403	0.479913362
GSM5656462_treat	0.212602361	0.419228687	0.541595076	0.543661729	0.490712474	0.458901912	0.2113123	0.673989987	0.383290444	0.550908978	0.538225661	0.278844543	0.398969189	0.749069403	0.309231411	0.570440081	0.321079226	0.643593318	0.416432855	0.417766713	0.450861307	0.290320129	0.424704037
GSM5656466_treat	0.311938559	0.46439025	0.560591116	0.541833536	0.446895168	0.465912255	0.279642438	0.691432268	0.330220082	0.601412607	0.498734335	0.286771546	0.413805451	0.691673745	0.335979048	0.548023319	0.18424217	0.628758895	0.376084146	0.398075518	0.460983352	0.275143629	0.518753536

GSM5656468_treat	0.233919854	0.349619981	0.537221704	0.534696916	0.486405737	0.468326943	0.267655872	0.603523862	0.360417615	0.551721089	0.44761026	0.254308041	0.398662566	0.743137702	0.329454212	0.536663631	0.300999948	0.644576306	0.326906172	0.391773506	0.444315895	0.29597966	0.418167175
GSM5656472_treat	0.651074535	0.684702132	0.656088806	0.718214809	0.536899886	0.506101511	0.302081606	0.692004003	0.558875973	0.687894839	0.822052652	0.443689484	0.45529623	0.774750446	0.428940854	0.64206265	0.583632725	0.778850956	0.555461553	0.490758206	0.536365525	0.389632805	0.578785935
GSM5656475_treat	0.51873375	0.558377709	0.658334983	0.622117822	0.533800655	0.412157531	0.359424241	0.711305946	0.509887158	0.656894583	0.676938338	0.303058639	0.530840971	0.723216727	0.374644116	0.659422098	0.32092742	0.699489144	0.544670748	0.53486547	0.483393681	0.285392387	0.560071566
GSM5656476_treat	0.418498434	0.548468157	0.647669283	0.671237144	0.516074642	0.501523018	0.428779633	0.699403471	0.537929516	0.623619797	0.735025727	0.431523509	0.568580342	0.765462661	0.404220113	0.616648713	0.580822489	0.699516389	0.59161597	0.483103047	0.518103813	0.379900304	0.553147773
GSM5656	0.451604	0.5070919	0.6248219	0.562894973	0.512385469	0.491835922	0.204	0.6701080	0.445681	0.548061002	0.477	0.256	0.338	0.738	0.3110569	0.579813	0.312	0.648144663	0.443686	0.4058359	0.4626684	0.3104890	0.5044261

479_treat	231	44	6				888494	35	133		183639	15056	943095	13823	65	142	630269		324	61	97	66	24
GSM5656484_treat	0.3130435556	0.384685602	0.57450351	0.562860882	0.466142884	0.505829877	0.164920109	0.614747669	0.427334303	0.551242656	0.53264022	0.268999063	0.336133806	0.781654847	0.296037275	0.506322929	0.362810456	0.633197116	0.383656294	0.402911896	0.445587872	0.256562384	0.332129561
GSM5656491_treat	0.513825571	0.577831683	0.569138529	0.629595892	0.502524504	0.489135478	0.232695912	0.619961784	0.499133666	0.619618174	0.609314517	0.317721925	0.374661306	0.724528892	0.329742335	0.614253246	0.384226223	0.689193675	0.467302464	0.440081003	0.460119796	0.314360196	0.58788596
GSM5656494_treat	0.385832381	0.461025205	0.593973402	0.53796907	0.48302762	0.453076808	0.317045774	0.633079307	0.420237597	0.533126093	0.433535655	0.242313576	0.351725024	0.739094777	0.314364322	0.572039434	0.306177567	0.624757587	0.364748888	0.416000454	0.461323877	0.272890959	0.450122221
GSM5656497_treat	0.394035492	0.50560085	0.565807104	0.614989803	0.498525211	0.459239049	0.4133584	0.711407206	0.410539688	0.650381057	0.6414791	0.3908738	0.5039922	0.7221204	0.375418772	0.643232258	0.2788288	0.664563324	0.532886133	0.488107077	0.477244677	0.323912631	0.550394947

							46				42	98	12	58			82						
GSM5656499_treat	0.46990847	0.607768518	0.543739153	0.573177428	0.506809458	0.458793971	0.234594799	0.700240867	0.460123041	0.593754142	0.536636825	0.300972977	0.360854064	0.735147132	0.334001312	0.606916485	0.232453386	0.679725817	0.489902959	0.452432299	0.438785194	0.312203927	0.572232081
GSM5656502_treat	0.246036892	0.453687194	0.514273241	0.541684446	0.472084434	0.465470404	0.149347008	0.654667498	0.341255364	0.576802882	0.486779556	0.273658951	0.309830534	0.720950905	0.301508692	0.567191665	0.439425069	0.662401132	0.415336811	0.38644695	0.417808404	0.299675329	0.459307157
GSM5656507_treat	0.373697275	0.511386777	0.633298277	0.563891216	0.522468825	0.44254136	0.286069004	0.650248417	0.482221855	0.583846776	0.435392887	0.27267946	0.420344607	0.715653708	0.332422206	0.647708349	0.313554667	0.669207635	0.438237321	0.429958202	0.476536365	0.308001001	0.570847021
GSM5656510_treat	0.426533852	0.669134423	0.551159073	0.583875628	0.488800435	0.428074022	0.424443552	0.718377555	0.426545685	0.592797473	0.600093122	0.312432185	0.492601478	0.695064651	0.344612513	0.585831174	0.425266067	0.633539024	0.516105781	0.436494183	0.475336354	0.295120506	0.590891995
GSM	0.29	0.494	0.612	0.6349	0.496368	0.48353	0.2	0.695	0.36	0.5765	0.6	0.3	0.4	0.7	0.330	0.60	0.4	0.66155	0.49	0.447	0.469	0.348	0.446

5656512_treat	9233189	814656	893166	03078	327	7131	00443846	561977	1868907	24885	18522655	45861348	44179007	92143786	446481	1195815	65120089	5914	8746629	82721	980567	383723	745924
GSM5656515_treat	0.483754727	0.686361592	0.632250257	0.596539595	0.502225202	0.434377685	0.314790322	0.694067003	0.545044421	0.627824806	0.611279673	0.336572258	0.449527503	0.722921272	0.351087038	0.66063047	0.252044505	0.659139345	0.513276291	0.464336534	0.481589694	0.326275651	0.614160841
GSM5656519_treat	0.225498494	0.460519809	0.555767444	0.57407989	0.485161189	0.464359044	0.309977913	0.66842654	0.297447006	0.587392485	0.527374211	0.281433588	0.483712914	0.729816492	0.345926833	0.597171152	0.376225207	0.654666778	0.455695792	0.438065662	0.443257267	0.243603165	0.462504284
GSM5656523_treat	0.171750628	0.556928773	0.448748463	0.582767346	0.496580586	0.465818383	0.248599747	0.679852405	0.308132261	0.590882335	0.475382148	0.278106762	0.3690878	0.738511167	0.325580112	0.568534081	0.237866307	0.636689782	0.388702517	0.401703958	0.404096413	0.305812296	0.51856611
GSM5656526_t	0.364137893	0.414765216	0.571488476	0.578090973	0.480356989	0.47820593	0.17431	0.665324131	0.453967849	0.611887976	0.47415	0.31406	0.38015	0.73895	0.367231146	0.608604404	0.27440	0.671813232	0.481357775	0.466195631	0.485223932	0.271701024	0.504631189

reat							4383				5558	5841	6718	788			2217						
GSM5656529_treat	0.310436246	0.494398907	0.492115754	0.563228019	0.511525895	0.480416838	0.242447615	0.662132024	0.425080764	0.608425788	0.526820004	0.281007758	0.283143787	0.707815268	0.312187033	0.554938223	0.266988473	0.631683825	0.431061293	0.40840978	0.434876737	0.316892668	0.460145328
GSM5656538_treat	0.217661756	0.474122961	0.566759534	0.546272102	0.472775686	0.466263417	0.171863856	0.628718213	0.359995	0.537208406	0.443742712	0.250027209	0.346360811	0.732610395	0.290825039	0.544805014	0.327388115	0.635321133	0.356078366	0.395227786	0.423887401	0.284559018	0.456373201
GSM5656540_treat	0.330963983	0.43518192	0.594529455	0.59093634	0.489314304	0.470333802	0.284683932	0.684167592	0.35234177	0.572331011	0.625059044	0.353546105	0.475026838	0.758153446	0.336227807	0.613212179	0.405400228	0.684843105	0.469723057	0.420352333	0.481120433	0.276421729	0.465700764
GSM5656544_treat	0.51088878	0.547378863	0.665899839	0.551497147	0.507691544	0.446521884	0.287775019	0.696749901	0.554859701	0.587225233	0.550433191	0.309161604	0.421260306	0.741367433	0.325111683	0.601799898	0.31333168	0.63144332	0.445128024	0.4525349	0.475022875	0.302755426	0.518385404

GSM5656547_treat	0.346950971	0.549487493	0.55709449	0.577421405	0.487137034	0.462495194	0.317961408	0.695141199	0.381641152	0.588819472	0.550979451	0.307521285	0.376247067	0.738418964	0.357667852	0.589908891	0.294909401	0.675109815	0.474689525	0.440367775	0.473042396	0.294509143	0.547254217
GSM5656550_treat	0.37550444	0.657472762	0.65989735	0.626663495	0.491300744	0.487045298	0.30978532	0.75973278	0.487050876	0.590964955	0.682378862	0.406090787	0.501770619	0.786056787	0.364558525	0.565713856	0.440892456	0.691256857	0.535845695	0.4613512	0.485738167	0.321688748	0.507595786
GSM5656552_treat	0.428347102	0.604340241	0.65092929	0.605922885	0.512042547	0.448572497	0.352296429	0.754870365	0.538832907	0.625098589	0.681508413	0.382456107	0.531638431	0.771975524	0.391069021	0.62723333	0.377084213	0.681547025	0.564504155	0.513187106	0.484222042	0.316751679	0.57068358
GSM5656553_treat	0.305993849	0.504705851	0.555321524	0.60514177	0.477220791	0.443520523	0.298597191	0.679783335	0.388854804	0.587739052	0.571587231	0.312256259	0.418747946	0.751671478	0.349461359	0.613558708	0.293744424	0.676129178	0.473378548	0.444434452	0.478563083	0.327092883	0.537353661
GSM5656	0.385714	0.3545880	0.5422921	0.535843514	0.495063499	0.465954953	0.297	0.6486562	0.402493	0.546855079	0.498	0.261	0.461	0.730	0.2907785	0.585793	0.209	0.633242091	0.425401	0.4039871	0.4518332	0.2737207	0.4052404

557_treat	76	07	8				32844	86	071		08301	596346	983382	821397	72	138	588241		228	55	03	33	26
GSM565660_treat	0.3123629971	0.554076124	0.51394268	0.556320664	0.453966097	0.437879867	0.260752834	0.700662466	0.413650131	0.578931524	0.479669925	0.269375197	0.389519143	0.709548567	0.327991894	0.558520509	0.255321235	0.644407753	0.428176073	0.432764897	0.421533796	0.316005996	0.54062758
GSM565661_treat	0.290526659	0.354094666	0.555571236	0.521938375	0.486510229	0.49116832	0.116845479	0.637706811	0.379314874	0.520259434	0.424669144	0.260567062	0.303637409	0.78579479	0.294288731	0.541951562	0.313208958	0.59906553	0.370038796	0.384335549	0.440624387	0.308608185	0.323990536
GSM565666_treat	0.429951261	0.582988412	0.62558038	0.572181588	0.508587608	0.434574587	0.362360489	0.744788495	0.539608988	0.611854962	0.596190492	0.345763429	0.491222116	0.735696061	0.37093546	0.602382601	0.281831176	0.660683518	0.530479184	0.498730192	0.450187641	0.300672883	0.565726718
GSM565668_treat	0.288773917	0.511713649	0.556035944	0.588666025	0.498127924	0.470260761	0.3590473	0.689456526	0.34689535	0.582965208	0.5695221	0.3100166	0.5213437	0.7650250	0.342948395	0.601065145	0.3964526	0.64657651	0.442120434	0.440627387	0.4701678	0.288940407	0.482733952

							73				45	12	67	07			42						
GSM5656572_treat	0.436951545	0.53534606	0.517609687	0.520347094	0.455618078	0.466074152	0.361831501	0.638914555	0.547329132	0.585919531	0.59012155	0.306709304	0.394539731	0.680352556	0.327998171	0.55254863	0.243427088	0.662206975	0.460825501	0.413057562	0.419351445	0.335873495	0.510431604
GSM5656574_treat	0.194171994	0.398013003	0.482632973	0.514816469	0.466308305	0.470212738	0.108051642	0.639252036	0.317322137	0.534722058	0.345482327	0.240959179	0.260717312	0.75471021	0.300116141	0.492887568	0.277989471	0.617170891	0.340931549	0.366996673	0.398744713	0.28404291	0.401502902
GSM5656577_treat	0.2775404444	0.484001446	0.404231467	0.516247396	0.433701047	0.505369376	0.247804641	0.650492553	0.345642103	0.544399736	0.435838124	0.224227293	0.257983646	0.648112167	0.294319296	0.478244947	0.199890591	0.675022116	0.42044454	0.35335106	0.367707612	0.341785334	0.464003886
GSM5656580_treat	0.367514338	0.617135575	0.641387554	0.586157084	0.50761847	0.448431834	0.293948278	0.755201759	0.45846268	0.572538779	0.599688157	0.340559704	0.417060805	0.722633117	0.312696759	0.594909256	0.395168043	0.674034675	0.502515969	0.427625804	0.443802087	0.341881648	0.544032894
GSM	0.47	0.639	0.613	0.6112	0.504687	0.47900	0.4	0.725	0.43	0.6214	0.6	0.3	0.4	0.7	0.371	0.61	0.4	0.71362	0.54	0.487	0.490	0.316	0.588

5656582_treat	0315645	668373	076528	03026	98	8359	09453837	179543	1660047	87973	63783907	63149052	84956823	52748434	697527	7859003	1269984	7834	746685	217422	102752	312453	824997
GSM5656586_treat	0.396686673	0.635505897	0.624644868	0.670987651	0.516104535	0.474541534	0.229292126	0.728350806	0.516495379	0.620617871	0.705185099	0.392070031	0.518358388	0.774289385	0.368959279	0.611584364	0.526832958	0.700196178	0.556611418	0.499352753	0.490755485	0.359770824	0.527826479
GSM5656587_treat	0.521330558	0.571075008	0.628761119	0.60606703	0.507034949	0.462604377	0.283840409	0.737147394	0.494777807	0.615788139	0.642806256	0.368348386	0.553486577	0.767531249	0.369471996	0.604318979	0.43431484	0.684076566	0.505295499	0.476765364	0.475622833	0.299476456	0.539028408
GSM5656592_treat	0.580079866	0.656414547	0.616173573	0.580639626	0.517666764	0.488223943	0.371881983	0.723320308	0.632401458	0.599793868	0.637072718	0.379844178	0.494782458	0.7371426	0.375571065	0.572919942	0.350314201	0.670950514	0.541906093	0.500546933	0.48915626	0.334596642	0.527733087
GSM5656593_t	0.400130867	0.508776718	0.596690657	0.663957913	0.50404102	0.516641639	0.37678	0.703118212	0.470660068	0.634836994	0.68845	0.34077	0.64188	0.79045	0.373201346	0.659382959	0.58886	0.682475308	0.550326821	0.470933189	0.502641942	0.271182819	0.478248306

reat							5203				5903	0743	8131	4008			7555						
GSM565598_treat	0.468838868	0.541659584	0.698944667	0.558084862	0.542860923	0.459100872	0.433863016	0.758899907	0.510248915	0.595769234	0.635287791	0.365753159	0.568533114	0.711324029	0.33278195	0.593480423	0.427639393	0.640949204	0.515313747	0.474758023	0.459526081	0.327515093	0.484056058
GSM565605_treat	0.291971276	0.393867973	0.622007269	0.566683361	0.486404368	0.471442599	0.128575765	0.605121338	0.319396998	0.535317658	0.453725508	0.268387669	0.324058358	0.774069596	0.320175442	0.56543314	0.339164481	0.629483248	0.370510237	0.402618564	0.455446968	0.316865684	0.397671467
GSM565607_treat	0.262050407	0.434020735	0.519678377	0.543394681	0.492387949	0.462986633	0.263337704	0.648060698	0.268120189	0.563411699	0.466143029	0.241085486	0.430705266	0.770515931	0.318259766	0.568776801	0.299742086	0.6480602	0.334914889	0.399769498	0.433757773	0.273850069	0.466777113
GSM565610_treat	0.270335721	0.306241172	0.522715806	0.575829596	0.481160359	0.488695389	0.205652925	0.647677809	0.353623345	0.582867261	0.519081309	0.284833876	0.506778102	0.773276042	0.356184975	0.62359418	0.31577166	0.657613077	0.412358776	0.421198811	0.454836897	0.247895188	0.401232948

GSM5656613_treat	0.32803642	0.523336939	0.725758566	0.699584628	0.498110858	0.502253829	0.276971733	0.705876692	0.405669954	0.624582706	0.749877984	0.388198511	0.479013988	0.828906451	0.368842128	0.620351664	0.484254063	0.689791447	0.533632575	0.520495133	0.532621873	0.30551255	0.457632663
GSM5656616_treat	0.31616811	0.394553329	0.559883219	0.537507569	0.495854303	0.480734869	0.205377746	0.665829755	0.376017962	0.547694487	0.450036812	0.24655198	0.386916169	0.766534341	0.331058448	0.579610387	0.286721512	0.634701483	0.393447643	0.389305967	0.450526078	0.261667882	0.45936508
GSM5656618_treat	0.177139897	0.306928867	0.47487793	0.531357674	0.468944213	0.472657454	0.165701007	0.62942391	0.254485444	0.534985944	0.394074181	0.239453947	0.319099763	0.77535752	0.32742264	0.566919236	0.26891811	0.618443863	0.321026418	0.385756743	0.426589426	0.264026808	0.381744482
GSM5656626_treat	0.496259004	0.452645961	0.578103183	0.568000455	0.504189256	0.504641165	0.249350007	0.624849478	0.483066932	0.54880402	0.478202222	0.25788497	0.328608388	0.791738021	0.324031713	0.582461875	0.282845809	0.650922831	0.39119848	0.410097936	0.453873913	0.264109902	0.4062319
GSM5656	0.193657	0.4193931	0.6615208	0.627080767	0.455112049	0.513166652	0.238	0.6295213	0.344745	0.540860596	0.561	0.279	0.364	0.784	0.3334659	0.577043	0.364	0.612322141	0.442045	0.4129873	0.4965385	0.3111226	0.4219597

628_treat	719	42	93				734142	96	871		878984	721849	066204	672406	82	81	187849		138	28	91	94	07
GSM5656630_treat	0.162205779	0.424468903	0.543426514	0.607538943	0.492009068	0.50376696	0.139314126	0.594806566	0.251385371	0.54476937	0.515245001	0.264672049	0.373396362	0.751562047	0.301926208	0.504237091	0.44153682	0.60938264	0.41346773	0.37833415	0.444201325	0.283257295	0.377239815
GSM5656633_treat	0.170961646	0.30545118	0.532815305	0.525294797	0.467912089	0.507774675	0.276938699	0.604654734	0.31000364	0.533177056	0.401084972	0.224198138	0.419019024	0.787699518	0.318289429	0.566278682	0.128992374	0.631315986	0.323679617	0.365969324	0.451968331	0.278423313	0.387553614
GSM5656636_treat	0.211155551	0.315546106	0.566330016	0.512227073	0.481437102	0.465634511	0.230365543	0.620020296	0.325419653	0.555947184	0.398260154	0.229183855	0.370891007	0.78420019	0.314788223	0.594877337	0.208518339	0.629722701	0.304810425	0.366586602	0.451709224	0.235964675	0.463157221
GSM5656638_treat	0.492892658	0.52976346	0.61133035	0.632786951	0.50896832	0.471978396	0.2616813	0.689964588	0.510843239	0.594925663	0.6341171	0.3498541	0.5018230	0.7798711	0.371468593	0.616909798	0.4053585	0.690528956	0.498347177	0.444504908	0.494511219	0.355447911	0.550097321

							61				76	22	15	52			73						
GSM5656640_treat	0.122133952	0.245347667	0.583458606	0.530908076	0.476675802	0.50593032	0.117609703	0.606692141	0.264556955	0.512729588	0.360373861	0.215881866	0.219446694	0.772021051	0.304071321	0.512167029	0.258789802	0.605318884	0.298953327	0.376023923	0.44183017	0.298308099	0.269094236
GSM5656642_treat	0.36455088	0.57241415	0.636018457	0.652008548	0.49894043	0.475110658	0.232034819	0.732233221	0.39573278	0.623719108	0.683133401	0.375005712	0.504751033	0.782382351	0.370937788	0.630352478	0.468419263	0.680019532	0.521621498	0.459331369	0.480287361	0.335918491	0.550357268
GSM5656644_treat	0.248494259	0.412461137	0.432159017	0.552485989	0.451732715	0.481097691	0.176411769	0.650357397	0.306494653	0.575130975	0.458334566	0.27390807	0.394243715	0.761673978	0.346349327	0.59388033	0.214878142	0.690386892	0.380907799	0.398546528	0.398948656	0.261082576	0.471497758
GSM5656646_treat	0.306063643	0.350317002	0.524586648	0.511012006	0.471098782	0.451807289	0.148935022	0.624940718	0.389509024	0.542913855	0.34831585	0.22010442	0.342832942	0.750744203	0.307611322	0.568051383	0.249645533	0.640231173	0.315607381	0.398017966	0.445517937	0.286044492	0.417877518
GSM	0.45	0.567	0.585	0.5082	0.490247	0.42708	0.2	0.696	0.54	0.5757	0.4	0.2	0.3	0.7	0.313	0.56	0.1	0.62807	0.38	0.416	0.465	0.285	0.506

5656650_treat	9156878	746767	124975	39884	008	725	40151552	054278	6711822	9828	42545635	53483143	27212709	22409104	197637	1466965	70154709	0304	9893743	012474	877008	421883	945903
GSM5656651_treat	0.243560623	0.271771295	0.524120714	0.551549136	0.463355139	0.464377603	0.168584568	0.64937558	0.330552068	0.551641515	0.386801819	0.268548325	0.375902029	0.751917972	0.306598255	0.589180717	0.26070426	0.622008177	0.363444221	0.395833219	0.45122605	0.295583665	0.425093754
GSM5656654_treat	0.25807934	0.366173009	0.458417556	0.529403136	0.452852454	0.487541769	0.163488215	0.624686021	0.32687686	0.525573669	0.350672636	0.220625491	0.272059135	0.755819594	0.31253424	0.550697317	0.151271542	0.580918859	0.312348673	0.366714539	0.427097452	0.256030648	0.413264199
GSM5656658_treat	0.187328006	0.405419848	0.59690325	0.559868327	0.484851931	0.502061034	0.175853294	0.659634324	0.347329217	0.525460721	0.451276967	0.249099349	0.335503989	0.786725173	0.30943752	0.563617677	0.288384099	0.624014259	0.356276384	0.379257705	0.448249791	0.280240711	0.409840268
GSM5656170_t	0.285447001	0.491596081	0.601328736	0.568106212	0.485819976	0.47586955	0.13655	0.675629848	0.309635276	0.535933837	0.38254	0.22458	0.29866	0.77417	0.312880433	0.547209966	0.28000	0.640035642	0.363405299	0.4153395	0.437761682	0.282651502	0.437306389

reat							0768				3313	5416	13	77			4324						
GSM5656172_treat	0.712963879	0.654137558	0.613892809	0.584870195	0.483236638	0.4596607	0.23748994	0.72370089	0.646227359	0.586767961	0.586207673	0.316178915	0.396448963	0.703910517	0.36184236	0.56217953	0.245492434	0.664135166	0.509930651	0.486976859	0.473968848	0.344125377	0.545138038
GSM5656176_treat	0.362865316	0.490882244	0.548163599	0.541739081	0.493988626	0.456005256	0.186942867	0.652427291	0.397359726	0.530905938	0.438121432	0.241910674	0.317980502	0.744539624	0.28009365	0.582464561	0.324588822	0.636185201	0.385271091	0.406040022	0.433251203	0.29433875	0.468711961
GSM5656178_treat	0.295789935	0.334979623	0.517895304	0.530237106	0.464669822	0.490008388	0.096890442	0.653676376	0.28913971	0.548652509	0.279462262	0.252636053	0.319123218	0.73912919	0.321194997	0.507170252	0.271238608	0.642091223	0.376540261	0.3798277	0.433918854	0.274342422	0.370965821
GSM5656181_treat	0.253286871	0.480998884	0.623458934	0.546495366	0.525118612	0.430131614	0.233283517	0.724582167	0.415478801	0.601557071	0.491899957	0.341708118	0.41134897	0.702181188	0.344446772	0.584400803	0.321494028	0.664782931	0.488957809	0.478613784	0.450771434	0.296198345	0.508757033

GSM5656188_treat	0.416355251	0.59831845	0.515765047	0.569755423	0.516991725	0.439231236	0.18332708	0.703006199	0.393764924	0.581405827	0.517752	0.303943403	0.440115629	0.719327432	0.312956409	0.555526087	0.409592366	0.664671451	0.428608509	0.424652765	0.438703037	0.348549217	0.545375885
GSM5656197_treat	0.121490265	0.503249015	0.549302144	0.486606524	0.494886117	0.472536511	0.175679762	0.641933317	0.356984596	0.52957146	0.320919035	0.262511061	0.273533241	0.696585996	0.284530852	0.485806516	0.283148433	0.614902399	0.41092378	0.374613483	0.422155923	0.305913164	0.459945361
GSM5656201_treat	0.238576264	0.402135306	0.567418569	0.605555335	0.479113025	0.499397349	0.098163861	0.662812831	0.273290096	0.560977188	0.427886186	0.269205945	0.343756242	0.764435536	0.332363037	0.537283429	0.367806103	0.636630989	0.410786766	0.426139339	0.435300712	0.30886148	0.438555887
GSM5656204_treat	0.349329865	0.458047427	0.52805258	0.568572674	0.474949738	0.479750228	0.159113013	0.668149415	0.422057123	0.570560686	0.419239595	0.260772587	0.324966044	0.720332586	0.298314568	0.571333681	0.310712977	0.658095325	0.40297489	0.424323561	0.431576134	0.300106172	0.451696859
GSM5656	0.214112	0.4594337	0.5500798	0.551438054	0.487554549	0.472615213	0.098	0.6586623	0.356936	0.547683452	0.387	0.253	0.332	0.738	0.3083762	0.530271	0.293	0.632231747	0.383378	0.4096212	0.4251647	0.2903401	0.4589716

206_treat	865	2	3				796706	61	7		043923	535007	477265	527739	56	465	843257		228	2	17	61	18
GSM5656213_treat	0.236657395	0.42009486	0.544529636	0.569989029	0.506008025	0.455076171	0.145616886	0.645166948	0.308372683	0.555124122	0.425040005	0.258378546	0.35655228	0.740082424	0.336549022	0.544918795	0.272849983	0.646920539	0.391390896	0.435354904	0.428189456	0.28647514	0.441501786
GSM5656216_treat	0.297758292	0.523321915	0.538857997	0.583553783	0.516618524	0.505401643	0.206957759	0.677636876	0.302836529	0.616937767	0.510902684	0.304068379	0.440126641	0.699859956	0.365540449	0.524385529	0.354736639	0.666251226	0.443868259	0.4246311196	0.430151482	0.325266312	0.512720824
GSM5656221_treat	0.263578805	0.620320623	0.557206656	0.631380741	0.496737289	0.449302528	0.301303912	0.724477578	0.433993538	0.64192718	0.651561987	0.394504539	0.582048522	0.720018647	0.336978417	0.535477077	0.679436858	0.711895422	0.506270157	0.453663155	0.454276958	0.363200543	0.526494941
GSM5656224_treat	0.274263661	0.46354187272	0.6101872	0.567249616	0.495219047	0.49854191	0.1506903	0.694134259	0.307953303	0.550235679	0.4499243	0.2483128	0.3526120	0.7244981	0.329171158	0.516884061	0.3189654	0.644268434	0.398512329	0.457992899	0.433668368	0.288216543	0.451781148

							99				45	59	49	98			75						
GSM5656225_treat	0.255684614	0.478338158	0.562413458	0.609734722	0.50173295	0.481717538	0.142522903	0.66615948	0.326645479	0.592781346	0.53755171	0.302179835	0.402185559	0.774736037	0.334357885	0.580030504	0.345357948	0.65371024	0.420969191	0.43578182	0.438673642	0.320762368	0.472125641
GSM5656229_treat	0.325352603	0.494097021	0.557795386	0.561997938	0.480811175	0.465464273	0.135323652	0.701929773	0.356432309	0.56261091	0.438516979	0.276437342	0.36039708	0.717997824	0.339072813	0.509195149	0.332381146	0.643914987	0.409531618	0.418513569	0.421577719	0.288157972	0.492015091
GSM5656233_treat	0.312203841	0.660572502	0.550546508	0.635367523	0.498316818	0.445486932	0.243595008	0.758458968	0.470110439	0.629533566	0.652408556	0.345981663	0.456752422	0.717214635	0.373096298	0.559463613	0.45322892	0.708145961	0.534056816	0.463849148	0.454235452	0.357439497	0.622854167
GSM5656242_treat	0.275135891	0.269469098	0.417208686	0.517392394	0.497464729	0.448666507	0.106822001	0.600420646	0.343533639	0.58187313	0.460835007	0.237085979	0.295749944	0.693527402	0.276920972	0.513279569	0.073085072	0.653798252	0.321058998	0.357095317	0.352833205	0.372537988	0.37520347
GSM	0.62	0.714	0.618	0.5974	0.494763	0.45187	0.2	0.710	0.68	0.6308	0.7	0.4	0.4	0.5	0.380	0.53	0.4	0.66582	0.58	0.518	0.469	0.365	0.595

5656248_treat	1531741	689202	952438	36477	36	7059	95977244	026009	3158939	39885	00726818	18843275	32054604	73941378	232562	2802854	89302071	5394	323159	276244	599002	088973	671326
GSM5656250_treat	0.321772848	0.577759144	0.615119858	0.588623145	0.513020818	0.456922218	0.172580568	0.723714984	0.368999355	0.574719302	0.534234484	0.305516721	0.411940496	0.698368866	0.35955567	0.562974284	0.348637401	0.651691917	0.472938193	0.453861831	0.43993282	0.28872139	0.53811656
GSM5656252_treat	0.261447813	0.546572168	0.550783873	0.494639622	0.501200037	0.44277435	0.142372362	0.700455974	0.303627437	0.537605684	0.307857602	0.255945502	0.275547128	0.693074157	0.307893181	0.503125395	0.269228818	0.619824395	0.377274137	0.419209481	0.396379211	0.320020028	0.508923472
GSM5656257_treat	0.344687475	0.523356514	0.669363049	0.604671027	0.563682147	0.479595302	0.27931964	0.733834185	0.317733586	0.618752126	0.616139011	0.343329611	0.487387498	0.740218968	0.351349955	0.549018452	0.326073328	0.660738733	0.5169453	0.484115054	0.464613574	0.33308203	0.508207399
GSM5656260_t	0.282906512	0.529592864	0.563945405	0.506592464	0.512668745	0.445264569	0.18935	0.681935556	0.329001574	0.567073405	0.38482	0.25639	0.37427	0.68948	0.316612954	0.547793227	0.26492	0.616645393	0.373869231	0.440657152	0.427481517	0.279299204	0.514344728

reat							4958				0401	9308	9362	2467			4363						
GSM5656261_treat	0.352317506	0.523992947	0.500559243	0.584063739	0.48411553	0.469066581	0.109688088	0.657564578	0.358751016	0.54831075	0.436566806	0.264168451	0.333187144	0.736586927	0.32937178	0.523424648	0.327169314	0.630766191	0.410795555	0.403066429	0.417926404	0.323890962	0.478723031
GSM5656266_treat	0.36033935	0.420847956	0.613573572	0.558860838	0.485125716	0.500435358	0.142049419	0.639871813	0.424907162	0.564137031	0.407719968	0.225697145	0.319052418	0.699193606	0.340963666	0.476661153	0.333861156	0.603331238	0.394570505	0.43717373	0.430453661	0.293796774	0.392814839
GSM5656269_treat	0.225781097	0.378758462	0.482675251	0.547694681	0.494983163	0.464107862	0.099466713	0.6524445	0.349869406	0.562420312	0.418378732	0.264911668	0.348591648	0.724399318	0.342415882	0.537228652	0.268216636	0.66462853	0.388894318	0.41412787	0.432826661	0.264662952	0.427235459
GSM5656276_treat	0.407216994	0.658117351	0.580880348	0.633712121	0.515597613	0.458133108	0.231991143	0.749461811	0.449062528	0.620972379	0.602623855	0.352529196	0.447419408	0.730679028	0.32212826	0.602684343	0.3587027	0.679751841	0.465374509	0.445218559	0.475324381	0.340321297	0.618378147

GSM5656284_treat	0.358227089	0.423498498	0.505403697	0.565763482	0.511414125	0.45866924	0.162198286	0.695824919	0.33649841	0.56390856	0.450003949	0.289255242	0.435811984	0.752514267	0.323931221	0.581173554	0.258813121	0.651398031	0.449260937	0.439301246	0.452017681	0.27637194	0.439719854
GSM5656290_treat	0.302282859	0.542984975	0.44215794	0.627969635	0.487685141	0.510090927	0.076437234	0.630501064	0.38552527	0.608161976	0.493645011	0.315597463	0.360237482	0.72492233	0.350370656	0.523025344	0.402221681	0.685515847	0.413127669	0.398501037	0.422419056	0.368602555	0.54772181
GSM5656295_treat	0.242695901	0.407036567	0.482773444	0.574202484	0.492321824	0.472974919	0.103415793	0.675606719	0.302618914	0.568549603	0.404890859	0.270982607	0.410532229	0.765645256	0.348580567	0.543038973	0.304985199	0.649141475	0.435994241	0.441945362	0.451268458	0.268941055	0.462283227
GSM5656298_treat	0.364612709	0.667602469	0.613064849	0.574986504	0.500020629	0.445066313	0.283071434	0.715749799	0.425784104	0.616724739	0.577159314	0.326706615	0.459656861	0.673766983	0.369548157	0.528653697	0.378134123	0.651785702	0.507505818	0.468808937	0.460368905	0.340940993	0.567432149
GSM5656	0.366361	0.5362592	0.5722566	0.538627375	0.504844349	0.422696234	0.203	0.7098021	0.380889	0.575587473	0.416	0.277	0.358	0.726	0.3111341	0.593559	0.258	0.643998449	0.450811	0.4438184	0.4490867	0.2869363	0.5265717

302_treat	021	34	64				347792	22	145		463752	429978	605566	412347	44	38	534647		458	11	03	84	57
GSM5656306_treat	0.21142833	0.440344175	0.477466642	0.559210912	0.508040151	0.47602585	0.105327864	0.682508708	0.2220670062	0.564709254	0.417466325	0.276093756	0.332455379	0.697679409	0.331211337	0.523909384	0.280703694	0.623693341	0.393939459	0.367238833	0.39062374	0.307282093	0.497217316
GSM5656312_treat	0.322398164	0.583935843	0.447595761	0.508136307	0.467774203	0.445430671	0.27040675	0.711722528	0.40977434	0.578886134	0.515482433	0.278442741	0.316763733	0.603808479	0.343631126	0.504664511	0.323232135	0.625979405	0.473284316	0.395405922	0.38014829	0.372903653	0.52521265
GSM5656315_treat	0.214972873	0.477422916	0.592342215	0.601651932	0.496552501	0.465967138	0.193880683	0.698951579	0.296585518	0.579891234	0.471831476	0.294970368	0.399386672	0.768128339	0.343214087	0.582558172	0.3844951	0.645816243	0.462247825	0.454189421	0.460664356	0.323793511	0.502616868
GSM5656317_treat	0.280329272	0.591104473	0.602351258	0.461755213	0.54659193	0.416591848	0.3178672	0.7124338	0.391266792	0.570838173	0.4371100	0.3058171	0.3348966	0.6613475	0.352428818	0.564654803	0.2895749	0.631961461	0.483641594	0.446858453	0.46283338	0.331642257	0.533698088

							69				31	22	95	44			41						
GSM5656319_treat	0.237473436	0.559057098	0.615122433	0.570977843	0.512178706	0.47262533	0.183029209	0.715681976	0.377542851	0.554915872	0.493590095	0.280859258	0.377211102	0.768928523	0.326810221	0.568159369	0.259173949	0.6472843	0.448887507	0.429897851	0.432743129	0.384035387	0.528672824
GSM5656322_treat	0.292769175	0.648957159	0.607959702	0.624160527	0.521148416	0.480330964	0.172746561	0.707401999	0.333711228	0.605007789	0.606882288	0.321871165	0.428651814	0.766134058	0.338542567	0.587043812	0.438511483	0.689029064	0.447675845	0.436142197	0.463463472	0.391001731	0.585746824
GSM5656330_treat	0.668344399	0.620447876	0.668120665	0.574062195	0.513651979	0.476806908	0.235318214	0.722058389	0.670432469	0.559369068	0.569303477	0.298010733	0.372480448	0.770814995	0.335689534	0.578000999	0.279354213	0.662638419	0.461814962	0.490893349	0.468043817	0.320780966	0.499900935
GSM5656333_treat	0.257343046	0.42635087	0.525369078	0.594116295	0.504011356	0.477044484	0.171623189	0.693816057	0.314876999	0.550128296	0.477512135	0.271445916	0.341085045	0.738231312	0.314133582	0.53651373	0.242067601	0.647617012	0.441324559	0.42645862	0.417871531	0.321626747	0.415223683
GSM	0.59	0.605	0.561	0.5707	0.503576	0.44395	0.2	0.718	0.68	0.5785	0.5	0.3	0.3	0.7	0.327	0.57	0.3	0.63635	0.46	0.478	0.467	0.352	0.546

5656337_treat	3627837	493742	458391	358	13	549	93074743	689723	0641608	27031	59874524	17259572	9455897	12805083	127252	502122	0443805	5092	6784501	707905	470209	351537	703773
GSM5656339_treat	0.423393533	0.566968573	0.622155251	0.552905017	0.498967865	0.451234408	0.213109353	0.758867348	0.457292452	0.576981133	0.512487004	0.298915165	0.369209618	0.726945608	0.311783163	0.571754114	0.296466893	0.639644663	0.460867866	0.443699377	0.444655912	0.324500394	0.525002117
GSM5656341_treat	0.352606564	0.439011334	0.556041516	0.544008787	0.499088085	0.462698154	0.10664996	0.681373835	0.352552704	0.56355521	0.383870296	0.26853708	0.386804612	0.753763625	0.322125596	0.536303866	0.247046759	0.640342395	0.376750724	0.418397039	0.443120723	0.280695194	0.424143178
GSM5656344_treat	0.250451871	0.426263228	0.568800777	0.581135265	0.510682849	0.493901797	0.182231722	0.684010499	0.411716372	0.562735907	0.459372208	0.306742645	0.364712836	0.75114912	0.316976656	0.568931415	0.389664155	0.64127338	0.418542981	0.416858596	0.44443921	0.285735946	0.426797213
GSM5656347_t	0.511642115	0.595720878	0.55905752	0.583729576	0.502813217	0.452663646	0.226421	0.708172003	0.535577851	0.594496348	0.55770	0.34475	0.43612	0.71316	0.317286293	0.588274477	0.39420	0.660104871	0.455599472	0.460233887	0.455152702	0.324335625	0.539784325

reat							8393				5	9521	3444	3794			5556						
GSM5656352_treat	0.280875711	0.447012774	0.575306233	0.602040711	0.496834915	0.459085596	0.100237552	0.685000332	0.363753741	0.559497787	0.462033069	0.280349237	0.391106035	0.771089408	0.327319539	0.568821424	0.311960404	0.63992262	0.425705956	0.427695822	0.450599749	0.274478943	0.475159832
GSM5656356_treat	0.613585416	0.574903825	0.695134422	0.633315836	0.515027929	0.496793847	0.214513729	0.694931722	0.540228608	0.561151264	0.64248949	0.313815741	0.373661695	0.784973252	0.356186587	0.582274063	0.360910555	0.660170901	0.49121668	0.496791525	0.507076826	0.367908477	0.477923061
GSM5656358_treat	0.277720238	0.482946837	0.567858412	0.528610968	0.529823548	0.4385349	0.190031537	0.696522156	0.328783802	0.558570011	0.425389704	0.278050819	0.384055741	0.719971009	0.34082006	0.563500928	0.230865426	0.655369245	0.429424685	0.453004194	0.445431674	0.269921891	0.469484489
GSM5656360_treat	0.308431208	0.576359106	0.664892518	0.605931817	0.517682671	0.47243424	0.149746323	0.680526823	0.378234845	0.532401002	0.493358391	0.280725738	0.36222303	0.75596537	0.31431003	0.571471426	0.210833512	0.646325462	0.417695349	0.43807631	0.472497712	0.304054231	0.49881784

GSM5656364_treat	0.339652863	0.502791725	0.55602285	0.564169935	0.491718991	0.446557988	0.150138477	0.666410714	0.365258257	0.567646927	0.426005464	0.271916362	0.334210933	0.736430172	0.315160332	0.552593485	0.300628808	0.663198158	0.424213593	0.415790212	0.436954913	0.277348107	0.453170262
GSM5656369_treat	0.455130652	0.614182351	0.612486949	0.639118956	0.51540867	0.47788298	0.234351558	0.6913739	0.542385784	0.594352221	0.667991282	0.365135057	0.472272967	0.769916805	0.34557843	0.620610288	0.44245192	0.701432762	0.504688068	0.470137323	0.471572124	0.362178657	0.576920503
GSM5656376_treat	0.413081736	0.452660233	0.61668061	0.563750745	0.469124183	0.500471994	0.144851933	0.706650147	0.392964387	0.583553134	0.461751557	0.341674686	0.395292587	0.744263085	0.345665583	0.561959187	0.27770587	0.658404165	0.480868564	0.45134845	0.454404723	0.289486082	0.408116596
GSM5656383_treat	0.373115633	0.521653832	0.622598973	0.577761118	0.485306764	0.464048158	0.172091594	0.687338057	0.455025978	0.567836495	0.462593003	0.301479298	0.376653291	0.749538118	0.328193575	0.569158447	0.319755954	0.656908321	0.432317297	0.439088779	0.450909388	0.320354702	0.506396927
GSM5656	0.238835	0.4504582	0.5079851	0.511839477	0.491945388	0.500511095	0.125	0.6522688	0.436923	0.557683163	0.378	0.245	0.341	0.690	0.2923173	0.441283	0.285	0.609350802	0.377017	0.3921538	0.4196924	0.3797093	0.4414286

385_treat	602	32	96				727584	41	552		752289	763261	493494	251427	83	884	896729		419	52	5	76	73
GSM5656389_treat	0.25871328	0.445664344	0.579341855	0.59452955	0.489464172	0.487579792	0.215935221	0.675251747	0.39207103	0.546138669	0.4606903	0.279117136	0.383054791	0.759502925	0.322320888	0.546655623	0.311023316	0.642960701	0.433169857	0.453289207	0.466189474	0.338653327	0.445587652
GSM5656394_treat	0.187482552	0.469465775	0.564172685	0.495390182	0.487201804	0.460852503	0.171032312	0.666849542	0.427361514	0.54521521	0.356365869	0.257215	0.314087998	0.703540374	0.309013897	0.497176754	0.294176778	0.623140924	0.393794341	0.390189924	0.42935292	0.330208992	0.46211008
GSM5656396_treat	0.430520907	0.64196325	0.563613323	0.568526166	0.508714538	0.416577065	0.258791278	0.697374367	0.466127861	0.603567367	0.55838663	0.31642116	0.418291442	0.692573207	0.376405467	0.59898198	0.318630899	0.667860556	0.500342448	0.464570005	0.441900188	0.309739688	0.563204245
GSM5656398_treat	0.201973049	0.454268058	0.612760947	0.546029244	0.470640353	0.517993468	0.1273437	0.604655881	0.381686925	0.525900287	0.4651198	0.2620246	0.3363106	0.7243284	0.311746338	0.487811755	0.3433951	0.62857142	0.366989715	0.394968254	0.459526271	0.297853165	0.416148362

							81				08	26	11	85			31						
GSM5656405_treat	0.271007254	0.504511682	0.552154863	0.537019082	0.507788214	0.442233652	0.252183138	0.670548385	0.323943214	0.579879575	0.488129911	0.279869257	0.385812826	0.703790291	0.34355736	0.547142141	0.233782228	0.648209052	0.40132557	0.42200414	0.427791245	0.289129875	0.494757766
GSM5656410_treat	0.319111032	0.532183011	0.505525254	0.54222455	0.470930459	0.433402539	0.231284279	0.711349567	0.383705575	0.574702771	0.458518942	0.281975886	0.356521133	0.670799124	0.337058268	0.512540284	0.319848141	0.65142316	0.446758453	0.426570303	0.409223182	0.293991413	0.505410434
GSM5656414_treat	0.23740794	0.572942198	0.567082439	0.542654021	0.495658675	0.473193737	0.216462103	0.727735131	0.423620927	0.573459283	0.531946351	0.306108295	0.383591632	0.707734832	0.321428833	0.522047959	0.357822528	0.630789436	0.480886145	0.446720758	0.42901624	0.331776722	0.480340672
GSM5656417_treat	0.205188734	0.452591519	0.553779119	0.519399487	0.483839941	0.462910883	0.109339117	0.665653981	0.311226248	0.536341387	0.372885877	0.249072226	0.296555984	0.727901762	0.2961089	0.484009637	0.254475143	0.63179183	0.33797604	0.385672426	0.415549192	0.304905348	0.433649782
GSM	0.17	0.417	0.487	0.4904	0.475389	0.46027	0.1	0.655	0.30	0.5268	0.2	0.2	0.3	0.6	0.299	0.48	0.2	0.61753	0.36	0.377	0.399	0.328	0.417

5656420_treat	9792999	540995	666869	72881	381	0958	25884522	657001	1116641	35053	89889895	18695791	01669113	87749384	527072	4403197	50287346	8239	3758731	055344	455155	191984	069992
GSM5656423_treat	0.312977883	0.599877422	0.62972507	0.594077779	0.490426001	0.477785826	0.248489489	0.73623175	0.360844538	0.580090545	0.565878367	0.31218673	0.371772614	0.731810858	0.335285509	0.563680749	0.398678098	0.651627607	0.51977724	0.462748961	0.456485421	0.325990151	0.508468666
GSM5656425_treat	0.296892966	0.44511859	0.501649488	0.570047579	0.478186259	0.436526972	0.155560727	0.693761967	0.391381364	0.551477441	0.453190512	0.273297064	0.325734925	0.734131712	0.32528776	0.525476107	0.245031805	0.659957635	0.404309214	0.427379657	0.43732943	0.270956169	0.434210806
GSM5656428_treat	0.369187631	0.407972857	0.591166003	0.542214998	0.506380347	0.47010097	0.151234711	0.670937139	0.416985916	0.552617144	0.407553158	0.288405271	0.31105978	0.741757848	0.316517241	0.56092129	0.238565917	0.639655354	0.413666601	0.419107402	0.438220714	0.271311323	0.454727125
GSM5656430_t	0.327238117	0.46250221	0.615701394	0.593593768	0.48972309	0.46534566	0.25488	0.681395122	0.418791481	0.582531417	0.55588	0.29329	0.41233	0.77065	0.334312144	0.608548479	0.32467	0.657830557	0.468421554	0.442704669	0.460095892	0.268292329	0.474777162

reat							9722				7462	2042	1756	4151			074						
GSM5656437_treat	0.494156416	0.510088446	0.556815568	0.551221293	0.500490154	0.440612783	0.151026418	0.703836478	0.472375708	0.563918633	0.439406599	0.282746942	0.347914614	0.730731947	0.319284877	0.541440649	0.286929284	0.641354383	0.425050876	0.439539469	0.429719233	0.329602073	0.430285981
GSM5656441_treat	0.284693852	0.447130305	0.532270129	0.568766759	0.488399688	0.45671636	0.165479585	0.720664488	0.378339238	0.585237402	0.493604492	0.295873099	0.347399378	0.71676266	0.332703581	0.542815373	0.335300804	0.66359553	0.468684832	0.445645298	0.457717088	0.280584445	0.443533012
GSM5656445_treat	0.270202216	0.47876082	0.526894129	0.508575411	0.47944222	0.443743522	0.149622783	0.686502522	0.33009412	0.559591275	0.369768915	0.271425337	0.315465157	0.675118651	0.303993084	0.514842754	0.155450462	0.615542476	0.349946668	0.415606422	0.420500083	0.298354043	0.477733169
GSM5656448_treat	0.276274908	0.455166841	0.536227775	0.532712564	0.478877958	0.449429481	0.184830482	0.675492905	0.333590711	0.544444539	0.369081547	0.254167224	0.352410888	0.732912861	0.314979094	0.517416573	0.276731065	0.652547376	0.311465001	0.401912255	0.406479849	0.307169898	0.455591812

GSM5656450_treat	0.230543881	0.363052548	0.562158093	0.521350643	0.47159055	0.472445572	0.158673441	0.670263151	0.29068679	0.559016183	0.33231699	0.225055465	0.270215011	0.721903981	0.288519769	0.524278472	0.273000068	0.652748264	0.360300615	0.39276177	0.423292025	0.277813088	0.414659105
GSM5656455_treat	0.391241353	0.502902067	0.524942006	0.627716457	0.494849219	0.487996527	0.238370169	0.663968764	0.434540138	0.574180033	0.569648172	0.339793936	0.371853785	0.746388174	0.321933276	0.525513398	0.523160339	0.645301956	0.463157593	0.420576979	0.442872574	0.365283216	0.511622551
GSM5656463_treat	0.257475964	0.530553604	0.583911602	0.598388799	0.498249402	0.470866095	0.19346871	0.684353289	0.381841189	0.5771342	0.553036658	0.294619798	0.397768593	0.751770534	0.32295415	0.54684838	0.352314224	0.660646204	0.431059335	0.399860812	0.444903591	0.356005344	0.506854228
GSM5656470_treat	0.163972138	0.35594368	0.464512563	0.470264289	0.473933106	0.475429045	0.099259599	0.601503718	0.375136068	0.53567033	0.251434548	0.227815194	0.226833605	0.69294661	0.289357976	0.433366157	0.245377866	0.613430086	0.347330458	0.350603682	0.388317035	0.268532656	0.391368262
GSM5656	0.252813	0.4696398	0.4921850	0.524317144	0.481188683	0.446422959	0.143	0.6755253	0.319165	0.568161656	0.397	0.257	0.335	0.681	0.3380012	0.541807	0.260	0.65538969	0.364576	0.4193154	0.4239579	0.2862785	0.4751005

471_treat	745	38	83				133283	73	105		281815	193897	038461	731666	18	827	389527		584	31	9	17	99
GSM5656474_treat	0.187860729	0.449139846	0.535922944	0.50115674	0.482869944	0.461975526	0.189550002	0.64444546	0.320733665	0.544308032	0.316324567	0.225877048	0.321712837	0.706443269	0.302192725	0.496882532	0.310082403	0.606828377	0.363602848	0.41105926	0.411961961	0.328066729	0.411635432
GSM5656477_treat	0.331270569	0.50645124	0.500707653	0.475607214	0.458915467	0.45404594	0.171237273	0.653203354	0.406800217	0.576536687	0.315479985	0.242145285	0.273394564	0.684733269	0.296511196	0.501335979	0.290035384	0.615959696	0.35407746	0.384846294	0.412568394	0.334488667	0.539191314
GSM5656480_treat	0.196509876	0.430237582	0.48683753	0.492990876	0.454307022	0.474047013	0.105766731	0.660682119	0.261721468	0.562362596	0.335822543	0.208007504	0.28238576	0.684731646	0.282344629	0.528991537	0.215306371	0.633779012	0.366443811	0.361593822	0.408165006	0.339560935	0.508084813
GSM5656482_treat	0.201821001	0.355686854	0.5236151	0.555376501	0.468228216	0.482539182	0.0478846	0.632513291	0.227297816	0.535376682	0.3191448	0.2296822	0.2437903	0.7509098	0.277266128	0.492743346	0.2876535	0.6225421	0.30647906	0.364426018	0.405179645	0.302169048	0.403788969

							89				28	67	54	86			73						
GSM5656485_treat	0.186145736	0.392854895	0.525977453	0.503930675	0.486190262	0.488585784	0.180958607	0.638922465	0.375800654	0.534579898	0.368758582	0.239936074	0.314135404	0.715826313	0.317401224	0.474982027	0.322630733	0.617936686	0.361562736	0.376941441	0.416488124	0.287598278	0.415279891
GSM5656488_treat	0.340565865	0.485706311	0.446647059	0.499123725	0.478123216	0.516000598	0.119902601	0.640853549	0.416300428	0.560254727	0.385440656	0.235795535	0.305660355	0.67743097	0.284960518	0.526664677	0.157595206	0.62779599	0.356343512	0.344678108	0.381022056	0.365843299	0.489015971
GSM5656489_treat	0.251301714	0.528194834	0.552720448	0.530752805	0.479430052	0.444287228	0.176731678	0.691554834	0.350060517	0.576083653	0.460212195	0.300161616	0.358445627	0.678781125	0.336919847	0.537787584	0.355010533	0.646214805	0.408475904	0.417708919	0.436213527	0.310016989	0.492544047
GSM5656492_treat	0.27675557	0.467158597	0.620774791	0.575961806	0.501370295	0.472264113	0.09714922	0.685407049	0.323692154	0.554422041	0.419282596	0.251265319	0.331078584	0.751843173	0.324990675	0.546127976	0.268071744	0.668965033	0.399750293	0.418294267	0.437521393	0.286632686	0.437591082
GSM	0.29	0.573	0.576	0.5652	0.497366	0.45748	0.1	0.686	0.39	0.5457	0.4	0.2	0.3	0.7	0.299	0.52	0.3	0.63108	0.43	0.425	0.430	0.325	0.494

5656496_treat	5711317	772474	408777	52381	062	0115	94916635	721964	0983169	47771	62676639	71134396	43247248	45176011	010709	3849896	50530947	016	0565044	73247	441662	380055	290294
GSM5656500_treat	0.53492626266	0.566374655	0.591611233	0.537807989	0.509046185	0.446671783	0.283331325	0.673262634	0.670444766	0.572088153	0.521164156	0.29315674	0.336529986	0.718102102	0.313920809	0.548686579	0.347707944	0.619072307	0.437647653	0.435258032	0.45952962	0.300605387	0.473056387
GSM5656503_treat	0.54052334	0.718388012	0.541899006	0.582959232	0.521945991	0.437848529	0.275296888	0.716482736	0.647749827	0.632087819	0.609548472	0.319441331	0.392700242	0.706057455	0.335995708	0.581871852	0.412982871	0.679453192	0.468549773	0.440950107	0.467983739	0.360284767	0.652908437
GSM5656506_treat	0.395339174	0.544095366	0.580659985	0.523885621	0.49539337	0.488325966	0.205831848	0.666941821	0.512042685	0.545392716	0.394322875	0.239896476	0.303536594	0.678775334	0.301097476	0.498777495	0.298469766	0.628623751	0.389295941	0.40032127	0.436267227	0.300022196	0.497479513
GSM5656508_t	0.285962734	0.463421692	0.523439766	0.587037794	0.509034816	0.478624681	0.13933	0.661200433	0.277972709	0.562313161	0.45435	0.26410	0.36359	0.77813	0.327867592	0.562658978	0.28760	0.670128533	0.383815576	0.427185293	0.445097965	0.298583892	0.475702837

reat							7909				115	0836	754	3048			3789						
GSM565514_treat	0.299788131	0.573923191	0.552505755	0.485904266	0.520438485	0.409722948	0.180603868	0.684504113	0.388648756	0.552196441	0.401412363	0.294718882	0.317943353	0.667640869	0.297400954	0.538562387	0.283665852	0.622234537	0.398231113	0.396624517	0.421636807	0.338050759	0.546792993
GSM565518_treat	0.365204432	0.484512013	0.536870479	0.497833261	0.493896317	0.458891874	0.15392504	0.641733757	0.389744774	0.538125144	0.316291502	0.228421823	0.349482916	0.70225739	0.304272039	0.518157441	0.155827496	0.616709315	0.350267135	0.386176741	0.414844998	0.267327968	0.444522705
GSM565530_treat	0.164705143	0.340064067	0.46766537	0.494384021	0.493020169	0.469853653	0.09222498	0.634302655	0.275767888	0.549620544	0.256030185	0.229066392	0.299545008	0.699317425	0.291503862	0.491002182	0.236379672	0.607619384	0.323129391	0.365754693	0.395155061	0.296124667	0.391109919
GSM565532_treat	0.573423519	0.604182646	0.655480811	0.628628298	0.503994455	0.521665671	0.210735854	0.660052912	0.633275117	0.592464178	0.711469149	0.351140442	0.399571207	0.72518953	0.387710629	0.613253804	0.338370942	0.667525408	0.476825393	0.456302974	0.465246727	0.350450459	0.442778016

GSM5656534_treat	0.220633043	0.50655396	0.594108326	0.555933184	0.486511987	0.477429947	0.125665548	0.643912204	0.381830494	0.55497718	0.41873932	0.24795509	0.324251061	0.751466204	0.310797028	0.537063016	0.270829001	0.640819085	0.374269744	0.403683479	0.427983437	0.290318051	0.490166787
GSM5656537_treat	0.133371492	0.454633719	0.559714803	0.510901223	0.469735563	0.486137761	0.148373242	0.630517425	0.330994702	0.51467256	0.31717139	0.232495567	0.263819694	0.70487563	0.270703928	0.437431614	0.291216587	0.59862624	0.349715014	0.366137428	0.395016317	0.299894597	0.417876231
GSM5656542_treat	0.383377665	0.521787225	0.593913977	0.537970231	0.511791788	0.449730958	0.209803251	0.704994906	0.429117111	0.555161414	0.446839922	0.29332371	0.354951872	0.711664187	0.330780577	0.569901912	0.281240819	0.641064975	0.464430287	0.43948053	0.455860954	0.312968212	0.49814884
GSM5656546_treat	0.341588861	0.493488729	0.556388035	0.547368805	0.491642555	0.466918417	0.130182146	0.670677469	0.40971825	0.54228675	0.426335295	0.260823568	0.313333662	0.740583438	0.299504122	0.516209378	0.314781386	0.631692138	0.368328313	0.429312523	0.432723324	0.293885857	0.462155301
GSM5656	0.310828	0.4803370	0.5828534	0.56102139	0.488618229	0.464215793	0.188	0.6991758	0.304469	0.579151147	0.475	0.287	0.338	0.768	0.3412798	0.558988	0.245	0.679178759	0.429638	0.4263530	0.4522431	0.2575059	0.4447986

548_treat	716	22	46				788692	33	902		990319	283645	770988	514429	85	07	79805		782	49	61	67	78
GSM56565555_treat	0.22052817	0.396918393	0.46811242	0.511380292	0.45060231	0.462292843	0.158185065	0.658500702	0.294182387	0.558808591	0.301504841	0.229587757	0.303285388	0.706341305	0.290044161	0.507460776	0.267000076	0.634572055	0.319569845	0.397451586	0.4220869	0.281435706	0.452974504
GSM56565558_treat	0.28574684	0.359812889	0.450543375	0.464077321	0.491389914	0.436251892	0.113195023	0.625646737	0.335239817	0.556533683	0.354146745	0.206564059	0.289991269	0.618771289	0.267056016	0.526089413	0.026564412	0.636151992	0.349770206	0.340689091	0.364249248	0.362707542	0.447422628
GSM56565562_treat	0.359343517	0.413470282	0.547650927	0.59297212	0.488707676	0.490182545	0.18533986	0.660224934	0.451529907	0.566650826	0.496677068	0.275886385	0.385024706	0.776035052	0.319015119	0.565318992	0.367771662	0.624556678	0.412681453	0.442887231	0.446461287	0.322849158	0.450934487
GSM56565564_treat	0.235770771	0.376885173	0.612596856	0.577493945	0.513002517	0.508905463	0.1104876	0.628290748	0.275421889	0.510762075	0.3969664	0.2379042	0.2527281	0.7693406	0.313681457	0.521765998	0.2946552	0.610516025	0.349590923	0.399091366	0.441480936	0.324027841	0.335064979

							41				8	66	25	35			49						
GSM5656570_treat	0.33919243	0.3979735	0.533457738	0.583000044	0.469689057	0.455635143	0.120748828	0.678584934	0.383039233	0.54969098	0.458387565	0.292875613	0.330331215	0.746834823	0.320393654	0.545080532	0.255850676	0.627232775	0.406915172	0.418768868	0.430017846	0.254111264	0.427412653
GSM5656575_treat	0.264884201	0.493413035	0.533631955	0.58559737	0.490910544	0.4575116	0.173971793	0.718565997	0.359284442	0.565837421	0.494451914	0.309623799	0.374861622	0.740492088	0.320340964	0.563876139	0.329460634	0.663271316	0.461970887	0.455686832	0.450463449	0.353094987	0.502710037
GSM5656579_treat	0.449833506	0.667178274	0.589674868	0.606641228	0.515488018	0.446844188	0.267002119	0.728419844	0.558042115	0.623506336	0.620367888	0.336954807	0.5163635	0.72558578	0.333035522	0.590947096	0.476899203	0.672729185	0.494155596	0.459698807	0.479130268	0.377608614	0.562317786
GSM5656584_treat	0.308488551	0.521540822	0.49330373	0.529310166	0.462389634	0.465536972	0.188299146	0.651390419	0.333637608	0.562306113	0.454237448	0.243839834	0.311924565	0.676630966	0.329134956	0.481581928	0.263820172	0.646426699	0.404166945	0.413222681	0.410742778	0.310640331	0.4897773
GSM	0.28	0.471	0.525	0.4928	0.503382	0.42464	0.1	0.672	0.38	0.5345	0.3	0.2	0.3	0.6	0.312	0.54	0.2	0.62615	0.37	0.397	0.423	0.270	0.464

56565590_treat	1990163	159257	471076	23119	755	732	98977094	410481	2682232	45123	25764685	41601982	37332422	74809997	807629	1173318	40487833	4251	58207	783233	629526	934992	218572
GSM5656594_treat	0.566506875	0.644453375	0.593769389	0.601554331	0.520899826	0.454700472	0.316538628	0.735517135	0.656576296	0.598800189	0.643546778	0.347210199	0.482687677	0.698919656	0.362130982	0.58722387	0.412638402	0.659601701	0.522970352	0.477750697	0.47398604	0.326034684	0.609658441
GSM5656597_treat	0.075827645	0.289717044	0.54283507	0.527630761	0.465516146	0.493113215	0.046408615	0.587109666	0.298414224	0.502259509	0.29633393	0.191237645	0.227448641	0.771904064	0.305149219	0.446078577	0.287792352	0.633431159	0.293085412	0.391445734	0.412753019	0.277821287	0.259862713
GSM5656599_treat	0.422948985	0.668695539	0.585531109	0.554500952	0.463496525	0.44674332	0.279938797	0.732036692	0.577081315	0.589859073	0.548435448	0.325321497	0.36667261	0.704059023	0.33963704	0.559287515	0.389238307	0.665210692	0.511148571	0.451239407	0.423138403	0.356080243	0.541118857
GSM5656609_t	0.618516659	0.660988762	0.653904449	0.696745173	0.534778839	0.463740911	0.31342	0.748731877	0.653429283	0.673188049	0.80007	0.42162	0.57764	0.78153	0.400776788	0.644263776	0.54260	0.727167203	0.553915128	0.510436285	0.544212565	0.365236503	0.588101938

reat							0101				4757	0241	9189	0821			9182						
GSM5656614_treat	0.252889973	0.402102143	0.520617117	0.537414909	0.468753548	0.44757336	0.134046204	0.651527609	0.35263705	0.545122567	0.340505547	0.224463818	0.287438337	0.73809928	0.297190509	0.503816123	0.273584494	0.629562921	0.345431064	0.418323297	0.414345858	0.271193898	0.366780787
GSM5656620_treat	0.335637489	0.592057307	0.54639296	0.597694144	0.513375804	0.477263522	0.197497054	0.705094375	0.394884099	0.567680759	0.525772502	0.325831682	0.388385992	0.710439042	0.325923954	0.574661235	0.39164019	0.636657063	0.429678211	0.402834009	0.444512012	0.363741156	0.542254598
GSM5656622_treat	0.330135657	0.480335336	0.629762408	0.661493382	0.501618595	0.494059825	0.244360816	0.708754277	0.434084007	0.608357043	0.655052417	0.380343045	0.529164363	0.769848928	0.35672475	0.55600034	0.545930102	0.655477014	0.511954948	0.471376449	0.496089163	0.373733509	0.515556463
GSM5656623_treat	0.332676949	0.554355887	0.55797607	0.60346524	0.527038669	0.448011189	0.31757434	0.696198093	0.503293468	0.610178501	0.650733675	0.358935812	0.5542027	0.728626583	0.372239483	0.583645775	0.540137627	0.697446965	0.485667374	0.470718434	0.474585556	0.316205529	0.520320236

GSM5656629_treat	0.647853424	0.778260957	0.744360759	0.642607498	0.555120185	0.453746318	0.426141758	0.742852769	0.719009923	0.691670163	0.842208503	0.490770688	0.564987398	0.7469697	0.493151334	0.644404441	0.330835171	0.757181049	0.658451782	0.544668234	0.615959075	0.371864751	0.652014352
GSM5656634_treat	0.262048466	0.289714696	0.423839056	0.516664927	0.472131941	0.531239592	0	0.619655249	0.320116525	0.540964892	0.317436041	0.228777293	0.219507741	0.71194051	0.252589532	0.491588518	0.197263676	0.6448163	0.324246063	0.339936443	0.358974434	0.347284213	0.372495607
GSM5656643_treat	0.249672556	0.384786851	0.451797629	0.508587135	0.469063765	0.479370104	0.117022155	0.68102159	0.318696865	0.525133136	0.283055734	0.228572929	0.300536093	0.717084809	0.302154915	0.545802818	0.207208553	0.629729431	0.302786406	0.358095128	0.413453262	0.259703911	0.452720508
GSM5656649_treat	0.217307448	0.465924147	0.496369819	0.55579511	0.484636068	0.455243709	0.187198077	0.669357936	0.324824507	0.55948615	0.403811833	0.256389009	0.302005209	0.711939436	0.310587951	0.52012968	0.241416108	0.641507668	0.396660206	0.405287057	0.423175996	0.279760429	0.473285785
GSM5656	0.312228	0.4766828	0.6271520	0.5465472	0.502971267	0.508067616	0.171	0.7241253	0.366948	0.564607404	0.489	0.304	0.366	0.720	0.3470864	0.593593	0.322	0.642065811	0.421493	0.4246413	0.4347746	0.3383501	0.4881908

652_treat	82	87	24				780522	79	116		312071	108499	653217	513702	77	266	462954		883	7	84	46	07
GSM5656656_treat	0.2550845594	0.464593422	0.573735912	0.602796316	0.476894378	0.468581636	0.164088222	0.678221761	0.337573045	0.554721576	0.45096888	0.260034921	0.37449157	0.757746893	0.33530595	0.575691261	0.29516345	0.67684342	0.377889026	0.437636271	0.455194864	0.290722399	0.453167242
