# Supplementary material for: Prognostic and therapeutic implication of m6A methylation in Crohn disease
Source: Medicine (Baltimore). 2022 Dec 23;101(51):e32399. doi: 10.1097/MD.0000000000032399 (PMC9794314; doi:10.1097/MD.0000000000032399)
Supplement: Supplementary file 7 [file medi-101-e32399-s007.pdf]

Supplemental Table 7. The expression of intersection genes

ID	XPNPEP2	REEP6	ACE	SLC13A2
GSM5656171_treat	9.732475	9.118614	9.138196	8.362452
GSM5656174_treat	11.406923	10.946884	11.519007	10.631469
GSM5656175_treat	5.163738	4.837144	7.030282	3.566394
GSM5656177_treat	10.093601	8.704666	9.977483	8.399943
GSM5656179_treat	8.532462	7.89087	8.843356	7.368328
GSM5656183_treat	7.07144	5.684777	5.795069	5.828093
GSM5656184_treat	9.628785	9.596681	10.086795	7.506515
GSM5656186_treat	10.636552	10.013397	10.681403	8.892645
GSM5656187_treat	10.152344	8.600057	9.151717	8.581335
GSM5656192_treat	8.488399	9.676213	9.381184	9.061832
GSM5656193_treat	6.611702	6.475026	6.167809	3.732575
GSM5656195_treat	11.305403	11.028736	11.684091	10.256516
GSM5656199_treat	11.156359	10.53124	10.870559	9.863202
GSM5656200_treat	11.845877	11.634585	11.681149	10.701946
GSM5656203_treat	10.267845	9.837184	9.758314	8.818733
GSM5656207_treat	12.015797	10.965403	11.472	9.907109
GSM5656209_treat	9.949023	8.009574	9.896186	8.343403
GSM5656212_treat	10.895779	9.975144	10.628779	8.768255
GSM5656214_treat	11.628654	10.667162	11.300917	8.768255
GSM5656217_treat	8.368091	7.568672	8.813815	7.396797
GSM5656220_treat	6.766978	5.333718	5.536297	3.510334
GSM5656223_treat	11.464173	10.628779	11.145921	9.514963
GSM5656226_treat	11.583872	10.41077	11.363323	8.396253
GSM5656228_treat	9.180371	8.03651	9.360649	8.472443
GSM5656231_treat	6.198551	5.781888	6.383862	3.512183
GSM5656235_treat	10.725343	10.363404	10.887934	9.729139
GSM5656237_treat	10.769532	9.255287	9.666323	8.220976
GSM5656240_treat	10.413962	9.374582	10.120841	10.363404
GSM5656243_treat	11.583872	10.460447	11.456579	9.322929
GSM5656246_treat	9.447386	7.792719	8.378855	8.137131
GSM5656249_treat	11.192781	10.42937	11.170523	10.018234
GSM5656254_treat	9.734423	10.09696	10.128593	8.514109
GSM5656255_treat	5.154635	4.684489	5.484946	3.49244
GSM5656259_treat	9.409038	8.835229	9.676213	7.542824
GSM5656262_treat	9.379525	8.787864	8.895873	8.826709
GSM5656264_treat	9.818802	9.1677	9.989744	10.155025
GSM5656265_treat	9.145446	7.53481	8.196332	8.160136
GSM5656267_treat	10.3306	9.478077	10.373807	8.229074
GSM5656273_treat	5.573637	5.339167	5.095833	4.4874
GSM5656274_treat	6.504364	6.949304	7.158625	6.472923
GSM5656277_treat	9.674973	9.640898	9.82621	7.657133

GSM5656280_treat	8.722087	8.298777	9.47414	7.706497
GSM5656282_treat	11.583872	10.466146	10.815088	10.432617
GSM5656285_treat	10.772393	10.182985	10.742322	10.016611
GSM5656291_treat	9.896928	9.077733	9.758982	9.404582
GSM5656294_treat	7.679003	7.151059	8.275534	7.057645
GSM5656297_treat	9.295817	8.612643	8.974017	8.99975
GSM5656303_treat	11.812578	10.818144	11.10663	10.649872
GSM5656305_treat	11.537615	10.593831	11.453983	10.288314
GSM5656309_treat	10.316816	8.774148	9.65153	8.124137
GSM5656311_treat	11.86634	10.782909	11.258674	9.462992
GSM5656316_treat	6.935634	6.151073	6.271058	4.999719
GSM5656318_treat	7.420233	7.531132	7.305708	6.982409
GSM5656321_treat	9.553106	9.27095	9.366601	8.575009
GSM5656323_treat	6.622685	5.85882	5.974603	3.746581
GSM5656325_treat	10.135633	9.368731	10.393582	8.520614
GSM5656327_treat	5.895562	5.042006	5.833598	3.513567
GSM5656329_treat	4.995608	5.580188	6.173588	5.82843
GSM5656334_treat	6.353851	6.035739	6.440955	3.552717
GSM5656335_treat	6.96625	6.415874	7.278185	4.899031
GSM5656338_treat	10.293282	9.407888	9.243114	8.092072
GSM5656342_treat	6.054939	6.465204	6.390561	4.531528
GSM5656346_treat	7.539459	7.198726	7.215507	5.611011
GSM5656348_treat	10.610045	10.398901	10.497408	9.723376
GSM5656351_treat	10.899049	9.954353	10.105538	9.867489
GSM5656353_treat	10.623688	9.480813	10.88956	7.881039
GSM5656355_treat	6.679282	5.9771	6.726835	5.090361
GSM5656359_treat	5.295897	5.740758	5.934253	3.549461
GSM5656361_treat	7.222725	6.608173	7.381743	6.183471
GSM5656365_treat	7.437648	7.092902	8.192163	5.969622
GSM5656366_treat	6.072359	5.108513	6.520236	3.58539
GSM5656368_treat	6.412301	8.660466	7.098372	5.865814
GSM5656372_treat	5.421793	5.007881	5.712618	5.114541
GSM5656374_treat	10.029454	9.184912	9.603287	8.145165
GSM5656375_treat	4.743107	4.89372	4.996614	3.739522
GSM5656380_treat	6.0317	6.00119	6.739117	4.355402
GSM5656381_treat	8.552858	8.224537	8.876042	6.96873
GSM5656384_treat	10.639254	9.609296	10.622507	8.188619
GSM5656387_treat	12.453098	11.419087	12.1497	9.346636
GSM5656388_treat	10.582815	9.986651	10.996804	9.442893
GSM5656390_treat	10.88956	9.079586	9.743044	8.647766
GSM5656392_treat	5.528794	6.514587	6.752787	3.619499
GSM5656393_treat	4.2757	4.43444	5.255473	3.822416
GSM5656395_treat	7.061781	6.721807	7.839385	7.066994

GSM5656399_treat	5.294901	6.455872	6.325207	3.613082
GSM5656400_treat	9.044571	8.653732	9.059475	8.815453
GSM5656403_treat	9.509258	8.860061	9.114445	8.625242
GSM5656406_treat	5.737625	5.480575	6.317311	3.61131
GSM5656407_treat	8.451533	7.756015	8.732645	6.898929
GSM5656409_treat	6.699047	6.381689	5.881554	3.989575
GSM5656412_treat	4.51054	5.639591	6.543097	3.553191
GSM5656416_treat	11.274075	10.973868	10.975562	9.854597
GSM5656418_treat	9.083327	8.868785	8.732239	8.516398
GSM5656421_treat	5.355078	7.473964	5.791959	3.384223
GSM5656424_treat	10.950159	10.29429	10.188646	7.798432
GSM5656426_treat	7.283382	6.814877	7.751489	5.64674
GSM5656429_treat	10.697668	10.017404	10.100337	9.000637
GSM5656431_treat	10.483518	10.731097	10.036841	9.291725
GSM5656432_treat	10.201615	9.251134	10.140032	8.265156
GSM5656434_treat	8.787864	7.547612	8.333843	6.981333
GSM5656435_treat	11.581209	10.775367	11.170523	8.466333
GSM5656439_treat	9.366069	8.996914	9.472509	7.369696
GSM5656443_treat	4.995608	4.943225	5.881929	3.425443
GSM5656444_treat	4.642717	5.079455	5.816208	4.539095
GSM5656447_treat	11.224107	10.138298	10.462712	9.457375
GSM5656451_treat	5.126216	6.158017	6.176546	3.931972
GSM5656452_treat	9.99129	9.422628	9.425318	7.897044
GSM5656454_treat	7.848806	8.021564	7.845773	6.271445
GSM5656458_treat	11.600467	11.120224	10.984277	10.948524
GSM5656460_treat	10.741035	10.251868	10.590226	8.239305
GSM5656461_treat	10.097812	9.069301	9.482499	9.183439
GSM5656464_treat	11.162334	10.169417	9.586495	8.679128
GSM5656465_treat	10.436913	8.660872	9.611092	9.422034
GSM5656467_treat	6.813147	6.53778	6.246904	3.57334
GSM5656469_treat	10.875109	10.71995	11.456579	8.162534
GSM5656473_treat	7.367644	7.619152	6.660996	6.087432
GSM5656478_treat	10.23593	9.350381	9.839239	8.560932
GSM5656481_treat	7.324986	8.301612	7.646616	7.914394
GSM5656483_treat	10.460447	9.549593	8.943253	8.157968
GSM5656486_treat	9.58475	8.750502	9.385044	8.098695
GSM5656487_treat	11.835381	11.019607	10.034289	8.401764
GSM5656490_treat	5.555568	7.335442	6.609919	5.9613
GSM5656493_treat	9.053926	9.177919	10.19236	9.222561
GSM5656495_treat	9.702748	8.993345	9.379001	9.133391
GSM5656498_treat	8.325402	7.970764	7.097686	6.244787
GSM5656501_treat	6.699761	8.025391	6.500466	6.335477
GSM5656504_treat	6.303074	7.083998	6.198165	6.107601

GSM5656505_treat	8.004709	6.49143	7.086325	3.83581
GSM5656509_treat	9.301395	9.309531	9.051123	8.082977
GSM5656511_treat	9.65153	8.48084	8.960109	7.942084
GSM5656513_treat	10.714212	9.318263	10.076231	8.002346
GSM5656516_treat	7.807247	8.255494	6.524498	7.100389
GSM5656517_treat	10.861124	10.403258	9.761696	9.114904
GSM5656520_treat	11.909348	9.447947	10.769532	9.514393
GSM5656521_treat	7.910665	6.922225	7.905566	5.629648
GSM5656522_treat	4.51969	4.356184	5.682711	3.531615
GSM5656524_treat	10.750771	9.115836	10.229252	9.5196
GSM5656525_treat	4.995608	7.420971	7.828981	5.94427
GSM5656527_treat	8.84845	8.92378	8.055763	8.464844
GSM5656528_treat	9.668841	9.413329	9.483642	6.696952
GSM5656531_treat	6.813489	7.841407	8.549311	6.921164
GSM5656533_treat	5.927817	7.036821	7.343295	5.612384
GSM5656535_treat	10.741035	10.205212	10.689705	9.26034
GSM5656536_treat	10.143688	9.244141	10.681403	8.091402
GSM5656541_treat	11.959821	11.174511	11.556403	9.887445
GSM5656543_treat	4.997617	7.16037	5.658758	5.073256
GSM5656545_treat	7.5167	7.191193	5.760971	7.118467
GSM5656549_treat	4.606163	5.01707	5.447557	3.514529
GSM5656551_treat	5.247384	6.030623	6.298978	3.351113
GSM5656554_treat	7.729915	8.927689	9.316308	8.825885
GSM5656556_treat	7.423259	7.552903	6.737704	6.75138
GSM5656559_treat	5.698528	6.89784	7.145933	4.499289
GSM5656563_treat	11.485292	10.336598	11.835381	10.194161
GSM5656565_treat	10.558328	10.169417	10.83775	8.917699
GSM5656567_treat	6.594223	6.86412	7.090811	5.137399
GSM5656569_treat	8.46374	7.440738	8.930781	8.712027
GSM5656571_treat	9.933207	9.560692	10.069617	7.918129
GSM5656573_treat	4.492012	4.907896	6.209552	3.457453
GSM5656576_treat	6.752084	7.068682	7.129158	5.845889
GSM5656578_treat	10.750771	10.168531	10.90901	9.924325
GSM5656581_treat	6.99701	7.113027	6.421309	5.051318
GSM5656583_treat	6.267058	7.090811	7.082659	5.693403
GSM5656585_treat	6.698339	7.177908	6.897497	6.471547
GSM5656588_treat	10.943525	9.178869	11.224107	8.327258
GSM5656589_treat	6.985647	7.599738	7.034081	5.074244
GSM5656591_treat	4.728838	5.598371	6.403425	3.317147
GSM5656595_treat	7.146605	6.55732	6.841077	4.715177
GSM5656596_treat	10.12598	9.627036	10.491484	7.584354
GSM5656600_treat	4.691099	4.828544	5.314059	3.267058
GSM5656601_treat	10.38832	10.187719	10.24267	8.91905

GSM5656602_treat	9.799495	8.587903	8.683553	6.386369
GSM5656603_treat	9.833623	9.99693	10.098665	10.112282
GSM5656604_treat	8.757975	9.125735	9.465272	7.849805
GSM5656606_treat	10.5053	10.250942	10.652613	10.610045
GSM5656608_treat	5.040584	6.556626	6.689548	6.208801
GSM5656611_treat	10.208923	9.54485	10.450234	9.423141
GSM5656612_treat	10.490337	10.226464	10.440291	9.571378
GSM5656615_treat	10.9302	9.666323	10.728106	10.475426
GSM5656617_treat	10.352968	9.184403	10.731097	7.735353
GSM5656619_treat	10.883143	10.44131	11.09121	9.722006
GSM5656621_treat	7.563016	7.730262	6.239316	5.183014
GSM5656624_treat	10.332578	9.485311	9.643991	9.551357
GSM5656625_treat	9.812546	9.168725	9.342372	8.218458
GSM5656627_treat	10.895779	10.304971	10.622507	10.581593
GSM5656631_treat	11.065022	10.769532	10.71995	9.41827
GSM5656632_treat	12.317272	11.278483	12.070021	10.87374
GSM5656635_treat	12.290403	10.597401	11.503406	9.961319
GSM5656637_treat	10.464988	10.181093	10.381147	10.092791
GSM5656639_treat	10.879915	9.80436	10.130245	8.598492
GSM5656641_treat	8.263393	8.010593	7.906253	6.855654
GSM5656645_treat	7.880703	8.507942	8.197437	7.420971
GSM5656647_treat	9.99129	10.071207	9.81328	9.99447
GSM5656648_treat	9.029407	8.925138	9.011039	8.077404
GSM5656653_treat	11.230394	10.725343	10.752297	11.094968
GSM5656655_treat	5.356124	5.16816	5.832253	3.538476
GSM5656657_treat	9.534427	9.313736	8.935214	8.553999
GSM5656173_treat	11.267405	11.012483	11.065022	10.426011
GSM5656182_treat	11.367956	11.024902	11.10837	10.265047
GSM5656191_treat	7.909621	7.873999	7.628269	8.950345
GSM5656194_treat	10.994908	10.485815	10.484676	10.680168
GSM5656196_treat	11.170523	10.900752	11.551018	10.153267
GSM5656198_treat	9.942917	9.437447	10.027096	9.358485
GSM5656205_treat	6.033867	6.054939	6.374802	3.593176
GSM5656210_treat	9.937673	8.513341	9.177919	8.15047
GSM5656211_treat	11.12611	10.114003	10.046598	9.389877
GSM5656215_treat	12.118363	11.030451	11.521682	9.500981
GSM5656218_treat	10.333636	9.737195	10.250033	9.097321
GSM5656219_treat	11.404375	10.369592	11.426348	9.404582
GSM5656222_treat	12.140738	11.204936	11.077944	10.176535
GSM5656227_treat	7.886452	6.465895	6.57142	5.262366
GSM5656234_treat	11.608908	10.490337	11.411895	10.143688
GSM5656236_treat	10.549181	8.414715	8.89893	8.321718
GSM5656239_treat	7.977975	7.385432	8.045567	8.031355

GSM5656241_treat	8.649818	8.19427	8.360274	9.214555
GSM5656244_treat	12.434543	11.433736	11.779333	10.605018
GSM5656247_treat	7.299944	6.410145	6.659557	7.403313
GSM5656251_treat	11.052467	10.56648	11.581209	9.733115
GSM5656253_treat	10.103012	10.736718	11.170523	8.553605
GSM5656256_treat	8.736003	7.910665	7.551898	8.468252
GSM5656263_treat	9.507525	8.543633	8.527897	9.133391
GSM5656268_treat	11.433736	10.697668	11.077944	9.645274
GSM5656272_treat	10.918644	9.972136	10.773901	8.867066
GSM5656275_treat	10.070394	10.529922	10.612491	10.339566
GSM5656278_treat	10.526488	10.059625	10.948524	8.481644
GSM5656281_treat	11.25216	10.686926	10.98602	8.941024
GSM5656286_treat	11.54031	11.180437	11.112311	10.639254
GSM5656289_treat	11.118185	10.257504	11.196777	10.30402
GSM5656293_treat	11.116235	10.41077	11.172537	9.636104
GSM5656296_treat	9.034041	7.783222	7.925955	7.687299
GSM5656301_treat	11.967853	11.085388	11.296722	11.15441
GSM5656307_treat	10.870559	11.01075	10.972139	10.921842
GSM5656308_treat	9.569541	8.932554	8.944609	8.61782
GSM5656320_treat	11.005453	10.714212	10.693808	10.040883
GSM5656324_treat	11.186651	10.598686	9.867489	8.955652
GSM5656326_treat	11.54836	10.810593	11.339906	10.411836
GSM5656328_treat	11.102815	10.644536	10.76101	9.953541
GSM5656331_treat	5.857088	5.352364	6.409419	3.927646
GSM5656332_treat	11.03962	10.703312	10.343656	8.404386
GSM5656336_treat	11.606199	10.632752	11.375262	9.739771
GSM5656340_treat	9.452901	9.749112	7.889862	7.801147
GSM5656343_treat	11.323554	10.454725	10.963783	8.922503
GSM5656345_treat	11.093054	10.298254	10.791743	8.897605
GSM5656349_treat	12.048931	11.300917	11.723224	10.93873
GSM5656350_treat	11.543137	10.57785	11.254459	10.479911
GSM5656354_treat	11.948938	11.0686	11.757489	9.273463
GSM5656357_treat	10.665822	10.29429	9.844753	9.336521
GSM5656362_treat	11.28973	10.533659	10.66962	9.923578
GSM5656363_treat	11.815803	10.257504	11.451588	9.254252
GSM5656367_treat	10.791743	10.380122	10.84394	9.368196
GSM5656370_treat	11.416737	10.396812	10.980706	9.288203
GSM5656371_treat	6.240438	5.941108	5.821119	5.43845
GSM5656373_treat	11.54031	10.839361	11.0686	10.007753
GSM5656377_treat	10.667162	9.92577	9.426917	7.240465
GSM5656378_treat	8.66538	8.010593	8.664573	6.701493
GSM5656379_treat	10.688334	9.460737	10.038451	10.277444
GSM5656382_treat	11.553652	10.384159	10.957049	8.909809

GSM5656386_treat	12.611309	11.464173	11.806103	9.44009
GSM5656391_treat	11.089285	10.337597	10.163066	8.339344
GSM5656397_treat	8.646597	8.602404	8.641431	8.485445
GSM5656401_treat	11.672291	10.717023	11.059527	9.05578
GSM5656402_treat	11.307668	10.883143	11.160365	10.693808
GSM5656404_treat	9.95122	8.977612	9.555423	9.008774
GSM5656408_treat	11.28973	10.20068	11.18473	9.294832
GSM5656411_treat	11.472	10.686926	11.219664	9.071094
GSM5656413_treat	10.504199	9.190888	10.007753	8.329111
GSM5656415_treat	11.059527	10.511685	10.373807	9.582858
GSM5656419_treat	10.729591	10.828595	11.112311	9.930209
GSM5656422_treat	11.174511	10.701946	10.602545	8.922063
GSM5656427_treat	10.249009	9.406772	9.832234	8.715669
GSM5656433_treat	11.269591	9.749112	10.419495	9.857538
GSM5656436_treat	10.046598	9.266816	9.941381	7.606076
GSM5656438_treat	8.921641	8.285296	8.714008	7.772541
GSM5656440_treat	10.717023	10.389393	10.682848	8.321019
GSM5656442_treat	11.18473	9.952044	10.513459	8.904126
GSM5656446_treat	9.740453	8.549311	9.334454	9.090746
GSM5656449_treat	10.804868	11.495881	10.80057	10.0449
GSM5656453_treat	11.28973	10.610045	10.468484	9.97439
GSM5656456_treat	9.665084	9.783371	9.608104	7.516363
GSM5656457_treat	11.570059	11.448901	10.816578	10.953528
GSM5656459_treat	10.613719	10.321746	10.479911	8.859643
GSM5656462_treat	11.416737	10.471975	10.524147	10.032707
GSM5656466_treat	10.281357	9.245113	9.792715	10.100337
GSM5656468_treat	11.729238	11.065022	11.862939	8.98389
GSM5656472_treat	6.922225	7.046946	6.200386	4.024904
GSM5656475_treat	9.246579	9.372999	9.348837	6.865544
GSM5656476_treat	10.363404	10.10461	9.705962	8.830565
GSM5656479_treat	9.615352	10.175685	9.510982	9.543106
GSM5656484_treat	11.729238	10.80762	11.56738	10.715575
GSM5656491_treat	7.375043	8.291146	8.043525	6.295365
GSM5656494_treat	10.89433	10.502984	10.878301	10.563991
GSM5656497_treat	8.580112	8.324285	7.676262	8.21733
GSM5656499_treat	8.649394	8.835628	8.007886	7.582653
GSM5656502_treat	10.005376	9.095475	9.370309	8.459192
GSM5656507_treat	9.138677	8.727406	8.677534	8.317323
GSM5656510_treat	8.680359	8.248997	8.038294	8.11334
GSM5656512_treat	11.394381	10.262308	10.994908	8.575009
GSM5656515_treat	7.961147	8.380728	7.373662	7.39924
GSM5656519_treat	11.014236	10.250942	9.48702	9.065061
GSM5656523_treat	9.95049	9.848956	9.790003	9.958993

GSM5656526_treat	10.301157	11.034182	11.479787	9.29123
GSM5656529_treat	8.366964	8.514494	8.328352	7.621165
GSM5656538_treat	11.059527	10.387269	11.222008	9.519029
GSM5656540_treat	11.920039	11.070533	11.433736	10.035106
GSM5656544_treat	10.617627	10.11485	9.855284	9.520762
GSM5656547_treat	9.731782	9.657132	10.738281	9.779919
GSM5656550_treat	10.306926	9.929449	10.265975	9.267291
GSM5656552_treat	10.212558	9.246579	9.99129	8.080561
GSM5656553_treat	9.53729	10.255529	10.390464	10.040883
GSM5656557_treat	11.493281	10.787302	10.779096	9.775096
GSM5656560_treat	9.094124	8.520217	8.985265	7.636157
GSM5656561_treat	11.95624	11.234632	11.551018	10.376933
GSM5656566_treat	10.745146	9.830941	10.595059	8.422251
GSM5656568_treat	11.063258	10.383151	11.224107	10.333636
GSM5656572_treat	7.662813	6.641502	8.132673	5.985731
GSM5656574_treat	11.292	10.424855	10.439195	9.886731
GSM5656577_treat	5.999733	5.376452	4.992532	3.737729
GSM5656580_treat	9.988147	9.265283	9.127678	8.546266
GSM5656582_treat	9.690752	9.264784	9.374582	8.283887
GSM5656586_treat	10.875109	10.103868	10.507543	8.190057
GSM5656587_treat	11.532271	10.500728	11.030451	9.146879
GSM5656592_treat	10.311929	9.435195	10.05068	8.465956
GSM5656593_treat	10.842356	9.517291	10.007753	8.45384
GSM5656598_treat	9.178394	9.138196	8.867476	8.185439
GSM5656605_treat	11.78927	10.88956	11.394381	10.062115
GSM5656607_treat	10.931869	10.641762	11.124017	10.00229
GSM5656610_treat	11.654894	11.479787	11.702381	11.198815
GSM5656613_treat	11.344293	10.88956	10.831637	11.076071
GSM5656616_treat	11.131891	10.509957	10.876742	10.722655
GSM5656618_treat	11.766845	10.840867	11.479787	10.26884
GSM5656626_treat	11.466689	10.881487	11.269591	10.326612
GSM5656628_treat	11.283089	10.519434	10.521822	10.752297
GSM5656630_treat	11.234632	10.217084	10.276544	9.904954
GSM5656633_treat	11.382451	11.493281	11.776455	11.300917
GSM5656636_treat	11.796023	11.342089	11.256633	10.500728
GSM5656638_treat	10.769532	10.30402	10.689705	10.481092
GSM5656640_treat	12.46607	11.702381	11.411895	10.554119
GSM5656642_treat	10.87374	10.582815	10.40746	9.369794
GSM5656644_treat	10.207073	10.84394	10.515845	10.027096
GSM5656646_treat	11.174511	10.633964	10.86269	10.439195
GSM5656650_treat	10.086795	9.634273	9.43076	9.398089
GSM5656651_treat	11.192781	10.920239	10.775367	10.917014
GSM5656654_treat	11.431102	10.346728	10.991338	10.678902

GSM5656658_treat	11.856051	10.776825	11.114146	11.18473
GSM5656170_treat	11.594975	10.706064	10.484676	9.205127
GSM5656172_treat	8.510257	8.780805	9.14495	8.37771
GSM5656176_treat	10.220816	9.893129	10.496184	8.282745
GSM5656178_treat	11.292	10.781282	11.059527	9.228044
GSM5656181_treat	10.98602	9.756972	10.41077	8.588663
GSM5656188_treat	9.818802	9.117653	9.119576	8.301999
GSM5656197_treat	9.92207	9.217115	9.300892	8.547041
GSM5656201_treat	11.714282	11.096846	10.747926	10.099493
GSM5656204_treat	10.743827	9.86391	9.908706	8.230823
GSM5656206_treat	10.887934	10.320706	10.57785	9.377809
GSM5656213_treat	11.24303	10.747926	10.393582	9.430203
GSM5656216_treat	10.401068	9.354189	9.47139	8.118913
GSM5656221_treat	10.076231	8.193587	9.539572	8.027559
GSM5656224_treat	10.368511	10.065446	9.504171	8.294425
GSM5656225_treat	10.918644	10.19236	10.299209	8.796159
GSM5656229_treat	10.736718	9.919887	10.356154	9.314261
GSM5656233_treat	7.578503	7.445728	7.460637	6.789569
GSM5656242_treat	4.816013	5.658758	6.701139	6.024537
GSM5656248_treat	8.279466	6.909013	6.623059	4.750436
GSM5656250_treat	8.89893	8.922503	8.310306	8.668535
GSM5656252_treat	10.206165	9.137719	9.969007	8.508326
GSM5656257_treat	9.757626	9.95049	8.68276	8.404038
GSM5656260_treat	9.9497	9.230523	9.910936	8.14173
GSM5656261_treat	10.579097	9.185953	10.265047	7.756981
GSM5656266_treat	10.658019	9.705307	9.806386	8.731011
GSM5656269_treat	10.887934	10.479911	10.409681	9.115374
GSM5656276_treat	8.789846	9.195314	8.998814	8.809671
GSM5656284_treat	10.978983	10.633964	10.695061	9.794026
GSM5656290_treat	7.795388	7.546214	6.828252	7.099372
GSM5656295_treat	10.917014	10.572709	10.80762	9.846078
GSM5656298_treat	9.012848	8.927689	8.41808	7.938663
GSM5656302_treat	10.87374	9.557131	9.749744	8.467453
GSM5656306_treat	9.531014	10.017404	9.346636	9.214004
GSM5656312_treat	6.20993	5.717099	5.652256	4.728099
GSM5656315_treat	10.984277	9.971426	10.224611	8.574248
GSM5656317_treat	10.060506	9.094124	8.904126	8.475581
GSM5656319_treat	10.606236	10.18201	10.655429	9.44121
GSM5656322_treat	9.873927	9.187923	8.583271	7.489631
GSM5656330_treat	10.742322	10.146223	10.53484	9.32755
GSM5656333_treat	10.612491	10.314723	9.670613	8.477486
GSM5656337_treat	8.945038	8.952063	8.15437	7.798776
GSM5656339_treat	9.761004	9.99129	9.339174	8.331624

GSM5656341_treat	11.256633	10.579097	10.991338	9.958234
GSM5656344_treat	11.083521	9.9228	10.321746	9.222038
GSM5656347_treat	10.009333	9.643991	10.152344	8.455025
GSM5656352_treat	11.399395	10.569012	10.910447	9.753012
GSM5656356_treat	11.057792	10.128593	9.841957	9.325477
GSM5656358_treat	11.495881	10.940317	10.968712	9.215039
GSM5656360_treat	11.01075	10.098665	10.369592	8.157244
GSM5656364_treat	11.280832	9.542485	10.554119	8.936109
GSM5656369_treat	10.301157	9.020687	8.997885	7.310932
GSM5656376_treat	10.083476	10.788787	10.561626	9.685116
GSM5656383_treat	11.037824	9.843386	10.555176	8.045926
GSM5656385_treat	10.236832	9.215597	10.108873	7.748075
GSM5656389_treat	11.245291	10.533659	10.20426	8.681195
GSM5656394_treat	10.501868	9.420998	9.278013	8.858391
GSM5656396_treat	8.709925	8.564841	8.719229	7.207036
GSM5656398_treat	10.962069	10.460447	9.284603	8.678722
GSM5656405_treat	10.968712	9.830941	10.865864	8.288935
GSM5656410_treat	9.456807	8.286754	8.984794	8.644246
GSM5656414_treat	9.270423	9.138196	9.360096	7.96523
GSM5656417_treat	11.145921	9.860362	10.599978	8.957449
GSM5656420_treat	10.825685	8.854027	10.217084	8.005043
GSM5656423_treat	8.786168	9.272959	8.95383	8.093493
GSM5656425_treat	10.65928	10.038451	10.994908	9.464683
GSM5656428_treat	11.178435	9.893906	10.213498	9.071094
GSM5656430_treat	11.625943	10.98602	11.32127	9.043199
GSM5656437_treat	10.333636	9.881728	9.969773	8.193915
GSM5656441_treat	10.23593	9.696456	10.348841	8.691706
GSM5656445_treat	10.073787	9.157941	9.680589	8.254085
GSM5656448_treat	10.731097	9.933976	10.11485	8.346355
GSM5656450_treat	10.491484	10.185811	10.000687	9.250085
GSM5656455_treat	10.290254	9.686403	9.238678	8.730614
GSM5656463_treat	10.689705	9.965824	9.385524	8.488854
GSM5656470_treat	10.198841	9.478077	9.719403	8.579695
GSM5656471_treat	10.491484	9.459639	9.227529	8.607933
GSM5656474_treat	10.741035	9.61232	9.956656	8.545887
GSM5656477_treat	10.161298	9.429634	9.505319	9.472509
GSM5656480_treat	9.241116	9.999222	9.896186	9.790003
GSM5656482_treat	11.42394	11.10837	10.875109	9.756972
GSM5656485_treat	10.955251	9.511608	9.937673	9.052553
GSM5656488_treat	4.54176	4.576193	5.755811	3.908626
GSM5656489_treat	10.057172	9.147393	9.511608	8.422606
GSM5656492_treat	10.672252	10.758133	10.3138	9.687038
GSM5656496_treat	10.406306	8.972696	9.673683	8.134703

GSM5656500_treat	10.324687	9.151211	9.112067	8.211664
GSM5656503_treat	7.785188	6.895398	6.20589	5.733462
GSM5656506_treat	10.091088	9.076326	9.633077	8.855329
GSM5656508_treat	11.556403	10.540817	10.668384	9.178869
GSM5656514_treat	10.474264	8.361362	9.54485	8.057845
GSM5656518_treat	10.607595	9.542485	9.498706	8.373591
GSM5656530_treat	11.098825	10.331599	10.558953	9.001976
GSM5656532_treat	6.714231	6.995601	7.380036	5.46312
GSM5656534_treat	10.56648	10.277444	10.341569	8.992026
GSM5656537_treat	10.745146	9.849629	10.318717	8.825455
GSM5656542_treat	10.203395	9.343982	10.376933	7.621165
GSM5656546_treat	10.946884	10.41282	10.127747	9.343443
GSM5656548_treat	11.202859	10.693808	11.431102	9.59731
GSM5656555_treat	10.017404	10.387269	10.847089	9.643383
GSM5656558_treat	5.55869	5.255473	5.277002	4.250257
GSM5656562_treat	10.735286	10.168531	10.318717	9.181363
GSM5656564_treat	11.583872	10.576499	10.881487	9.198238
GSM5656570_treat	10.782909	10.036841	10.3138	9.186456
GSM5656575_treat	10.458181	9.849629	10.3306	8.859236
GSM5656579_treat	9.536713	8.929447	8.395895	7.598771
GSM5656584_treat	9.899188	9.16676	8.676699	8.579695
GSM5656590_treat	9.73379	9.61723	9.741775	8.284219
GSM5656594_treat	8.774543	7.15968	8.651737	7.245971
GSM5656597_treat	12.21765	11.258674	11.469311	9.411246
GSM5656599_treat	7.53545	7.436297	7.871261	7.372692
GSM5656609_treat	9.410697	9.462434	9.555971	7.8863
GSM5656614_treat	11.335162	10.101176	11.148069	9.212002
GSM5656620_treat	9.700236	8.731011	9.481369	8.574248
GSM5656622_treat	10.439195	10.131143	10.389393	9.211505
GSM5656623_treat	10.458181	9.541913	9.191404	8.46522
GSM5656629_treat	4.146969	5.109183	6.232078	3.315652
GSM5656634_treat	7.526104	6.288109	6.726835	4.391659
GSM5656643_treat	10.747926	10.175685	10.722655	9.198731
GSM5656649_treat	10.133857	9.334961	8.592262	8.990224
GSM5656652_treat	10.525331	10.585254	9.484177	9.279588
GSM5656656_treat	11.681149	10.61497	10.840867	9.387151
