# Supplementary material for: Prognostic and therapeutic implication of m6A methylation in Crohn disease
Source: Medicine (Baltimore). 2022 Dec 23;101(51):e32399. doi: 10.1097/MD.0000000000032399 (PMC9794314; doi:10.1097/MD.0000000000032399)
Supplement: Supplementary file 8 [file medi-101-e32399-s008.pdf]

Supplemental Table 8. GO enrichment analysis

ONTOLOGY	ID	Description	GeneRatio	BgRatio	pvalue	p.adjust	qvalue	geneID	Count
BP	GO:0046942	carboxylic acid transport	2/4	273/18723	0.001246629	0.025867257	0.006188339	ACE/SLC13A2	2
BP	GO:0015849	organic acid transport	2/4	303/18723	0.001532918	0.025867257	0.006188339	ACE/SLC13A2	2
BP	GO:0015711	organic anion transport	2/4	354/18723	0.002085708	0.025867257	0.006188339	ACE/SLC13A2	2
BP	GO:0002002	regulation of angiotensin levels in blood	1/4	10/18723	0.00213487	0.025867257	0.006188339	ACE	1
BP	GO:0002003	angiotensin maturation	1/4	10/18723	0.00213487	0.025867257	0.006188339	ACE	1
BP	GO:0071838	cell proliferation in bone marrow	1/4	10/18723	0.00213487	0.025867257	0.006188339	ACE	1
BP	GO:2000169	regulation of peptidyl-cysteine S-nitrosylation	1/4	10/18723	0.00213487	0.025867257	0.006188339	ACE	1
BP	GO:0042447	hormone catabolic process	1/4	11/18723	0.002348168	0.025867257	0.006188339	ACE	1
BP	GO:1902033	regulation of hematopoietic stem cell proliferation	1/4	11/18723	0.002348168	0.025867257	0.006188339	ACE	1
BP	GO:0001991	regulation of systemic arterial blood pressure by circulatory renin-angiotensin	1/4	16/18723	0.00341415	0.025867257	0.006188339	ACE	1
BP	GO:0003084	positive regulation of systemic arterial blood pressure	1/4	16/18723	0.00341415	0.025867257	0.006188339	ACE	1
BP	GO:0017014	protein nitrosylation	1/4	16/18723	0.00341415	0.025867257	0.006188339	ACE	1
BP	GO:0018119	peptidyl-cysteine S-nitrosylation	1/4	16/18723	0.00341415	0.025867257	0.006188339	ACE	1
BP	GO:0016264	gap junction assembly	1/4	17/18723	0.003627243	0.025867257	0.006188339	ACE	1
BP	GO:0038083	peptidyl-tyrosine autophosphorylation	1/4	21/18723	0.004479276	0.025867257	0.006188339	ACE	1
BP	GO:0050908	detection of light stimulus involved in visual perception	1/4	21/18723	0.004479276	0.025867257	0.006188339	REEP6	1
BP	GO:0050962	detection of light stimulus involved in sensory perception	1/4	21/18723	0.004479276	0.025867257	0.006188339	REEP6	1
BP	GO:0003081	regulation of systemic arterial blood pressure by renin-angiotensin	1/4	23/18723	0.004905088	0.025867257	0.006188339	ACE	1
BP	GO:0016486	peptide hormone processing	1/4	24/18723	0.005117942	0.025867257	0.006188339	ACE	1

BP	GO:0140448	signaling receptor ligand precursor processing	1/4	25/18723	0.005330763	0.025867257	0.006188339	ACE	1
BP	GO:0071425	hematopoietic stem cell proliferation	1/4	26/18723	0.005543549	0.025867257	0.006188339	ACE	1
BP	GO:0031954	positive regulation of protein autophosphorylation	1/4	27/18723	0.005756301	0.025867257	0.006188339	ACE	1
BP	GO:0002474	antigen processing and presentation of peptide antigen via MHC class I	1/4	28/18723	0.005969019	0.025867257	0.006188339	ACE	1
BP	GO:0015740	C4-dicarboxylate transport	1/4	28/18723	0.005969019	0.025867257	0.006188339	SLC13A2	1
BP	GO:0060218	hematopoietic stem cell differentiation	1/4	30/18723	0.006394352	0.025867257	0.006188339	ACE	1
BP	GO:0043171	peptide catabolic process	1/4	31/18723	0.006606968	0.025867257	0.006188339	ACE	1
BP	GO:0050482	arachidonic acid secretion	1/4	31/18723	0.006606968	0.025867257	0.006188339	ACE	1
BP	GO:1903963	arachidonate transport	1/4	31/18723	0.006606968	0.025867257	0.006188339	ACE	1
BP	GO:1901889	negative regulation of cell junction assembly	1/4	32/18723	0.006819549	0.025867257	0.006188339	ACE	1
BP	GO:0002446	neutrophil mediated immunity	1/4	34/18723	0.00724461	0.02656357	0.006354921	ACE	1
BP	GO:0001990	regulation of systemic arterial blood pressure by hormone	1/4	37/18723	0.007881945	0.027968192	0.006690955	ACE	1
BP	GO:0045777	positive regulation of blood pressure	1/4	40/18723	0.008518973	0.029283969	0.007005734	ACE	1
BP	GO:0031952	regulation of protein autophosphorylation	1/4	43/18723	0.009155694	0.030186942	0.007221756	ACE	1
BP	GO:0032309	icosanoid secretion	1/4	45/18723	0.009580004	0.030186942	0.007221756	ACE	1
BP	GO:0001974	blood vessel remodeling	1/4	46/18723	0.009792109	0.030186942	0.007221756	ACE	1
BP	GO:0003044	regulation of systemic arterial blood pressure mediated by a chemical signal	1/4	47/18723	0.010004179	0.030186942	0.007221756	ACE	1
BP	GO:0018198	peptidyl-cysteine modification	1/4	49/18723	0.010428216	0.030186942	0.007221756	ACE	1
BP	GO:0072091	regulation of stem cell proliferation	1/4	49/18723	0.010428216	0.030186942	0.007221756	ACE	1
BP	GO:0009584	detection of visible	1/4	51/18723	0.010852118	0.030608538	0.007322617	REEP6	1

		light							
BP	GO:0071715	icosanoid transport	1/4	53/18723	0.011275883	0.031008679	0.007418344	ACE	1
BP	GO:0061098	positive regulation of protein tyrosine kinase activity	1/4	55/18723	0.011699513	0.031388936	0.007509315	ACE	1
BP	GO:0048002	antigen processing and presentation of peptide antigen	1/4	62/18723	0.013181143	0.034048419	0.008145555	ACE	1
BP	GO:0015909	long-chain fatty acid transport	1/4	65/18723	0.013815617	0.034048419	0.008145555	ACE	1
BP	GO:0019229	regulation of vasoconstriction	1/4	66/18723	0.01402704	0.034048419	0.008145555	ACE	1
BP	GO:0009583	detection of light stimulus	1/4	67/18723	0.01423843	0.034048419	0.008145555	REEP6	1
BP	GO:0050435	amyloid-beta metabolic process	1/4	67/18723	0.01423843	0.034048419	0.008145555	ACE	1
BP	GO:0072089	stem cell proliferation	1/4	73/18723	0.015506052	0.036290761	0.008682	ACE	1
BP	GO:0050886	endocrine process	1/4	83/18723	0.01761604	0.038182022	0.009134455	ACE	1
BP	GO:0006835	dicarboxylic acid transport	1/4	84/18723	0.017826852	0.038182022	0.009134455	SLC13A2	1
BP	GO:0042310	vasoconstriction	1/4	84/18723	0.017826852	0.038182022	0.009134455	ACE	1
BP	GO:0032092	positive regulation of protein binding	1/4	85/18723	0.01803763	0.038182022	0.009134455	ACE	1
BP	GO:0015908	fatty acid transport	1/4	86/18723	0.018248374	0.038182022	0.009134455	ACE	1
BP	GO:0007029	endoplasmic reticulum organization	1/4	87/18723	0.018459084	0.038182022	0.009134455	REEP6	1
BP	GO:0014910	regulation of smooth muscle cell migration	1/4	89/18723	0.018880403	0.038182022	0.009134455	ACE	1
BP	GO:0061097	regulation of protein tyrosine kinase activity	1/4	90/18723	0.019091011	0.038182022	0.009134455	ACE	1
BP	GO:0032091	negative regulation of protein binding	1/4	94/18723	0.019933106	0.039001299	0.009330454	ACE	1
BP	GO:0003073	regulation of systemic arterial blood pressure	1/4	96/18723	0.02035395	0.039001299	0.009330454	ACE	1
BP	GO:0014909	smooth muscle cell migration	1/4	97/18723	0.020564321	0.039001299	0.009330454	ACE	1
BP	GO:0002444	myeloid leukocyte mediated immunity	1/4	99/18723	0.020984962	0.039124505	0.00935993	ACE	1
BP	GO:0019882	antigen processing and presentation	1/4	106/18723	0.022456138	0.041169586	0.009849183	ACE	1
BP	GO:0014812	muscle cell migration	1/4	110/18723	0.023296065	0.041704126	0.009977064	ACE	1
BP	GO:0015718	monocarboxylic acid transport	1/4	111/18723	0.023505962	0.041704126	0.009977064	ACE	1

BP	GO:0002244	hematopoietic progenitor cell differentiation	1/4	114/18723	0.02413545	0.042141262	0.010081642	ACE	1
BP	GO:0009581	detection of external stimulus	1/4	135/18723	0.028533352	0.048522958	0.011608363	REEP6	1
BP	GO:0009582	detection of abiotic stimulus	1/4	138/18723	0.029160408	0.048522958	0.011608363	REEP6	1
BP	GO:0035296	regulation of tube diameter	1/4	141/18723	0.029787161	0.048522958	0.011608363	ACE	1
BP	GO:0097746	blood vessel diameter maintenance	1/4	141/18723	0.029787161	0.048522958	0.011608363	ACE	1
BP	GO:0035150	regulation of tube size	1/4	142/18723	0.029996011	0.048522958	0.011608363	ACE	1
BP	GO:0007043	cell-cell junction assembly	1/4	146/18723	0.030831074	0.049059376	0.011736693	ACE	1
BP	GO:1905039	carboxylic acid transmembrane transport	1/4	149/18723	0.031457017	0.049059376	0.011736693	SLC13A2	1
BP	GO:1903825	organic acid transmembrane transport	1/4	150/18723	0.031665597	0.049059376	0.011736693	SLC13A2	1
BP	GO:0051100	negative regulation of binding	1/4	162/18723	0.034165935	0.052197956	0.012487549	ACE	1
BP	GO:0051099	positive regulation of binding	1/4	173/18723	0.036453655	0.054805518	0.013111368	ACE	1
BP	GO:0048771	tissue remodeling	1/4	175/18723	0.036869167	0.054805518	0.013111368	ACE	1
BP	GO:0008217	regulation of blood pressure	1/4	186/18723	0.039152081	0.057423052	0.013737572	ACE	1
BP	GO:0050731	positive regulation of peptidyl-tyrosine phosphorylation	1/4	193/18723	0.04060273	0.058767109	0.014059117	ACE	1
BP	GO:0043393	regulation of protein binding	1/4	196/18723	0.041223934	0.058891334	0.014088836	ACE	1
BP	GO:0045216	cell-cell junction organization	1/4	200/18723	0.042051736	0.05930373	0.014187495	ACE	1
BP	GO:1901888	regulation of cell junction assembly	1/4	204/18723	0.042879002	0.059527097	0.014240932	ACE	1
BP	GO:0048863	stem cell differentiation	1/4	206/18723	0.043292434	0.059527097	0.014240932	ACE	1
BP	GO:0007601	visual perception	1/4	215/18723	0.045151221	0.060932682	0.014577197	REEP6	1
BP	GO:0042445	hormone metabolic process	1/4	218/18723	0.045770214	0.060932682	0.014577197	ACE	1
BP	GO:0050953	sensory perception of light stimulus	1/4	219/18723	0.045976478	0.060932682	0.014577197	REEP6	1
BP	GO:0016485	protein processing	1/4	225/18723	0.047213361	0.061632856	0.014744702	ACE	1

BP	GO:0046777	protein autophosphorylation	1/4	227/18723	0.047625388	0.061632856	0.014744702	ACE	1
CC	GO:0001917	photoreceptor inner segment	1/4	64/19550	0.013031463	0.055308729	0.016634204	REEP6	1
CC	GO:0030665	clathrin-coated vesicle membrane	1/4	116/19550	0.0235254	0.055308729	0.016634204	REEP6	1
CC	GO:0031225	anchored component of membrane	1/4	170/19550	0.034334147	0.055308729	0.016634204	XPNPEP2	1
CC	GO:0030662	coated vesicle membrane	1/4	181/19550	0.036524881	0.055308729	0.016634204	REEP6	1
CC	GO:0030136	clathrin-coated vesicle	1/4	196/19550	0.039506235	0.055308729	0.016634204	REEP6	1
MF	GO:0008235	metalloexopeptidase activity	2/4	72/18368	9.05E-05	0.002713649	0.00066651	XPNPEP2/ACE	2
MF	GO:0008238	exopeptidase activity	2/4	103/18368	0.000185481	0.002782212	0.00068335	XPNPEP2/ACE	2
MF	GO:0008237	metallopeptidase activity	2/4	190/18368	0.000629972	0.00629972	0.0015473	XPNPEP2/ACE	2
MF	GO:0070008	serine-type exopeptidase activity	1/4	10/18368	0.0021761	0.013050204	0.003205313	ACE	1
MF	GO:0031434	mitogen-activated protein kinase kinase binding	1/4	11/18368	0.002393515	0.013050204	0.003205313	ACE	1
MF	GO:0031404	chloride ion binding	1/4	13/18368	0.002828237	0.013050204	0.003205313	ACE	1
MF	GO:0016805	dipeptidase activity	1/4	15/18368	0.003262817	0.013050204	0.003205313	ACE	1
MF	GO:0015556	C4-dicarboxylate transmembrane transporter activity	1/4	16/18368	0.003480054	0.013050204	0.003205313	SLC13A2	1
MF	GO:0051019	mitogen-activated protein kinase binding	1/4	25/18368	0.005433589	0.01629411	0.004002062	ACE	1
MF	GO:0070006	metalloaminopeptidase activity	1/4	27/18368	0.005867317	0.01629411	0.004002062	XPNPEP2	1
MF	GO:0005310	dicarboxylic acid transmembrane transporter activity	1/4	30/18368	0.006517644	0.01629411	0.004002062	SLC13A2	1
MF	GO:0005343	organic acid:sodium symporter activity	1/4	30/18368	0.006517644	0.01629411	0.004002062	SLC13A2	1
MF	GO:0004177	aminopeptidase activity	1/4	40/18368	0.008683095	0.019997228	0.0049116	XPNPEP2	1
MF	GO:0004180	carboxypeptidase activity	1/4	43/18368	0.00933204	0.019997228	0.0049116	ACE	1
MF	GO:0015370	solute:sodium symporter activity	1/4	73/18368	0.015803976	0.031607952	0.007763357	SLC13A2	1
MF	GO:0015294	solute:cation symporter activity	1/4	104/18368	0.022458276	0.042109268	0.010342627	SLC13A2	1

MF	GO:0015293	symporter activity	1/4	144/18368	0.030994543	0.052862474	0.012983766	SLC13A2	1
MF	GO:0015081	sodium ion transmembrane transporter activity	1/4	152/18368	0.032695065	0.052862474	0.012983766	SLC13A2	1
MF	GO:0046943	carboxylic acid transmembrane transporter activity	1/4	163/18368	0.035029626	0.052862474	0.012983766	SLC13A2	1
MF	GO:0005342	organic acid transmembrane transporter activity	1/4	164/18368	0.03524165	0.052862474	0.012983766	SLC13A2	1
MF	GO:0008514	organic anion transmembrane transporter activity	1/4	185/18368	0.03968607	0.054517899	0.013390361	SLC13A2	1
MF	GO:0008236	serine-type peptidase activity	1/4	191/18368	0.04095308	0.054517899	0.013390361	ACE	1
MF	GO:0017171	serine hydrolase activity	1/4	195/18368	0.041797056	0.054517899	0.013390361	ACE	1
