# Supplementary material for: Prognostic and therapeutic implication of m6A methylation in Crohn disease
Source: Medicine (Baltimore). 2022 Dec 23;101(51):e32399. doi: 10.1097/MD.0000000000032399 (PMC9794314; doi:10.1097/MD.0000000000032399)
Supplement: Supplementary file 9 [file medi-101-e32399-s009.pdf]

Supplemental Table 9. KEGG enrichment analysis

ID	Description	GeneRatio	BgRatio	pvalue	p.adjust	qvalue	geneID	Count
hsa04614	Renin-angiotensin system	1/2	23/8112	0.005662921	0.035328724	0.00531259	ACE	1
hsa04924	Renin secretion	1/2	69/8112	0.016940523	0.035328724	0.00531259	ACE	1
hsa05410	Hypertrophic cardiomyopathy	1/2	90/8112	0.02206761	0.035328724	0.00531259	ACE	1
hsa05142	Chagas disease	1/2	102/8112	0.024991355	0.035328724	0.00531259	ACE	1
hsa04974	Protein digestion and absorption	1/2	103/8112	0.025234803	0.035328724	0.00531259	XPNPEP2	1
hsa05415	Diabetic cardiomyopathy	1/2	203/8112	0.049426084	0.056384699	0.008478902	ACE	1
