# Supplementary material for: Prognostic and therapeutic implication of m6A methylation in Crohn disease
Source: Medicine (Baltimore). 2022 Dec 23;101(51):e32399. doi: 10.1097/MD.0000000000032399 (PMC9794314; doi:10.1097/MD.0000000000032399)
Supplement: Supplementary file 10 [file medi-101-e32399-s010.pdf]

Supplemental Table 10. Sample classification based on differential expression of intersection genes

ID	geneCluster
GSM5656171_treat	A
GSM5656174_treat	B
GSM5656175_treat	C
GSM5656177_treat	A
GSM5656179_treat	A
GSM5656183_treat	C
GSM5656184_treat	A
GSM5656186_treat	B
GSM5656187_treat	A
GSM5656192_treat	A
GSM5656193_treat	C
GSM5656195_treat	B
GSM5656199_treat	B
GSM5656200_treat	B
GSM5656203_treat	A
GSM5656207_treat	B
GSM5656209_treat	A
GSM5656212_treat	B
GSM5656214_treat	B
GSM5656217_treat	A
GSM5656220_treat	C
GSM5656223_treat	B
GSM5656226_treat	B
GSM5656228_treat	A
GSM5656231_treat	C
GSM5656235_treat	B
GSM5656237_treat	A
GSM5656240_treat	B
GSM5656243_treat	B
GSM5656246_treat	A
GSM5656249_treat	B
GSM5656254_treat	A
GSM5656255_treat	C
GSM5656259_treat	A
GSM5656262_treat	A
GSM5656264_treat	A
GSM5656265_treat	A
GSM5656267_treat	A
GSM5656273_treat	C
GSM5656274_treat	C
GSM5656277_treat	A

GSM5656280_treat	A
GSM5656282_treat	B
GSM5656285_treat	B
GSM5656291_treat	A
GSM5656294_treat	A
GSM5656297_treat	A
GSM5656303_treat	B
GSM5656305_treat	B
GSM5656309_treat	A
GSM5656311_treat	B
GSM5656316_treat	C
GSM5656318_treat	C
GSM5656321_treat	A
GSM5656323_treat	C
GSM5656325_treat	A
GSM5656327_treat	C
GSM5656329_treat	C
GSM5656334_treat	C
GSM5656335_treat	C
GSM5656338_treat	A
GSM5656342_treat	C
GSM5656346_treat	C
GSM5656348_treat	B
GSM5656351_treat	B
GSM5656353_treat	A
GSM5656355_treat	C
GSM5656359_treat	C
GSM5656361_treat	C
GSM5656365_treat	C
GSM5656366_treat	C
GSM5656368_treat	C
GSM5656372_treat	C
GSM5656374_treat	A
GSM5656375_treat	C
GSM5656380_treat	C
GSM5656381_treat	A
GSM5656384_treat	B
GSM5656387_treat	B
GSM5656388_treat	B
GSM5656390_treat	A
GSM5656392_treat	C
GSM5656393_treat	C
GSM5656395_treat	C

GSM5656399_treat	C
GSM5656400_treat	A
GSM5656403_treat	A
GSM5656406_treat	C
GSM5656407_treat	A
GSM5656409_treat	C
GSM5656412_treat	C
GSM5656416_treat	B
GSM5656418_treat	A
GSM5656421_treat	C
GSM5656424_treat	B
GSM5656426_treat	C
GSM5656429_treat	B
GSM5656431_treat	B
GSM5656432_treat	A
GSM5656434_treat	A
GSM5656435_treat	B
GSM5656439_treat	A
GSM5656443_treat	C
GSM5656444_treat	C
GSM5656447_treat	B
GSM5656451_treat	C
GSM5656452_treat	A
GSM5656454_treat	C
GSM5656458_treat	B
GSM5656460_treat	B
GSM5656461_treat	A
GSM5656464_treat	B
GSM5656465_treat	A
GSM5656467_treat	C
GSM5656469_treat	B
GSM5656473_treat	C
GSM5656478_treat	A
GSM5656481_treat	A
GSM5656483_treat	A
GSM5656486_treat	A
GSM5656487_treat	B
GSM5656490_treat	C
GSM5656493_treat	A
GSM5656495_treat	A
GSM5656498_treat	C
GSM5656501_treat	C
GSM5656504_treat	C

GSM5656505_treat	C
GSM5656509_treat	A
GSM5656511_treat	A
GSM5656513_treat	A
GSM5656516_treat	C
GSM5656517_treat	B
GSM5656520_treat	B
GSM5656521_treat	C
GSM5656522_treat	C
GSM5656524_treat	B
GSM5656525_treat	C
GSM5656527_treat	A
GSM5656528_treat	A
GSM5656531_treat	C
GSM5656533_treat	C
GSM5656535_treat	B
GSM5656536_treat	A
GSM5656541_treat	B
GSM5656543_treat	C
GSM5656545_treat	C
GSM5656549_treat	C
GSM5656551_treat	C
GSM5656554_treat	A
GSM5656556_treat	C
GSM5656559_treat	C
GSM5656563_treat	B
GSM5656565_treat	B
GSM5656567_treat	C
GSM5656569_treat	A
GSM5656571_treat	A
GSM5656573_treat	C
GSM5656576_treat	C
GSM5656578_treat	B
GSM5656581_treat	C
GSM5656583_treat	C
GSM5656585_treat	C
GSM5656588_treat	B
GSM5656589_treat	C
GSM5656591_treat	C
GSM5656595_treat	C
GSM5656596_treat	A
GSM5656600_treat	C
GSM5656601_treat	B

GSM5656602_treat	A
GSM5656603_treat	B
GSM5656604_treat	A
GSM5656606_treat	B
GSM5656608_treat	C
GSM5656611_treat	B
GSM5656612_treat	B
GSM5656615_treat	B
GSM5656617_treat	A
GSM5656619_treat	B
GSM5656621_treat	C
GSM5656624_treat	A
GSM5656625_treat	A
GSM5656627_treat	B
GSM5656631_treat	B
GSM5656632_treat	B
GSM5656635_treat	B
GSM5656637_treat	B
GSM5656639_treat	B
GSM5656641_treat	A
GSM5656645_treat	A
GSM5656647_treat	B
GSM5656648_treat	A
GSM5656653_treat	B
GSM5656655_treat	C
GSM5656657_treat	A
GSM5656173_treat	B
GSM5656182_treat	B
GSM5656191_treat	A
GSM5656194_treat	B
GSM5656196_treat	B
GSM5656198_treat	A
GSM5656205_treat	C
GSM5656210_treat	A
GSM5656211_treat	B
GSM5656215_treat	B
GSM5656218_treat	B
GSM5656219_treat	B
GSM5656222_treat	B
GSM5656227_treat	C
GSM5656234_treat	B
GSM5656236_treat	A
GSM5656239_treat	A

GSM5656241_treat	A
GSM5656244_treat	B
GSM5656247_treat	C
GSM5656251_treat	B
GSM5656253_treat	B
GSM5656256_treat	A
GSM5656263_treat	A
GSM5656268_treat	B
GSM5656272_treat	B
GSM5656275_treat	B
GSM5656278_treat	B
GSM5656281_treat	B
GSM5656286_treat	B
GSM5656289_treat	B
GSM5656293_treat	B
GSM5656296_treat	A
GSM5656301_treat	B
GSM5656307_treat	B
GSM5656308_treat	A
GSM5656320_treat	B
GSM5656324_treat	B
GSM5656326_treat	B
GSM5656328_treat	B
GSM5656331_treat	C
GSM5656332_treat	B
GSM5656336_treat	B
GSM5656340_treat	A
GSM5656343_treat	B
GSM5656345_treat	B
GSM5656349_treat	B
GSM5656350_treat	B
GSM5656354_treat	B
GSM5656357_treat	B
GSM5656362_treat	B
GSM5656363_treat	B
GSM5656367_treat	B
GSM5656370_treat	B
GSM5656371_treat	C
GSM5656373_treat	B
GSM5656377_treat	A
GSM5656378_treat	A
GSM5656379_treat	B
GSM5656382_treat	B

GSM5656386_treat	B
GSM5656391_treat	B
GSM5656397_treat	A
GSM5656401_treat	B
GSM5656402_treat	B
GSM5656404_treat	A
GSM5656408_treat	B
GSM5656411_treat	B
GSM5656413_treat	A
GSM5656415_treat	B
GSM5656419_treat	B
GSM5656422_treat	B
GSM5656427_treat	A
GSM5656433_treat	B
GSM5656436_treat	A
GSM5656438_treat	A
GSM5656440_treat	B
GSM5656442_treat	B
GSM5656446_treat	A
GSM5656449_treat	B
GSM5656453_treat	B
GSM5656456_treat	A
GSM5656457_treat	B
GSM5656459_treat	B
GSM5656462_treat	B
GSM5656466_treat	B
GSM5656468_treat	B
GSM5656472_treat	C
GSM5656475_treat	A
GSM5656476_treat	A
GSM5656479_treat	A
GSM5656484_treat	B
GSM5656491_treat	C
GSM5656494_treat	B
GSM5656497_treat	A
GSM5656499_treat	A
GSM5656502_treat	A
GSM5656507_treat	A
GSM5656510_treat	A
GSM5656512_treat	B
GSM5656515_treat	A
GSM5656519_treat	B
GSM5656523_treat	B

GSM5656526_treat	B
GSM5656529_treat	A
GSM5656538_treat	B
GSM5656540_treat	B
GSM5656544_treat	B
GSM5656547_treat	B
GSM5656550_treat	B
GSM5656552_treat	A
GSM5656553_treat	B
GSM5656557_treat	B
GSM5656560_treat	A
GSM5656561_treat	B
GSM5656566_treat	B
GSM5656568_treat	B
GSM5656572_treat	C
GSM5656574_treat	B
GSM5656577_treat	C
GSM5656580_treat	A
GSM5656582_treat	A
GSM5656586_treat	B
GSM5656587_treat	B
GSM5656592_treat	A
GSM5656593_treat	A
GSM5656598_treat	A
GSM5656605_treat	B
GSM5656607_treat	B
GSM5656610_treat	B
GSM5656613_treat	B
GSM5656616_treat	B
GSM5656618_treat	B
GSM5656626_treat	B
GSM5656628_treat	B
GSM5656630_treat	B
GSM5656633_treat	B
GSM5656636_treat	B
GSM5656638_treat	B
GSM5656640_treat	B
GSM5656642_treat	B
GSM5656644_treat	B
GSM5656646_treat	B
GSM5656650_treat	A
GSM5656651_treat	B
GSM5656654_treat	B

GSM5656658_treat	B
GSM5656170_treat	B
GSM5656172_treat	A
GSM5656176_treat	A
GSM5656178_treat	B
GSM5656181_treat	B
GSM5656188_treat	A
GSM5656197_treat	A
GSM5656201_treat	B
GSM5656204_treat	A
GSM5656206_treat	B
GSM5656213_treat	B
GSM5656216_treat	A
GSM5656221_treat	A
GSM5656224_treat	A
GSM5656225_treat	B
GSM5656229_treat	B
GSM5656233_treat	C
GSM5656242_treat	C
GSM5656248_treat	C
GSM5656250_treat	A
GSM5656252_treat	A
GSM5656257_treat	A
GSM5656260_treat	A
GSM5656261_treat	A
GSM5656266_treat	A
GSM5656269_treat	B
GSM5656276_treat	A
GSM5656284_treat	B
GSM5656290_treat	C
GSM5656295_treat	B
GSM5656298_treat	A
GSM5656302_treat	A
GSM5656306_treat	A
GSM5656312_treat	C
GSM5656315_treat	B
GSM5656317_treat	A
GSM5656319_treat	B
GSM5656322_treat	A
GSM5656330_treat	B
GSM5656333_treat	B
GSM5656337_treat	A
GSM5656339_treat	A

GSM5656341_treat	B
GSM5656344_treat	B
GSM5656347_treat	A
GSM5656352_treat	B
GSM5656356_treat	B
GSM5656358_treat	B
GSM5656360_treat	B
GSM5656364_treat	B
GSM5656369_treat	A
GSM5656376_treat	B
GSM5656383_treat	B
GSM5656385_treat	A
GSM5656389_treat	B
GSM5656394_treat	A
GSM5656396_treat	A
GSM5656398_treat	B
GSM5656405_treat	B
GSM5656410_treat	A
GSM5656414_treat	A
GSM5656417_treat	B
GSM5656420_treat	A
GSM5656423_treat	A
GSM5656425_treat	B
GSM5656428_treat	B
GSM5656430_treat	B
GSM5656437_treat	A
GSM5656441_treat	A
GSM5656445_treat	A
GSM5656448_treat	B
GSM5656450_treat	B
GSM5656455_treat	A
GSM5656463_treat	A
GSM5656470_treat	A
GSM5656471_treat	A
GSM5656474_treat	A
GSM5656477_treat	A
GSM5656480_treat	A
GSM5656482_treat	B
GSM5656485_treat	B
GSM5656488_treat	C
GSM5656489_treat	A
GSM5656492_treat	B
GSM5656496_treat	A

GSM5656500_treat	A
GSM5656503_treat	C
GSM5656506_treat	A
GSM5656508_treat	B
GSM5656514_treat	A
GSM5656518_treat	A
GSM5656530_treat	B
GSM5656532_treat	C
GSM5656534_treat	B
GSM5656537_treat	B
GSM5656542_treat	A
GSM5656546_treat	B
GSM5656548_treat	B
GSM5656555_treat	B
GSM5656558_treat	C
GSM5656562_treat	B
GSM5656564_treat	B
GSM5656570_treat	B
GSM5656575_treat	B
GSM5656579_treat	A
GSM5656584_treat	A
GSM5656590_treat	A
GSM5656594_treat	A
GSM5656597_treat	B
GSM5656599_treat	A
GSM5656609_treat	A
GSM5656614_treat	B
GSM5656620_treat	A
GSM5656622_treat	B
GSM5656623_treat	A
GSM5656629_treat	C
GSM5656634_treat	C
GSM5656643_treat	B
GSM5656649_treat	A
GSM5656652_treat	B
GSM5656656_treat	B
