# Supplementary material for: Prognostic and therapeutic implication of m6A methylation in Crohn disease
Source: Medicine (Baltimore). 2022 Dec 23;101(51):e32399. doi: 10.1097/MD.0000000000032399 (PMC9794314; doi:10.1097/MD.0000000000032399)
Supplement: Supplementary file 11 [file medi-101-e32399-s011.pdf]

Supplemental Table 11. m6Ascore

ID	m6Ascore
GSM5656180_con	-3.564820282
GSM5656185_con	-1.979446692
GSM5656189_con	0.928410925
GSM5656190_con	1.477303951
GSM5656202_con	-2.634473428
GSM5656208_con	0.141088957
GSM5656230_con	1.378125121
GSM5656232_con	-3.695098807
GSM5656238_con	-3.244636659
GSM5656245_con	-3.91001151
GSM5656258_con	-2.85660684
GSM5656270_con	-0.452689683
GSM5656271_con	-3.066674065
GSM5656279_con	-4.24648339
GSM5656283_con	-3.937675166
GSM5656287_con	-3.720595327
GSM5656288_con	-2.770990797
GSM5656292_con	-2.71958852
GSM5656299_con	-2.735832576
GSM5656300_con	-0.492298048
GSM5656304_con	-3.151374591
GSM5656310_con	-2.834791296
GSM5656313_con	-1.920566504
GSM5656314_con	0.41616948
GSM5656539_con	-0.904431193
GSM5656171_treat	-0.594100518
GSM5656174_treat	1.353988868
GSM5656175_treat	-2.568742789
GSM5656177_treat	5.034455597
GSM5656179_treat	-1.598073971
GSM5656183_treat	1.82954602
GSM5656184_treat	-1.990164602
GSM5656186_treat	0.126256728
GSM5656187_treat	2.569246635
GSM5656192_treat	0.860584675
GSM5656193_treat	-1.36386678
GSM5656195_treat	-1.898135717
GSM5656199_treat	1.279810412
GSM5656200_treat	-1.25330923
GSM5656203_treat	1.643550997
GSM5656207_treat	-0.479003723

GSM5656209_treat	-3.241318789
GSM5656212_treat	-1.862863953
GSM5656214_treat	-0.070232362
GSM5656217_treat	-3.562083122
GSM5656220_treat	0.031999816
GSM5656223_treat	-1.390733131
GSM5656226_treat	-0.781471146
GSM5656228_treat	-1.418618678
GSM5656231_treat	2.598612835
GSM5656235_treat	-1.637798615
GSM5656237_treat	1.66166166
GSM5656240_treat	-1.543635782
GSM5656243_treat	-2.905441869
GSM5656246_treat	-1.099824268
GSM5656249_treat	0.302846155
GSM5656254_treat	-2.939744984
GSM5656255_treat	-0.818110147
GSM5656259_treat	-3.23458407
GSM5656262_treat	-0.430455204
GSM5656264_treat	-1.391134907
GSM5656265_treat	-1.089528377
GSM5656267_treat	-1.207960664
GSM5656273_treat	-2.653460852
GSM5656274_treat	-0.534877961
GSM5656277_treat	1.080096218
GSM5656280_treat	2.009038917
GSM5656282_treat	0.162652933
GSM5656285_treat	-0.171733838
GSM5656291_treat	-1.60347063
GSM5656294_treat	-2.004581478
GSM5656297_treat	-2.908219237
GSM5656303_treat	1.543874047
GSM5656305_treat	-0.609685204
GSM5656309_treat	-1.51836177
GSM5656311_treat	-0.263854626
GSM5656316_treat	-0.35777271
GSM5656318_treat	-1.922689256
GSM5656321_treat	-1.356710457
GSM5656323_treat	-3.016457018
GSM5656325_treat	-2.019741162
GSM5656327_treat	-2.55754586
GSM5656329_treat	-2.067965055
GSM5656334_treat	-3.129213365

GSM5656335_treat	-1.529665458
GSM5656338_treat	-1.429905209
GSM5656342_treat	-2.36824322
GSM5656346_treat	-0.14344631
GSM5656348_treat	-0.01173891
GSM5656351_treat	2.726962133
GSM5656353_treat	-1.004269027
GSM5656355_treat	-3.589537148
GSM5656359_treat	-0.479267366
GSM5656361_treat	-1.65698717
GSM5656365_treat	0.061564166
GSM5656366_treat	-2.672653104
GSM5656368_treat	-1.067938243
GSM5656372_treat	1.133403868
GSM5656374_treat	0.034008824
GSM5656375_treat	0.874731344
GSM5656380_treat	-1.110166038
GSM5656381_treat	-1.334026477
GSM5656384_treat	-0.592358391
GSM5656387_treat	-3.060936335
GSM5656388_treat	-1.89915542
GSM5656390_treat	6.236808728
GSM5656392_treat	-2.610329333
GSM5656393_treat	3.906332588
GSM5656395_treat	-3.901913626
GSM5656399_treat	-2.305487302
GSM5656400_treat	1.658773277
GSM5656403_treat	1.366164798
GSM5656406_treat	0.797791989
GSM5656407_treat	1.184662421
GSM5656409_treat	-2.906807225
GSM5656412_treat	-0.30990641
GSM5656416_treat	-0.715377075
GSM5656418_treat	1.043571713
GSM5656421_treat	-1.923648227
GSM5656424_treat	-0.03343426
GSM5656426_treat	2.74784888
GSM5656429_treat	1.766324176
GSM5656431_treat	-2.004529057
GSM5656432_treat	-1.731382863
GSM5656434_treat	-0.241957378
GSM5656435_treat	0.235768525
GSM5656439_treat	0.861969302

GSM5656443_treat	0.618130401
GSM5656444_treat	2.131473407
GSM5656447_treat	0.845381237
GSM5656451_treat	2.810705808
GSM5656452_treat	2.250779869
GSM5656454_treat	4.698910006
GSM5656458_treat	-0.26285654
GSM5656460_treat	-2.077134703
GSM5656461_treat	3.486899674
GSM5656464_treat	2.466063903
GSM5656465_treat	-0.643507396
GSM5656467_treat	-0.439636625
GSM5656469_treat	0.405311185
GSM5656473_treat	-2.067014357
GSM5656478_treat	-0.849446786
GSM5656481_treat	1.252265019
GSM5656483_treat	3.791112711
GSM5656486_treat	-0.718448432
GSM5656487_treat	5.38988464
GSM5656490_treat	1.325142096
GSM5656493_treat	-2.678645738
GSM5656495_treat	-2.265729921
GSM5656498_treat	-1.573402804
GSM5656501_treat	0.548342963
GSM5656504_treat	1.659204684
GSM5656505_treat	-2.084707957
GSM5656509_treat	0.176569667
GSM5656511_treat	2.521791348
GSM5656513_treat	-1.477967007
GSM5656516_treat	0.082294266
GSM5656517_treat	2.092667616
GSM5656520_treat	-4.882487594
GSM5656521_treat	-0.037977002
GSM5656522_treat	-0.02498954
GSM5656524_treat	0.583518169
GSM5656525_treat	-2.180116305
GSM5656527_treat	-0.101704438
GSM5656528_treat	1.128765047
GSM5656531_treat	0.484961009
GSM5656533_treat	-0.277994525
GSM5656535_treat	-3.376285827
GSM5656536_treat	-1.258239247
GSM5656541_treat	-1.269668229

GSM5656543_treat	-1.895049459
GSM5656545_treat	-0.971526929
GSM5656549_treat	-2.95007674
GSM5656551_treat	-2.593995621
GSM5656554_treat	-0.861373403
GSM5656556_treat	1.926478227
GSM5656559_treat	-3.428592586
GSM5656563_treat	-1.262228741
GSM5656565_treat	-1.535042793
GSM5656567_treat	-3.213660542
GSM5656569_treat	-2.890890209
GSM5656571_treat	-1.873842697
GSM5656573_treat	-1.817091876
GSM5656576_treat	-4.570194513
GSM5656578_treat	-0.980955063
GSM5656581_treat	-0.99857938
GSM5656583_treat	-2.908667555
GSM5656585_treat	-4.7731139
GSM5656588_treat	-1.132589559
GSM5656589_treat	-2.245742232
GSM5656591_treat	-3.769292666
GSM5656595_treat	-0.99849233
GSM5656596_treat	-0.976622233
GSM5656600_treat	1.549021685
GSM5656601_treat	-1.584984502
GSM5656602_treat	0.287474916
GSM5656603_treat	-0.217378737
GSM5656604_treat	-1.966999569
GSM5656606_treat	-4.316455471
GSM5656608_treat	-1.989756202
GSM5656611_treat	-2.033435268
GSM5656612_treat	1.017168133
GSM5656615_treat	-1.419232972
GSM5656617_treat	0.071860554
GSM5656619_treat	-2.949675312
GSM5656621_treat	-0.800587323
GSM5656624_treat	-0.737789155
GSM5656625_treat	-1.483254799
GSM5656627_treat	-2.296450112
GSM5656631_treat	0.336941677
GSM5656632_treat	-0.83047748
GSM5656635_treat	-0.167278232
GSM5656637_treat	1.257638904

GSM5656639_treat	-1.187543053
GSM5656641_treat	-2.379166074
GSM5656645_treat	-2.359154417
GSM5656647_treat	1.068583849
GSM5656648_treat	-1.705273235
GSM5656653_treat	-0.622945918
GSM5656655_treat	-2.065188601
GSM5656657_treat	-1.019761442
GSM5656173_treat	1.149642556
GSM5656182_treat	-0.505010272
GSM5656191_treat	-2.281734848
GSM5656194_treat	-1.128724202
GSM5656196_treat	-1.233425613
GSM5656198_treat	2.042145002
GSM5656205_treat	1.778031913
GSM5656210_treat	2.104518272
GSM5656211_treat	3.135019157
GSM5656215_treat	0.40566163
GSM5656218_treat	-1.821082825
GSM5656219_treat	3.11702869
GSM5656222_treat	-0.96120623
GSM5656227_treat	0.192260681
GSM5656234_treat	-0.864523668
GSM5656236_treat	6.488933879
GSM5656239_treat	-1.860760051
GSM5656241_treat	-0.801439051
GSM5656244_treat	-1.38650401
GSM5656247_treat	-1.688222285
GSM5656251_treat	3.92265265
GSM5656253_treat	-2.798441375
GSM5656256_treat	-0.045422455
GSM5656263_treat	0.256054891
GSM5656268_treat	2.190236577
GSM5656272_treat	1.556409279
GSM5656275_treat	-0.969865411
GSM5656278_treat	-0.625022183
GSM5656281_treat	1.129209052
GSM5656286_treat	-0.429380458
GSM5656289_treat	-0.31959912
GSM5656293_treat	0.473634307
GSM5656296_treat	-1.123194977
GSM5656301_treat	0.718128829
GSM5656307_treat	-1.063735572

GSM5656308_treat	-2.559876969
GSM5656320_treat	-0.405956047
GSM5656324_treat	-1.534233521
GSM5656326_treat	-0.809514164
GSM5656328_treat	0.238627231
GSM5656331_treat	-3.12030304
GSM5656332_treat	-1.632085427
GSM5656336_treat	-0.239375124
GSM5656340_treat	0.323369224
GSM5656343_treat	-1.76216641
GSM5656345_treat	0.103252196
GSM5656349_treat	-0.631648323
GSM5656350_treat	1.892510316
GSM5656354_treat	-0.2023347
GSM5656357_treat	-0.831283043
GSM5656362_treat	-1.975433742
GSM5656363_treat	-0.657251842
GSM5656367_treat	-1.783722993
GSM5656370_treat	0.047608227
GSM5656371_treat	0.373056467
GSM5656373_treat	-0.442879946
GSM5656377_treat	1.376534412
GSM5656378_treat	1.895875065
GSM5656379_treat	4.895175486
GSM5656382_treat	1.590188417
GSM5656386_treat	3.492034104
GSM5656391_treat	2.850117531
GSM5656397_treat	-1.836386176
GSM5656401_treat	-0.006726694
GSM5656402_treat	0.250689215
GSM5656404_treat	4.479340213
GSM5656408_treat	0.596560746
GSM5656411_treat	1.533729726
GSM5656413_treat	3.793255977
GSM5656415_treat	3.396534781
GSM5656419_treat	-1.062845537
GSM5656422_treat	-1.260056883
GSM5656427_treat	3.888946072
GSM5656433_treat	-0.16625052
GSM5656436_treat	2.976605704
GSM5656438_treat	4.196889988
GSM5656440_treat	2.324499359
GSM5656442_treat	2.269270402

GSM5656446_treat	4.095646654
GSM5656449_treat	2.804177416
GSM5656453_treat	2.42981788
GSM5656456_treat	2.297702136
GSM5656457_treat	0.77085921
GSM5656459_treat	-2.469644429
GSM5656462_treat	1.406050015
GSM5656466_treat	0.146292936
GSM5656468_treat	-1.006552566
GSM5656472_treat	-3.23635066
GSM5656475_treat	-2.47574182
GSM5656476_treat	0.774831316
GSM5656479_treat	2.370568574
GSM5656484_treat	-0.05600672
GSM5656491_treat	0.77269479
GSM5656494_treat	3.983104451
GSM5656497_treat	1.639356976
GSM5656499_treat	0.343816787
GSM5656502_treat	1.977958396
GSM5656507_treat	3.460279475
GSM5656510_treat	2.191873064
GSM5656512_treat	1.039055959
GSM5656515_treat	0.020754154
GSM5656519_treat	-0.583175289
GSM5656523_treat	-0.031966184
GSM5656526_treat	-0.889658423
GSM5656529_treat	3.243623264
GSM5656538_treat	0.649830148
GSM5656540_treat	-0.270903648
GSM5656544_treat	0.754998726
GSM5656547_treat	-1.646804768
GSM5656550_treat	-3.197524547
GSM5656552_treat	-0.196705958
GSM5656553_treat	0.346686121
GSM5656557_treat	1.701733079
GSM5656560_treat	1.263977407
GSM5656561_treat	0.267769692
GSM5656566_treat	-1.554941173
GSM5656568_treat	-0.897796763
GSM5656572_treat	1.312086396
GSM5656574_treat	-0.698170761
GSM5656577_treat	0.211676469
GSM5656580_treat	0.56860653

GSM5656582_treat	-3.161026344
GSM5656586_treat	-2.686565205
GSM5656587_treat	-1.22309594
GSM5656592_treat	-3.977529438
GSM5656593_treat	-0.515225086
GSM5656598_treat	0.799649982
GSM5656605_treat	-0.622185672
GSM5656607_treat	-0.942097488
GSM5656610_treat	-1.353073458
GSM5656613_treat	-0.529221014
GSM5656616_treat	0.271026925
GSM5656618_treat	-1.451287034
GSM5656626_treat	0.478189568
GSM5656628_treat	1.253939079
GSM5656630_treat	0.743364647
GSM5656633_treat	0.411581535
GSM5656636_treat	2.269029811
GSM5656638_treat	-0.892040462
GSM5656640_treat	-0.614792035
GSM5656642_treat	-1.306634952
GSM5656644_treat	-2.84697201
GSM5656646_treat	0.695976482
GSM5656650_treat	1.421062101
GSM5656651_treat	-1.122472378
GSM5656654_treat	1.744215027
GSM5656658_treat	-0.094546626
GSM5656170_treat	-0.03479625
GSM5656172_treat	-1.491515669
GSM5656176_treat	0.809401733
GSM5656178_treat	-0.029083347
GSM5656181_treat	3.117569554
GSM5656188_treat	2.195962815
GSM5656197_treat	4.100659088
GSM5656201_treat	-2.835752774
GSM5656204_treat	0.737918337
GSM5656206_treat	0.944863236
GSM5656213_treat	-2.099564608
GSM5656216_treat	-1.327791456
GSM5656221_treat	4.450366626
GSM5656224_treat	-2.386899641
GSM5656225_treat	-0.329197215
GSM5656229_treat	-2.369457191
GSM5656233_treat	-2.752250101

GSM5656242_treat	-3.809595596
GSM5656248_treat	-3.282553406
GSM5656250_treat	-0.716582816
GSM5656252_treat	4.114606723
GSM5656257_treat	-0.136357488
GSM5656260_treat	3.423238398
GSM5656261_treat	-3.192506313
GSM5656266_treat	-1.168939628
GSM5656269_treat	-2.297499578
GSM5656276_treat	0.63161679
GSM5656284_treat	-1.486590803
GSM5656290_treat	-4.665682238
GSM5656295_treat	-1.557699124
GSM5656298_treat	-2.23666173
GSM5656302_treat	3.105486191
GSM5656306_treat	-2.063658478
GSM5656312_treat	0.509450712
GSM5656315_treat	-1.533611048
GSM5656317_treat	5.584891856
GSM5656319_treat	-1.107998568
GSM5656322_treat	-0.988936444
GSM5656330_treat	-1.969052834
GSM5656333_treat	1.384881911
GSM5656337_treat	4.581556723
GSM5656339_treat	-1.154200442
GSM5656341_treat	-1.264308462
GSM5656344_treat	0.772446194
GSM5656347_treat	3.83672416
GSM5656352_treat	-0.936593392
GSM5656356_treat	-1.314401157
GSM5656358_treat	-1.379852227
GSM5656360_treat	0.524160767
GSM5656364_treat	0.503040755
GSM5656369_treat	-0.408456976
GSM5656376_treat	-3.463677095
GSM5656383_treat	1.815183524
GSM5656385_treat	0.66595578
GSM5656389_treat	2.280906007
GSM5656394_treat	5.860617242
GSM5656396_treat	0.48062497
GSM5656398_treat	3.957656935
GSM5656405_treat	1.524491229
GSM5656410_treat	1.311591033

GSM5656414_treat	-1.462028396
GSM5656417_treat	2.116231344
GSM5656420_treat	3.741281114
GSM5656423_treat	-2.318721132
GSM5656425_treat	0.901196077
GSM5656428_treat	4.348420861
GSM5656430_treat	-0.488711785
GSM5656437_treat	-1.849134754
GSM5656441_treat	2.849627885
GSM5656445_treat	4.165050404
GSM5656448_treat	0.445404332
GSM5656450_treat	5.377327187
GSM5656455_treat	2.816315757
GSM5656463_treat	2.219595619
GSM5656470_treat	4.659318373
GSM5656471_treat	1.60247945
GSM5656474_treat	2.239247542
GSM5656477_treat	4.261184689
GSM5656480_treat	4.080980548
GSM5656482_treat	-0.311387487
GSM5656485_treat	3.351627614
GSM5656488_treat	3.477279678
GSM5656489_treat	1.806215822
GSM5656492_treat	-1.377222575
GSM5656496_treat	2.151800811
GSM5656500_treat	6.098670351
GSM5656503_treat	4.707900283
GSM5656506_treat	4.489993839
GSM5656508_treat	-0.187583222
GSM5656514_treat	6.917791364
GSM5656518_treat	4.716344748
GSM5656530_treat	2.436082925
GSM5656532_treat	1.338879292
GSM5656534_treat	0.058392614
GSM5656537_treat	2.270881636
GSM5656542_treat	5.143797882
GSM5656546_treat	-0.434146155
GSM5656548_treat	-2.383027815
GSM5656555_treat	2.342810121
GSM5656558_treat	5.845868117
GSM5656562_treat	1.788946762
GSM5656564_treat	1.302625572
GSM5656570_treat	1.627518503

GSM5656575_treat	0.656786932
GSM5656579_treat	3.311497952
GSM5656584_treat	0.684887226
GSM5656590_treat	6.329234467
GSM5656594_treat	5.867710521
GSM5656597_treat	-0.357958403
GSM5656599_treat	2.449885002
GSM5656609_treat	-1.021046415
GSM5656614_treat	-1.405134195
GSM5656620_treat	4.306584873
GSM5656622_treat	1.712329781
GSM5656623_treat	3.547834567
GSM5656629_treat	4.290538939
GSM5656634_treat	-0.036150166
GSM5656643_treat	3.522732329
GSM5656649_treat	-0.460650578
GSM5656652_treat	-0.462882227
GSM5656656_treat	-1.716789089
